# Description of the updated nutrition calculation of the Oxford WebQ questionnaire and comparison with the previous version among 207,144 participants in UK Biobank

**DOI:** 10.1007/s00394-021-02558-4

**Published:** 2021-05-06

**Authors:** Aurora Perez-Cornago, Zoe Pollard, Heather Young, Marloes van Uden, Colm Andrews, Carmen Piernas, Timothy J. Key, Angela Mulligan, Marleen Lentjes

**Affiliations:** 1 Cancer Epidemiology Unit, Nuffield Department of Population Health, University of Oxford, Richard Doll Building, Roosevelt Drive, Oxford, OX3 7LF UK; 2 Department of Public Health & Primary Care, Institute of Public Health, University of Cambridge, Cambridge, UK; 3 Nuffield Department of Primary Care Health Sciences, University of Oxford, Oxford, UK; 4 NIHR BRC Diet, Anthropometry and Physical Activity Group, MRC Epidemiology Unit, University of Cambridge, Cambridge, UK; 5 School of Medical Sciences, Clinical Epidemiology and Biostatistics, Örebro University, Örebro, Sweden

**Keywords:** Online 24-h dietary assessment, Oxford WebQ, UK Biobank, Comparative study, Food composition table

## Abstract

**Purpose:**

The Oxford WebQ is a web-based 24-h dietary assessment method which has been used in UK Biobank and other large prospective studies. The food composition table used to calculate nutrient intakes has recently been replaced with the UK Nutrient Databank, which has food composition data closer in time to when participants completed the questionnaire, and new dietary variables were incorporated. Here we describe the updated version of the Oxford WebQ questionnaire nutrient calculation, and compare nutrient intakes with the previous version used.

**Methods:**

207,144 UK Biobank participants completed ≥ 1 Oxford WebQs, and means and standard deviations of nutrient intakes were averaged for all completed 24-h dietary assessments. Spearman correlations and weighted kappa statistics were used to compare the re-classification and agreement of nutrient intakes between the two versions.

**Results:**

35 new nutrients were incorporated in the updated version. Compared to the previous version, most nutrients were very similar in the updated version except for a few nutrients which showed a difference of > 10%: lower with the new version for trans-fat (− 20%), and vitamin C (− 15%), but higher for retinol (+ 42%), vitamin D (+ 26%) and vitamin E (+ 20%). Most participants were in the same (> 60%) or adjacent (> 90%) quintile of intake for the two versions. Except for trans-fat (*r* = 0.58, *κ* = 0.42), very high correlations were found between the nutrients calculated using the two versions (*r* > 0.79 and *κ* > 0.60).

**Conclusion:**

Small absolute differences in nutrient intakes were observed between the two versions, and the ranking of individuals was minimally affected, except for trans-fat.

**Supplementary Information:**

The online version contains supplementary material available at 10.1007/s00394-021-02558-4.

## Introduction

Traditional methods to determine dietary intake in large prospective studies, such us paper-based food frequency questionnaires (FFQ) and/or interviewer administered 24-h recalls, are costly and time-consuming. Recently, self-administered online 24-h dietary assessments have been incorporated in some large prospective studies and been shown to facilitate data analyses and decrease the researcher burden, including data entry and data coding, by automatically calculating nutrient intakes [1].

The Oxford WebQ is a fully automated web-based 24-h dietary assessment tool which seeks information from participants about their consumption of food and drink during the previous 24 h [2]. This online questionnaire has already been used by several large-scale cohort studies, such us the UK Biobank [3] and the Million Women Study [4], as it is easy and quick (~ 12 min) to self-complete and suitable for repeated use in large-scale prospective studies. Moreover, nutrients are automatically estimated via built-in algorithms and food composition data. Until now, the food composition table (FCT) used for the Oxford WebQ has been the UK McCance and Widdowson’s “The Composition of Foods 6th edition (2002) and its supplements [5–15], of which 550 of 1200 foods were incorporated into the Oxford WebQ. This FCT has now been replaced by the UK Nutrient Databank (UKNDB) (2013), which provides food composition data measured closer in time to when participants completed the questionnaire in UK Biobank (2009–2012) and contains over 5600 foods, of which 681 food codes have been incorporated into the Oxford WebQ [16, 17]. The UKNDB is commissioned by Public Health England as part of the National Diet and Nutrition Survey (NDNS), and is available in electronic format as an integrated dataset, and contains up-to-date nutrient composition data. Data in the UKNDB are very similar to the UK McCance and Widdowson’s FCT but includes a larger range of processed foods and composite dishes and missing values were reviewed and replaced with plausible values and it is maintained as part of NDNS. As well as replacing the FCT used to calculate nutrient intakes, we have made other changes such as some changes in portion sizes, personalisation of fats used in cooking, and updating the underlying program code for the nutrient calculation, and new dietary variables such as energy density, and animal and plant fats and proteins, have been incorporated. This paper describes the main changes made to nutrient estimation for the Oxford WebQ questionnaire, and compares the two versions of obtained nutrient intakes in over 200,000 UK Biobank participants.

## Methods

### Study design

UK Biobank includes a total of 211,031 participants aged 40–69 years who have completed the Oxford WebQ dietary assessment at least once between 2009 and 2012. Details about the UK Biobank study can be found elsewhere [3]. Briefly, participants provided detailed information on a range of sociodemographic, physical, lifestyle, and health-related factors via self-completed touch-screen questionnaires and a computer-assisted personal interview at recruitment [3].

The study protocol and information about data access are available online (http://www.ukbiobank.ac.uk/wp-content/uploads/2011/11/UK-Biobank-Protocol.pdf) and in the literature [18].

### Dietary assessment—the Oxford WebQ questionnaire

The Oxford WebQ questionnaire was developed to obtain information on the quantities of up to 206 types of foods and 32 types of drinks consumed over the previous day (24 h; https://biobank.ctsu.ox.ac.uk/crystal/crystal/docs/DietWebQ.pdf) [2]. The quantity of each food or drink consumed is calculated by multiplying the assigned portion size (Supplementary Table 1) of each food or beverage by the amount consumed [19]. This questionnaire has recently been validated; compared to recovery biomarkers for energy, protein and potassium, and was considered to perform well in approximating true dietary intake [20]. This questionnaire also provided similar mean estimates of energy and nutrient intakes when compared with an interviewer administered 24-h dietary recall [2]. Further information about the Oxford WebQ can be found here https://www.ceu.ox.ac.uk/research/oxford-webq.

For the previous version of calculating nutrient intakes for the Oxford WebQ, the UK McCance and Widdowson’s 6th edition (2002) FCT and its supplements were used [2]. The nutrients determined were total energy intake, total protein, total fat, saturated fatty acids (SFA), monounsaturated fatty acids (MUFA), polyunsaturated fatty acids (PUFA), cholesterol, carbohydrates, total sugars, fibre, alcohol, calcium, iron, magnesium, potassium, carotene, vitamin B6, vitamin B12, vitamin C, vitamin D and vitamin E. Details about the nutrient calculation can be found in Supplementary Table 2. Trans fatty acids (TFA) and retinol in the previous version of the nutrient calculation were excluded since there were multiple food codes with missing values; for the purpose of comparison, illustration of the consequences of missing data, and because TFA have a public health impact, we are however presenting the results from the previous calculation here.

For the updated version of the nutrient calculation of the Oxford WebQ, nutrient intakes were calculated using the UKNDB FCT from survey year 6, which includes FCT for years 2012–2013 and 2013–2014. Moreover, changes in allocated portion sizes, personalisation of milk types and fats used in cooking, gluten-free versions and the underlying code for nutrient calculation were revised and updated (details in Table 1 and Supplementary Tables 2, 3). Except for total PUFA, all the nutrients available in the previous version are also available in the UKNDB (and total PUFA can be calculated by adding n-3 and n-6 PUFA). Moreover, the following further dietary variables are now available: energy density, animal protein, plant protein, animal fat, plant fat, MUFA, n-3 PUFA, n-6 PUFA, free sugars, non-free sugars, non-milk extrinsic sugars, intrinsic and milk sugars, fructose, glucose, sucrose, lactose, maltose, other sugars, alpha-carotene, beta-carotene, beta cryptoxanthin, vitamin a (retinol equivalents), biotin, chloride, copper, haem iron, non-haem iron, iodine, manganese, sodium, niacin equivalent, pantothenic acid, selenium, total nitrogen and zinc.

**Table 1:** Major changes between the previous (McCance and Widdowson) version and the updated (Nutrient databank + other changes) version

Item	Changes made to the updated version (Nutrient databank + other changes)
Portion size	Some food items had their serving size changed to better reflect what an average portion size would be, taking into account how the question was asked (e.g. Yorkshire pudding). Some portion sizes were revised based on published data (e.g. spreads). Some portion sizes were changed to reflect the state of the food item (e.g. edible part of fruit, or inclusion of liquid for powdered items). These changes can be found in Supplementary Table 1
Milk type	We have now taken into account each milk type beyond fat content, including cholesterol lowering milk, goat’s or sheep’s milk, powdered milk, rice, oat, almond, coconut milk, fortified soya milk, unfortified soya milk, other milk (e.g. lactose free) as well as skimmed, semi skimmed and whole milk This is now applied to all hot drinks where milk is added (i.e. tea, coffee, cappuccino, latte, hot chocolate), milk-based sauces, porridge, crepes and pancakes/blinis
Type of fat used in cooking vegetables	Participants were asked to select the type of fat/oil, if any, they use in the cooking, and a total 40 different types of fat/oils were available. We have now added an amount of fat/oils in certain vegetables such as onion, mushroom, mixed veg, peppers, courgette, leek, parsnip, veg other and mashed potato which are likely to be cooked with oils/fats. These fats/oils include: Butters, spreadable butters, hard margarine, lard, dairy spreads, polyunsaturated margarines, cholesterol lowering margarines, olive oil-based spreads, soya spreads, olive oil, rapeseed oil, sunflower oil, vegetable oil
Gluten-free versions	We have added a gluten-free version where available (e.g. for baguettes, bread rolls, sliced bread, and pasta)
Powdered milk	A water code was added to powdered milk codes so the food volume fits with the way the food is served (important in relation to e.g. energy density)
‘Other’ items	We studied the free text entered by the UK Biobank participants and where possible mapped the ‘other’ items against commonly entered foods (i.e. according to the participants’ understanding of the questions). Whereas previously, these were mapped against a more generic item or a selection of items which were truly different from the specific items listed due to lack of a suitable food code

Further details about these changes can be found in Supplementary Table 1

### Updated nutrient calculation in the Oxford WebQ questionnaire

#### Step 1: Selection of UK Nutrient Databank

The 7th edition of the McCance and Widdowson’s Composition of Foods (abbreviated with CoF) and the UK Nutrient Databank FCT (UKNDB) were considered as possible replacements of the previous FCT. We decided to use the UK Nutrient Databank because missing values were reviewed and replaced with plausible values and it is maintained as part of the National Diet and Nutrition Survey and is updated annually. We used the FCT from survey year 6 as it includes the food composition tables for years 2012–2013 and 2013–2014 [16].

#### Step 2: Changes in the nutrient calculation

Together with changing all the food codes to equivalent food codes from the UKNDB, we reviewed all the portion sizes and took into account the milk type, fats for cooking vegetables, and gluten-free foods in this updated version.

Food codes: We incorporated food codes that better reflected the WebQ item reported by the participants by looking at how each question was asked in the Oxford WebQ questionnaire. Each WebQ item resulted in up to 11 different food codes, with percentages being assigned to each food code (e.g. the food codes used for grapes are 50% black/red grapes and 50% green grapes; see Supplementary Table 2 for details). Unless the WebQ item was specified to be fortified, non-fortified food items were selected. Non-specific answer choices are now mapped to food items reflecting the most likely food choices in the UK biobank population.

Portion sizes: All the portion sizes were revised and updated if required. For this, we took into account how each question was asked to try to understand what the participant may have understood a portion size was, and we also used UK standard portion sizes [19] and product information on packaging from different UK online supermarkets. The changes in portion sizes can be found in Supplementary Table 1.

Milk type: Participants were asked “which type of milk did you use most frequently yesterday?” We have taken into account each milk type including cholesterol lowering milk, goat’s or sheep’s milk, powdered milk, rice, oat, almond, coconut milk, fortified soya milk, unfortified soya milk, other milk (e.g. lactose free) as well as skimmed, semi skimmed and whole milk. We incorporated this into tea, different types of coffee, hot chocolate, milk based sauces, porridge, crepes and pancakes/blinis. A small amount of water was added to the WebQ item of coffee latte and cappuccino to account for the foam in these types of coffees.

Personalisation of fats used in cooking vegetables: Participants were asked “which types of butter, margarine or oil, were used in cooking your food yesterday?” We have taken into account the 40 different types of fat/oils used in the cooking where available and added an amount of fat/oils to certain vegetables: onions, mushrooms, miscellaneous vegetable pieces, peppers, courgette, leek, parsnip, other/unspecified vegetables and mashed potato which are likely to be cooked with oils/fats. These fats/oils include: Butters, spreadable butters, hard margarine, lard, dairy spreads, polyunsaturated margarines, cholesterol lowering margarines, olive oil-based spreads, soya spreads, olive oil, rapeseed oil, sunflower oil, and vegetable oil.

Gluten-free versions: Participants were asked whether they follow a special diet, and this included gluten-free diets. We have added a gluten-free version where available (e.g. for baguettes, bread rolls, large baps, sliced bread, sweet biscuits, scones, pasta). Supplementary Table 1 indicates for which food codes this was not available, and, therefore, the nutrients are the same as the gluten version.

Powdered milk in cereal, and in a glass: A water code has been added to these dried food codes to be “made up” and to account for food volume fitting in better with the portion sizes.

#### Step 3: New dietary variables

Energy density*:* Energy density was calculated for all foods except beverages by dividing total food energy (kJ) by total food weight (g) [21].

Animal and plant protein: The amount of animal and plant protein in each food item was determined examining the food sources.

Animal and plant fat: The amount of animal and plant fat in each food item was determined examining the food sources.

Free sugars: Foods and drinks were classified as containing free sugars (all monosaccharides and disaccharides added to foods by the manufacturer, cook or consumer, plus sugars naturally present in honey, syrups and unsweetened fruit juices) based on the Scientific Advisory Committee on Nutrition (SACN) in the UK definitions [22].

Non-free sugars: Non-free sugars were calculated as the difference between total sugars and free sugars.

Other dietary variables: 24 dietary variables available in the Nutrient databank resource were incorporated (full list of nutrients in Table 4).

#### Step 4: Output and calculation of nutrient intakes

Nutrient intakes per 100 g were calculated for each food item in the questionnaire (Supplementary Table 2). The following assumptions were made when calculating nutrient intakes:

For unanswered questions, it was assumed that the participant did not consume that food.

For spread on bread:

- If no thickness was selected, medium was assumed.
- Participants are required to select at least one spread type. If multiple are selected, then equal proportions from the portion size selected are assigned (e.g. 1 portion of spread in baguette, 50% to butter spreadable and 50% to margarine).
- If no spread sub options were selected (for those spreads with sub options), “don’t know” was assumed.

Like the spreads, other items with multiple sub options (such as glass size for wine, ingredient type in soup, flour type for bread), were given an equal proportion per sub option (e.g. 2 bowls of soup with meat and vegetable ingredients selected, then that would be treated as 1 bowl of meat soup and 1 bowl of vegetable soup).

For meat: for most meat questions, there is a compulsory question about removing the fat. If “don’t know” or “varied” were selected, then half the number of servings were assigned to codes of meat with fat not removed, and half of serving were assigned to codes of meat with fat removed.

Similarly for chicken/turkey, there is a compulsory question about removing the skin. If “don’t know” or “varied” were selected, then half the number of servings were assigned to codes of poultry with skin left on, and half of serving were assigned to codes of poultry with skin removed.

For items that included a question on sugar (cereal, tea and coffee), if “varied” was selected, then 1tsp of sugar was assumed.

For breakfast cereals, the following questions is asked “Did your cereal contain any dried fruit?” If “varied” is selected, then half the number of servings were assigned to codes of breakfast cereals with dried fruit, and half of serving were assigned to codes of breakfast cereals without dried fruit.

Similarly, for other items in which “varied” was an option (i.e. decaf status for black tea/coffee, whether milk was added to cereal, tea or coffee), varied was treated as half with and half without.

For wine, if no glass size was selected, medium was assumed.

For porridge, if neither “made with milk” nor “made with water” were selected, then it was handled as half milk and half water.

Similarly for yogurt, if neither “full fat” nor “low fat” were selected, then it was handled as half full fat and half low fat yogurt.

#### Quality assessment

Five researchers were involved in the quality assessment. The first version of the matching of the foods in the questionnaire with the food codes in the UKNDB and the updated version of the portion sizes was done separately by two researchers (AM, ML), and inconsistencies were discussed; MU also contributed to this initial update. A third researcher (APC) reviewed all the food matching and portion sizes, suggested changes to the portion sizes, food codes, and fractions assigned to each food code, and further modifications were made after discussion with the other researchers (AM, ML, APC, HY). Each food item in the nutrient calculation file was comprehensively checked, and the amounts of each nutrient within each food item was compared with the amounts in the previous version of the nutrient calculation file (ZP, APC; Supplementary Table 3). Where more than 10% difference in nutrient intakes were found, these food codes were further reviewed and discussed with the other researchers, explanations for these changes were found, and where necessary the food codes or portion sizes were changed. HY helped with the overall quality check of this updated version, reviewing it, incorporating it into the database and identifying problems such as detecting the fractions of each food code that did not add up to 100% or verifying that the food codes selected did not have any missing nutrient values. After all these quality controls, APC, ZP, AM, and ML reviewed independently the final version of the nutrient calculation file (Supplementary Tables 1 and 2).

The new variables (energy density, animal and plant protein, animal and plant fat, free sugars, and non-free sugars) were determined separately by APC, ZP and CP, inconsistencies were discussed and the necessary changes were made.

### Participants

A subsample of UK Biobank participants recruited towards the end of the recruitment period (from April 2009 to September 2010) was invited to complete the Oxford WebQ questionnaire. Moreover, those who provided email addresses were invited to complete the Oxford WebQ a total of four times every 3–4 months on variable days of the week during the follow-up period (online cycle 1, February 2011 to April 2011; online cycle 2, June 2011 to September 2011; online cycle 3, October 2011 to December 2011; online cycle 4, April 2012 to June 2012). 24-h dietary assessments with extreme energy intakes (men: < 3347 or > 17,573 kJ/days or < 800 or > 4200 kcal/days); women: < 2092 or > 14,644 kJ/days or < 600 or > 3500 kcal/days) [23] as calculated with either version of the FCT, were excluded. For this reason, 3887 participants were excluded because they did not have a valid WebQ. In this analysis, we are not interested in usual intakes for individuals but in comparing the estimates of intakes of the participants in the completed 24-h dietary assessments; therefore, we have not excluded participants with only one dietary assessment. However, researchers using this dietary assessment tool for diet–disease associations are advised to use at least two 24-h dietary assessments(but more if possible), since intakes from one 24-h dietary assessment are unlikely to reflect usual intakes[20]. A total of 207,144 (out of 211,031, 98%) participants were included in this study.

### Statistical analyses

The WebQ results were averaged for all completed 24-h dietary questionnaire for each participant. Means, standard deviations (SDs), and the 5th and 95th percentiles of nutrient intakes are given. The differences and percentage difference (see equation) in nutrient intakes between the previous and the updated version of the nutrient calculation were determined, and means were compared using paired *t* tests or Wilcoxon's rank sum test, depending on the normality of the distribution.

$$\mathrm{\% difference}=\frac{\left(\mathrm{updated}-\mathrm{previous}\right)}{\mathrm{previous}}\times 100\%.$$

The Spearman correlations of the nutrient data were calculated. Participants were divided into fifths of intake for each nutrient in the two versions of the nutrient calculation and weighted kappa statistics and the percentage of participants who were categorised into the same or adjacent fifth were calculated, since most prospective studies on diet and disease risk examine associations by comparing disease incidence in categories of the dietary factor of interest. Weighted kappas should be interpreted as follows: values ≤ 0 indicates no agreement, 0.01–0.20 as none to slight, 0.21–0.40 as fair, 0.41–0.60 as moderate, 0.61–0.80 as substantial, and 0.81–1.00 as almost perfect agreement [24].

All analyses were conducted using the STATA statistical software package version 14 (Stata Corporation, College Station, TX, USA).

## Results

The mean age at recruitment was 56 years (SD 8) and 55% were women. Participants completed on average 2.14 (SD 1.16) 24-h dietary assessments. Table 2 shows the mean, median, percentiles, and mean differences of energy and nutrient intakes in the two versions. There were small but significant differences (likely due to the large sample size) in the mean nutrient intakes between the existing version and the updated version. Compared to the previous version, intakes in the updated version were > 10% different for the following nutrients: lower for TFA (− 20%), vitamin C (− 15%) and iron (− 9.5%), but higher for retinol (+ 42%), vitamin D (+ 26%) and vitamin E (+ 20%). SFA and TFA intakes provided 12.4% and 0.63% from total energy intake in the previous version of the nutrient calculation, while they provided 11.6% and 0.52% respectively in the updated version.

**Table 2:** Comparison of total energy and nutrient intake between previous (McCance and Widdowson) and updated (Nutrient databank + other updates) datasources in 207,144 participants from UK Biobank

Nutrient	Previous version: McCance and Widdowson				Updated version: Nutrient databank				Mean difference^a^	
	Mean (SD)	Median	5th percentile	95th percentile	Mean (SD)	Median	5th percentile	95th percentile		Percentage change, %^b^
Energy (kJ/day)	8675 (2238)	8479	5311	12,708	8547 (2204)	8350	5250	12,519	− 128.6	− 1.48
Protein (g/day)	81.3 (23.3)	79.6	46.4	121.3	80.1 (22.9)	78.30	46.07	119.72	− 1.18	− 1.45
Total fat (g/day)	76.5 (27.5)	73.7	36.6	126.0	72.1 (26.3)	69.27	34.27	119.49	− 4.38	− 5.72
SFA (g/day)	29.2 (11.7)	27.8	12.7	50.7	26.7 (11.2)	25.22	11.03	47.19	− 2.57	− 8.80
PUFA (g/day)^c^	14.1 (6.88)	13.1	5.1	26.8	12.7 (5.488)	11.94	5.43	22.80	− 1.39	− 10.37
TFA (g/day)	1.47 (0.794)	1.34	0.43	2.93	1.20 (0.649)	1.08	0.34	2.36	− 0.29	− 19.8
Carbohydrates (g/day)	249 (74.4)	243	139	381	252 (72.7)	246	143.30	380.44	2.45	0.98
Total sugars (g/day)	119 (45.5)	113	54	201	124 (46.3)	119	58.32	207.25	5.44	4.59
Englyst fibre (g/day)	16.3 (6.34)	15.6	7.3	27.5	17.7 (6.40)	17.11	8.44	28.98	1.42	8.70
Alcohol (g/day)	16.1 (21.0)	8.7	0.0	58.5	16.9 (21.7)	9.05	0.00	59.91	0.74	4.62
Calcium (mg/day)	959 (329)	923	492	1545	975 (325)	942	509	1550	16.33	1.70
Iron (mg/day)	13.6 (4.14)	13.2	7.3	20.8	12.3 (3.60)	11.98	6.85	18.55	− 1.29	− 9.55
Magnesium (mg/day)	343 (95.5)	335	203	513	330 (88.4)	323	199	486	− 13.03	− 3.80
Potassium (mg/day)	3695 (1064)	3609	2121	5553	3640 (1007)	3571	2115	5384	− 55.20	− 1.49
Total carotene (μg/day)	3091 (2583)	2522	316	7876	2959 (2791)	2095	334	8063	− 131.8	− 4.26
Folate (μg/day)	301 (107.2)	287	152	496	310 (104)	300	160	493	9.79	3.26
Vitamin B6 (mg/day)	2.16 (0.691)	2.11	1.13	3.36	2.05 (0.664)	1.99	1.09	3.23	− 0.11	− 5.07
Vitamin B12 (μg/day)	6.47 (4.49)	5.39	1.64	14.90	6.11 (3.25)	5.56	2.21	11.66	− 0.36	− 5.61
Vitamin C (mg/day)	150 (102)	131	30	338	127 (76.5)	115	29	266	− 23.35	− 15.53
Vitamin D (μg/day)	2.85 (2.71)	2.02	0.34	8.92	3.60 (2.866)	2.83	0.66	9.45	0.75	26.24
Vitamin E (mg/day)	9.01 (3.98)	8.47	3.60	16.27	10.8 (4.26)	10.30	4.85	18.54	1.80	19.99
Retinol (µg/day)	323 (169)	300	89	641	461 (884)	305	89	880	137.2	42.0

All mean differences were statistically significant from zero when using paired *t* tests or Wilcoxon’s rank sum test (*P* < 0.05)

*PUFA* polyunsaturated fatty acids, *SFA* saturated fatty acids, *TFA* trans fatty acids

^a^Calculated as the difference of the mean (UK Nutrient Databank—McCance and Widdowson)

^b^Calculated as the difference of the mean (UK Nutrient Databank—McCance and Widdowson) divided by McCance and Widdowson and multiplied by 100

^c^For the Nutrient databank this is the sum of n−3 and n−6 PUFAs

A total of 35 new nutrients and exposures of interest were available in the UKNDB, and intakes of these nutrients in this population are displayed in Table 3.

**Table 3:** New nutrients incorporated in the updated version (Nutrient databank + other updates) data source in 207,144 participants from UK Biobank

Nutrient	Updated version: Nutrient databank + other updates			
	Mean (SD)	Median	5th percentile	95th percentile
Energy density (kJ/g per day)*	6.47 (1.67)	6.28	4.10	9.45
Animal protein (g/day)*	52.1 (20.6)	51.00	20.55	87.00
Plant protein (g/day)*	28.0 (9.84)	26.78	14.42	45.56
Animal fat (g/day)*	40.4 (18.7)	37.89	14.63	74.62
Plant fat (g/day)*	31.7 (15.3)	29.45	11.30	59.78
MUFA (g/day)	26.1 (10.2)	24.92	11.74	44.55
n−3 PUFA (g/day)	1.97 (0.966)	1.79	0.77	3.75
n−6 PUFA (g/day)	10.80 (4.86)	10.02	4.41	19.70
Free sugar (% daily energy intake)	11.8 (5.8)	11	3.7	22.1
Free sugars (g/day)*	60.0 (34.7)	54.31	15.09	123.73
Non-free sugars (g/day)*	63.9 (30.3)	59.99	22.70	118.40
Non-milk extrinsic sugars (g/day)	64 (35)	59	18	128
Intrinsic and milk sugars (g/day)	60 (27)	57	22	108
Fructose (g/day)	28 (14)	26	8.33	53
Glucose (g/day)	26 (13)	25	8.96	49
Sucrose (g/day)	47 (24)	43	16	91
Lactose (g/day)	14 (8)	13	2.68	27
Maltose (g/day)	6.67 (6.85)	4.69	1.15	20.17
Other sugars (g/day)	2.30 (2.89)	1.63	0.04	6.32
Alpha-carotene (µg/day)	516 (644)	266	3.60	1651
Beta-carotene (µg/day)	2615 (2415)	1887	303	7024
Beta cryptoxanthin (µg/day)	172 (378)	103	6.6	386
Vitamin A (retinol equivalents) (µg/day)	954 (999)	729	241	2243
Biotin (µg/day)	43 (16)	40	22	71
Chloride (mg/day)	3351 (1135)	3201	1779	5418
Copper (mg/day)	1.37 (0.49)	1.31	0.75	2.22
Iron, haem (mg/day)	0.60 (0.49)	0.50	0	1.44
Iron, non-haem (mg/day)	12 (3.5)	11	6.4	18
Iodine (µg/day)	209 (100)	190	91	392
Manganese (mg/day)	4.20 (1.46)	4.07	2.07	6.79
Sodium (mg/day)	1937 (735)	1831	946	3288
Niacin equivalent (mg/day)	38 (11)	37	21	57
Pantothenic acid (mg/day)	461 (884)	305	89	880
Selenium (µg/day)	52 (24)	48	23	95
Total nitrogen (g/day)	12 (4)	12	7.3	19
Zinc (mg/day)	9.65 (3.12)	9.32	5.24	15.1

*PUFA* polyunsaturated fatty acids, *MUFA* monounsaturated fatty acids

*Nutrients not available in the Nutrient databank food composition tables (please see details in Supplementary methods)

Table 4 shows the correlations and the strengths of agreement on ranking nutrient intakes between the previous and the updated version. Except for TFA (*r* = 0.58) and some of the fat-soluble vitamins, high correlations (*r* > 0.90) were found between nutrients calculated using the two versions: energy (*r* = 0.96), protein (*r* = 0.97), total fat (*r* = 0.95), carbohydrates (*r* = 0.95), saturated fat (*r* = 0.91), total sugars (*r* = 0.96), and fibre (*r* = 0.94), with the strongest correlation being for alcohol intake (*r* = 0.99). The percentage of agreement between the two versions was generally good, with the majority of the nutrients classified into the same or adjacent fifth ranging from 90.7% for retinol (*κ* = 0.64) to 99.3% for protein (*κ* = 0.88); however, the percentage agreement was lower for TFA (76.3%, *κ* = 0.42), and slightly lower for vitamin E (88.1%, *κ* = 0.60) and vitamin B6 (89.7%, *κ* = 0.63). The full list of nutrients and the categorization of participants into fifths based on the previous and the updated version is shown in Tables 5 and 6, while the range of intakes within each fifth is reported in Supplementary Table 4. Finally, each food item in the updated version of the nutrient calculation was assigned to a food group, which is showed in Supplementary Table 5 and explained in detail elsewhere [25]

**Table 4:** Comparison of total energy and nutrient intake between previous (McCance and Widdowson) and updated (Nutrient databank + other updates) in 207,144 participants from UK Biobank

Nutrient	Spearman's *r*	Percentage in the same fifth	Percentage in the same or adjacent fifth	Weighted *k*
Energy	0.962	78.7	98.9	0.86
Protein	0.973	81.4	99.3	0.88
Total fat	0.952	71.1	98.8	0.81
SFA	0.908	62.3	96.5	0.74
PUFA^a^	0.887	58.2	94.6	0.71
TFA	0.583	37.6	76.3	0.42
Carbohydrates	0.952	77.1	98.5	0.84
Total sugars	0.959	77.8	98.6	0.85
Englyst fibre	0.935	67.8	97.6	0.79
Alcohol	0.990	93.0	100.0	0.96
Calcium	0.935	72.4	97.6	0.81
Iron	0.939	67.5	98.2	0.79
Magnesium	0.957	76.4	98.7	0.84
Potassium	0.945	76.1	98.1	0.84
Total carotene	0.894	61.4	95.2	0.73
Folate	0.914	64.0	96.5	0.76
Vitamin B6	0.813	50.7	89.7	0.63
Vitamin B12	0.911	65.4	96.2	0.76
Vitamin C	0.955	73.6	98.8	0.83
Vitamin D	0.856	58.2	92.6	0.69
Vitamin E	0.790	48.9	88.1	0.60
Retinol	0.797	52.5	90.7	0.64

*PUFA* polyunsaturated fatty acids, *SFA* saturated fatty acids, *TFA* trans fatty acids

^a^For the Nutrient databank this is the sum of n−3 and n−6 PUFAs

**Table 5:**
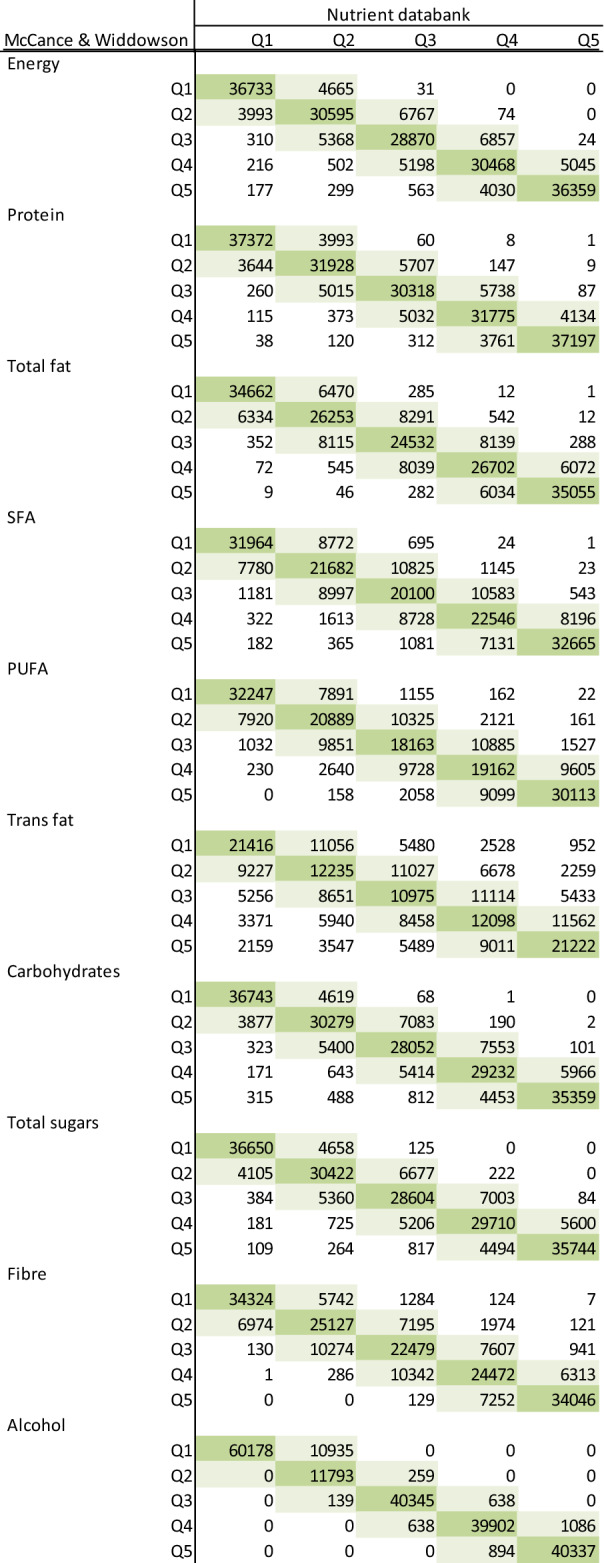
Dietary intakes of energy, macronutrients and fibre by fifths, shaded cells depict participants categorised into the same (dark shading) or adjacent (light shading) quintile using the previous (McCance and Widdowson) and the updated (Nutrient databank + other updates)

For the Nutrient databank total PUFA was determined as the sum of n−3 and n−6 PUFAs

**Table 6:**
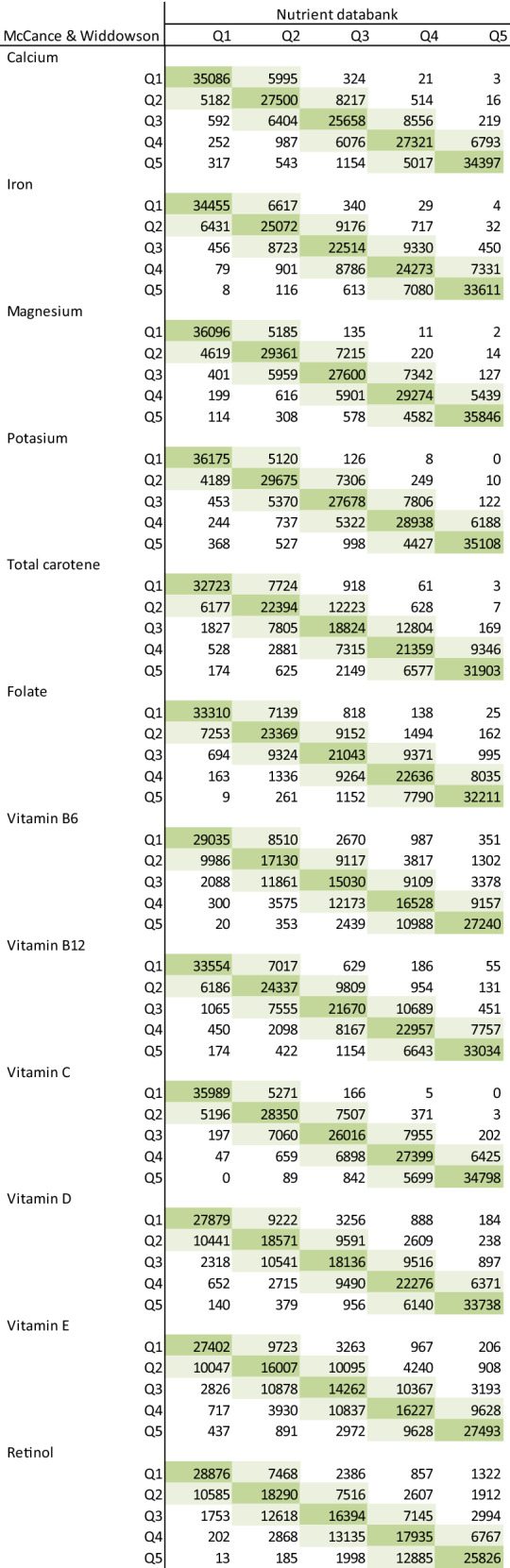
Dietary intakes of micronutrients by fifths, shaded cells depict participants categorised into the same (dark shading) or adjacent (light shading) quintile using the previous (McCance and Widdowson) and the updated (Nutrient databank + other updates)

## Discussion

We have described the updated version of the Oxford WebQ 24-h dietary assessment and compared it with the previous version of this questionnaire among participants in UK Biobank. In general, small absolute mean differences in nutrient intakes between the two versions were observed, and the ranking of individuals was minimally affected for most nutrients. The only substantial differences were observed for TFA and vitamin C, for which intakes in the updated version were lower and for retinol, vitamin D and E, for which intakes were higher. We have incorporated new dietary variables, which will allow researchers to assess whether they are related to non-communicable diseases. Also, with this update, we have made it easier for future users to continue this updating process using future releases of the UKNDB.

After categorising the nutrient intakes, there was very high agreement between the two versions for total energy intake and macronutrients. The closest agreement was observed for alcohol intake, for which 100% of the participants were in the same or adjacent fifth, followed by total protein. Although the absolute intakes of carbohydrates and total sugars did not differ much between the two versions, we did observe that a small number of participants who were in the highest quintile of consumption in the previous version are now in the lowest quintile. This may be due to a concentrated fruit juice code not being sufficiently diluted with water in the previous version of the nutrient calculation. As expected, intakes of TFA were lower in the updated version of the nutrient calculation and there was moderate agreement with the previous version. Most TFA in the diet are produced when converting vegetable oils into semi-solid fats during the process of partial hydrogenation. TFA are well-established risk factors for cardiovascular disease [26], and the food industry has voluntarily reduced or eliminated some artificial TFA in processed foods in the UK in the last 15 years [27]. The previous version used FCT in which nutrient content was published from foods chemically analysed up to 2002 (including analytic data pre-dating the publication date), and, therefore, the ‘true’ TFA intake in 2009–2012, when the participants completed the Oxford WebQ, was likely lower [28]. This previous version also had substantial missing data for TFA, and for this reason this nutrient was not released in UK Biobank. The lower mean TFA intake in the updated version is likely an underestimated difference due to previous missing data on TFA, and also due to food reformulation over time and/or the different imputations of TFA between the two FCT versions of the nutrient calculation. The main sources of TFA in the previous version were likely to be fat spreads and desserts and biscuits, while in the updated version they are likely to be mainly naturally occurring TFA in food produced from ruminant animals. Intakes of TFA are below the dietary reference value of < 2% of total energy, and values are consistent with those reported by the UK NDNS [29].

Intakes of SFAs were also lower in the updated version of the nutrient calculation, but with high agreement in ranking between the two versions. One of the major contributors to SFA in this cohort is dairy fat spread, and, therefore, it is possible that the decrease in SFA may be due to the decrease of 20–60% in the portion sizes allocated for some spreads in the revised version (e.g. spreads on crispbreads, slices of bread, bread rolls, and oatcakes, see supplement for more details).

There were also differences in vitamin intakes between the two versions. Vitamin C intake was on average 17% lower in the updated version compared to the previous version. When vitamin C intake was divided into fifths, the majority of the participants remained classified in the same or an adjacent category. The decrease in vitamin C may be due to fruit juice, which is the largest source of vitamin C in this cohort and in which the previous version of the questionnaire had a concentrated fruit juice code not sufficiently diluted with water. On the other hand, we observed an increase in the intake estimates of retinol and vitamins D and E, although there was substantial agreement between the two versions when these nutrients were categorised. This may be due to the incorporation of fats used when cooking in this updated version, which were mainly vegetable oils; increases in vitamin D may also have occurred due to increases in food fortification, although no fortified foods were preferred when allocating food codes to the WebQ items. Moreover, differences in micronutrient content between the different FCTs are to be expected even if these FCT were created from similar sources; this may be due to for example food reformulation, re-analysis of foods resulting in differences due to storage conditions, fortification or season when the food was sampled. Lastly, imputation of missing values in the UKNDB may have contributed to changes in the nutrient intakes observed [30].

Among the new dietary variables that have been incorporated in this updated version, are MUFAs, n-3 and n-6 PUFAs. The UKNDB does not have information on total essential PUFAs, but n-3 and n-6 fatty acids account for the vast majority of PUFAs in the diet; therefore, researchers using this resource could sum these two fatty acids as a proxy of total PUFA. Other dietary variables that have been incorporated are animal and plant fat and protein, and free sugars. The mean intake of free sugars in this population is slightly above the recommended value of < 10% of total energy intake by the World Health Organization [31].

This study has some strengths and limitations. The updated FCT has over three times more food codes than the previous one, which allowed for a better matching between reported food intakes and nutrient composition. This updated version of the nutrient calculation was developed to improve accuracy and in very few cases also validity (where the original food code did not accurately match the food description in the WebQ) of the dietary intakes of the participants when they completed the questionnaire, and so it is expected to decrease measurement error. Non-differential misclassification of dietary intakes may attenuate the relationship in diet–disease associations in prospective studies [32]. However, it should be emphasized that, as in all questionnaire-based assessments of dietary intake, there will be some measurement error, especially systematic bias due to underreporting [20].

In conclusion, we have described an updated version of the nutrient calculation of the Oxford WebQ 24-h dietary assessment and compared it with the previous version. Small absolute group differences in nutrient intakes between the two versions were observed and the ranking of individuals was minimally affected for most nutrients. The greatest differences were observed for TFA and vitamin C, for which intakes in the updated version were lower; and for retinol, vitamin D and E, for which the reported intakes were higher. This updated version of the nutrient calculation was developed to improve accuracy and personalisation of the dietary intakes of the participants and, therefore, some reduction in non-differential misclassification in diet–disease associations is expected. This new version of the nutrient calculation and new dietary variables will be returned to UK Biobank, together with a food grouping system developed using this updated version of the nutrient calculation [25].

## Supplementary Information

Below is the link to the electronic supplementary material.

Supplementary file1 (PDF 368 KB)

Supplementary file2 (PDF 272 KB)

Supplementary file3 (PDF 132 KB)

Supplementary file4 (DOCX 20 KB)

Supplementary file5 (PDF 838 KB)

## Data Availability

UK Biobank is an open access resource. Bona fide researchers can apply to use the UK Biobank data set by registering and applying at http://www.ukbiobank.ac.uk/register-apply.
