# Supplementary material for: Description of the updated nutrition calculation of the Oxford WebQ questionnaire and comparison with the previous version among 207,144 participants in UK Biobank
Source: Eur J Nutr. 2021 May 6;60(7):4019–30. doi: 10.1007/s00394-021-02558-4 (PMC8437868; doi:10.1007/s00394-021-02558-4)
Supplement: Supplementary file 2 — Supplementary file2 (PDF 272 KB) [file 394_2021_2558_MOESM2_ESM.pdf]

Supplementary table 2. Nutrient calculation in the updated version (Nutrient databank).

	Percentage of the total					Food codes from UK Nutrient Databank and the % used from each food code																					
Food item	Free sugars	Plant prot	Animal prot	Plant fat	Animal fat	Code1	%	Code2	%	Code3	%	Code4	%	Code5	%	Code6	%	Code7	%	Code8	%	code9	%	code10	%	code11	%
add_salt	0%	100%	0%	100%	0%	2522	100																				
alcohol_beercider	100%	100%	0%	100%	0%	2374	35	2362	35	2379	6	8350	6	8349	6	2380	6	2380	6								
alcohol_other	97%	100%	0%	1%	99%	2397	33.4	2399	33.3	2400	33.3																
alcohol_spirits	0%	100%	0%	100%	0%	2402	100																				
alcohol_wine_fort	100%	100%	0%	100%	0%	2391	16.7	2392	16.7	2393	16.6	2390	50														
alcohol_wine_red_large	100%	100%	0%	100%	0%	8352	100																				
alcohol_wine_red_med	100%	100%	0%	100%	0%	8352	100																				
alcohol_wine_red_small	100%	100%	0%	100%	0%	8352	100																				
alcohol_wine_rose_large	100%	100%	0%	100%	0%	8353	100																				
alcohol_wine_rose_med	100%	100%	0%	100%	0%	8353	100																				
alcohol_wine_rose_small	100%	100%	0%	100%	0%	8353	100																				
alcohol_wine_white_large	100%	100%	0%	100%	0%	8354	25	8355	25	8357	25	8356	25														
alcohol_wine_white_med	100%	100%	0%	100%	0%	8354	25	8355	25	8357	25	8356	25														
alcohol_wine_white_small	100%	100%	0%	100%	0%	8354	25	8355	25	8357	25	8356	25														
biscuit_choc	96%	30%	70%	50%	50%	7662	25	260	50	10516	25																
biscuit_choc_gf	96%	31%	69%	50%	50%	7662	25	260	50	10516	25																
biscuit_choccov	98%	30%	70%	50%	50%	8193	34	253	33	8194	33																
biscuit_choccov_gf	98%	31%	69%	50%	50%	8193	34	253	33	8194	33																
biscuit_sweet	92%	60%	40%	60%	40%	259	33.4	263	33.3	8162	33.3																
biscuit_sweet_gf	100%	61%	39%	60%	40%	8872	100																				
bread_baguette_gf_nonwhite	1%	99%	1%	100%	0%	10459	50	8394	50																		
bread_baguette_gf_unanswered	1%	90%	10%	100%	0%	8864	33.4	10459	33.3	8395	33.3																
bread_baguette_gf_white	1%	90%	10%	100%	0%	8864	100																				
bread_baguette_mixed	0%	100%	0%	100%	0%	112	33.4	102	33.3	7609	16.7	10775	16.6														
bread_baguette_other	0%	100%	0%	100%	0%	110	20	114	20	120	60																
bread_baguette_seeded	0%	100%	0%	100%	0%	2168	33.4	2167	33.3	8148	33.3																
bread_baguette_spread_butter_dunno_med	0%	20%	80%	20%	80%	9407	25	10039	25	851	25	852	25														
bread_baguette_spread_butter_dunno_thick	0%	20%	80%	20%	80%	9407	25	10039	25	851	25	852	25														
bread_baguette_spread_butter_dunno_thin	0%	20%	80%	20%	80%	9407	25	10039	25	851	25	852	25														
bread_baguette_spread_butter_fat_med	0%	0%	100%	0%	100%	851	50	852	50																		
bread_baguette_spread_butter_fat_thick	0%	0%	100%	0%	100%	851	50	852	50																		
bread_baguette_spread_butter_fat_thin	0%	0%	100%	0%	100%	851	50	852	50																		
bread_baguette_spread_butter_lowfat_med	0%	45%	55%	45%	55%	10140	100																				
bread_baguette_spread_butter_lowfat_thick	0%	45%	55%	45%	55%	10140	100																				
bread_baguette_spread_butter_lowfat_thin	0%	45%	55%	45%	55%	10140	100																				
bread_baguette_spread_butter_spread_fat_med	0%	40%	60%	40%	60%	9407	50	10039	50																		
bread_baguette_spread_butter_spread_fat_thick	0%	40%	60%	40%	60%	9407	50	10039	50																		
bread_baguette_spread_butter_spread_fat_thin	0%	40%	60%	40%	60%	9407	50	10039	50																		
bread_baguette_spread_butter_spread_lowfat_med	0%	45%	55%	45%	55%	3891	50	10894	50																		
bread_baguette_spread_butter_spread_lowfat_thick	0%	45%	55%	45%	55%	3891	50	10894	50																		
bread_baguette_spread_butter_spread_lowfat_thin	0%	45%	55%	45%	55%	3891	50	10894	50																		
bread_baguette_spread_dairy_chol_med	0%	97%	3%	97%	3%	3848	100																				
bread_baguette_spread_dairy_chol_thick	0%	97%	3%	97%	3%	3848	100																				
bread_baguette_spread_dairy_chol_thin	0%	97%	3%	97%	3%	3848	100																				
bread_baguette_spread_dairy_dunno_med	0%	97%	3%	97%	3%	10047	50	7775	50																		
bread_baguette_spread_dairy_dunno_thick	0%	97%	3%	97%	3%	10047	50	7775	50																		
bread_baguette_spread_dairy_dunno_thin	0%	97%	3%	97%	3%	10047	50	7775	50																		
bread_baguette_spread_dairy_fat_med	0%	97%	3%	97%	3%	7775	100																				
bread_baguette_spread_dairy_fat_thick	0%	97%	3%	97%	3%	7775	100																				
bread_baguette_spread_dairy_fat_thin	0%	97%	3%	97%	3%	7775	100																				
bread_baguette_spread_dairy_lowfat_med	0%	99%	1%	99%	1%	10047	100																				
bread_baguette_spread_dairy_lowfat_thick	0%	99%	1%	99%	1%	10047	100																				
bread_baguette_spread_dairy_lowfat_thin	0%	99%	1%	99%	1%	10047	100																				
bread_baguette_spread_dairy_vlowfat_med	0%	99%	1%	99%	1%	10047	100																				
bread_baguette_spread_dairy_vlowfat_thick	0%	99%	1%	99%	1%	10047	100																				
bread_baguette_spread_dairy_vlowfat_thin	0%	99%	1%	99%	1%	10047	100																				
bread_baguette_spread_dunno_chol_med	0%	97%	3%	97%	3%	3848	33.4	3243	33.3	2849	33.3																
bread_baguette_spread_dunno_chol_thick	0%	97%	3%	97%	3%	3848	33.4	3243	33.3	2849	33.3																
bread_baguette_spread_dunno_chol_thin	0%	97%	3%	97%	3%	3848	33.4	3243	33.3	2849	33.3																
bread_baguette_spread_dunno_dunno_med	0%	97%	3%	97%	3%	10047	25	10049	25	10043	25	7775	25														
bread_baguette_spread_dunno_dunno_thick	0%	97%	3%	97%	3%	10047	25	10049	25	10043	25	7775	25														
bread_baguette_spread_dunno_dunno_thin	0%	97%	3%	97%	3%	10047	25	10049	25	10043	25	7775	25														
bread_baguette_spread_dunno_fat_med	0%	97%	3%	97%	3%	10043	50	7775	50																		
bread_baguette_spread_dunno_fat_thick	0%	97%	3%	97%	3%	10043	50	7775	50																		
bread_baguette_spread_dunno_fat_thin	0%	97%	3%	97%	3%	10043	50	7775	50																		
bread_baguette_spread_dunno_lowfat_med	0%	97%	3%	97%	3%	10047	50	10049	50																		
bread_baguette_spread_dunno_lowfat_thick	0%	97%	3%	97%	3%	10047	50	10049	50																		
bread_baguette_spread_dunno_lowfat_thin	0%	97%	3%	97%	3%	10047	50	10049	50																		
bread_baguette_spread_dunno_vlowfat_med	0%	97%	3%	97%	3%	10047	50	10049	50																		
bread_baguette_spread_dunno_vlowfat_thick	0%	97%	3%	97%	3%	10047	50	10049	50																		
bread_baguette_spread_dunno_vlowfat_thin	0%	97%	3%	97%	3%	10047	50	10049	50																		
bread_baguette_spread_hardmarg_med	0%	100%	0%	100%	0%	860	100																				
bread_baguette_spread_hardmarg_thick	0%	100%	0%	100%	0%	860	100																				
bread_baguette_spread_hardmarg_thin	0%	100%	0%	100%	0%	860	100																				
bread_baguette_spread_olive_chol_med	0%	99%	1%	99%	1%	3364	50	10053	50																		
bread_baguette_spread_olive_chol_thick	0%	99%	1%	99%	1%	3364	50	10053	50																		
bread_baguette_spread_olive_chol_thin	0%	99%	1%	99%	1%	3364	50	10053	50																		
bread_baguette_spread_olive_dunno_med	0%	99%	1%	99%	1%	10048	50	10042	25	10131	25																
bread_baguette_spread_olive_dunno_thick	0%	99%	1%	99%	1%	10048	50	10042	25	10131	25																
bread_baguette_spread_olive_dunno_thin	0%	99%	1%	99%	1%	10048	50	10042	25	10131	25																
bread_baguette_spread_olive_fat_med	0%	99%	1%	99%	1%	10042	75	10131	25																		
bread_baguette_spread_olive_fat_thick	0%	99%	1%	99%	1%	10042	75	10131	25																		
bread_baguette_spread_olive_fat_thin	0%	99%	1%	99%	1%	10042	75	10131	25																		
bread_baguette_spread_olive_lowfat_med	0%	100%	1%	100%	0%	10048	100																				
bread_baguette_spread_olive_lowfat_thick	0%	100%	1%	100%	0%	10048	100																				
bread_baguette_spread_olive_lowfat_thin	0%	100%	1%	100%	0%	10048	100																				
bread_baguette_spread_olive_vlowfat_med	0%	100%	1%	100%	0%	10048	100																				
bread_baguette_spread_olive_vlowfat_thick	0%	100%	1%	100%	0%	10048	100																				
bread_baguette_spread_olive_vlowfat_thin	0%	100%	1%	100%	0%	10048	100																				
bread_baguette_spread_other_med	0%	60%	40%	60%	40%	856	18	851	7.5	852	7.5	3848	33.5	2849	33.5												
bread_baguette_spread_other_thick	0%	60%	40%	60%	40%	856	18	851	7.5	852	7.5	3848	33.5	2849	33.5												
bread_baguette_spread_other_thin	0%	60%	40%	60%	40%	856	18	851	7.5	852	7.5	3848	33.5	2849	33.5												
bread_baguette_spread_polymarg_chol_med	0%	100%	0%	100%	0%	2849	33.4	3848	33.3	3243	33.3																
bread_baguette_spread_polymarg_chol_thick	0%	100%	0%	100%	0%	2849	33.4	3848	33.3	3243	33.3																
bread_baguette_spread_polymarg_chol_thin	0%	100%	0%	100%	0%	2849	33.4	3848	33.3	3243	33.3																
bread_baguette_spread_polymarg_dunno_med	0%	100%	0%	100%	0%	10049	50	10043	25	10044	25																
bread_baguette_spread_polymarg_dunno_thick	0%	100%	0%	100%	0%	10049	50	10043	25	10044	25																
bread_baguette_spread_polymarg_dunno_thin	0%	100%	0%	100%	0%	10049	50	10043	25	10044	25																
bread_baguette_spread_polymarg_fat_med	0%	100%	0%	100%	0%	10043	50	10044	50																		
bread_baguette_spread_polymarg_fat_thick	0%	100%	0%	100%	0%	10043	50	10044	50																		
bread_baguette_spread_polymarg_fat_thin	0%	100%	0%	100%	0%	10043	50	10044	50																		
bread_baguette_spread_polymarg_lowfat_med	0%	100%	0%	100%	0%	10049	100																				
bread_baguette_spread_polymarg_lowfat_thick	0%	100%	0%	100%	0%	10049	100																				
bread_baguette_spread_polymarg_lowfat_thin	0%	100%	0%	100%	0%	10049	100																				
bread_baguette_spread_polymarg_vlowfat_med	0%	100%	0%	100%	0%	10049	100																				
bread_baguette_spread_polymarg_vlowfat_thick	0%	100%	0%	100%	0%	10049	100																				
bread_baguette_spread_polymarg_vlowfat_thin	0%	100%	0%	100%	0%	10049	100																				
bread_baguette_spread_soya_chol_med	0%	100%	1%	100%	1%	3848	16.6	3243	16.6	2849	16.8	10786	50														
bread_baguette_spread_soya_chol_thick	0%	100%	1%	100%	1%	3848	16.6	3243	16.6	2849	16.8	10786	50														
bread_baguette_spread_soya_chol_thin	0%	100%	1%	100%	1%	3848	16.6	3243	16.6	2849	16.8	10786	50														
bread_baguette_spread_soya_dunno_med	0%	100%	0%	100%	0%	10980	50	10786	50																		
bread_baguette_spread_soya_dunno_thick	0%	100%	0%	100%	0%	10980	50	10786	50																		
bread_baguette_spread_soya_dunno_thin	0%	100%	0%	100%	0%	10980	50	10786	50																		
bread_baguette_spread_soya_fat_med	0%	100%	0%	100%	0%	10786	100																				
bread_baguette_spread_soya_fat_thick	0%	100%	0%	100%	0%	10786	100																				
bread_baguette_spread_soya_fat_thin	0%	100%	0%	100%	0%	10786	100																				
bread_baguette_spread_soya_lowfat_med	0%	100%	0%	100%	0%	10980	100																				
bread_baguette_spread_soya_lowfat_thick	0%	100%	0%	100%	0%	10980	100																				
bread_baguette_spread_soya_lowfat_thin	0%	100%	0%	100%	0%	10980	100																				
bread_baguette_spread_soya_vlowfat_med	0%	100%	0%	100%	0%	10980	100																				
bread_baguette_spread_soya_vlowfat_thick	0%	100%	0%	100%	0%	10980	100																				
bread_baguette_spread_soya_vlowfat_thin	0%	100%	0%	100%	0%	10980	100																				
bread_baguette_unanswered	0%	100%	0%	100%	0%	120	33.4	133	33.3	102	33.3																
bread_baguette_white	0%	100%	0%	100%	0%	127	50	6977	50																		
bread_baguette_wholemeal	0%	100%	0%	100%	0%	133	100																				
bread_crisp	0%	100%	0%	100%	0%	256	30	255	30	7652	30	3267	10														
bread_crisp_gf	0%	100%	0%	100%	0%	256	30	255	30	7652	30	3267	10														
bread_crisp_spread_butter_dunno_med	0%	20%	80%	20%	80%	9407	25	10039	25	851	25	852	25														
bread_crisp_spread_butter_dunno_thick	0%	20%	80%	20%	80%	9407	25	10039	25	851	25	852	25														
bread_crisp_spread_butter_dunno_thin	0%	20%	80%	20%	80%	9407	25	10039	25	851	25	852	25														
bread_crisp_spread_butter_fat_med	0%	0%	100%	0%	100%	851	50	852	50																		
bread_crisp_spread_butter_fat_thick	0%	0%	100%	0%	100%	851	50	852	50																		
bread_crisp_spread_butter_fat_thin	0%	0%	100%	0%	100%	851	50	852	50																		
bread_crisp_spread_butter_lowfat_med	0%	45%	55%	45%	55%	10140	100																				
bread_crisp_spread_butter_lowfat_thick	0%	45%	55%	45%	55%	10140	100																				
bread_crisp_spread_butter_lowfat_thin	0%	45%	55%	45%	55%	10140	100																				
bread_crisp_spread_butter_spread_fat_med	0%	40%	60%	40%	60%	9407	50	10039	50																		
bread_crisp_spread_butter_spread_fat_thick	0%	40%	60%	40%	60%	9407	50	10039	50																		
bread_crisp_spread_butter_spread_fat_thin	0%	40%	60%	40%	60%	9407	50	10039	50																		
bread_crisp_spread_butter_spread_lowfat_med	0%	45%	55%	45%	55%	3891	50	10894	50																		
bread_crisp_spread_butter_spread_lowfat_thick	0%	45%	55%	45%	55%	3891	50	10894	50																		
bread_crisp_spread_butter_spread_lowfat_thin	0%	45%	55%	45%	55%	3891	50	10894	50																		
bread_crisp_spread_dairy_chol_med	0%	97%	3%	97%	3%	3848	100																				
bread_crisp_spread_dairy_chol_thick	0%	97%	3%	97%	3%	3848	100																				
bread_crisp_spread_dairy_chol_thin	0%	97%	3%	97%	3%	3848	100																				
bread_crisp_spread_dairy_dunno_med	0%	97%	3%	97%	3%	10047	50	7775	50																		
bread_crisp_spread_dairy_dunno_thick	0%	97%	3%	97%	3%	10047	50	7775	50																		
bread_crisp_spread_dairy_dunno_thin	0%	97%	3%	97%	3%	10047	50	7775	50																		
bread_crisp_spread_dairy_fat_med	0%	97%	3%	97%	3%	7775	100																				
bread_crisp_spread_dairy_fat_thick	0%	97%	3%	97%	3%	7775	100																				
bread_crisp_spread_dairy_fat_thin	0%	97%	3%	97%	3%	7775	100																				
bread_crisp_spread_dairy_lowfat_med	0%	99%	1%	99%	1%	10047	100																				
bread_crisp_spread_dairy_lowfat_thick	0%	99%	1%	99%	1%	10047	100																				
bread_crisp_spread_dairy_lowfat_thin	0%	99%	1%	99%	1%	10047	100																				
bread_crisp_spread_dairy_vlowfat_med	0%	99%	1%	99%	1%	10047	100																				
bread_crisp_spread_dairy_vlowfat_thick	0%	99%	1%	99%	1%	10047	100																				
bread_crisp_spread_dairy_vlowfat_thin	0%	99%	1%	99%	1%	10047	100																				
bread_crisp_spread_dunno_chol_med	0%	97%	3%	97%	3%	3848	33.4	3243	33.3	2849	33.3																
bread_crisp_spread_dunno_chol_thick	0%	97%	3%	97%	3%	3848	33.4	3243	33.3	2849	33.3																
bread_crisp_spread_dunno_chol_thin	0%	97%	3%	97%	3%	3848	33.4	3243	33.3	2849	33.3																
bread_crisp_spread_dunno_dunno_med	0%	97%	3%	97%	3%	10047	25	10049	25	10043	25	7775	25														
bread_crisp_spread_dunno_dunno_thick	0%	97%	3%	97%	3%	10047	25	10049	25	10043	25	7775	25														
bread_crisp_spread_dunno_dunno_thin	0%	97%	3%	97%	3%	10047	25	10049	25	10043	25	7775	25														

bread_crisp_spread_dunno_fat_med	0%	97%	3%	97%	3%	10043	50	7775	50						
bread_crisp_spread_dunno_fat_thick	0%	97%	3%	97%	3%	10043	50	7775	50						
bread_crisp_spread_dunno_fat_thin	0%	97%	3%	97%	3%	10043	50	7775	50						
bread_crisp_spread_dunno_lowfat_med	0%	97%	3%	97%	3%	10047	50	10049	50						
bread_crisp_spread_dunno_lowfat_thick	0%	97%	3%	97%	3%	10047	50	10049	50						
bread_crisp_spread_dunno_lowfat_thin	0%	97%	3%	97%	3%	10047	50	10049	50						
bread_crisp_spread_dunno_vlowfat_med	0%	97%	3%	97%	3%	10047	50	10049	50						
bread_crisp_spread_dunno_vlowfat_thick	0%	97%	3%	97%	3%	10047	50	10049	50						
bread_crisp_spread_dunno_vlowfat_thin	0%	97%	3%	97%	3%	10047	50	10049	50						
bread_crisp_spread_hardmarg_med	0%	100%	0%	100%	0%	860	100								
bread_crisp_spread_hardmarg_thick	0%	100%	0%	100%	0%	860	100								
bread_crisp_spread_hardmarg_thin	0%	100%	0%	100%	0%	860	100								
bread_crisp_spread_olive_chol_med	0%	99%	1%	99%	1%	3364	50	10053	50						
bread_crisp_spread_olive_chol_thick	0%	99%	1%	99%	1%	3364	50	10053	50						
bread_crisp_spread_olive_chol_thin	0%	99%	1%	99%	1%	3364	50	10053	50						
bread_crisp_spread_olive_dunno_med	0%	99%	1%	99%	1%	10048	50	10042	25	10131	25				
bread_crisp_spread_olive_dunno_thick	0%	99%	1%	99%	1%	10048	50	10042	25	10131	25				
bread_crisp_spread_olive_dunno_thin	0%	99%	1%	99%	1%	10048	50	10042	25	10131	25				
bread_crisp_spread_olive_fat_med	0%	99%	1%	99%	1%	10042	75	10131	25						
bread_crisp_spread_olive_fat_thick	0%	99%	1%	99%	1%	10042	75	10131	25						
bread_crisp_spread_olive_fat_thin	0%	99%	1%	99%	1%	10042	75	10131	25						
bread_crisp_spread_olive_lowfat_med	0%	100%	1%	100%	0%	10048	100								
bread_crisp_spread_olive_lowfat_thick	0%	100%	1%	100%	0%	10048	100								
bread_crisp_spread_olive_lowfat_thin	0%	100%	1%	100%	0%	10048	100								
bread_crisp_spread_olive_vlowfat_med	0%	100%	1%	100%	0%	10048	100								
bread_crisp_spread_olive_vlowfat_thick	0%	100%	1%	100%	0%	10048	100								
bread_crisp_spread_olive_vlowfat_thin	0%	100%	1%	100%	0%	10048	100								
bread_crisp_spread_other_med	0%	60%	40%	60%	40%	856	18	851	7.5	852	7.5	3848	33.5	2849	33.5
bread_crisp_spread_other_thick	0%	60%	40%	60%	40%	856	18	851	7.5	852	7.5	3848	33.5	2849	33.5
bread_crisp_spread_other_thin	0%	60%	40%	60%	40%	856	18	851	7.5	852	7.5	3848	33.5	2849	33.5
bread_crisp_spread_polymarg_chol_med	0%	100%	0%	100%	0%	2849	33.4	3848	33.3	3243	33.3				
bread_crisp_spread_polymarg_chol_thick	0%	100%	0%	100%	0%	2849	33.4	3848	33.3	3243	33.3				
bread_crisp_spread_polymarg_chol_thin	0%	100%	0%	100%	0%	2849	33.4	3848	33.3	3243	33.3				
bread_crisp_spread_polymarg_dunno_med	0%	100%	0%	100%	0%	10049	50	10043	25	10044	25				
bread_crisp_spread_polymarg_dunno_thick	0%	100%	0%	100%	0%	10049	50	10043	25	10044	25				
bread_crisp_spread_polymarg_dunno_thin	0%	100%	0%	100%	0%	10049	50	10043	25	10044	25				
bread_crisp_spread_polymarg_fat_med	0%	100%	0%	100%	0%	10043	50	10044	50						
bread_crisp_spread_polymarg_fat_thick	0%	100%	0%	100%	0%	10043	50	10044	50						
bread_crisp_spread_polymarg_fat_thin	0%	100%	0%	100%	0%	10043	50	10044	50						
bread_crisp_spread_polymarg_lowfat_med	0%	100%	0%	100%	0%	10049	100								
bread_crisp_spread_polymarg_lowfat_thick	0%	100%	0%	100%	0%	10049	100								
bread_crisp_spread_polymarg_lowfat_thin	0%	100%	0%	100%	0%	10049	100								
bread_crisp_spread_polymarg_vlowfat_med	0%	100%	0%	100%	0%	10049	100								
bread_crisp_spread_polymarg_vlowfat_thick	0%	100%	0%	100%	0%	10049	100								
bread_crisp_spread_polymarg_vlowfat_thin	0%	100%	0%	100%	0%	10049	100								
bread_crisp_spread_soya_chol_med	0%	100%	1%	100%	1%	3848	16.6	3243	16.6	2849	16.8	10786	50		
bread_crisp_spread_soya_chol_thick	0%	100%	1%	100%	1%	3848	16.6	3243	16.6	2849	16.8	10786	50		
bread_crisp_spread_soya_chol_thin	0%	100%	1%	100%	1%	3848	16.6	3243	16.6	2849	16.8	10786	50		
bread_crisp_spread_soya_dunno_med	0%	100%	0%	100%	0%	10980	50	10786	50						
bread_crisp_spread_soya_dunno_thick	0%	100%	0%	100%	0%	10980	50	10786	50						
bread_crisp_spread_soya_dunno_thin	0%	100%	0%	100%	0%	10980	50	10786	50						
bread_crisp_spread_soya_fat_med	0%	100%	0%	100%	0%	10786	100								
bread_crisp_spread_soya_fat_thick	0%	100%	0%	100%	0%	10786	100								
bread_crisp_spread_soya_fat_thin	0%	100%	0%	100%	0%	10786	100								
bread_crisp_spread_soya_lowfat_med	0%	100%	0%	100%	0%	10980	100								
bread_crisp_spread_soya_lowfat_thick	0%	100%	0%	100%	0%	10980	100								
bread_crisp_spread_soya_lowfat_thin	0%	100%	0%	100%	0%	10980	100								
bread_crisp_spread_soya_vlowfat_med	0%	100%	0%	100%	0%	10980	100								
bread_crisp_spread_soya_vlowfat_thick	0%	100%	0%	100%	0%	10980	100								
bread_crisp_spread_soya_vlowfat_thin	0%	100%	0%	100%	0%	10980	100								
bread_garlic	0%	99%	1%	99%	1%	7615	100								
bread_large_bap_gf_nonwhite	1%	99%	1%	100%	0%	10459	50	8394	50						
bread_large_bap_gf_unanswered	1%	90%	10%	100%	0%	8864	33.4	10459	33.3	8395	33.3				
bread_large_bap_gf_white	1%	90%	10%	100%	0%	8864	100								
bread_large_bap_mixed	0%	100%	0%	100%	0%	7620	33.4	7621	33.3	10779	33.3				
bread_large_bap_other	0%	100%	0%	100%	0%	110	20	114	20	120	60				
bread_large_bap_seeded	0%	100%	0%	100%	0%	2168	33.4	2167	33.3	8148	33.3				
bread_large_bap_spread_butter_dunno_med	0%	20%	80%	20%	80%	9407	25	10039	25	851	25	852	25		
bread_large_bap_spread_butter_dunno_thick	0%	20%	80%	20%	80%	9407	25	10039	25	851	25	852	25		
bread_large_bap_spread_butter_dunno_thin	0%	20%	80%	20%	80%	9407	25	10039	25	851	25	852	25		
bread_large_bap_spread_butter_fat_med	0%	0%	100%	0%	100%	851	50	852	50						
bread_large_bap_spread_butter_fat_thick	0%	0%	100%	0%	100%	851	50	852	50						
bread_large_bap_spread_butter_fat_thin	0%	0%	100%	0%	100%	851	50	852	50						
bread_large_bap_spread_butter_lowfat_med	0%	45%	55%	45%	55%	10140	100								
bread_large_bap_spread_butter_lowfat_thick	0%	45%	55%	45%	55%	10140	100								
bread_large_bap_spread_butter_lowfat_thin	0%	45%	55%	45%	55%	10140	100								
bread_large_bap_spread_butter_spread_fat_med	0%	40%	60%	40%	60%	9407	50	10039	50						
bread_large_bap_spread_butter_spread_fat_thick	0%	40%	60%	40%	60%	9407	50	10039	50						
bread_large_bap_spread_butter_spread_fat_thin	0%	40%	60%	40%	60%	9407	50	10039	50						
bread_large_bap_spread_butter_spread_lowfat_med	0%	45%	55%	45%	55%	3891	50	10894	50						
bread_large_bap_spread_butter_spread_lowfat_thick	0%	45%	55%	45%	55%	3891	50	10894	50						
bread_large_bap_spread_butter_spread_lowfat_thin	0%	45%	55%	45%	55%	3891	50	10894	50						
bread_large_bap_spread_dairy_chol_med	0%	97%	3%	97%	3%	3848	100								
bread_large_bap_spread_dairy_chol_thick	0%	97%	3%	97%	3%	3848	100								
bread_large_bap_spread_dairy_chol_thin	0%	97%	3%	97%	3%	3848	100								
bread_large_bap_spread_dairy_dunno_med	0%	97%	3%	97%	3%	10047	50	7775	50						
bread_large_bap_spread_dairy_dunno_thick	0%	97%	3%	97%	3%	10047	50	7775	50						
bread_large_bap_spread_dairy_dunno_thin	0%	97%	3%	97%	3%	10047	50	7775	50						
bread_large_bap_spread_dairy_fat_med	0%	97%	3%	97%	3%	7775	100								
bread_large_bap_spread_dairy_fat_thick	0%	97%	3%	97%	3%	7775	100								
bread_large_bap_spread_dairy_fat_thin	0%	97%	3%	97%	3%	7775	100								
bread_large_bap_spread_dairy_lowfat_med	0%	99%	1%	99%	1%	10047	100								
bread_large_bap_spread_dairy_lowfat_thick	0%	99%	1%	99%	1%	10047	100								
bread_large_bap_spread_dairy_lowfat_thin	0%	99%	1%	99%	1%	10047	100								
bread_large_bap_spread_dairy_vlowfat_med	0%	99%	1%	99%	1%	10047	100								
bread_large_bap_spread_dairy_vlowfat_thick	0%	99%	1%	99%	1%	10047	100								
bread_large_bap_spread_dairy_vlowfat_thin	0%	99%	1%	99%	1%	10047	100								
bread_large_bap_spread_dunno_chol_med	0%	97%	3%	97%	3%	3848	33.4	3243	33.3	2849	33.3				
bread_large_bap_spread_dunno_chol_thick	0%	97%	3%	97%	3%	3848	33.4	3243	33.3	2849	33.3				
bread_large_bap_spread_dunno_chol_thin	0%	97%	3%	97%	3%	3848	33.4	3243	33.3	2849	33.3				
bread_large_bap_spread_dunno_dunno_med	0%	97%	3%	97%	3%	10047	25	10049	25	10043	25	7775	25		
bread_large_bap_spread_dunno_dunno_thick	0%	97%	3%	97%	3%	10047	25	10049	25	10043	25	7775	25		
bread_large_bap_spread_dunno_dunno_thin	0%	97%	3%	97%	3%	10047	25	10049	25	10043	25	7775	25		
bread_large_bap_spread_dunno_fat_med	0%	97%	3%	97%	3%	10043	50	7775	50						
bread_large_bap_spread_dunno_fat_thick	0%	97%	3%	97%	3%	10043	50	7775	50						
bread_large_bap_spread_dunno_fat_thin	0%	97%	3%	97%	3%	10043	50	7775	50						
bread_large_bap_spread_dunno_lowfat_med	0%	97%	3%	97%	3%	10047	50	10049	50						
bread_large_bap_spread_dunno_lowfat_thick	0%	97%	3%	97%	3%	10047	50	10049	50						
bread_large_bap_spread_dunno_lowfat_thin	0%	97%	3%	97%	3%	10047	50	10049	50						
bread_large_bap_spread_dunno_vlowfat_med	0%	97%	3%	97%	3%	10047	50	10049	50						
bread_large_bap_spread_dunno_vlowfat_thick	0%	97%	3%	97%	3%	10047	50	10049	50						
bread_large_bap_spread_dunno_vlowfat_thin	0%	97%	3%	97%	3%	10047	50	10049	50						
bread_large_bap_spread_hardmarg_med	0%	100%	0%	100%	0%	860	100								
bread_large_bap_spread_hardmarg_thick	0%	100%	0%	100%	0%	860	100								
bread_large_bap_spread_hardmarg_thin	0%	100%	0%	100%	0%	860	100								
bread_large_bap_spread_olive_chol_med	0%	99%	1%	99%	1%	3364	50	10053	50						
bread_large_bap_spread_olive_chol_thick	0%	99%	1%	99%	1%	3364	50	10053	50						
bread_large_bap_spread_olive_chol_thin	0%	99%	1%	99%	1%	3364	50	10053	50						
bread_large_bap_spread_olive_dunno_med	0%	99%	1%	99%	1%	10048	50	10042	25	10131	25				
bread_large_bap_spread_olive_dunno_thick	0%	99%	1%	99%	1%	10048	50	10042	25	10131	25				
bread_large_bap_spread_olive_dunno_thin	0%	99%	1%	99%	1%	10048	50	10042	25	10131	25				
bread_large_bap_spread_olive_fat_med	0%	99%	1%	99%	1%	10042	75	10131	25						
bread_large_bap_spread_olive_fat_thick	0%	99%	1%	99%	1%	10042	75	10131	25						
bread_large_bap_spread_olive_fat_thin	0%	99%	1%	99%	1%	10042	75	10131	25						
bread_large_bap_spread_olive_lowfat_med	0%	100%	1%	100%	0%	10048	100								
bread_large_bap_spread_olive_lowfat_thick	0%	100%	1%	100%	0%	10048	100								
bread_large_bap_spread_olive_lowfat_thin	0%	100%	1%	100%	0%	10048	100								
bread_large_bap_spread_olive_vlowfat_med	0%	100%	1%	100%	0%	10048	100								
bread_large_bap_spread_olive_vlowfat_thick	0%	100%	1%	100%	0%	10048	100								
bread_large_bap_spread_olive_vlowfat_thin	0%	100%	1%	100%	0%	10048	100								
bread_large_bap_spread_other_med	0%	60%	40%	60%	40%	856	18	851	7.5	852	7.5	3848	33.5	2849	33.5
bread_large_bap_spread_other_thick	0%	60%	40%	60%	40%	856	18	851	7.5	852	7.5	3848	33.5	2849	33.5
bread_large_bap_spread_other_thin	0%	60%	40%	60%	40%	856	18	851	7.5	852	7.5	3848	33.5	2849	33.5
bread_large_bap_spread_polymarg_chol_med	0%	100%	0%	100%	0%	2849	33.4	3848	33.3	3243	33.3				
bread_large_bap_spread_polymarg_chol_thick	0%	100%	0%	100%	0%	2849	33.4	3848	33.3	3243	33.3				
bread_large_bap_spread_polymarg_chol_thin	0%	100%	0%	100%	0%	2849	33.4	3848	33.3	3243	33.3				
bread_large_bap_spread_polymarg_dunno_med	0%	100%	0%	100%	0%	10049	50	10043	25	10044	25				
bread_large_bap_spread_polymarg_dunno_thick	0%	100%	0%	100%	0%	10049	50	10043	25	10044	25				
bread_large_bap_spread_polymarg_dunno_thin	0%	100%	0%	100%	0%	10049	50	10043	25	10044	25				
bread_large_bap_spread_polymarg_fat_med	0%	100%	0%	100%	0%	10043	50	10044	50						
bread_large_bap_spread_polymarg_fat_thick	0%	100%	0%	100%	0%	10043	50	10044	50						
bread_large_bap_spread_polymarg_fat_thin	0%	100%	0%	100%	0%	10043	50	10044	50						
bread_large_bap_spread_polymarg_lowfat_med	0%	100%	0%	100%	0%	10049	100								
bread_large_bap_spread_polymarg_lowfat_thick	0%	100%	0%	100%	0%	10049	100								
bread_large_bap_spread_polymarg_lowfat_thin	0%	100%	0%	100%	0%	10049	100								
bread_large_bap_spread_polymarg_vlowfat_med	0%	100%	0%	100%	0%	10049	100								
bread_large_bap_spread_polymarg_vlowfat_thick	0%	100%	0%	100%	0%	10049	100								
bread_large_bap_spread_polymarg_vlowfat_thin	0%	100%	0%	100%	0%	10049	100								
bread_large_bap_spread_soya_chol_med	0%	100%	1%	100%	1%	3848	16.6	3243	16.6	2849	16.8	10786	50		
bread_large_bap_spread_soya_chol_thick	0%	100%	1%	100%	1%	3848	16.6	3243	16.6	2849	16.8	10786	50		
bread_large_bap_spread_soya_chol_thin	0%	100%	1%	100%	1%	3848	16.6	3243	16.6	2849	16.8	10786	50		
bread_large_bap_spread_soya_dunno_med	0%	100%	0%	100%	0%	10980	50	10786	50						
bread_large_bap_spread_soya_dunno_thick	0%	100%	0%	100%	0%	10980	50	10786	50						
bread_large_bap_spread_soya_dunno_thin	0%	100%	0%	100%	0%	10980	50	10786	50						
bread_large_bap_spread_soya_fat_med	0%	100%	0%	100%	0%	10786	100								
bread_large_bap_spread_soya_fat_thick	0%	100%	0%	100%	0%	10786	100								
bread_large_bap_spread_soya_fat_thin	0%	100%	0%	100%	0%	10786	100								
bread_large_bap_spread_soya_lowfat_med	0%	100%	0%	100%	0%	10980	100								
bread_large_bap_spread_soya_lowfat_thick	0%	100%	0%	100%	0%	10980	100								
bread_large_bap_spread_soya_lowfat_thin	0%	100%	0%	100%	0%	10980	100								
bread_large_bap_spread_soya_vlowfat_med	0%	100%	0%	100%	0%	10980	100								
bread_large_bap_spread_soya_vlowfat_thick	0%	100%	0%	100%	0%	10980	100								
bread_large_bap_spread_soya_vlowfat_thin	0%	100%	0%	100%	0%	10980	100								
bread_large_bap_unanswered	0%	100%	0%	100%	0%	159	33.4	7621	33.3	161	33.3				
bread_large_bap_white	0%	100%	0%	100%	0%	116	50	159	50						
bread_large_bap_wholemeal	0%	100%	0%	100%	0%	161	50	117	50						
bread_naan	0%	99%	1%	99%	1%	7622	100								
bread_other	0%	100%	0%	100%	0%	6838	40	148	40	7649	10	384	10		
bread_other_gf	1%	90%	10%	100%	0%	6838	40	148	40	7649	10	384	10		

bread_other_spread_butter_dunno_med	0%	20%	80%	20%	80%	9407	25	10039	25	851	25	852	25		
bread_other_spread_butter_dunno_thick	0%	20%	80%	20%	80%	9407	25	10039	25	851	25	852	25		
bread_other_spread_butter_dunno_thin	0%	20%	80%	20%	80%	9407	25	10039	25	851	25	852	25		
bread_other_spread_butter_fat_med	0%	0%	100%	0%	100%	851	50	852	50						
bread_other_spread_butter_fat_thick	0%	0%	100%	0%	100%	851	50	852	50						
bread_other_spread_butter_fat_thin	0%	0%	100%	0%	100%	851	50	852	50						
bread_other_spread_butter_lowfat_med	0%	45%	55%	45%	55%	10140	100								
bread_other_spread_butter_lowfat_thick	0%	45%	55%	45%	55%	10140	100								
bread_other_spread_butter_lowfat_thin	0%	45%	55%	45%	55%	10140	100								
bread_other_spread_butter_spread_fat_med	0%	40%	60%	40%	60%	9407	50	10039	50						
bread_other_spread_butter_spread_fat_thick	0%	40%	60%	40%	60%	9407	50	10039	50						
bread_other_spread_butter_spread_fat_thin	0%	40%	60%	40%	60%	9407	50	10039	50						
bread_other_spread_butter_spread_lowfat_med	0%	45%	55%	45%	55%	3891	50	10894	50						
bread_other_spread_butter_spread_lowfat_thick	0%	45%	55%	45%	55%	3891	50	10894	50						
bread_other_spread_butter_spread_lowfat_thin	0%	45%	55%	45%	55%	3891	50	10894	50						
bread_other_spread_dairy_chol_med	0%	97%	3%	97%	3%	3848	100								
bread_other_spread_dairy_chol_thick	0%	97%	3%	97%	3%	3848	100								
bread_other_spread_dairy_chol_thin	0%	97%	3%	97%	3%	3848	100								
bread_other_spread_dairy_dunno_med	0%	97%	3%	97%	3%	10047	50	7775	50						
bread_other_spread_dairy_dunno_thick	0%	97%	3%	97%	3%	10047	50	7775	50						
bread_other_spread_dairy_dunno_thin	0%	97%	3%	97%	3%	10047	50	7775	50						
bread_other_spread_dairy_fat_med	0%	97%	3%	97%	3%	7775	100								
bread_other_spread_dairy_fat_thick	0%	97%	3%	97%	3%	7775	100								
bread_other_spread_dairy_fat_thin	0%	97%	3%	97%	3%	7775	100								
bread_other_spread_dairy_lowfat_med	0%	99%	1%	99%	1%	10047	100								
bread_other_spread_dairy_lowfat_thick	0%	99%	1%	99%	1%	10047	100								
bread_other_spread_dairy_lowfat_thin	0%	99%	1%	99%	1%	10047	100								
bread_other_spread_dairy_vlowfat_med	0%	99%	1%	99%	1%	10047	100								
bread_other_spread_dairy_vlowfat_thick	0%	99%	1%	99%	1%	10047	100								
bread_other_spread_dairy_vlowfat_thin	0%	99%	1%	99%	1%	10047	100								
bread_other_spread_dunno_chol_med	0%	97%	3%	97%	3%	3848	33.4	3243	33.3	2849	33.3				
bread_other_spread_dunno_chol_thick	0%	97%	3%	97%	3%	3848	33.4	3243	33.3	2849	33.3				
bread_other_spread_dunno_chol_thin	0%	97%	3%	97%	3%	3848	33.4	3243	33.3	2849	33.3				
bread_other_spread_dunno_dunno_med	0%	97%	3%	97%	3%	10047	25	10049	25	10043	25	7775	25		
bread_other_spread_dunno_dunno_thick	0%	97%	3%	97%	3%	10047	25	10049	25	10043	25	7775	25		
bread_other_spread_dunno_dunno_thin	0%	97%	3%	97%	3%	10047	25	10049	25	10043	25	7775	25		
bread_other_spread_dunno_fat_med	0%	97%	3%	97%	3%	10043	50	7775	50						
bread_other_spread_dunno_fat_thick	0%	97%	3%	97%	3%	10043	50	7775	50						
bread_other_spread_dunno_fat_thin	0%	97%	3%	97%	3%	10043	50	7775	50						
bread_other_spread_dunno_lowfat_med	0%	97%	3%	97%	3%	10047	50	10049	50						
bread_other_spread_dunno_lowfat_thick	0%	97%	3%	97%	3%	10047	50	10049	50						
bread_other_spread_dunno_lowfat_thin	0%	97%	3%	97%	3%	10047	50	10049	50						
bread_other_spread_dunno_vlowfat_med	0%	97%	3%	97%	3%	10047	50	10049	50						
bread_other_spread_dunno_vlowfat_thick	0%	97%	3%	97%	3%	10047	50	10049	50						
bread_other_spread_dunno_vlowfat_thin	0%	97%	3%	97%	3%	10047	50	10049	50						
bread_other_spread_hardmarg_med	0%	100%	0%	100%	0%	860	100								
bread_other_spread_hardmarg_thick	0%	100%	0%	100%	0%	860	100								
bread_other_spread_hardmarg_thin	0%	100%	0%	100%	0%	860	100								
bread_other_spread_olive_chol_med	0%	99%	1%	99%	1%	3364	50	10053	50						
bread_other_spread_olive_chol_thick	0%	99%	1%	99%	1%	3364	50	10053	50						
bread_other_spread_olive_chol_thin	0%	99%	1%	99%	1%	3364	50	10053	50						
bread_other_spread_olive_dunno_med	0%	99%	1%	99%	1%	10048	50	10042	25	10131	25				
bread_other_spread_olive_dunno_thick	0%	99%	1%	99%	1%	10048	50	10042	25	10131	25				
bread_other_spread_olive_dunno_thin	0%	99%	1%	99%	1%	10048	50	10042	25	10131	25				
bread_other_spread_olive_fat_med	0%	99%	1%	99%	1%	10042	75	10131	25						
bread_other_spread_olive_fat_thick	0%	99%	1%	99%	1%	10042	75	10131	25						
bread_other_spread_olive_fat_thin	0%	99%	1%	99%	1%	10042	75	10131	25						
bread_other_spread_olive_lowfat_med	0%	100%	1%	100%	0%	10048	100								
bread_other_spread_olive_lowfat_thick	0%	100%	1%	100%	0%	10048	100								
bread_other_spread_olive_lowfat_thin	0%	100%	1%	100%	0%	10048	100								
bread_other_spread_olive_vlowfat_med	0%	100%	1%	100%	0%	10048	100								
bread_other_spread_olive_vlowfat_thick	0%	100%	1%	100%	0%	10048	100								
bread_other_spread_olive_vlowfat_thin	0%	100%	1%	100%	0%	10048	100								
bread_other_spread_other_med	0%	60%	40%	60%	40%	856	18	851	7.5	852	7.5	3848	33.5	2849	33.5
bread_other_spread_other_thick	0%	60%	40%	60%	40%	856	18	851	7.5	852	7.5	3848	33.5	2849	33.5
bread_other_spread_other_thin	0%	60%	40%	60%	40%	856	18	851	7.5	852	7.5	3848	33.5	2849	33.5
bread_other_spread_polymarg_chol_med	0%	100%	0%	100%	0%	2849	33.4	3848	33.3	3243	33.3				
bread_other_spread_polymarg_chol_thick	0%	100%	0%	100%	0%	2849	33.4	3848	33.3	3243	33.3				
bread_other_spread_polymarg_chol_thin	0%	100%	0%	100%	0%	2849	33.4	3848	33.3	3243	33.3				
bread_other_spread_polymarg_dunno_med	0%	100%	0%	100%	0%	10049	50	10043	25	10044	25				
bread_other_spread_polymarg_dunno_thick	0%	100%	0%	100%	0%	10049	50	10043	25	10044	25				
bread_other_spread_polymarg_dunno_thin	0%	100%	0%	100%	0%	10049	50	10043	25	10044	25				
bread_other_spread_polymarg_fat_med	0%	100%	0%	100%	0%	10043	50	10044	50						
bread_other_spread_polymarg_fat_thick	0%	100%	0%	100%	0%	10043	50	10044	50						
bread_other_spread_polymarg_fat_thin	0%	100%	0%	100%	0%	10043	50	10044	50						
bread_other_spread_polymarg_lowfat_med	0%	100%	0%	100%	0%	10049	100								
bread_other_spread_polymarg_lowfat_thick	0%	100%	0%	100%	0%	10049	100								
bread_other_spread_polymarg_lowfat_thin	0%	100%	0%	100%	0%	10049	100								
bread_other_spread_polymarg_vlowfat_med	0%	100%	0%	100%	0%	10049	100								
bread_other_spread_polymarg_vlowfat_thick	0%	100%	0%	100%	0%	10049	100								
bread_other_spread_polymarg_vlowfat_thin	0%	100%	0%	100%	0%	10049	100								
bread_other_spread_soya_chol_med	0%	100%	1%	100%	1%	3848	16.6	3243	16.6	2849	16.8	10786	50		
bread_other_spread_soya_chol_thick	0%	100%	1%	100%	1%	3848	16.6	3243	16.6	2849	16.8	10786	50		
bread_other_spread_soya_chol_thin	0%	100%	1%	100%	1%	3848	16.6	3243	16.6	2849	16.8	10786	50		
bread_other_spread_soya_dunno_med	0%	100%	0%	100%	0%	10980	50	10786	50						
bread_other_spread_soya_dunno_thick	0%	100%	0%	100%	0%	10980	50	10786	50						
bread_other_spread_soya_dunno_thin	0%	100%	0%	100%	0%	10980	50	10786	50						
bread_other_spread_soya_fat_med	0%	100%	0%	100%	0%	10786	100								
bread_other_spread_soya_fat_thick	0%	100%	0%	100%	0%	10786	100								
bread_other_spread_soya_fat_thin	0%	100%	0%	100%	0%	10786	100								
bread_other_spread_soya_lowfat_med	0%	100%	0%	100%	0%	10980	100								
bread_other_spread_soya_lowfat_thick	0%	100%	0%	100%	0%	10980	100								
bread_other_spread_soya_lowfat_thin	0%	100%	0%	100%	0%	10980	100								
bread_other_spread_soya_vlowfat_med	0%	100%	0%	100%	0%	10980	100								
bread_other_spread_soya_vlowfat_thick	0%	100%	0%	100%	0%	10980	100								
bread_other_spread_soya_vlowfat_thin	0%	100%	0%	100%	0%	10980	100								
bread_roll_gf_nonwhite	1%	99%	1%	100%	0%	10459	50	8394	50						
bread_roll_gf_unanswered	1%	90%	10%	100%	0%	8864	33.4	10459	33.3	8395	33.3				
bread_roll_gf_white	1%	90%	10%	100%	0%	8864	100								
bread_roll_mixed	0%	100%	0%	100%	0%	7620	33.4	7621	33.3	10779	33.3				
bread_roll_other	0%	100%	0%	100%	0%	110	20	114	20	120	60				
bread_roll_seeded	0%	100%	0%	100%	0%	2168	33.4	2167	33.3	8148	33.3				
bread_roll_spread_butter_dunno_med	0%	20%	80%	20%	80%	9407	25	10039	25	851	25	852	25		
bread_roll_spread_butter_dunno_thick	0%	20%	80%	20%	80%	9407	25	10039	25	851	25	852	25		
bread_roll_spread_butter_dunno_thin	0%	20%	80%	20%	80%	9407	25	10039	25	851	25	852	25		
bread_roll_spread_butter_fat_med	0%	0%	100%	0%	100%	851	50	852	50						
bread_roll_spread_butter_fat_thick	0%	0%	100%	0%	100%	851	50	852	50						
bread_roll_spread_butter_fat_thin	0%	0%	100%	0%	100%	851	50	852	50						
bread_roll_spread_butter_lowfat_med	0%	45%	55%	45%	55%	10140	100								
bread_roll_spread_butter_lowfat_thick	0%	45%	55%	45%	55%	10140	100								
bread_roll_spread_butter_lowfat_thin	0%	45%	55%	45%	55%	10140	100								
bread_roll_spread_butter_spread_fat_med	0%	40%	60%	40%	60%	9407	50	10039	50						
bread_roll_spread_butter_spread_fat_thick	0%	40%	60%	40%	60%	9407	50	10039	50						
bread_roll_spread_butter_spread_fat_thin	0%	40%	60%	40%	60%	9407	50	10039	50						
bread_roll_spread_butter_spread_lowfat_med	0%	45%	55%	45%	55%	3891	50	10894	50						
bread_roll_spread_butter_spread_lowfat_thick	0%	45%	55%	45%	55%	3891	50	10894	50						
bread_roll_spread_butter_spread_lowfat_thin	0%	45%	55%	45%	55%	3891	50	10894	50						
bread_roll_spread_dairy_chol_med	0%	97%	3%	97%	3%	3848	100								
bread_roll_spread_dairy_chol_thick	0%	97%	3%	97%	3%	3848	100								
bread_roll_spread_dairy_chol_thin	0%	97%	3%	97%	3%	3848	100								
bread_roll_spread_dairy_dunno_med	0%	97%	3%	97%	3%	10047	50	7775	50						
bread_roll_spread_dairy_dunno_thick	0%	97%	3%	97%	3%	10047	50	7775	50						
bread_roll_spread_dairy_dunno_thin	0%	97%	3%	97%	3%	10047	50	7775	50						
bread_roll_spread_dairy_fat_med	0%	97%	3%	97%	3%	7775	100								
bread_roll_spread_dairy_fat_thick	0%	97%	3%	97%	3%	7775	100								
bread_roll_spread_dairy_fat_thin	0%	97%	3%	97%	3%	7775	100								
bread_roll_spread_dairy_lowfat_med	0%	99%	1%	99%	1%	10047	100								
bread_roll_spread_dairy_lowfat_thick	0%	99%	1%	99%	1%	10047	100								
bread_roll_spread_dairy_lowfat_thin	0%	99%	1%	99%	1%	10047	100								
bread_roll_spread_dairy_vlowfat_med	0%	99%	1%	99%	1%	10047	100								
bread_roll_spread_dairy_vlowfat_thick	0%	99%	1%	99%	1%	10047	100								
bread_roll_spread_dairy_vlowfat_thin	0%	99%	1%	99%	1%	10047	100								
bread_roll_spread_dunno_chol_med	0%	97%	3%	97%	3%	3848	33.4	3243	33.3	2849	33.3				
bread_roll_spread_dunno_chol_thick	0%	97%	3%	97%	3%	3848	33.4	3243	33.3	2849	33.3				
bread_roll_spread_dunno_chol_thin	0%	97%	3%	97%	3%	3848	33.4	3243	33.3	2849	33.3				
bread_roll_spread_dunno_dunno_med	0%	97%	3%	97%	3%	10047	25	10049	25	10043	25	7775	25		
bread_roll_spread_dunno_dunno_thick	0%	97%	3%	97%	3%	10047	25	10049	25	10043	25	7775	25		
bread_roll_spread_dunno_dunno_thin	0%	97%	3%	97%	3%	10047	25	10049	25	10043	25	7775	25		
bread_roll_spread_dunno_fat_med	0%	97%	3%	97%	3%	10043	50	7775	50						
bread_roll_spread_dunno_fat_thick	0%	97%	3%	97%	3%	10043	50	7775	50						
bread_roll_spread_dunno_fat_thin	0%	97%	3%	97%	3%	10043	50	7775	50						
bread_roll_spread_dunno_lowfat_med	0%	97%	3%	97%	3%	10047	50	10049	50						
bread_roll_spread_dunno_lowfat_thick	0%	97%	3%	97%	3%	10047	50	10049	50						
bread_roll_spread_dunno_lowfat_thin	0%	97%	3%	97%	3%	10047	50	10049	50						
bread_roll_spread_dunno_vlowfat_med	0%	97%	3%	97%	3%	10047	50	10049	50						
bread_roll_spread_dunno_vlowfat_thick	0%	97%	3%	97%	3%	10047	50	10049	50						
bread_roll_spread_dunno_vlowfat_thin	0%	97%	3%	97%	3%	10047	50	10049	50						
bread_roll_spread_hardmarg_med	0%	100%	0%	100%	0%	860	100								
bread_roll_spread_hardmarg_thick	0%	100%	0%	100%	0%	860	100								
bread_roll_spread_hardmarg_thin	0%	100%	0%	100%	0%	860	100								
bread_roll_spread_olive_chol_med	0%	99%	1%	99%	1%	3364	50	10053	50						
bread_roll_spread_olive_chol_thick	0%	99%	1%	99%	1%	3364	50	10053	50						
bread_roll_spread_olive_chol_thin	0%	99%	1%	99%	1%	3364	50	10053	50						
bread_roll_spread_olive_dunno_med	0%	99%	1%	99%	1%	10048	50	10042	25	10131	25				
bread_roll_spread_olive_dunno_thick	0%	99%	1%	99%	1%	10048	50	10042	25	10131	25				
bread_roll_spread_olive_dunno_thin	0%	99%	1%	99%	1%	10048	50	10042	25	10131	25				
bread_roll_spread_olive_fat_med	0%	99%	1%	99%	1%	10042	75	10131	25						
bread_roll_spread_olive_fat_thick	0%	99%	1%	99%	1%	10042	75	10131	25						
bread_roll_spread_olive_fat_thin	0%	99%	1%	99%	1%	10042	75	10131	25						
bread_roll_spread_olive_lowfat_med	0%	100%	1%	100%	0%	10048	100								
bread_roll_spread_olive_lowfat_thick	0%	100%	1%	100%	0%	10048	100								
bread_roll_spread_olive_lowfat_thin	0%	100%	1%	100%	0%	10048	100								
bread_roll_spread_olive_vlowfat_med	0%	100%	1%	100%	0%	10048	100								
bread_roll_spread_olive_vlowfat_thick	0%	100%	1%	100%	0%	10048	100								
bread_roll_spread_olive_vlowfat_thin	0%	100%	1%	100%	0%	10048	100								
bread_roll_spread_other_med	0%	60%	40%	60%	40%	856	18	851	7.5	852	7.5	3848	33.5	2849	33.5
bread_roll_spread_other_thick	0%	60%	40%	60%	40%	856	18	851	7.5	852	7.5	3848	33.5	2849	33.5
bread_roll_spread_other_thin	0%	60%	40%	60%	40%	856	18	851	7.5	852	7.5	3848	33.5	2849	33.5
bread_roll_spread_polymarg_chol_med	0%	100%	0%	100%	0%	2849	33.4	3848	33.3	3243	33.3				

bread_roll_spread_polymarg_chol_thick	0%	100%	0%	100%	0%	2849	33.4	3848	33.3	3243	33.3																
bread_roll_spread_polymarg_chol_thin	0%	100%	0%	100%	0%	2849	33.4	3848	33.3	3243	33.3																
bread_roll_spread_polymarg_dunno_med	0%	100%	0%	100%	0%	10049	50	10043	25	10044	25																
bread_roll_spread_polymarg_dunno_thick	0%	100%	0%	100%	0%	10049	50	10043	25	10044	25																
bread_roll_spread_polymarg_dunno_thin	0%	100%	0%	100%	0%	10049	50	10043	25	10044	25																
bread_roll_spread_polymarg_fat_med	0%	100%	0%	100%	0%	10043	50	10044	50																		
bread_roll_spread_polymarg_fat_thick	0%	100%	0%	100%	0%	10043	50	10044	50																		
bread_roll_spread_polymarg_fat_thin	0%	100%	0%	100%	0%	10043	50	10044	50																		
bread_roll_spread_polymarg_lowfat_med	0%	100%	0%	100%	0%	10049	100																				
bread_roll_spread_polymarg_lowfat_thick	0%	100%	0%	100%	0%	10049	100																				
bread_roll_spread_polymarg_lowfat_thin	0%	100%	0%	100%	0%	10049	100																				
bread_roll_spread_polymarg_vlowfat_med	0%	100%	0%	100%	0%	10049	100																				
bread_roll_spread_polymarg_vlowfat_thick	0%	100%	0%	100%	0%	10049	100																				
bread_roll_spread_polymarg_vlowfat_thin	0%	100%	0%	100%	0%	10049	100																				
bread_roll_spread_soya_chol_med	0%	100%	1%	100%	1%	3848	16.6	3243	16.6	2849	16.8	10786	50														
bread_roll_spread_soya_chol_thick	0%	100%	1%	100%	1%	3848	16.6	3243	16.6	2849	16.8	10786	50														
bread_roll_spread_soya_chol_thin	0%	100%	1%	100%	1%	3848	16.6	3243	16.6	2849	16.8	10786	50														
bread_roll_spread_soya_dunno_med	0%	100%	0%	100%	0%	10980	50	10786	50																		
bread_roll_spread_soya_dunno_thick	0%	100%	0%	100%	0%	10980	50	10786	50																		
bread_roll_spread_soya_dunno_thin	0%	100%	0%	100%	0%	10980	50	10786	50																		
bread_roll_spread_soya_fat_med	0%	100%	0%	100%	0%	10786	100																				
bread_roll_spread_soya_fat_thick	0%	100%	0%	100%	0%	10786	100																				
bread_roll_spread_soya_fat_thin	0%	100%	0%	100%	0%	10786	100																				
bread_roll_spread_soya_lowfat_med	0%	100%	0%	100%	0%	10980	100																				
bread_roll_spread_soya_lowfat_thick	0%	100%	0%	100%	0%	10980	100																				
bread_roll_spread_soya_lowfat_thin	0%	100%	0%	100%	0%	10980	100																				
bread_roll_spread_soya_vlowfat_med	0%	100%	0%	100%	0%	10980	100																				
bread_roll_spread_soya_vlowfat_thick	0%	100%	0%	100%	0%	10980	100																				
bread_roll_spread_soya_vlowfat_thin	0%	100%	0%	100%	0%	10980	100																				
bread_roll_unanswered	0%	100%	0%	100%	0%	159	33.4	7621	33.3	161	33.3																
bread_roll_white	0%	100%	0%	100%	0%	158	50	157	50																		
bread_roll_wholemeal	0%	100%	0%	100%	0%	161	100																				
bread_sliced_gf_nonwhite	1%	99%	1%	100%	0%	10459	50	8394	50																		
bread_sliced_gf_unanswered	1%	90%	10%	100%	0%	8864	33.4	10459	33.3	8394	33.3																
bread_sliced_gf_white	1%	90%	10%	100%	0%	8864	100																				
bread_sliced_mixed	0%	100%	0%	100%	0%	112	33.4	102	33.3	7609	16.7	10775	16.6														
bread_sliced_other	0%	100%	0%	100%	0%	110	25	114	25	102	25	120	25														
bread_sliced_seeded	0%	100%	0%	100%	0%	2168	33.4	2167	33.3	8148	33.3																
bread_sliced_spread_butter_dunno_med	0%	20%	80%	20%	80%	9407	25	10039	25	851	25	852	25														
bread_sliced_spread_butter_dunno_thick	0%	20%	80%	20%	80%	9407	25	10039	25	851	25	852	25														
bread_sliced_spread_butter_dunno_thin	0%	20%	80%	20%	80%	9407	25	10039	25	851	25	852	25														
bread_sliced_spread_butter_fat_med	0%	0%	100%	0%	100%	851	50	852	50																		
bread_sliced_spread_butter_fat_thick	0%	0%	100%	0%	100%	851	50	852	50																		
bread_sliced_spread_butter_fat_thin	0%	0%	100%	0%	100%	851	50	852	50																		
bread_sliced_spread_butter_lowfat_med	0%	45%	55%	45%	55%	10140	100																				
bread_sliced_spread_butter_lowfat_thick	0%	45%	55%	45%	55%	10140	100																				
bread_sliced_spread_butter_lowfat_thin	0%	45%	55%	45%	55%	10140	100																				
bread_sliced_spread_butter_spread_fat_med	0%	40%	60%	40%	60%	9407	50	10039	50																		
bread_sliced_spread_butter_spread_fat_thick	0%	40%	60%	40%	60%	9407	50	10039	50																		
bread_sliced_spread_butter_spread_fat_thin	0%	40%	60%	40%	60%	9407	50	10039	50																		
bread_sliced_spread_butter_spread_lowfat_med	0%	45%	55%	45%	55%	3891	50	10894	50																		
bread_sliced_spread_butter_spread_lowfat_thick	0%	45%	55%	45%	55%	3891	50	10894	50																		
bread_sliced_spread_butter_spread_lowfat_thin	0%	45%	55%	45%	55%	3891	50	10894	50																		
bread_sliced_spread_dairy_chol_med	0%	97%	3%	97%	3%	3848	100																				
bread_sliced_spread_dairy_chol_thick	0%	97%	3%	97%	3%	3848	100																				
bread_sliced_spread_dairy_chol_thin	0%	97%	3%	97%	3%	3848	100																				
bread_sliced_spread_dairy_dunno_med	0%	97%	3%	97%	3%	10047	50	7775	50																		
bread_sliced_spread_dairy_dunno_thick	0%	97%	3%	97%	3%	10047	50	7775	50																		
bread_sliced_spread_dairy_dunno_thin	0%	97%	3%	97%	3%	10047	50	7775	50																		
bread_sliced_spread_dairy_fat_med	0%	97%	3%	97%	3%	7775	100																				
bread_sliced_spread_dairy_fat_thick	0%	97%	3%	97%	3%	7775	100																				
bread_sliced_spread_dairy_fat_thin	0%	97%	3%	97%	3%	7775	100																				
bread_sliced_spread_dairy_lowfat_med	0%	99%	1%	99%	1%	10047	100																				
bread_sliced_spread_dairy_lowfat_thick	0%	99%	1%	99%	1%	10047	100																				
bread_sliced_spread_dairy_lowfat_thin	0%	99%	1%	99%	1%	10047	100																				
bread_sliced_spread_dairy_vlowfat_med	0%	99%	1%	99%	1%	10047	100																				
bread_sliced_spread_dairy_vlowfat_thick	0%	99%	1%	99%	1%	10047	100																				
bread_sliced_spread_dairy_vlowfat_thin	0%	99%	1%	99%	1%	10047	100																				
bread_sliced_spread_dunno_chol_med	0%	97%	3%	97%	3%	3848	33.4	3243	33.3	2849	33.3																
bread_sliced_spread_dunno_chol_thick	0%	97%	3%	97%	3%	3848	33.4	3243	33.3	2849	33.3																
bread_sliced_spread_dunno_chol_thin	0%	97%	3%	97%	3%	3848	33.4	3243	33.3	2849	33.3																
bread_sliced_spread_dunno_dunno_med	0%	97%	3%	97%	3%	10047	25	10049	25	10043	25	7775	25														
bread_sliced_spread_dunno_dunno_thick	0%	97%	3%	97%	3%	10047	25	10049	25	10043	25	7775	25														
bread_sliced_spread_dunno_dunno_thin	0%	97%	3%	97%	3%	10047	25	10049	25	10043	25	7775	25														
bread_sliced_spread_dunno_fat_med	0%	97%	3%	97%	3%	10043	50	7775	50																		
bread_sliced_spread_dunno_fat_thick	0%	97%	3%	97%	3%	10043	50	7775	50																		
bread_sliced_spread_dunno_fat_thin	0%	97%	3%	97%	3%	10043	50	7775	50																		
bread_sliced_spread_dunno_lowfat_med	0%	97%	3%	97%	3%	10047	50	10049	50																		
bread_sliced_spread_dunno_lowfat_thick	0%	97%	3%	97%	3%	10047	50	10049	50																		
bread_sliced_spread_dunno_lowfat_thin	0%	97%	3%	97%	3%	10047	50	10049	50																		
bread_sliced_spread_dunno_vlowfat_med	0%	97%	3%	97%	3%	10047	50	10049	50																		
bread_sliced_spread_dunno_vlowfat_thick	0%	97%	3%	97%	3%	10047	50	10049	50																		
bread_sliced_spread_dunno_vlowfat_thin	0%	97%	3%	97%	3%	10047	50	10049	50																		
bread_sliced_spread_hardmarg_med	0%	100%	0%	100%	0%	860	100																				
bread_sliced_spread_hardmarg_thick	0%	100%	0%	100%	0%	860	100																				
bread_sliced_spread_hardmarg_thin	0%	100%	0%	100%	0%	860	100																				
bread_sliced_spread_olive_chol_med	0%	99%	1%	99%	1%	3364	50	10053	50																		
bread_sliced_spread_olive_chol_thick	0%	99%	1%	99%	1%	3364	50	10053	50																		
bread_sliced_spread_olive_chol_thin	0%	99%	1%	99%	1%	3364	50	10053	50																		
bread_sliced_spread_olive_dunno_med	0%	99%	1%	99%	1%	10048	50	10042	25	10131	25																
bread_sliced_spread_olive_dunno_thick	0%	99%	1%	99%	1%	10048	50	10042	25	10131	25																
bread_sliced_spread_olive_dunno_thin	0%	99%	1%	99%	1%	10048	50	10042	25	10131	25																
bread_sliced_spread_olive_fat_med	0%	99%	1%	99%	1%	10042	75	10131	25																		
bread_sliced_spread_olive_fat_thick	0%	99%	1%	99%	1%	10042	75	10131	25																		
bread_sliced_spread_olive_fat_thin	0%	99%	1%	99%	1%	10042	75	10131	25																		
bread_sliced_spread_olive_lowfat_med	0%	100%	1%	100%	0%	10048	100																				
bread_sliced_spread_olive_lowfat_thick	0%	100%	1%	100%	0%	10048	100																				
bread_sliced_spread_olive_lowfat_thin	0%	100%	1%	100%	0%	10048	100																				
bread_sliced_spread_olive_vlowfat_med	0%	100%	1%	100%	0%	10048	100																				
bread_sliced_spread_olive_vlowfat_thick	0%	100%	1%	100%	0%	10048	100																				
bread_sliced_spread_olive_vlowfat_thin	0%	100%	1%	100%	0%	10048	100																				
bread_sliced_spread_other_med	0%	60%	40%	60%	40%	856	18	851	7.5	852	7.5	3848	33.5	2849	33.5												
bread_sliced_spread_other_thick	0%	60%	40%	60%	40%	856	18	851	7.5	852	7.5	3848	33.5	2849	33.5												
bread_sliced_spread_other_thin	0%	60%	40%	60%	40%	856	18	851	7.5	852	7.5	3848	33.5	2849	33.5												
bread_sliced_spread_polymarg_chol_med	0%	100%	0%	100%	0%	2849	33.4	3848	33.3	3243	33.3																
bread_sliced_spread_polymarg_chol_thick	0%	100%	0%	100%	0%	2849	33.4	3848	33.3	3243	33.3																
bread_sliced_spread_polymarg_chol_thin	0%	100%	0%	100%	0%	2849	33.4	3848	33.3	3243	33.3																
bread_sliced_spread_polymarg_dunno_med	0%	100%	0%	100%	0%	10049	50	10043	25	10044	25																
bread_sliced_spread_polymarg_dunno_thick	0%	100%	0%	100%	0%	10049	50	10043	25	10044	25																
bread_sliced_spread_polymarg_dunno_thin	0%	100%	0%	100%	0%	10049	50	10043	25	10044	25																
bread_sliced_spread_polymarg_fat_med	0%	100%	0%	100%	0%	10043	50	10044	50																		
bread_sliced_spread_polymarg_fat_thick	0%	100%	0%	100%	0%	10043	50	10044	50																		
bread_sliced_spread_polymarg_fat_thin	0%	100%	0%	100%	0%	10043	50	10044	50																		
bread_sliced_spread_polymarg_lowfat_med	0%	100%	0%	100%	0%	10049	100																				
bread_sliced_spread_polymarg_lowfat_thick	0%	100%	0%	100%	0%	10049	100																				
bread_sliced_spread_polymarg_lowfat_thin	0%	100%	0%	100%	0%	10049	100																				
bread_sliced_spread_polymarg_vlowfat_med	0%	100%	0%	100%	0%	10049	100																				
bread_sliced_spread_polymarg_vlowfat_thick	0%	100%	0%	100%	0%	10049	100																				
bread_sliced_spread_polymarg_vlowfat_thin	0%	100%	0%	100%	0%	10049	100																				
bread_sliced_spread_soya_chol_med	0%	100%	1%	100%	1%	3848	16.6	3243	16.6	2849	16.8	10786	50														
bread_sliced_spread_soya_chol_thick	0%	100%	1%	100%	1%	3848	16.6	3243	16.6	2849	16.8	10786	50														
bread_sliced_spread_soya_chol_thin	0%	100%	1%	100%	1%	3848	16.6	3243	16.6	2849	16.8	10786	50														
bread_sliced_spread_soya_dunno_med	0%	100%	0%	100%	0%	10980	50	10786	50																		
bread_sliced_spread_soya_dunno_thick	0%	100%	0%	100%	0%	10980	50	10786	50																		
bread_sliced_spread_soya_dunno_thin	0%	100%	0%	100%	0%	10980	50	10786	50																		
bread_sliced_spread_soya_fat_med	0%	100%	0%	100%	0%	10786	100																				
bread_sliced_spread_soya_fat_thick	0%	100%	0%	100%	0%	10786	100																				
bread_sliced_spread_soya_fat_thin	0%	100%	0%	100%	0%	10786	100																				
bread_sliced_spread_soya_lowfat_med	0%	100%	0%	100%	0%	10980	100																				
bread_sliced_spread_soya_lowfat_thick	0%	100%	0%	100%	0%	10980	100																				
bread_sliced_spread_soya_lowfat_thin	0%	100%	0%	100%	0%	10980	100																				
bread_sliced_spread_soya_vlowfat_med	0%	100%	0%	100%	0%	10980	100																				
bread_sliced_spread_soya_vlowfat_thick	0%	100%	0%	100%	0%	10980	100																				
bread_sliced_spread_soya_vlowfat_thin	0%	100%	0%	100%	0%	10980	100																				
bread_sliced_unanswered	0%	100%	0%	100%	0%	120	33.4	133	33.3	102	33.3																
bread_sliced_white	0%	100%	0%	100%	0%	120	100																				
bread_sliced_wholemeal	0%	100%	0%	100%	0%	133	100																				
cake	99%	30%	70%	20%	80%	3897	20	357	20	3899	20	6965	20	6597	20												
cake_gf	99%	31%	69%	20%	80%	3897	20	357	20	3899	20	6965	20	6597	20												
cereal_bran	88%	100%	0%	100%	0%	202	25	7623	25	201	25	8482	25														
cereal_bran_driedfruit	66%	100%	0%	100%	0%	229	25	5327	25	7624	25	203	25														
cereal_muesli	38%	99%	1%	99%	1%	212	50	214	50																		
cereal_muesli_driedfruit	50%	99%	1%	99%	1%	7629	50	6836	50																		
cereal_oatcrunch	81%	100%	0%	100%	0%	5328	50	8712	50																		
cereal_oatcrunch_driedfruit	72%	100%	0%	100%	0%	5328	45	8712	45	2665	10																
cereal_other	80%	100%	0%	100%	0%	7637	20	8410	40	8103	40																
cereal_other_driedfruit	71%	100%	0%	100%	0%	7637	15	8088	32.5	8103	32.5	2665	20														
cereal_plain	87%	100%	0%	100%	0%	223	33.4	205	16.7	206	16.6	220	16.7	7630	16.6												
cereal_plain_driedfruit	75%	100%	0%	100%	0%	223	22.7	205	11.4	206	11.3	220	11.3	7630	11.3	2665	32										
cereal_porridge_milk_chol	0%	60%	40%	70%	30%	10514	3.5	10515	3.5	9215	7	7183	86														
cereal_porridge_milk_chol_driedfruit	3%	61%	39%	70%	30%	10514	2.75	10515	2.75	9215	5.5	2665	13	7183	76												
cereal_porridge_milk_dontknow	0%	60%	40%	70%	30%	10514	3.5	10515	3.5	9215	7	608	21.5	8543	21.5	613	8.6	8544	8.6	602	8.6	603	8.6	10251	8.6		
cereal_porridge_milk_dontknow_driedfruit	3%	61%	39%	70%	30%	10514	2.75	10515	2.75	9215	5.5	2665	13	608	19	8543	19	613	7.6	8544	7.6	602	7.6	603	7.6	10251	7.6
cereal_porridge_milk_goatsheep	0%	60%	40%	70%	30%	10514	3.5	10515	3.5	9215	7	10397	43	625	21.5	626	21.5										
cereal_porridge_milk_goatsheep_driedfruit	3%	61%	39%	70%	30%	10514	2.75	10515	2.75	9215	5.5	2665	13	10397	38	625	19	626	19								
cereal_porridge_milk_other	0%	60%	40%	70%	30%	10514	3.5	10515	3.5	9215	7	10251	23	9493	14	10932	14	10898	35								
cereal_porridge_milk_other_driedfruit	3%	61%	39%	70%	30%	10514	2.75	10515	2.75	9215	5.5	2665	13	10251	20	9493	13	10932	13	10898	30						
cereal_porridge_milk_powdered	0%	60%	40%	70%	30%	10514	3.5	10515	3.5	9215	7	695	43	696	43												
cereal_porridge_milk_powdered_driedfruit	3%	61%	39%	70%	30%	10514	2.75	10515	2.75	9215	5.5	2665	13	695	38	696	38										
cereal_porridge_milk_riceoatveg	80%	100%	0%	100%	0%	10514	3.5	10515	3.5	9215	7	10572	21.5	10898	21.5	10159	21.5	10836	21.5								
cereal_porridge_milk_riceoatveg_driedfruit	83%	100%	0%	100%	0%	10514	2.75	10515	2.75	9215	5.5	2665	13	10572	19	10898	19	10159	19	10836	19						
cereal_porridge_milk_semi	0%	60%	40%	70%	30%	10514	3.5	10515	3.5	9215	7	608	43	8543	43												
cereal_porridge_milk_semi_driedfruit	3%	61%	39%	70%	30%	10514	2.75	10515	2.75	9215	5.5	2665	13	608	38	8543	38										
cereal_porridge_milk_skimmed	0%	60%	40%	70%	30%	10514	3.5	10515	3.5	9215	7	613	43	8544	43												
cereal_porridge_milk_skimmed_driedfruit	3%	61%	39%	70%	30%	10514	2.75	10515	2.75	9215	5.5	2665	13	613	38	8544	38										
cereal_porridge_milk_soya_ca	80%	100%	0%	100%	0%	10514	3.5	10515	3.5	9215	7	8726	28.7	10974	28.7	3769	28.6										
cereal_porridge_milk_soya_ca_driedfruit	83%	100%	0%	100%	0%	10514	2.75	10515	2.75	9215	5.5	2665	13	8726	25.4	10974	25.3	3769	25.3								
cereal_porridge_milk_soya_noca	80%	100%	0%	100%	0%	10514	3.5	10515	3.5	9215	7	8512	43	650	43												
cereal_porridge_milk_soya_noca_driedfruit	83%	100%	0%	100%	0%	10514	2.75	10515	2.75	9215	5.5	2665	13	8512	38	650	38										

cereal_porridge_milk_whole	0%	60%	40%	70%	30%	10514	3.5	10515	3.5	9215	7	602	43	603	43						
cereal_porridge_milk_whole_driedfruit	3%	61%	39%	70%	30%	10514	2.75	10515	2.75	9215	5.5	2665	13	602	38	603	38				
cereal_porridge_water	0%	100%	0%	100%	0%	10514	3.5	10515	3.5	9215	7	5106	86								
cereal_porridge_water_driedfruit	3%	100%	0%	100%	0%	10514	2.75	10515	2.75	9215	5.5	2665	13	5106	76						
cereal_sugar	100%	75%	25%	100%	0%	2205	25	2217	25	2214	25	2206	25								
cereal_sweet	99%	100%	0%	100%	0%	9275	16.7	227	8.4	7626	8.3	10512	16.7	10513	16.6	204	16.7	8483	16.6		
cereal_sweet_driedfruit	72%	100%	0%	100%	0%	9275	18.3	227	9.1	7626	9.1	10512	18.25	10513	18.3	2665	27				
cereal_wwheat	49%	100%	0%	100%	0%	221	33.4	10510	16.7	10511	16.6	225	33.3								
cereal_wwheat_driedfruit	71%	100%	0%	100%	0%	8190	25	7051	25	6132	50										
cerealbar	98%	90%	10%	80%	20%	6883	10	10060	10	10059	10	10057	10	10058	10	7656	25	8442	25		
cerealbar_gf	98%	91%	9%	80%	20%	6883	10	10060	10	10059	10	10057	10	10058	10	7656	25	8442	25		
cheese_blue	0%	0%	100%	0%	100%	681	33.4	664	33.3	679	33.3										
cheese_cottage	0%	0%	100%	0%	100%	687	33.4	7725	33.3	686	33.3										
cheese_feta	0%	0%	100%	0%	100%	669	100														
cheese_goat	0%	0%	100%	0%	100%	7057	100														
cheese_hard	0%	0%	100%	0%	100%	654	20	665	20	667	20	672	20	676	20						
cheese_hard_lof	0%	0%	100%	0%	100%	659	50	7731	50												
cheese_mozzarella	0%	0%	100%	0%	100%	675	100														
cheese_other	0%	0%	100%	0%	100%	654	20	651	30	683	30	6981	10	7057	10						
cheese_soft	0%	0%	100%	0%	100%	691	50	652	50												
cheese_spread	0%	0%	100%	0%	100%	684	25	2703	50	10413	25										
cheese_spread_lof	0%	0%	100%	0%	100%	10503	16.7	10504	16.7	10505	16.6	6979	25	7112	25						
cheesecake	75%	10%	90%	10%	90%	509	50	510	50												
cheesecake_gf	75%	11%	89%	10%	90%	509	50	510	50												
choc_bar	86%	30%	70%	50%	50%	2265	34	7964	33	7963	33										
choc_dark	100%	100%	0%	100%	0%	2255	100														
choc_milk	84%	20%	80%	50%	50%	2254	100														
choc_sweets	94%	20%	80%	50%	50%	10427	100														
choc_white	82%	20%	80%	50%	50%	7960	100														
chocyog_raisin	65%	30%	70%	50%	50%	7958	50	9274	50												
chutney	92%	100%	0%	100%	0%	2417	25	2440	50	2418	25										
cof_capp	0%	100%	0%	100%	0%	2307	100														
cof_capp_decaf	0%	100%	0%	100%	0%	8312	100														
cof_capp_milk_chol	0%	0%	100%	0%	100%	7183	92.5	5103	7.5												
cof_capp_milk_dontknow	0%	0%	100%	0%	100%	608	23.13	8543	23.1	613	9.25	8544	9.25	602	9.25	603	9.25	10251	9.25	5103	7.5
cof_capp_milk_goatsheep	0%	0%	100%	0%	100%	625	23.13	626	23.1	10397	46.25	5103	7.5								
cof_capp_milk_other	0%	0%	100%	0%	100%	10251	46.25	10932	23.1	9493	23.125	5103	7.5								
cof_capp_milk_powdered	0%	0%	100%	0%	100%	695	46.25	696	46.3	5103	7.5										
cof_capp_milk_riceoatveg	100%	100%	0%	100%	0%	11150	11.1	10572	11.1	10898	23.125	10159	12.03	10966	12	10836	23.1	5103	7.5		
cof_capp_milk_semi	0%	0%	100%	0%	100%	608	46.25	8543	46.3	5103	7.5										
cof_capp_milk_skimmed	0%	0%	100%	0%	100%	613	46.25	8544	46.3	5103	7.5										
cof_capp_milk_soya_ca	100%	100%	0%	100%	0%	8726	31.45	10974	30.5	3769	30.525	5103	7.5								
cof_capp_milk_soya_noca	100%	100%	0%	100%	0%	8512	46.25	650	46.3	5103	7.5										
cof_capp_milk_whole	0%	0%	100%	0%	100%	602	46.25	603	46.3	5103	7.5										
cof_espresso	0%	100%	0%	100%	0%	2307	100														
cof_espresso_decaf	0%	100%	0%	100%	0%	8312	100														
cof_filter	0%	100%	0%	100%	0%	2307	50	2306	50												
cof_filter_decaf	0%	100%	0%	100%	0%	8312	50	8313	50												
cof_instant	0%	100%	0%	100%	0%	2308	0.9	5103	99.1												
cof_instant_decaf	0%	100%	0%	100%	0%	8314	0.9	5103	99.1												
cof_latte	0%	100%	0%	100%	0%	2307	100														
cof_latte_decaf	0%	100%	0%	100%	0%	8312	100														
cof_latte_milk_chol	0%	0%	100%	0%	100%	7183	96.2	5103	3.8												
cof_latte_milk_dontknow	0%	0%	100%	0%	100%	608	24.05	8543	24.1	613	9.62	8544	9.62	602	9.62	603	9.62	10251	9.62	5103	3.8
cof_latte_milk_goatsheep	0%	0%	100%	0%	100%	625	24.05	626	24.1	10397	48.1	5103	3.8								
cof_latte_milk_other	0%	0%	100%	0%	100%	10251	48.1	10932	24.1	9493	24.05	5103	3.8								
cof_latte_milk_powdered	0%	0%	100%	0%	100%	695	48.1	696	48.1	5103	3.8										
cof_latte_milk_riceoatveg	100%	100%	0%	100%	0%	11150	11.54	10572	11.5	10898	24.05	10159	12.51	10966	12.5	10836	24.1	5103	3.8		
cof_latte_milk_semi	0%	0%	100%	0%	100%	608	48.1	8543	48.1	5103	3.8										
cof_latte_milk_skimmed	0%	0%	100%	0%	100%	613	48.1	8544	48.1	5103	3.8										
cof_latte_milk_soya_ca	100%	100%	0%	100%	0%	8726	32.71	10974	31.7	3769	31.746	5103	3.8								
cof_latte_milk_soya_noca	100%	100%	0%	100%	0%	8512	48.1	650	48.1	5103	3.8										
cof_latte_milk_whole	0%	0%	100%	0%	100%	602	48.1	603	48.1	5103	3.8										
cof_other	0%	100%	0%	100%	0%	2308	0.45	5103	49.6	2307	25	2306	25								
cof_other_decaf	0%	100%	0%	100%	0%	8314	0.45	5103	49.6	8312	25	8313	25								
cof_sugar	100%	100%	0%	100%	0%	2205	66.7	2203	33.3												
cream	0%	0%	100%	0%	100%	638	16.7	634	16.7	633	16.6	3014	16.7	632	16.7	644	16.6				
croissant	0%	20%	80%	0%	100%	166	100														
crumble	20%	70%	30%	20%	80%	15	38	24	10	2205	20	852	29	654	3						
danish_pastry	76%	30%	70%	20%	80%	318	100														
dessert_milkbased	96%	20%	80%	20%	80%	582	50	7695	25	9533	25										
dessert_milkpuds	67%	20%	80%	10%	90%	546	17	548	17	559	33	9636	33								
dessert_other	84%	20%	80%	10%	90%	6967	40	1959	22.5	2150	22.5	574	7.5	573	7.5						
dessert_soya	79%	100%	0%	100%	0%	9115	17	10575	17	6541	33	9053	33								
double_crust	0%	60%	40%	5%	95%	400	90	405	10												
doughnut	99%	20%	80%	40%	60%	323	50	7678	25	324	25										
drink_diethotchoc	100%	1%	99%	3%	97%	2633	2.75	7888	2.75	5106	94.5										
drink_fizzy	100%	100%	0%	100%	0%	7895	33.4	8328	33.3	2322	33.3										
drink_grapefruit	100%	100%	0%	100%	0%	2329	50	2330	50												
drink_hotchoc	100%	1%	99%	3%	97%	2309	6.2	2632	6	2310	6	5106	81.8								
drink_hotchoc_milk_chol	0%	0%	100%	0%	100%	7183	100														
drink_hotchoc_milk_dontknow	0%	0%	100%	0%	100%	608	25	8543	25	613	10	8544	10	602	10	603	10	10251	10		
drink_hotchoc_milk_goatsheep	0%	0%	100%	0%	100%	10397	50	625	25	626	25										
drink_hotchoc_milk_other	0%	0%	100%	0%	100%	10251	50	9493	25	10932	25										
drink_hotchoc_milk_powdered	0%	0%	100%	0%	100%	695	50	696	50												
drink_hotchoc_milk_riceoatveg	100%	100%	0%	100%	0%	10572	25	10898	25	10159	25	10836	25								
drink_hotchoc_milk_semi	0%	0%	100%	0%	100%	608	50	8543	50												
drink_hotchoc_milk_skimmed	0%	0%	100%	0%	100%	613	50	8544	50												
drink_hotchoc_milk_soya_ca	100%	100%	0%	100%	0%	8726	34	10974	33	3769	33										
drink_hotchoc_milk_soya_noca	100%	100%	0%	100%	0%	8512	50	650	50												
drink_hotchoc_milk_whole	0%	0%	100%	0%	100%	602	50	603	50												
drink_lowcal	0%	100%	0%	100%	0%	7897	50	7902	25	2351	5	5102	20								
drink_milkbased	64%	1%	99%	2%	98%	711	20	8212	20	629	20	612	20	11128	0.6	11127	0.6	608	9.4	8543	9.4
drink_orange	100%	100%	0%	100%	0%	2337	33.4	2338	33.3	2339	33.3										
drink_other	33%	3%	97%	1%	99%	8333	35	608	15	8543	15	7053	0.09	5103	9.91	2338	12.5	2358	12.5		
drink_purejuice	100%	100%	0%	100%	0%	2319	20	2318	20	2345	20	2355	20	3846	20						
drink_squash	100%	100%	0%	100%	0%	9997	5	5101	60	2358	25	7913	5	2331	5						
drink_water	0%	100%	0%	100%	0%	5000	80	8333	20												
drizzle_oil	0%	100%	0%	100%	0%	874	100														
egg_omelet	0%	0%	100%	0%	100%	7765	25	9303	25	763	50										
egg_other	0%	10%	90%	10%	90%	8711	50	813	25	9861	25										
egg_scotch	0%	10%	90%	10%	90%	814	100														
egg_swich	0%	10%	90%	10%	90%	8066	50	755	45	2432	5										
egg_whole	0%	0%	100%	0%	100%	755	33.4	762	33.3	756	33.3										
fish_battered	0%	5%	95%	90%	10%	1415	50	7808	50												
fish_breaded	0%	5%	95%	50%	50%	11006	16.5	1606	16.5	9524	34	9574	33								
fish_lobcrab	0%	0%	100%	0%	100%	1561	50	1565	50												
fish_oily	0%	0%	100%	0%	100%	1495	10	1502	10	1514	10	9541	30	1523	10	9267	20	9932	10		
fish_other	0%	0%	100%	15%	85%	1522	60	10357	5	1639	15	11004	10	7819	10						
fish_prawns	0%	0%	100%	0%	100%	1573	100														
fish_shell	0%	0%	100%	0%	100%	1576	30	1568	70												
fish_tinnedtuna	0%	0%	100%	0%	100%	1534	50	1533	50												
fish_white	0%	0%	100%	0%	100%	7798	50	7799	50												
fruit_apple	0%	100%	0%	100%	0%	2601	100														
fruit_banana	0%	100%	0%	100%	0%	1977	100														
fruit_berry	0%	100%	0%	100%	0%	2143	25	2153	25	2001	5	11115	25	1985	20						
fruit_cherry	0%	100%	0%	100%	0%	1991	100														
fruit_dried	0%	100%	0%	100%	0%	8547	12.5	1960	12.5	2142	50	2021	12.5	2024	12.5						
fruit_grapefruit	0%	100%	0%	100%	0%	2045	70	2049	30												
fruit_grapes	0%	100%	0%	100%	0%	2041	50	2043	50												
fruit_mango	0%	100%	0%	100%	0%	2078	100														
fruit_melon	0%	100%	0%	100%	0%	2081	33.4	2083	33.3	2085	33.3										
fruit_mixed	0%	100%	0%	100%	0%	2035	35	2030	30	5954	35										
fruit_orange	0%	100%	0%	100%	0%	2092	100														
fruit_other	0%	100%	0%	100%	0%	2061	60	2099	10	8115	10	1963	10	2023	10						
fruit_peach	0%	100%	0%	100%	0%	2101	50	2088	50												
fruit_pear	0%	100%	0%	100%	0%	2241	100														
fruit_pineapple	0%	100%	0%	100%	0%	2117	70	2121	30												
fruit_plum	0%	100%	0%	100%	0%	2123	100														
fruit_prunes	0%	100%	0%	100%	0%	8558	100														
fruit_satsuma	0%	100%	0%	100%	0%	2157	100														
fruit_stewed	0%	100%	0%	100%	0%	1958	33.4	2150	33.3	2129	33.3										
fruitcake	71%	30%	70%	20%	80%	333	50	8105	50												
fruitcake_gf	71%	31%	69%	20%	80%	333	50	8105	50												
grains_couscous	0%	100%	0%	100%	0%	7600	50	8466	50												
grains_other	0%	100%	0%	100%	0%	5	20	7028	35	10424	35	10017	10								
guacamole	0%	90%	10%	90%	10%	8121	75	8126	25												
hummus	0%	100%	0%	100%	0%	1817	75	2978	25												
icecream	76%	0%	100%	60%	40%	720	33.4	6970	16.7	730	33.3	726	16.6								
indian_snack	0%	70%	30%	20%	80%	1797	50	1354	25	2623	25										
jam_honey	97%	80%	20%	100%	0%	2217	35	2214	20	2206	10	8559	35								
mayo	100%	20%	80%	80%	20%	2432	50	2441	50												
mayo_lowfat	100%	20%	80%	80%	20%	2433	50	10805	50												
meat_bacon_nofat	0%	0%	100%	0%	100%	9464	100														
meat_bacon_withfat	0%	0%	100%	0%	100%	914	33.4	915	33.3	916	33.3										
meat_beef_nofat	0%	0%	100%	0%	100%	953	25	970	25	942	25	8264	25								
meat_beef_withfat	0%	0%	100%	1%	99%	952	25	969	25	3829	25	8264	25								
meat_ham_nofat	0%	0%	100%	0%	100%	9508	100														
meat_ham_withfat	0%	0%	100%	0%	100%	8089	80	1274	10	8043	10										
meat_lamb_nofat	0%	0%	100%	0%	100%	980	30	9434	30	1003	20	5217	20								
meat_lamb_withfat	0%	0%	100%	0%	100%	982	30	9433	30	1002	20	5217	20								
meat_liverpate	0%	0%	100%	0%	100%	1191	20	1204	20	1258	60										
meat_other	0%	0%	100%	0%	100%	1131	64	1167	25	1139	11										
meat_pork_nofat	0%	0%	100%	0%	100%	1024	33.4	9451	33.3	9460	33.3										
meat_pork_withfat	0%	0%	100%	0%	100%	1026	33.4	9450	33.3	9460	33.3										
meat_sausage	0%	1%	99%	1%	99%	7791	25	1280	75												
milk_chol_cereal	0%	0%	100%	0%	100%	7183	100														
milk_chol_coffee	0%	0%	100%	0%	100%	7183	100														
milk_chol_glass	0%	0%	100%	0%	100%	7183	100														
milk_chol_tea	0%	0%	100%	0%	100%	7183	100														
milk_dontknow_cereal	0%	0%	100%	0%	100%	608	25	8543	25	613	10	8544	10	602	10	603	10	10251	10		
milk_dontknow_coffee	0%	0%	100%	0%	100%	608	25	8543	25	613	10	8544	10	602	10	603	10	10251	10		
milk_dontknow_glass	0%	0%	100%	0%	100%	608	25	8543	25	613	10	8544	10	602	10	603	10	10251	10		
milk_dontknow_tea	0%	0%	100%	0%	100%	608	25	8543	25	613	10	8544	10	602	10	603	10	10251	10		
milk_goatsheep_cereal	0%	0%	100%	0%	100%	623	16.7	624	16.7	10397	33.3	10416	33.3								
milk_goatsheep_coffee	0%	0%	100%	0%	100%	623	12.5	624	12.5	10397	25	10416	25	625	12.5	626	12.5				
milk_goatsheep_glass	0%	0%	100%	0%	100%	623	12.5	624	12.5	10397	25	10416	25	625	12.5	626	12.5				
milk_goatsheep_tea	0%	0%	100%	0%	100%	623	12.5	624	12.5	10397	25	10416	25	625	12.5	626	12.5				
milk_other_cereal	0%	0%	100%	0%	100%	10251	27	10898	40	9493	16.5	10932	16.5								

milk_other_coffee	0%	0%	100%	0%	100%	10251	27	10898	40	9493	16.5	10932	16.5
milk_other_glass	0%	0%	100%	0%	100%	10251	27	10898	40	9493	16.5	10932	16.5
milk_other_tea	0%	0%	100%	0%	100%	10251	27	10898	40	9493	16.5	10932	16.5
milk_powdered_cereal	0%	0%	100%	0%	100%	695	50	696	50
milk_powdered_coffee	0%	0%	100%	0%	100%	8149	25	10498	25	7717	25	8213	25
milk_powdered_glass	0%	0%	100%	0%	100%	695	50	696	50
milk_powdered_tea	0%	0%	100%	0%	100%	8149	50	10498	50
milk_riceoatveg_cereal	100%	100%	0%	100%	0%	11150	16.7	10898	16.7	10572	16.7	10159	16.7	10966	16.6	10836	16.6
milk_riceoatveg_coffee	100%	100%	0%	100%	0%	11150	16.7	10898	16.7	10572	16.7	10159	16.7	10966	16.6	10836	16.6
milk_riceoatveg_glass	100%	100%	0%	100%	0%	11150	16.7	10898	16.7	10572	16.7	10159	16.7	10966	16.6	10836	16.6
milk_riceoatveg_tea	100%	100%	0%	100%	0%	11150	16.7	10898	16.7	10572	16.7	10159	16.7	10966	16.6	10836	16.6
milk_semi_cereal	0%	0%	100%	0%	100%	608	50	8543	50
milk_semi_coffee	0%	0%	100%	0%	100%	608	50	8543	50
milk_semi_glass	0%	0%	100%	0%	100%	608	50	8543	50
milk_semi_tea	0%	0%	100%	0%	100%	608	50	8543	50
milk_skimmed_cereal	0%	0%	100%	0%	100%	613	50	8544	50
milk_skimmed_coffee	0%	0%	100%	0%	100%	613	50	8544	50
milk_skimmed_glass	0%	0%	100%	0%	100%	613	50	8544	50
milk_skimmed_tea	0%	0%	100%	0%	100%	613	50	8544	50
milk_soya_ca_cereal	100%	100%	0%	100%	0%	8726	34	10974	33	3769	33
milk_soya_ca_coffee	100%	100%	0%	100%	0%	8726	34	10974	33	3769	33
milk_soya_ca_glass	100%	100%	0%	100%	0%	8726	34	10974	33	3769	33
milk_soya_ca_tea	100%	100%	0%	100%	0%	8726	34	10974	33	3769	33
milk_soya_noca_cereal	100%	100%	0%	100%	0%	8512	50	650	50
milk_soya_noca_coffee	100%	100%	0%	100%	0%	8512	50	650	50
milk_soya_noca_glass	100%	100%	0%	100%	0%	8512	50	650	50
milk_soya_noca_tea	100%	100%	0%	100%	0%	8512	50	650	50
milk_whole_cereal	0%	0%	100%	0%	100%	602	50	603	50
milk_whole_coffee	0%	0%	100%	0%	100%	602	50	603	50
milk_whole_glass	0%	0%	100%	0%	100%	602	50	603	50
milk_whole_tea	0%	0%	100%	0%	100%	602	50	603	50
oatcakes	0%	100%	0%	100%	0%	267	100
oatcakes_spread_butter_dunno_med	0%	20%	80%	20%	80%	9407	25	10039	25	851	25	852	25
oatcakes_spread_butter_dunno_thick	0%	20%	80%	20%	80%	9407	25	10039	25	851	25	852	25
oatcakes_spread_butter_dunno_thin	0%	20%	80%	20%	80%	9407	25	10039	25	851	25	852	25
oatcakes_spread_butter_fat_med	0%	0%	100%	0%	100%	851	50	852	50
oatcakes_spread_butter_fat_thick	0%	0%	100%	0%	100%	851	50	852	50
oatcakes_spread_butter_fat_thin	0%	0%	100%	0%	100%	851	50	852	50
oatcakes_spread_butter_lowfat_med	0%	45%	55%	45%	55%	10140	100
oatcakes_spread_butter_lowfat_thick	0%	45%	55%	45%	55%	10140	100
oatcakes_spread_butter_lowfat_thin	0%	45%	55%	45%	55%	10140	100
oatcakes_spread_butter_spread_fat_med	0%	40%	60%	40%	60%	9407	50	10039	50
oatcakes_spread_butter_spread_fat_thick	0%	40%	60%	40%	60%	9407	50	10039	50
oatcakes_spread_butter_spread_fat_thin	0%	40%	60%	40%	60%	9407	50	10039	50
oatcakes_spread_butter_spread_lowfat_med	0%	45%	55%	45%	55%	3891	50	10894	50
oatcakes_spread_butter_spread_lowfat_thick	0%	45%	55%	45%	55%	3891	50	10894	50
oatcakes_spread_butter_spread_lowfat_thin	0%	45%	55%	45%	55%	3891	50	10894	50
oatcakes_spread_dairy_chol_med	0%	97%	3%	97%	3%	3848	100
oatcakes_spread_dairy_chol_thick	0%	97%	3%	97%	3%	3848	100
oatcakes_spread_dairy_chol_thin	0%	97%	3%	97%	3%	3848	100
oatcakes_spread_dairy_dunno_med	0%	97%	3%	97%	3%	10047	50	7775	50
oatcakes_spread_dairy_dunno_thick	0%	97%	3%	97%	3%	10047	50	7775	50
oatcakes_spread_dairy_dunno_thin	0%	97%	3%	97%	3%	10047	50	7775	50
oatcakes_spread_dairy_fat_med	0%	97%	3%	97%	3%	7775	100
oatcakes_spread_dairy_fat_thick	0%	97%	3%	97%	3%	7775	100
oatcakes_spread_dairy_fat_thin	0%	97%	3%	97%	3%	7775	100
oatcakes_spread_dairy_lowfat_med	0%	99%	1%	99%	1%	10047	100
oatcakes_spread_dairy_lowfat_thick	0%	99%	1%	99%	1%	10047	100
oatcakes_spread_dairy_lowfat_thin	0%	99%	1%	99%	1%	10047	100
oatcakes_spread_dairy_vlowfat_med	0%	99%	1%	99%	1%	10047	100
oatcakes_spread_dairy_vlowfat_thick	0%	99%	1%	99%	1%	10047	100
oatcakes_spread_dairy_vlowfat_thin	0%	99%	1%	99%	1%	10047	100
oatcakes_spread_dunno_chol_med	0%	97%	3%	97%	3%	3848	33.4	3243	33.3	2849	33.3
oatcakes_spread_dunno_chol_thick	0%	97%	3%	97%	3%	3848	33.4	3243	33.3	2849	33.3
oatcakes_spread_dunno_chol_thin	0%	97%	3%	97%	3%	3848	33.4	3243	33.3	2849	33.3
oatcakes_spread_dunno_dunno_med	0%	97%	3%	97%	3%	10047	25	10049	25	10043	25	7775	25
oatcakes_spread_dunno_dunno_thick	0%	97%	3%	97%	3%	10047	25	10049	25	10043	25	7775	25
oatcakes_spread_dunno_dunno_thin	0%	97%	3%	97%	3%	10047	25	10049	25	10043	25	7775	25
oatcakes_spread_dunno_fat_med	0%	97%	3%	97%	3%	10043	50	7775	50
oatcakes_spread_dunno_fat_thick	0%	97%	3%	97%	3%	10043	50	7775	50
oatcakes_spread_dunno_fat_thin	0%	97%	3%	97%	3%	10043	50	7775	50
oatcakes_spread_dunno_lowfat_med	0%	97%	3%	97%	3%	10047	50	10049	50
oatcakes_spread_dunno_lowfat_thick	0%	97%	3%	97%	3%	10047	50	10049	50
oatcakes_spread_dunno_lowfat_thin	0%	97%	3%	97%	3%	10047	50	10049	50
oatcakes_spread_dunno_vlowfat_med	0%	97%	3%	97%	3%	10047	50	10049	50
oatcakes_spread_dunno_vlowfat_thick	0%	97%	3%	97%	3%	10047	50	10049	50
oatcakes_spread_dunno_vlowfat_thin	0%	97%	3%	97%	3%	10047	50	10049	50
oatcakes_spread_hardmarg_med	0%	100%	0%	100%	0%	860	100
oatcakes_spread_hardmarg_thick	0%	100%	0%	100%	0%	860	100
oatcakes_spread_hardmarg_thin	0%	100%	0%	100%	0%	860	100
oatcakes_spread_olive_chol_med	0%	99%	1%	99%	1%	3364	50	10053	50
oatcakes_spread_olive_chol_thick	0%	99%	1%	99%	1%	3364	50	10053	50
oatcakes_spread_olive_chol_thin	0%	99%	1%	99%	1%	3364	50	10053	50
oatcakes_spread_olive_dunno_med	0%	99%	1%	99%	1%	10048	50	10042	25	10131	25
oatcakes_spread_olive_dunno_thick	0%	99%	1%	99%	1%	10048	50	10042	25	10131	25
oatcakes_spread_olive_dunno_thin	0%	99%	1%	99%	1%	10048	50	10042	25	10131	25
oatcakes_spread_olive_fat_med	0%	99%	1%	99%	1%	10042	75	10131	25
oatcakes_spread_olive_fat_thick	0%	99%	1%	99%	1%	10042	75	10131	25
oatcakes_spread_olive_fat_thin	0%	99%	1%	99%	1%	10042	75	10131	25
oatcakes_spread_olive_lowfat_med	0%	100%	1%	100%	0%	10048	100
oatcakes_spread_olive_lowfat_thick	0%	100%	1%	100%	0%	10048	100
oatcakes_spread_olive_lowfat_thin	0%	100%	1%	100%	0%	10048	100
oatcakes_spread_olive_vlowfat_med	0%	100%	1%	100%	0%	10048	100
oatcakes_spread_olive_vlowfat_thick	0%	100%	1%	100%	0%	10048	100
oatcakes_spread_olive_vlowfat_thin	0%	100%	1%	100%	0%	10048	100
oatcakes_spread_other_med	0%	60%	40%	60%	40%	856	18	851	7.5	852	7.5	3848	33.5	2849	33.5
oatcakes_spread_other_thick	0%	60%	40%	60%	40%	856	18	851	7.5	852	7.5	3848	33.5	2849	33.5
oatcakes_spread_other_thin	0%	60%	40%	60%	40%	856	18	851	7.5	852	7.5	3848	33.5	2849	33.5
oatcakes_spread_polymarg_chol_med	0%	100%	0%	100%	0%	2849	33.4	3848	33.3	3243	33.3
oatcakes_spread_polymarg_chol_thick	0%	100%	0%	100%	0%	2849	33.4	3848	33.3	3243	33.3
oatcakes_spread_polymarg_chol_thin	0%	100%	0%	100%	0%	2849	33.4	3848	33.3	3243	33.3
oatcakes_spread_polymarg_dunno_med	0%	100%	0%	100%	0%	10049	50	10043	25	10044	25
oatcakes_spread_polymarg_dunno_thick	0%	100%	0%	100%	0%	10049	50	10043	25	10044	25
oatcakes_spread_polymarg_dunno_thin	0%	100%	0%	100%	0%	10049	50	10043	25	10044	25
oatcakes_spread_polymarg_fat_med	0%	100%	0%	100%	0%	10043	50	10044	50
oatcakes_spread_polymarg_fat_thick	0%	100%	0%	100%	0%	10043	50	10044	50
oatcakes_spread_polymarg_fat_thin	0%	100%	0%	100%	0%	10043	50	10044	50
oatcakes_spread_polymarg_lowfat_med	0%	100%	0%	100%	0%	10049	100
oatcakes_spread_polymarg_lowfat_thick	0%	100%	0%	100%	0%	10049	100
oatcakes_spread_polymarg_lowfat_thin	0%	100%	0%	100%	0%	10049	100
oatcakes_spread_polymarg_vlowfat_med	0%	100%	0%	100%	0%	10049	100
oatcakes_spread_polymarg_vlowfat_thick	0%	100%	0%	100%	0%	10049	100
oatcakes_spread_polymarg_vlowfat_thin	0%	100%	0%	100%	0%	10049	100
oatcakes_spread_soya_chol_med	0%	100%	1%	100%	1%	3848	16.6	3243	16.6	2849	16.8	10786	50
oatcakes_spread_soya_chol_thick	0%	100%	1%	100%	1%	3848	16.6	3243	16.6	2849	16.8	10786	50
oatcakes_spread_soya_chol_thin	0%	100%	1%	100%	1%	3848	16.6	3243	16.6	2849	16.8	10786	50
oatcakes_spread_soya_dunno_med	0%	100%	0%	100%	0%	10980	50	10786	50
oatcakes_spread_soya_dunno_thick	0%	100%	0%	100%	0%	10980	50	10786	50
oatcakes_spread_soya_dunno_thin	0%	100%	0%	100%	0%	10980	50	10786	50
oatcakes_spread_soya_fat_med	0%	100%	0%	100%	0%	10786	100
oatcakes_spread_soya_fat_thick	0%	100%	0%	100%	0%	10786	100
oatcakes_spread_soya_fat_thin	0%	100%	0%	100%	0%	10786	100
oatcakes_spread_soya_lowfat_med	0%	100%	0%	100%	0%	10980	100
oatcakes_spread_soya_lowfat_thick	0%	100%	0%	100%	0%	10980	100
oatcakes_spread_soya_lowfat_thin	0%	100%	0%	100%	0%	10980	100
oatcakes_spread_soya_vlowfat_med	0%	100%	0%	100%	0%	10980	100
oatcakes_spread_soya_vlowfat_thick	0%	100%	0%	100%	0%	10980	100
oatcakes_spread_soya_vlowfat_thin	0%	100%	0%	100%	0%	10980	100
pancake_blini_chol	0%	30%	70%	10%	90%	15	26	755	12	871	6	7183	56
pancake_blini_dontknow	0%	30%	70%	10%	90%	15	26	755	12	871	6	608	14	8543	14	613	5.6	8544	5.6	602	5.6	603	5.6	10251	5.6
pancake_blini_goatsheep	0%	30%	70%	10%	90%	15	26	755	12	871	6	10397	28	625	14	626	14
pancake_blini_other	0%	30%	70%	10%	90%	15	26	755	12	871	6	10251	28	9493	14	10932	14
pancake_blini_powdered	0%	30%	70%	10%	90%	15	26	755	12	871	6	695	28	696	28
pancake_blini_riceoatveg	74%	90%	10%	90%	10%	15	26	755	12	871	6	10572	14	10898	14	10159	14	10836	14
pancake_blini_semi	0%	30%	70%	10%	90%	15	26	755	12	871	6	608	28	8543	28
pancake_blini_skimmed	0%	30%	70%	10%	90%	15	26	755	12	871	6	613	28	8544	28
pancake_blini_soya_ca	74%	90%	10%	90%	10%	15	26	755	12	871	6	8726	18.7	10974	18.7	3769	18.6
pancake_blini_soya_noca	74%	90%	10%	90%	10%	15	26	755	12	871	6	8512	28	650	28
pancake_blini_whole	0%	30%	70%	90%	10%	15	26	755	12	871	6	602	28	603	28
pancake_crepe_chol	0%	30%	70%	10%	90%	15	26	755	12	871	6	7183	56
pancake_crepe_dontknow	0%	30%	70%	10%	90%	15	26	755	12	871	6	608	14	8543	14	613	5.6	8544	5.6	602	5.6	603	5.6	10251	5.6
pancake_crepe_goatsheep	0%	30%	70%	10%	90%	15	26	755	12	871	6	10397	28	625	14	626	14
pancake_crepe_other	0%	30%	70%	10%	90%	15	26	755	12	871	6	10251	28	9493	14	10932	14
pancake_crepe_powdered	0%	30%	70%	10%	90%	15	26	755	12	871	6	695	28	696	28
pancake_crepe_riceoatveg	74%	90%	10%	90%	10%	15	26	755	12	871	6	10572	14	10898	14	10159	14	10836	14
pancake_crepe_semi	0%	30%	70%	10%	90%	15	26	755	12	871	6	608	28	8543	28
pancake_crepe_skimmed	0%	30%	70%	10%	90%	15	26	755	12	871	6	613	28	8544	28
pancake_crepe_soya_ca	74%	90%	10%	90%	10%	15	26	755	12	871	6	8726	18.7	10974	18.7	3769	18.6
pancake_crepe_soya_noca	74%	90%	10%	90%	10%	15	26	755	12	871	6	8512	28	650	28
pancake_crepe_whole	0%	30%	70%	90%	10%	15	26	755	12	871	6	602	28	603	28
pasta_brown	0%	100%	0%	100%	0%	36	100
pasta_gf	0%	100%	0%	100%	0%	7100	50	8716	50
pasta_white	0%	50%	50%	60%	40%	9371	25	34	50	32	25
pesto	0%	60%	40%	70%	30%	6036	100
pizza	0%	30%	70%	10%	90%	10026	100
pizza_gf	0%	30%	70%	10%	90%	10026	100
pnutbutter_nutella	100%	100%	1%	100%	1%	2195	70	2212	30
potato_boil	0%	100%	0%	100%	0%	8294	25	1830	25	1829	25	11117	25
potato_boil_marg	0%	50%	50%	50%	50%	10043	25	7775	25	851	25	852	25
potato_fried	0%	100%	0%	100%	0%	10561	20	1841	20	11123	20	1949	20	8684	20
potato_mashed_butter_dunno	0%	70%	30%	10%	90%	1829	89	608	3	8543	3	9407	1.25	10039	1.25	851	1.25	852	1.25
potato_mashed_butter_fat	0%	70%	30%	1%	99%	1829	89	608	3	8543	3	851	2.5	852	2.5
potato_mashed_butter_lowfat	0%	70%	30%	40%	60%	1829	89	608	3	8543	3	10140	5
potato_mashed_butter_spread_fat	0%	70%	30%	30%	70%	1829	89	608	3	8543	3	9407	2.5	10039	2.5
potato_mashed_butter_spread_lowfat	0%	70%	30%	40%	60%	1829	89	608	3	8543	3	10894	2.5	3891	2.5
potato_mashed_fat_dunno	0%	70%	30%	90%	10%	1829	89	608	3	8543	3	875	5
potato_mashed_fat_none	0%	70%	30%	10%	90%	1829	93	608	3.5	8543	3.5
potato_mashed_fat_other	0%	70%	30%	1%	99%	1829	89	608	3	8543	3	856	2.5	855	2.5
potato_mashed_lard	0%	70%	30%	1%	99%	1829	89	608	3	8543	3	858	5

potato_mashed_marg_hard	0%	70%	30%	90%	10%	1829	89	608	3	8543	3	860	5
potato_mashed_marg_poly_chol	0%	70%	30%	90%	10%	1829	89	608	3	8543	3	2849	1.7	3848	1.7	3243	1.6
potato_mashed_marg_poly_dunno	0%	70%	30%	90%	10%	1829	89	608	3	8543	3	10049	2.5	10043	1.25	10044	1.25
potato_mashed_marg_poly_fat	0%	70%	30%	90%	10%	1829	89	608	3	8543	3	10043	2.5	10044	2.5
potato_mashed_marg_poly_lowfat	0%	70%	30%	90%	10%	1829	89	608	3	8543	3	10049	5
potato_mashed_marg_poly_vlowfat	0%	70%	30%	90%	10%	1829	89	608	3	8543	3	10049	5
potato_mashed_marg_soya_chol	0%	70%	30%	90%	10%	1829	89	608	3	8543	3	3848	0.83	3243	0.83	2849	0.84	10786	2.5
potato_mashed_marg_soya_dunno	0%	70%	30%	90%	10%	1829	89	608	3	8543	3	10980	2.5	10786	2.5
potato_mashed_marg_soya_fat	0%	70%	30%	90%	10%	1829	89	608	3	8543	3	10786	5
potato_mashed_marg_soya_lowfat	0%	70%	30%	90%	10%	1829	89	608	3	8543	3	10980	5
potato_mashed_marg_soya_vlowfat	0%	70%	30%	90%	10%	1829	89	608	3	8543	3	10980	5
potato_mashed_oil_olive	0%	70%	30%	90%	10%	1829	89	608	3	8543	3	874	5
potato_mashed_oil_other	0%	70%	30%	90%	10%	1829	89	608	3	8543	3	870	1.5	870	1	10963	1.25	871	0.625	5000	0.63
potato_mashed_oil_rapeseed	0%	70%	30%	90%	10%	1829	89	608	3	8543	3	7990	5
potato_mashed_oil_sunflower	0%	70%	30%	90%	10%	1829	89	608	3	8543	3	873	5
potato_mashed_oil_veg	0%	70%	30%	90%	10%	1829	89	608	3	8543	3	872	2.5	871	2.5
potato_mashed_spread_dairy_chol	0%	70%	30%	80%	20%	1829	89	608	3	8543	3	3848	5
potato_mashed_spread_dairy_dunno	0%	70%	30%	80%	20%	1829	89	608	3	8543	3	10047	2.5	7775	2.5
potato_mashed_spread_dairy_fat	0%	70%	30%	80%	20%	1829	89	608	3	8543	3	7775	5
potato_mashed_spread_dairy_lowfat	0%	70%	30%	80%	20%	1829	89	608	3	8543	3	10047	5
potato_mashed_spread_dairy_vlowfat	0%	70%	30%	80%	20%	1829	89	608	3	8543	3	10047	5
potato_mashed_spread_dunno_chol	0%	70%	30%	80%	20%	1829	89	608	3	8543	3	3848	1.7	3243	1.7	2849	1.6
potato_mashed_spread_dunno_dunno	0%	70%	30%	80%	20%	1829	89	608	3	8543	3	10047	1.25	10049	1.25	10043	1.25	7775	1.25
potato_mashed_spread_dunno_fat	0%	70%	30%	80%	20%	1829	89	608	3	8543	3	10043	2.5	7775	2.5
potato_mashed_spread_dunno_lowfat	0%	70%	30%	80%	20%	1829	89	608	3	8543	3	10047	2.5	10049	2.5
potato_mashed_spread_dunno_vlowfat	0%	70%	30%	80%	20%	1829	89	608	3	8543	3	10047	2.5	10049	2.5
potato_mashed_spread_olive_chol	0%	70%	30%	80%	20%	1829	89	608	3	8543	3	3364	2.5	10053	2.5
potato_mashed_spread_olive_dunno	0%	70%	30%	80%	20%	1829	89	608	3	8543	3	10048	2.5	10042	1.25	10131	1.25
potato_mashed_spread_olive_fat	0%	70%	30%	80%	20%	1829	89	608	3	8543	3	10042	3.75	10131	1.25
potato_mashed_spread_olive_lowfat	0%	70%	30%	80%	20%	1829	89	608	3	8543	3	10048	5
potato_mashed_spread_olive_vlowfat	0%	70%	30%	80%	20%	1829	89	608	3	8543	3	10048	5
poultry_friedbcrumb_noskin	0%	1%	99%	10%	90%	5263	67	1087	33
poultry_friedbcrumb_withskin	0%	1%	99%	10%	90%	5263	67	1087	33
poultry_noskin	0%	0%	100%	1%	99%	1091	30	1090	30	5270	15	5265	15	1147	10
poultry_withskin	0%	0%	100%	0%	100%	1089	60	5271	15	5265	15	1146	10
rice_brown	0%	100%	0%	100%	0%	49	33.4	10009	33.3	10011	33.3
rice_white	0%	100%	0%	100%	0%	58	33.4	42	33.3	55	33.3
salad_dressing	100%	100%	0%	100%	0%	9391	100
sauce_brown	72%	100%	0%	100%	0%	2412	50	2409	50
sauce_cheese_chol	0%	10%	90%	10%	90%	8947	12.5	7183	87.5
sauce_cheese_dontknow	0%	10%	90%	10%	90%	8947	12.5	608	21.9	8543	21.875	613	8.75	8544	8.75	602	8.75	603	8.75	10251	8.75
sauce_cheese_goatsheep	0%	10%	90%	10%	90%	8947	12.5	10397	43.8	625	21.875	626	21.88
sauce_cheese_other	0%	10%	90%	10%	90%	8947	12.5	10251	43.8	9493	21.875	10932	21.88
sauce_cheese_powdered	0%	10%	90%	10%	90%	8947	12.5	695	43.8	696	43.75
sauce_cheese_riceoatveg	88%	30%	70%	30%	70%	8947	12.5	10572	21.9	10898	21.875	10159	21.88	10836	21.9
sauce_cheese_semi	0%	10%	90%	10%	90%	8947	12.5	608	43.8	8543	43.75
sauce_cheese_skimmed	0%	10%	90%	10%	90%	8947	12.5	613	43.8	8544	43.75
sauce_cheese_soya_ca	88%	50%	50%	50%	50%	8947	12.5	8726	29.2	10974	29.17	3769	29.16
sauce_cheese_soya_noca	88%	50%	50%	50%	50%	8947	12.5	8512	43.8	650	43.75
sauce_cheese_whole	0%	10%	90%	10%	90%	8947	12.5	602	43.8	603	43.75
sauce_gravy	0%	10%	90%	10%	90%	2426	50	2427	50
sauce_ketchup	84%	100%	0%	100%	0%	2448	100
sauce_tomato	90%	70%	30%	30%	70%	2450	50	3997	25	10311	25
sauce_white_chol	0%	10%	90%	10%	90%	9377	12.5	7183	87.5
sauce_white_dontknow	0%	10%	90%	10%	90%	9377	12.5	608	21.9	8543	21.875	613	8.75	8544	8.75	602	8.75	603	8.75	10251	8.75
sauce_white_goatsheep	0%	10%	90%	10%	90%	9377	12.5	10397	43.8	625	21.875	626	21.88
sauce_white_other	0%	10%	90%	10%	90%	9377	12.5	10251	43.8	9493	21.875	10932	21.88
sauce_white_powdered	0%	10%	90%	10%	90%	9377	12.5	695	43.8	696	43.75
sauce_white_riceoatveg	96%	30%	70%	30%	70%	9377	12.5	10572	21.9	10898	21.875	10159	21.88	10836	21.9
sauce_white_semi	0%	10%	90%	10%	90%	9377	12.5	608	43.8	8543	43.75
sauce_white_skimmed	0%	10%	90%	10%	90%	9377	12.5	613	43.8	8544	43.75
sauce_white_soya_ca	96%	50%	50%	50%	50%	9377	12.5	8726	29.2	10974	29.17	3769	29.16
sauce_white_soya_noca	96%	50%	50%	50%	50%	9377	12.5	8512	43.8	650	43.75
sauce_white_whole	0%	10%	90%	10%	90%	9377	12.5	602	43.8	603	43.75
scone	60%	30%	70%	10%	90%	369	40	368	40	367	20
scone_gf	76%	30%	70%	11%	89%	8870	100
single_crust	0%	60%	40%	5%	95%	393	40	400	50	405	10
smoothie_dairy	47%	3%	97%	5%	95%	10038	100
smoothie_fruit	78%	100%	0%	100%	0%	8023	20	8024	20	10534	20	10746	20	10747	20
snack_cheesybis	0%	50%	50%	50%	50%	251	100
snack_crisps	0%	100%	0%	100%	0%	10004	16.7	7872	25	10070	25	10001	16.7	10000	16.6
snack_olives	0%	100%	0%	100%	0%	2090	100
snack_saltednuts	0%	100%	0%	100%	0%	7304	25	8548	25	7884	50
snack_saltedpeanuts	0%	100%	0%	100%	0%	2193	100
snack_savourybis	0%	100%	0%	100%	0%	3236	34	3973	33	8155	33
snack_seeds	0%	100%	0%	100%	0%	2168	25	2167	25	2166	25	9741	25
snack_svyother	0%	100%	0%	100%	0%	2605	10	10563	30	10000	30	6825	20	4408	10
snack_swtother	83%	30%	70%	50%	50%	8136	10	8193	30	7662	30	2254	20	2269	10
snack_unsaltednuts	0%	100%	0%	100%	0%	2177	25	2199	25	2169	25	2197	25
snack_unsaltedpeanuts	0%	100%	0%	100%	0%	2191	100
snackpot	71%	60%	40%	60%	40%	10392	100
soup_canned_fish	0%	30%	70%	20%	80%	2951	100
soup_canned_meat	67%	30%	70%	20%	80%	2463	33.4	7925	33.3	2473	33.3
soup_canned_other	50%	70%	30%	60%	40%	2483	50	10960	40	7926	10
soup_canned_pasta	0%	70%	30%	20%	80%	8890	50	2467	50
soup_canned_pulse	60%	80%	20%	60%	40%	3772	50	10960	50
soup_canned_unanswered	60%	80%	20%	70%	30%	3772	33.3	2463	33.3	2483	33.4
soup_canned_veg	50%	100%	0%	100%	0%	7926	12.5	2478	25	2483	25	10731	12.5	2472	12.5	9971	12.5
soup_homemade_fish	0%	30%	70%	20%	80%	2951	100
soup_homemade_meat	0%	30%	70%	20%	80%	5668	50	6790	50
soup_homemade_other	0%	70%	30%	60%	40%	8659	10	9075	40	9166	50
soup_homemade_pasta	0%	70%	30%	20%	80%	8890	100
soup_homemade_pulse	0%	80%	20%	60%	40%	2469	50	2476	50
soup_homemade_unanswered	0%	80%	20%	70%	30%	2469	33.3	5668	33.3	9166	33.4
soup_homemade_veg	0%	100%	0%	100%	0%	8659	20	6880	20	9166	20	5997	20	5996	20
soup_powder	97%	60%	40%	50%	50%	2468	100
spongepuds	81%	20%	80%	20%	80%	567	34	11010	33	5749	33
spongepuds_gf	81%	21%	79%	20%	80%	567	34	11010	33	5749	33
spreadsauce_other	100%	50%	50%	50%	50%	2454	12.5	8074	12.5	9392	12.5	2159	12.5	3980	12.5	3981	12.5	2435	12.5	2428	12.5
sushi	16%	20%	80%	20%	80%	9153	33.4	2820	33.3	2821	33.3
sweets	100%	20%	80%	20%	80%	2251	25	2272	25	11013	12.5	8303	25	11014	12.5
sweets_diet	0%	20%	80%	20%	80%	8646	34	7968	33	10707	33
tea_black	0%	100%	0%	100%	0%	2314	50	2315	50
tea_black_decaf	0%	100%	0%	100%	0%	8316	50	8317	50
tea_green	0%	100%	0%	100%	0%	8006	100
tea_herbal	0%	100%	0%	100%	0%	5341	50	5340	50
tea_other	0%	100%	0%	100%	0%	2316	1.3	5104	48.7	5341	20	2314	15	2315	15
tea_rooibos	0%	100%	0%	100%	0%	5341	100
tea_sugar	100%	100%	0%	100%	0%	2205	100
veg_avocado	0%	100%	0%	100%	0%	1976	100
veg_bakedbeans	90%	100%	0%	100%	0%	1662	75	2646	25
veg_beetroot	0%	100%	0%	100%	0%	2456	50	1691	50
veg_broadbeans	0%	100%	0%	100%	0%	1666	50	1668	50
veg_broccoli	0%	100%	0%	100%	0%	11109	75	1694	25
veg_butternut	0%	100%	0%	100%	0%	3954	100
veg_cabbagekale	0%	100%	0%	100%	0%	1705	33.4	7845	33.3	1708	33.3
veg_carrots	0%	100%	0%	100%	0%	1711	40	1713	30	1712	10	1714	20
veg_cauli	0%	100%	0%	100%	0%	1719	100
veg_celery	0%	100%	0%	100%	0%	1725	100
veg_courgette_butter_dunno	0%	99%	1%	10%	90%	1734	97.5	9407	0.63	10039	0.625	851	0.625	852	0.63
veg_courgette_butter_fat	0%	99%	1%	1%	99%	1734	97.5	851	1.25	852	1.25
veg_courgette_butter_lowfat	0%	99%	1%	40%	60%	1734	97.5	10140	2.5
veg_courgette_butter_spread_fat	0%	99%	1%	30%	70%	1734	97.5	9407	1.25	10039	1.25
veg_courgette_butter_spread_lowfat	0%	99%	1%	40%	60%	1734	97.5	10894	1.25	3891	1.25
veg_courgette_fat_dunno	0%	99%	1%	100%	0%	1734	97.5	875	2.5
veg_courgette_fat_none	0%	100%	0%	100%	0%	1734	100
veg_courgette_fat_other	0%	99%	1%	1%	99%	1734	97.5	856	1.25	855	1.25
veg_courgette_lard	0%	99%	1%	1%	99%	1734	97.5	858	2.5
veg_courgette_marg_hard	0%	99%	1%	99%	1%	1734	97.5	860	2.5
veg_courgette_marg_poly_chol	0%	99%	1%	90%	10%	1734	97.5	2849	0.84	3848	0.83	3243	0.83
veg_courgette_marg_poly_dunno	0%	99%	1%	90%	10%	1734	97.5	10049	1.25	10043	0.625	10044	0.625
veg_courgette_marg_poly_fat	0%	99%	1%	90%	10%	1734	97.5	10043	1.25	10044	1.25
veg_courgette_marg_poly_lowfat	0%	99%	1%	90%	10%	1734	97.5	10049	2.5
veg_courgette_marg_poly_vlowfat	0%	99%	1%	90%	10%	1734	97.5	10049	2.5
veg_courgette_marg_soya_chol	0%	99%	1%	99%	1%	1734	97.5	3848	0.42	3243	0.41	2849	0.42	10786	1.25
veg_courgette_marg_soya_dunno	0%	99%	1%	100%	0%	1734	97.5	10980	1.25	10786	1.25
veg_courgette_marg_soya_fat	0%	99%	1%	100%	0%	1734	97.5	10786	2.5
veg_courgette_marg_soya_lowfat	0%	99%	1%	100%	0%	1734	97.5	10980	2.5
veg_courgette_marg_soya_vlowfat	0%	99%	1%	100%	0%	1734	97.5	10980	2.5
veg_courgette_oil_olive	0%	100%	0%	100%	0%	1734	97.5	874	2.5
veg_courgette_oil_other	0%	100%	0%	100%	0%	1734	97.5	870	0.75	870	0.5	10963	0.625	871	0.31	5000	0.31
veg_courgette_oil_rapeseed	0%	100%	0%	100%	0%	1734	97.5	7990	2.5
veg_courgette_oil_sunflower	0%	100%	0%	100%	0%	1734	97.5	873	2.5
veg_courgette_oil_veg	0%	100%	0%	100%	0%	1734	97.5	872	1.25	871	1.25
veg_courgette_spread_dairy_chol	0%	99%	1%	99%	1%	1734	97.5	3848	2.5
veg_courgette_spread_dairy_dunno	0%	99%	1%	99%	1%	1734	97.5	10047	1.25	7775	1.25
veg_courgette_spread_dairy_fat	0%	99%	1%	99%	1%	1734	97.5	7775	2.5
veg_courgette_spread_dairy_lowfat	0%	99%	1%	99%	1%	1734	97.5	10047	2.5
veg_courgette_spread_dairy_vlowfat	0%	99%	1%	99%	1%	1734	97.5	10047	2.5
veg_courgette_spread_dunno_chol	0%	99%	1%	99%	1%	1734	97.5	3848	0.84	3243	0.83	2849	0.83
veg_courgette_spread_dunno_dunno	0%	99%	1%	99%	1%	1734	97.5	10047	0.63	10049	0.625	10043	0.625	7775	0.63
veg_courgette_spread_dunno_fat	0%	99%	1%	99%	1%	1734	97.5	10043	1.25	7775	1.25
veg_courgette_spread_dunno_lowfat	0%	99%	1%	99%	1%	1734	97.5	10047	1.25	10049	1.25
veg_courgette_spread_dunno_vlowfat	0%	99%	1%	99%	1%	1734	97.5	10047	1.25	10049	1.25
veg_courgette_spread_olive_chol	0%	99%	1%	99%	1%	1734	97.5	3364	1.25	10053	1.25
veg_courgette_spread_olive_dunno	0%	99%	1%	99%	1%	1734	97.5	10048	1.25	10042	0.625	10131	0.625
veg_courgette_spread_olive_fat	0%	99%	1%	99%	1%	1734	97.5	10042	1.88	10131	0.625
veg_courgette_spread_olive_lowfat	0%	99%	1%	99%	1%	1734	97.5	10048	2.5
veg_courgette_spread_olive_vlowfat	0%	99%	1%	99%	1%	1734	97.5	10048	2.5
veg_cucumber	0%	100%	0%	100%	0%	1740	100
veg_garlic	0%	100%	0%	100%	0%	1743	100
veg_greenbeans	0%	100%	0%	100%	0%	1671	75	1681	12.5	1683	12.5
veg_leek_butter_dunno	0%	99%	1%	10%	90%	1756	98.75	9407	0.31	10039	0.3125	851	0.313	852	0.31
veg_leek_butter_fat	0%	99%	1%	1%	99%	1756	98.75	851	0.63	852	0.625
veg_leek_butter_lowfat	0%	99%	1%	40%	60%	1756	98.75	10140	1.25
veg_leek_butter_spread_fat	0%	99%	1%	30%	70%	1756	98.75	9407	0.63	10039	0.625
veg_leek_butter_spread_lowfat	0%	99%	1%	40%	60%	1756	98.75	10894	0.63	3891	0.625
veg_leek_fat_dunno	0%	99%	1%	100%	0%	1756	98.75	875	1.25
veg_leek_fat_none	0%	100%	0%	100%	0%	1756	100

veg_leek_fat_other	0%	99%	1%	1%	99%	1756	98.75	856	0.63	855	0.625
veg_leek_lard	0%	99%	1%	1%	99%	1756	98.75	858	1.25
veg_leek_marg_hard	0%	99%	1%	99%	1%	1756	98.75	860	1.25
veg_leek_marg_poly_chol	0%	99%	1%	90%	10%	1756	98.75	2849	0.42	3848	0.42	3243	0.41
veg_leek_marg_poly_dunno	0%	99%	1%	90%	10%	1756	98.75	10049	0.63	10043	0.3125	10044	0.313
veg_leek_marg_poly_fat	0%	99%	1%	90%	10%	1756	98.75	10043	0.63	10044	0.625
veg_leek_marg_poly_lowfat	0%	99%	1%	90%	10%	1756	98.75	10049	1.25
veg_leek_marg_poly_vlowfat	0%	99%	1%	90%	10%	1756	98.75	10049	1.25
veg_leek_marg_soya_chol	0%	99%	1%	99%	1%	1756	98.75	3848	0.21	3243	0.21	2849	0.205	10786	0.63
veg_leek_marg_soya_dunno	0%	99%	1%	100%	0%	1756	98.75	10980	0.63	10786	0.625
veg_leek_marg_soya_fat	0%	99%	1%	100%	0%	1756	98.75	10786	1.25
veg_leek_marg_soya_lowfat	0%	99%	1%	100%	0%	1756	98.75	10980	1.25
veg_leek_marg_soya_vlowfat	0%	99%	1%	100%	0%	1756	98.75	10980	1.25
veg_leek_oil_olive	0%	100%	0%	100%	0%	1756	98.75	874	1.25
veg_leek_oil_other	0%	100%	0%	100%	0%	1756	98.75	870	0.38	870	0.25	10963	0.31	871	0.16	5000	0.15
veg_leek_oil_rapeseed	0%	100%	0%	100%	0%	1756	98.75	7990	1.25
veg_leek_oil_sunflower	0%	100%	0%	100%	0%	1756	98.75	873	1.25
veg_leek_oil_veg	0%	100%	0%	100%	0%	1756	98.75	872	0.63	871	0.625
veg_leek_spread_dairy_chol	0%	99%	1%	99%	1%	1756	98.75	3848	1.25
veg_leek_spread_dairy_dunno	0%	99%	1%	99%	1%	1756	98.75	10047	0.63	7775	0.625
veg_leek_spread_dairy_fat	0%	99%	1%	99%	1%	1756	98.75	7775	1.25
veg_leek_spread_dairy_lowfat	0%	99%	1%	99%	1%	1756	98.75	10047	1.25
veg_leek_spread_dairy_vlowfat	0%	99%	1%	99%	1%	1756	98.75	10047	1.25
veg_leek_spread_dunno_chol	0%	99%	1%	99%	1%	1756	98.75	3848	0.42	3243	0.42	2849	0.41
veg_leek_spread_dunno_dunno	0%	99%	1%	99%	1%	1756	98.75	10047	0.31	10049	0.3125	10043	0.313	7775	0.31
veg_leek_spread_dunno_fat	0%	99%	1%	99%	1%	1756	98.75	10043	0.63	7775	0.625
veg_leek_spread_dunno_lowfat	0%	99%	1%	99%	1%	1756	98.75	10047	0.63	10049	0.625
veg_leek_spread_dunno_vlowfat	0%	99%	1%	99%	1%	1756	98.75	10047	0.63	10049	0.625
veg_leek_spread_olive_chol	0%	99%	1%	99%	1%	1756	98.75	3364	0.63	10053	0.625
veg_leek_spread_olive_dunno	0%	99%	1%	99%	1%	1756	98.75	10048	0.63	10042	0.31	10131	0.31
veg_leek_spread_olive_fat	0%	99%	1%	99%	1%	1756	98.75	10042	0.94	10131	0.3125
veg_leek_spread_olive_lowfat	0%	99%	1%	99%	1%	1756	98.75	10048	1.25
veg_leek_spread_olive_vlowfat	0%	99%	1%	99%	1%	1756	98.75	10048	1.25
veg_lettuce	0%	100%	0%	100%	0%	1762	100
veg_mixed	0%	100%	0%	100%	0%	1771	100
veg_mixtures_butter_dunno	0%	99%	1%	10%	90%	8377	95	9407	1.25	10039	1.25	851	1.25	852	1.25
veg_mixtures_butter_fat	0%	99%	1%	1%	99%	8377	95	851	2.5	852	2.5
veg_mixtures_butter_lowfat	0%	99%	1%	40%	60%	8377	95	10140	5
veg_mixtures_butter_spread_fat	0%	99%	1%	30%	70%	8377	95	9407	2.5	10039	2.5
veg_mixtures_butter_spread_lowfat	0%	99%	1%	40%	60%	8377	95	10894	2.5	3891	2.5
veg_mixtures_fat_dunno	0%	99%	1%	100%	0%	8377	95	875	5
veg_mixtures_fat_none	0%	99%	1%	100%	0%	8377	100
veg_mixtures_fat_other	0%	99%	1%	1%	99%	8377	95	856	2.5	855	2.5
veg_mixtures_lard	0%	99%	1%	1%	99%	8377	95	858	5
veg_mixtures_marg_hard	0%	99%	1%	99%	1%	8377	95	860	5
veg_mixtures_marg_poly_chol	0%	99%	1%	90%	10%	8377	95	2849	1.7	3848	1.7	3243	1.6
veg_mixtures_marg_poly_dunno	0%	99%	1%	90%	10%	8377	95	10049	2.5	10043	1.25	10044	1.25
veg_mixtures_marg_poly_fat	0%	99%	1%	90%	10%	8377	95	10043	2.5	10044	2.5
veg_mixtures_marg_poly_lowfat	0%	99%	1%	90%	10%	8377	95	10049	5
veg_mixtures_marg_poly_vlowfat	0%	99%	1%	90%	10%	8377	95	10049	5
veg_mixtures_marg_soya_chol	0%	99%	1%	99%	1%	8377	95	3848	0.83	3243	0.83	2849	0.84	10786	2.5
veg_mixtures_marg_soya_dunno	0%	99%	1%	100%	0%	8377	95	10980	2.5	10786	2.5
veg_mixtures_marg_soya_fat	0%	99%	1%	100%	0%	8377	95	10786	5
veg_mixtures_marg_soya_lowfat	0%	99%	1%	100%	0%	8377	95	10980	5
veg_mixtures_marg_soya_vlowfat	0%	99%	1%	100%	0%	8377	95	10980	5
veg_mixtures_oil_olive	0%	100%	0%	100%	0%	8377	95	874	5
veg_mixtures_oil_other	0%	100%	0%	100%	0%	8377	95	870	1.5	870	1	10963	1.25	871	0.63	5000	0.63
veg_mixtures_oil_rapeseed	0%	100%	0%	100%	0%	8377	95	7990	5
veg_mixtures_oil_sunflower	0%	100%	0%	100%	0%	8377	95	873	5
veg_mixtures_oil_veg	0%	100%	0%	100%	0%	8377	95	872	2.5	871	2.5
veg_mixtures_spread_dairy_chol	0%	99%	1%	99%	1%	8377	95	3848	5
veg_mixtures_spread_dairy_dunno	0%	99%	1%	99%	1%	8377	95	10047	2.5	7775	2.5
veg_mixtures_spread_dairy_fat	0%	99%	1%	99%	1%	8377	95	7775	5
veg_mixtures_spread_dairy_lowfat	0%	99%	1%	99%	1%	8377	95	10047	5
veg_mixtures_spread_dairy_vlowfat	0%	99%	1%	99%	1%	8377	95	10047	5
veg_mixtures_spread_dunno_chol	0%	99%	1%	99%	1%	8377	95	3848	1.7	3243	1.7	2849	1.6
veg_mixtures_spread_dunno_dunno	0%	99%	1%	99%	1%	8377	95	10047	1.25	10049	1.25	10043	1.25	7775	1.25
veg_mixtures_spread_dunno_fat	0%	99%	1%	99%	1%	8377	95	10043	2.5	7775	2.5
veg_mixtures_spread_dunno_lowfat	0%	99%	1%	99%	1%	8377	95	10047	2.5	10049	2.5
veg_mixtures_spread_dunno_vlowfat	0%	99%	1%	99%	1%	8377	95	10047	2.5	10049	2.5
veg_mixtures_spread_olive_chol	0%	99%	1%	99%	1%	8377	95	3364	2.5	10053	2.5
veg_mixtures_spread_olive_dunno	0%	99%	1%	99%	1%	8377	95	10048	2.5	10042	1.25	10131	1.25
veg_mixtures_spread_olive_fat	0%	99%	1%	99%	1%	8377	95	10042	3.75	10131	1.25
veg_mixtures_spread_olive_lowfat	0%	99%	1%	99%	1%	8377	95	10048	5
veg_mixtures_spread_olive_vlowfat	0%	99%	1%	99%	1%	8377	95	10048	5
veg_mushrooms_butter_dunno	0%	99%	1%	10%	90%	1779	97.5	9407	0.63	10039	0.625	851	0.625	852	0.63
veg_mushrooms_butter_fat	0%	99%	1%	1%	99%	1779	97.5	851	1.25	852	1.25
veg_mushrooms_butter_lowfat	0%	99%	1%	40%	60%	1779	97.5	10140	2.5
veg_mushrooms_butter_spread_fat	0%	99%	1%	30%	70%	1779	97.5	9407	1.25	10039	1.25
veg_mushrooms_butter_spread_lowfat	0%	99%	1%	40%	60%	1779	97.5	10894	1.25	3891	1.25
veg_mushrooms_fat_dunno	0%	99%	1%	100%	0%	1779	97.5	875	2.5
veg_mushrooms_fat_none	0%	100%	0%	100%	0%	1779	100
veg_mushrooms_fat_other	0%	99%	1%	1%	99%	1779	97.5	856	1.25	855	1.25
veg_mushrooms_lard	0%	99%	1%	1%	99%	1779	97.5	858	2.5
veg_mushrooms_marg_hard	0%	99%	1%	99%	1%	1779	97.5	860	2.5
veg_mushrooms_marg_poly_chol	0%	99%	1%	90%	10%	1779	97.5	2849	0.84	3848	0.83	3243	0.83
veg_mushrooms_marg_poly_dunno	0%	99%	1%	90%	10%	1779	97.5	10049	1.25	10043	0.625	10044	0.625
veg_mushrooms_marg_poly_fat	0%	99%	1%	90%	10%	1779	97.5	10043	1.25	10044	1.25
veg_mushrooms_marg_poly_lowfat	0%	99%	1%	90%	10%	1779	97.5	10049	2.5
veg_mushrooms_marg_poly_vlowfat	0%	99%	1%	90%	10%	1779	97.5	10049	2.5
veg_mushrooms_marg_soya_chol	0%	99%	1%	99%	1%	1779	97.5	3848	0.42	3243	0.41	2849	0.42	10786	1.25
veg_mushrooms_marg_soya_dunno	0%	99%	1%	100%	0%	1779	97.5	10980	1.25	10786	1.25
veg_mushrooms_marg_soya_fat	0%	99%	1%	100%	0%	1779	97.5	10786	2.5
veg_mushrooms_marg_soya_lowfat	0%	99%	1%	100%	0%	1779	97.5	10980	2.5
veg_mushrooms_marg_soya_vlowfat	0%	99%	1%	100%	0%	1779	97.5	10980	2.5
veg_mushrooms_oil_olive	0%	100%	0%	100%	0%	1779	97.5	874	2.5
veg_mushrooms_oil_other	0%	100%	0%	100%	0%	1779	97.5	870	0.75	870	0.5	10963	0.625	871	0.31	5000	0.31
veg_mushrooms_oil_rapeseed	0%	100%	0%	100%	0%	1779	97.5	7990	2.5
veg_mushrooms_oil_sunflower	0%	100%	0%	100%	0%	1779	97.5	873	2.5
veg_mushrooms_oil_veg	0%	100%	0%	100%	0%	1779	97.5	872	1.25	871	1.25
veg_mushrooms_spread_dairy_chol	0%	99%	1%	99%	1%	1779	97.5	3848	2.5
veg_mushrooms_spread_dairy_dunno	0%	99%	1%	99%	1%	1779	97.5	10047	1.25	7775	1.25
veg_mushrooms_spread_dairy_fat	0%	99%	1%	99%	1%	1779	97.5	7775	2.5
veg_mushrooms_spread_dairy_lowfat	0%	99%	1%	99%	1%	1779	97.5	10047	2.5
veg_mushrooms_spread_dairy_vlowfat	0%	99%	1%	99%	1%	1779	97.5	10047	2.5
veg_mushrooms_spread_dunno_chol	0%	99%	1%	99%	1%	1779	97.5	3848	0.84	3243	0.83	2849	0.83
veg_mushrooms_spread_dunno_dunno	0%	99%	1%	99%	1%	1779	97.5	10047	0.63	10049	0.625	10043	0.625	7775	0.63
veg_mushrooms_spread_dunno_fat	0%	99%	1%	99%	1%	1779	97.5	10043	1.25	7775	1.25
veg_mushrooms_spread_dunno_lowfat	0%	99%	1%	99%	1%	1779	97.5	10047	1.25	10049	1.25
veg_mushrooms_spread_dunno_vlowfat	0%	99%	1%	99%	1%	1779	97.5	10047	1.25	10049	1.25
veg_mushrooms_spread_olive_chol	0%	99%	1%	99%	1%	1779	97.5	3364	1.25	10053	1.25
veg_mushrooms_spread_olive_dunno	0%	99%	1%	99%	1%	1779	97.5	10048	1.25	10042	0.625	10131	0.625
veg_mushrooms_spread_olive_fat	0%	99%	1%	99%	1%	1779	97.5	10042	1.88	10131	0.625
veg_mushrooms_spread_olive_lowfat	0%	99%	1%	99%	1%	1779	97.5	10048	2.5
veg_mushrooms_spread_olive_vlowfat	0%	99%	1%	99%	1%	1779	97.5	10048	2.5
veg_onion_butter_dunno	0%	99%	1%	10%	90%	1785	20	9293	77.5	9407	0.625	10039	0.625	851	0.63	852	0.63
veg_onion_butter_fat	0%	99%	1%	1%	99%	1785	20	9293	77.5	851	1.25	852	1.25
veg_onion_butter_lowfat	0%	99%	1%	40%	60%	1785	20	9293	77.5	10140	2.5
veg_onion_butter_spread_fat	0%	99%	1%	30%	70%	1785	20	9293	77.5	9407	1.25	10039	1.25
veg_onion_butter_spread_lowfat	0%	99%	1%	40%	60%	1785	20	9293	77.5	10894	1.25	3891	1.25
veg_onion_fat_dunno	0%	99%	1%	100%	0%	1785	20	9293	77.5	875	2.5
veg_onion_fat_none	0%	100%	0%	100%	0%	1785	20	9293	80
veg_onion_fat_other	0%	99%	1%	1%	99%	1785	20	9293	77.5	856	1.25	855	1.25
veg_onion_lard	0%	99%	1%	1%	99%	1785	20	9293	77.5	858	2.5
veg_onion_marg_hard	0%	99%	1%	99%	1%	1785	20	9293	77.5	860	2.5
veg_onion_marg_poly_chol	0%	99%	1%	90%	10%	1785	20	9293	77.5	2849	0.84	3848	0.83	3243	0.83
veg_onion_marg_poly_dunno	0%	99%	1%	90%	10%	1785	20	9293	77.5	10049	1.25	10043	0.625	10044	0.63
veg_onion_marg_poly_fat	0%	99%	1%	90%	10%	1785	20	9293	77.5	10043	1.25	10044	1.25
veg_onion_marg_poly_lowfat	0%	99%	1%	90%	10%	1785	20	9293	77.5	10049	2.5
veg_onion_marg_poly_vlowfat	0%	99%	1%	90%	10%	1785	20	9293	77.5	10049	2.5
veg_onion_marg_soya_chol	0%	99%	1%	99%	1%	1785	20	9293	77.5	3848	0.42	3243	0.41	2849	0.42	10786	1.25
veg_onion_marg_soya_dunno	0%	99%	1%	100%	0%	1785	20	9293	77.5	10980	1.25	10786	1.25
veg_onion_marg_soya_fat	0%	99%	1%	100%	0%	1785	20	9293	77.5	10786	2.5
veg_onion_marg_soya_lowfat	0%	99%	1%	100%	0%	1785	20	9293	77.5	10980	2.5
veg_onion_marg_soya_vlowfat	0%	99%	1%	100%	0%	1785	20	9293	77.5	10980	2.5
veg_onion_oil_olive	0%	100%	0%	100%	0%	1785	20	9293	77.5	874	2.5
veg_onion_oil_other	0%	100%	0%	100%	0%	1785	20	9293	77.5	870	0.75	870	0.5	10963	0.63	871	0.31	5000	0.313
veg_onion_oil_rapeseed	0%	100%	0%	100%	0%	1785	20	9293	77.5	7990	2.5
veg_onion_oil_sunflower	0%	100%	0%	100%	0%	1785	20	9293	77.5	873	2.5
veg_onion_oil_veg	0%	100%	0%	100%	0%	1785	20	9293	77.5	872	1.25	871	1.25
veg_onion_spread_dairy_chol	0%	99%	1%	99%	1%	1785	20	9293	77.5	3848	2.5
veg_onion_spread_dairy_dunno	0%	99%	1%	99%	1%	1785	20	9293	77.5	10047	1.25	7775	1.25
veg_onion_spread_dairy_fat	0%	99%	1%	99%	1%	1785	20	9293	77.5	7775	2.5
veg_onion_spread_dairy_lowfat	0%	99%	1%	99%	1%	1785	20	9293	77.5	10047	2.5
veg_onion_spread_dairy_vlowfat	0%	99%	1%	99%	1%	1785	20	9293	77.5	10047	2.5
veg_onion_spread_dunno_chol	0%	99%	1%	99%	1%	1785	20	9293	77.5	3848	0.84	3243	0.83	2849	0.83
veg_onion_spread_dunno_dunno	0%	99%	1%	99%	1%	1785	20	9293	77.5	10047	0.625	10049	0.625	10043	0.63	7775	0.63
veg_onion_spread_dunno_fat	0%	99%	1%	99%	1%	1785	20	9293	77.5	10043	1.25	7775	1.25
veg_onion_spread_dunno_lowfat	0%	99%	1%	99%	1%	1785	20	9293	77.5	10047	1.25	10049	1.25
veg_onion_spread_dunno_vlowfat	0%	99%	1%	99%	1%	1785	20	9293	77.5	10047	1.25	10049	1.25
veg_onion_spread_olive_chol	0%	99%	1%	99%	1%	1785	20	9293	77.5	3364	1.25	10053	1.25
veg_onion_spread_olive_dunno	0%	99%	1%	99%	1%	1785	20	9293	77.5	10048	1.25	10042	0.625	10131	0.63
veg_onion_spread_olive_fat	0%	99%	1%	99%	1%	1785	20	9293	77.5	10042	1.875	10131	0.625
veg_onion_spread_olive_lowfat	0%	99%	1%	99%	1%	1785	20	9293	77.5	10048	2.5
veg_onion_spread_olive_vlowfat	0%	99%	1%	99%	1%	1785	20	9293	77.5	10048	2.5
veg_other_butter_dunno	0%	99%	1%	10%	90%	8478	5	1655	42.5	1908	5	1724	5	8471	34.5	1909	5	9407	0.75	10039	0.75	851	0.75	852	0.75
veg_other_butter_fat	0%	99%	1%	1%	99%	8478	5	1655	42.5	1908	5	1724	5	8471	34.5	1909	5	851	1.5	852	1.5
veg_other_butter_lowfat	0%	99%	1%	40%	60%	8478	5	1655	42.5	1908	5	1724	5	8471	34.5	1909	5	10140	3
veg_other_butter_spread_fat	0%	99%	1%	30%	70%	8478	5	1655	42.5	1908	5	1724	5	8471	34.5	1909	5	3891	1.5	10894	1.5
veg_other_butter_spread_lowfat	0%	99%	1%	40%	60%	8478	5	1655	42.5	1908	5	1724	5	8471	34.5	1909	5	9407	1.5	10039	1.5
veg_other_fat_dunno	0%	99%	1%	100%	0%	8478	5	1655	42.5	1908	5	1724	5	8471	34.5	1909	5	875	3
veg_other_fat_none	0%	100%	0%	100%	0%	8478	5	1655	44	1908	5	1724	5	8471	36	1909	5
veg_other_fat_other	0%	99%	1%	1%	99%	8478	5	1655	42.5	1908	5	1724	5	8471	34.5	1909	5	856	1.5	855	1.5
veg_other_lard	0%	99%	1%	1%	99%	8478	5	1655	42.5	1908	5	1724	5	8471	34.5	1909	5	858	3
veg_other_marg_hard	0%	99%	1%	99%	1%	8478	5	1655	42.5	1908	5	1724	5	8471	34.5	1909	5	860	3
veg_other_marg_poly_chol	0%	99%	1%	90%	10%	8478	5	1655	42.5	1908	5	1724	5	8471	34.5	1909	5	2849	1	3848	1	3243	1
veg_other_marg_poly_dunno	0%	99%	1%	90%	10%	8478	5	1655	42.5	1908	5	1724	5	8471	34.5	1909	5	10049	1.5	10043	0.75	10044	0.75
veg_other_marg_poly_fat	0%	99%	1%	90%	10%	8478	5	1655	42.5	1908	5	1724	5	8471	34.5	1909	5	10043	1.5	10044	1.5
veg_other_marg_poly_lowfat	0%	99%	1%	90%	10%	8478	5	1655	42.5	1908	5	1724	5	8471	34.5	1909	5	10049	3

veg_other_marg_poly_vlowfat	0%	99%	1%	90%	10%	8478	5	1655	42.5	1908	5	1724	5	8471	34.5	1909	5	10049	3								
veg_other_marg_soya_chol	0%	99%	1%	99%	1%	8478	5	1655	42.5	1908	5	1724	5	8471	34.5	1909	5	3848	0.5	3243	0.5	2849	0.5	10786	1.5		
veg_other_marg_soya_dunno	0%	99%	1%	100%	0%	8478	5	1655	42.5	1908	5	1724	5	8471	34.5	1909	5	10980	1.5	10786	1.5						
veg_other_marg_soya_fat	0%	99%	1%	100%	0%	8478	5	1655	42.5	1908	5	1724	5	8471	34.5	1909	5	10786	3								
veg_other_marg_soya_lowfat	0%	99%	1%	100%	0%	8478	5	1655	42.5	1908	5	1724	5	8471	34.5	1909	5	10980	3								
veg_other_marg_soya_vlowfat	0%	99%	1%	100%	0%	8478	5	1655	42.5	1908	5	1724	5	8471	34.5	1909	5	10980	3								
veg_other_oil_olive	0%	100%	0%	100%	0%	8478	5	1655	42.5	1908	5	1724	5	8471	34.5	1909	5	874	3								
veg_other_oil_other	0%	100%	0%	100%	0%	8478	5	1655	42.5	1908	5	1724	5	8471	34.5	1909	5	870	0.9	870	0.6	10963	0.75	871	0.375	5000	0.375
veg_other_oil_rapeseed	0%	100%	0%	100%	0%	8478	5	1655	42.5	1908	5	1724	5	8471	34.5	1909	5	7990	3								
veg_other_oil_sunflower	0%	100%	0%	100%	0%	8478	5	1655	42.5	1908	5	1724	5	8471	34.5	1909	5	873	3								
veg_other_oil_veg	0%	100%	0%	100%	0%	8478	5	1655	42.5	1908	5	1724	5	8471	34.5	1909	5	872	1.5	871	1.5						
veg_other_spread_dairy_chol	0%	99%	1%	99%	1%	8478	5	1655	42.5	1908	5	1724	5	8471	34.5	1909	5	3848	3								
veg_other_spread_dairy_dunno	0%	99%	1%	99%	1%	8478	5	1655	42.5	1908	5	1724	5	8471	34.5	1909	5	10047	1.5	7775	1.5						
veg_other_spread_dairy_fat	0%	99%	1%	99%	1%	8478	5	1655	42.5	1908	5	1724	5	8471	34.5	1909	5	7775	3								
veg_other_spread_dairy_lowfat	0%	99%	1%	99%	1%	8478	5	1655	42.5	1908	5	1724	5	8471	34.5	1909	5	10047	3								
veg_other_spread_dairy_vlowfat	0%	99%	1%	99%	1%	8478	5	1655	42.5	1908	5	1724	5	8471	34.5	1909	5	10047	3								
veg_other_spread_dunno_chol	0%	99%	1%	99%	1%	8478	5	1655	42.5	1908	5	1724	5	8471	34.5	1909	5	3848	1	3243	1	2849	1				
veg_other_spread_dunno_dunno	0%	99%	1%	99%	1%	8478	5	1655	42.5	1908	5	1724	5	8471	34.5	1909	5	10047	0.75	10049	0.75	10043	0.75	7775	0.75		
veg_other_spread_dunno_fat	0%	99%	1%	99%	1%	8478	5	1655	42.5	1908	5	1724	5	8471	34.5	1909	5	10043	1.5	7775	1.5						
veg_other_spread_dunno_lowfat	0%	99%	1%	99%	1%	8478	5	1655	42.5	1908	5	1724	5	8471	34.5	1909	5	10047	1.5	10049	1.5						
veg_other_spread_dunno_vlowfat	0%	99%	1%	99%	1%	8478	5	1655	42.5	1908	5	1724	5	8471	34.5	1909	5	10047	1.5	10049	1.5						
veg_other_spread_olive_chol	0%	99%	1%	99%	1%	8478	5	1655	42.5	1908	5	1724	5	8471	34.5	1909	5	3364	1.5	10053	1.5						
veg_other_spread_olive_dunno	0%	99%	1%	99%	1%	8478	5	1655	42.5	1908	5	1724	5	8471	34.5	1909	5	10048	1.5	10042	0.75	10131	0.75				
veg_other_spread_olive_fat	0%	99%	1%	99%	1%	8478	5	1655	42.5	1908	5	1724	5	8471	34.5	1909	5	10042	2.25	10131	0.75						
veg_other_spread_olive_lowfat	0%	99%	1%	99%	1%	8478	5	1655	42.5	1908	5	1724	5	8471	34.5	1909	5	10048	3								
veg_other_spread_olive_vlowfat	0%	99%	1%	99%	1%	8478	5	1655	42.5	1908	5	1724	5	8471	34.5	1909	5	10048	3								
veg_parsnip_butter_dunno	0%	99%	1%	10%	90%	1801	97.5	9407	0.63	10039	0.625	851	0.625	852	0.63												
veg_parsnip_butter_fat	0%	99%	1%	1%	99%	1801	97.5	851	1.25	852	1.25																
veg_parsnip_butter_lowfat	0%	99%	1%	40%	60%	1801	97.5	10140	2.5																		
veg_parsnip_butter_spread_fat	0%	99%	1%	30%	70%	1801	97.5	9407	1.25	10039	1.25																
veg_parsnip_butter_spread_lowfat	0%	99%	1%	40%	60%	1801	97.5	10894	1.25	3891	1.25																
veg_parsnip_fat_dunno	0%	99%	1%	100%	0%	1801	97.5	875	2.5																		
veg_parsnip_fat_none	0%	100%	0%	100%	0%	1801	100																				
veg_parsnip_fat_other	0%	99%	1%	1%	99%	1801	97.5	856	1.25	855	1.25																
veg_parsnip_lard	0%	99%	1%	1%	99%	1801	97.5	858	2.5																		
veg_parsnip_marg_hard	0%	99%	1%	99%	1%	1801	97.5	860	2.5																		
veg_parsnip_marg_poly_chol	0%	99%	1%	90%	10%	1801	97.5	2849	0.84	3848	0.83	3243	0.83														
veg_parsnip_marg_poly_dunno	0%	99%	1%	90%	10%	1801	97.5	10049	1.25	10043	0.625	10044	0.625														
veg_parsnip_marg_poly_fat	0%	99%	1%	90%	10%	1801	97.5	10043	1.25	10044	1.25																
veg_parsnip_marg_poly_lowfat	0%	99%	1%	90%	10%	1801	97.5	10049	2.5																		
veg_parsnip_marg_poly_vlowfat	0%	99%	1%	90%	10%	1801	97.5	10049	2.5																		
veg_parsnip_marg_soya_chol	0%	99%	1%	99%	1%	1801	97.5	3848	0.42	3243	0.41	2849	0.42	10786	1.25												
veg_parsnip_marg_soya_dunno	0%	99%	1%	100%	0%	1801	97.5	10980	1.25	10786	1.25																
veg_parsnip_marg_soya_fat	0%	99%	1%	100%	0%	1801	97.5	10786	2.5																		
veg_parsnip_marg_soya_lowfat	0%	99%	1%	100%	0%	1801	97.5	10980	2.5																		
veg_parsnip_marg_soya_vlowfat	0%	99%	1%	100%	0%	1801	97.5	10980	2.5																		
veg_parsnip_oil_olive	0%	100%	0%	100%	0%	1801	97.5	874	2.5																		
veg_parsnip_oil_other	0%	100%	0%	100%	0%	1801	97.5	870	0.75	870	0.5	10963	0.625	871	0.31	5000	0.31										
veg_parsnip_oil_rapeseed	0%	100%	0%	100%	0%	1801	97.5	7990	2.5																		
veg_parsnip_oil_sunflower	0%	100%	0%	100%	0%	1801	97.5	873	2.5																		
veg_parsnip_oil_veg	0%	100%	0%	100%	0%	1801	97.5	872	1.25	871	1.25																
veg_parsnip_spread_dairy_chol	0%	99%	1%	99%	1%	1801	97.5	3848	2.5																		
veg_parsnip_spread_dairy_dunno	0%	99%	1%	99%	1%	1801	97.5	10047	1.25	7775	1.25																
veg_parsnip_spread_dairy_fat	0%	99%	1%	99%	1%	1801	97.5	7775	2.5																		
veg_parsnip_spread_dairy_lowfat	0%	99%	1%	99%	1%	1801	97.5	10047	2.5																		
veg_parsnip_spread_dairy_vlowfat	0%	99%	1%	99%	1%	1801	97.5	10047	2.5																		
veg_parsnip_spread_dunno_chol	0%	99%	1%	99%	1%	1801	97.5	3848	0.84	3243	0.83	2849	0.83														
veg_parsnip_spread_dunno_dunno	0%	99%	1%	99%	1%	1801	97.5	10047	0.63	10049	0.625	10043	0.625	7775	0.63												
veg_parsnip_spread_dunno_fat	0%	99%	1%	99%	1%	1801	97.5	10043	1.25	7775	1.25																
veg_parsnip_spread_dunno_lowfat	0%	99%	1%	99%	1%	1801	97.5	10047	1.25	10049	1.25																
veg_parsnip_spread_dunno_vlowfat	0%	99%	1%	99%	1%	1801	97.5	10047	1.25	10049	1.25																
veg_parsnip_spread_olive_chol	0%	99%	1%	99%	1%	1801	97.5	3364	1.25	10053	1.25																
veg_parsnip_spread_olive_dunno	0%	99%	1%	99%	1%	1801	97.5	10048	1.25	10042	0.625	10131	0.625														
veg_parsnip_spread_olive_fat	0%	99%	1%	99%	1%	1801	97.5	10042	1.88	10131	0.625																
veg_parsnip_spread_olive_lowfat	0%	99%	1%	99%	1%	1801	97.5	10048	2.5																		
veg_parsnip_spread_olive_vlowfat	0%	99%	1%	99%	1%	1801	97.5	10048	2.5																		
veg_peas	0%	100%	0%	100%	0%	11105	90	10796	10																		
veg_pepper_bell_butter_dunno	0%	99%	1%	10%	90%	1824	19.5	7857	19.5	11102	19.5	7988	19.5	7989	19.5	9407	0.63	10039	0.625	851	0.63	852	0.63				
veg_pepper_bell_butter_fat	0%	99%	1%	1%	99%	1824	19.5	7857	19.5	11102	19.5	7988	19.5	7989	19.5	851	1.25	852	1.25								
veg_pepper_bell_butter_lowfat	0%	99%	1%	40%	60%	1824	19.5	7857	19.5	11102	19.5	7988	19.5	7989	19.5	10140	2.5										
veg_pepper_bell_butter_spread_fat	0%	99%	1%	30%	70%	1824	19.5	7857	19.5	11102	19.5	7988	19.5	7989	19.5	9407	1.25	10039	1.25								
veg_pepper_bell_butter_spread_lowfat	0%	99%	1%	40%	60%	1824	19.5	7857	19.5	11102	19.5	7988	19.5	7989	19.5	10894	1.25	3891	1.25								
veg_pepper_bell_fat_dunno	0%	99%	1%	100%	0%	1824	19.5	7857	19.5	11102	19.5	7988	19.5	7989	19.5	875	2.5										
veg_pepper_bell_fat_none	0%	100%	0%	100%	0%	1824	20	7857	20	11102	20	7988	20	7989	20												
veg_pepper_bell_fat_other	0%	99%	1%	1%	99%	1824	19.5	7857	19.5	11102	19.5	7988	19.5	7989	19.5	856	1.25	855	1.25								
veg_pepper_bell_lard	0%	99%	1%	1%	99%	1824	19.5	7857	19.5	11102	19.5	7988	19.5	7989	19.5	858	2.5										
veg_pepper_bell_marg_hard	0%	99%	1%	99%	1%	1824	19.5	7857	19.5	11102	19.5	7988	19.5	7989	19.5	860	2.5										
veg_pepper_bell_marg_poly_chol	0%	99%	1%	90%	10%	1824	19.5	7857	19.5	11102	19.5	7988	19.5	7989	19.5	2849	0.84	3848	0.83	3243	0.83						
veg_pepper_bell_marg_poly_dunno	0%	99%	1%	90%	10%	1824	19.5	7857	19.5	11102	19.5	7988	19.5	7989	19.5	10049	1.25	10043	0.625	10044	0.63						
veg_pepper_bell_marg_poly_fat	0%	99%	1%	90%	10%	1824	19.5	7857	19.5	11102	19.5	7988	19.5	7989	19.5	10043	1.25	10044	1.25								
veg_pepper_bell_marg_poly_lowfat	0%	99%	1%	90%	10%	1824	19.5	7857	19.5	11102	19.5	7988	19.5	7989	19.5	10049	2.5										
veg_pepper_bell_marg_poly_vlowfat	0%	99%	1%	90%	10%	1824	19.5	7857	19.5	11102	19.5	7988	19.5	7989	19.5	10049	2.5										
veg_pepper_bell_marg_soya_chol	0%	99%	1%	99%	1%	1824	19.5	7857	19.5	11102	19.5	7988	19.5	7989	19.5	3848	0.42	3243	0.41	2849	0.42	10786	1.25				
veg_pepper_bell_marg_soya_dunno	0%	99%	1%	100%	0%	1824	19.5	7857	19.5	11102	19.5	7988	19.5	7989	19.5	10980	1.25	10786	1.25								
veg_pepper_bell_marg_soya_fat	0%	99%	1%	100%	0%	1824	19.5	7857	19.5	11102	19.5	7988	19.5	7989	19.5	10786	2.5										
veg_pepper_bell_marg_soya_lowfat	0%	99%	1%	100%	0%	1824	19.5	7857	19.5	11102	19.5	7988	19.5	7989	19.5	10980	2.5										
veg_pepper_bell_marg_soya_vlowfat	0%	99%	1%	100%	0%	1824	19.5	7857	19.5	11102	19.5	7988	19.5	7989	19.5	10980	2.5										
veg_pepper_bell_oil_olive	0%	100%	0%	100%	0%	1824	19.5	7857	19.5	11102	19.5	7988	19.5	7989	19.5	874	2.5										
veg_pepper_bell_oil_other	0%	100%	0%	100%	0%	1824	19.5	7857	19.5	11102	19.5	7988	19.5	7989	19.5	870	0.75	870	0.5	10963	0.63	871	0.31	5000	0.313		
veg_pepper_bell_oil_rapeseed	0%	100%	0%	100%	0%	1824	19.5	7857	19.5	11102	19.5	7988	19.5	7989	19.5	7990	2.5										
veg_pepper_bell_oil_sunflower	0%	100%	0%	100%	0%	1824	19.5	7857	19.5	11102	19.5	7988	19.5	7989	19.5	873	2.5										
veg_pepper_bell_oil_veg	0%	100%	0%	100%	0%	1824	19.5	7857	19.5	11102	19.5	7988	19.5	7989	19.5	872	1.25	871	1.25								
veg_pepper_bell_spread_dairy_chol	0%	99%	1%	99%	1%	1824	19.5	7857	19.5	11102	19.5	7988	19.5	7989	19.5	3848	2.5										
veg_pepper_bell_spread_dairy_dunno	0%	99%	1%	99%	1%	1824	19.5	7857	19.5	11102	19.5	7988	19.5	7989	19.5	10047	1.25	7775	1.25								
veg_pepper_bell_spread_dairy_fat	0%	99%	1%	99%	1%	1824	19.5	7857	19.5	11102	19.5	7988	19.5	7989	19.5	7775	2.5										
veg_pepper_bell_spread_dairy_lowfat	0%	99%	1%	99%	1%	1824	19.5	7857	19.5	11102	19.5	7988	19.5	7989	19.5	10047	2.5										
veg_pepper_bell_spread_dairy_vlowfat	0%	99%	1%	99%	1%	1824	19.5	7857	19.5	11102	19.5	7988	19.5	7989	19.5	10047	2.5										
veg_pepper_bell_spread_dunno_chol	0%	99%	1%	99%	1%	1824	19.5	7857	19.5	11102	19.5	7988	19.5	7989	19.5	3848	0.84	3243	0.83	2849	0.83						
veg_pepper_bell_spread_dunno_dunno	0%	99%	1%	99%	1%	1824	19.5	7857	19.5	11102	19.5	7988	19.5	7989	19.5	10047	0.63	10049	0.625	10043	0.63	7775	0.63				
veg_pepper_bell_spread_dunno_fat	0%	99%	1%	99%	1%	1824	19.5	7857	19.5	11102	19.5	7988	19.5	7989	19.5	10043	1.25	7775	1.25								
veg_pepper_bell_spread_dunno_lowfat	0%	99%	1%	99%	1%	1824	19.5	7857	19.5	11102	19.5	7988	19.5	7989	19.5	10047	1.25	10049	1.25								
veg_pepper_bell_spread_dunno_vlowfat	0%	99%	1%	99%	1%	1824	19.5	7857	19.5	11102	19.5	7988	19.5	7989	19.5	10047	1.25	10049	1.25								
veg_pepper_bell_spread_olive_chol	0%	99%	1%	99%	1%	1824	19.5	7857	19.5	11102	19.5	7988	19.5	7989	19.5	3364	1.25	10053	1.25								
veg_pepper_bell_spread_olive_dunno	0%	99%	1%	99%	1%	1824	19.5	7857	19.5	11102	19.5	7988	19.5	7989	19.5	10048	1.25	10042	0.625	10131	0.63						
veg_pepper_bell_spread_olive_fat	0%	99%	1%	99%	1%	1824	19.5	7857	19.5	11102	19.5	7988	19.5	7989	19.5	10042	1.88	10131	0.625								
veg_pepper_bell_spread_olive_lowfat	0%	99%	1%	99%	1%	1824	19.5	7857	19.5	11102	19.5	7988	19.5	7989	19.5	10048	2.5										
veg_pepper_bell_spread_olive_vlowfat	0%	99%	1%	99%	1%	1824	19.5	7857	19.5	11102	19.5	7988	19.5	7989	19.5	10048	2.5										
veg_pulses	0%	100%	0%	100%	0%	1758	25	10788	12.5	10790	25	1669	25	10787	12.5												
veg_saladmayo	59%	20%	80%	50%	50%	8282	33.4	8079	33.3	8113	33.3																
veg_sidesalad	0%	100%	0%	100%	0%	1762	25	11103	50	1740	25																
veg_spinach	0%	100%	0%	100%	0%	1915	45	1916	45	11101	5	11100	5														
veg_sprouts	0%	100%	0%	100%	0%	1696	80	1698	20																		
veg_sweetcorn	0%	100%	0%	100%	0%	1922	50	10794	25	10795	25																
veg_sweetpot	0%	100%	0%	100%	0%	10553	50	1930	50																		
veg_tomato_fresh	0%	100%	0%	100%	0%	11103	50	11104	50																		
veg_tomato_tinned	0%	100%	0%	100%	0%	1939	50	1935	25	1932	25																
veg_turnip	0%	100%	0%	100%	0%	1941	50	1921	50																		
veg_watercress	0%	100%	0%	100%	0%	1947	100																				
vegalt_burger	0%	100%	0%	100%	0%	9279	50	4203	50																		
vegalt_other	0%	100%	0%	100%	0%	8587	75	8102	25																		
vegalt_quorn	0%	100%	0%	100%	0%	7189	33.4	5677	33.3	8108	33.3																
vegalt_tofu	0%	100%	0%	100%	0%	1686	80	1371	10	5106	10																
yeast_extract	0%	100%	0%	100%	0%	2431	100																				
yogurt_fullfat	18%	0%	100%	0%	100%	702	40	701	40	7741	20																
yogurt_lowfat	28%	0%	100%	0%	100%	705	25	704	50	2701	25																
yorkshirepud	0%	30%	70%	10%	90%	10711	25	10709	25	10712	50																
