# Supplementary material for: Description of the updated nutrition calculation of the Oxford WebQ questionnaire and comparison with the previous version among 207,144 participants in UK Biobank
Source: Eur J Nutr. 2021 May 6;60(7):4019–30. doi: 10.1007/s00394-021-02558-4 (PMC8437868; doi:10.1007/s00394-021-02558-4)
Supplement: Supplementary file 3 — Supplementary file3 (PDF 132 KB) [file 394_2021_2558_MOESM3_ESM.pdf]

Supplementary table 3. Nutrient calculation in the previous version (McCance and Widdowson).

	Food codes from McCance and Widdowson and the % used from each food code									
**Item**	**Code1**	**% from code 1**	**Code2**	**% from code 2**	**Code3**	**% from code 3**	**Code4**	**% from code 4**	**Code5**	**% from code 5**
add_salt	17367	100								
alcohol_beercider	17211	35	17215	35	17222	15	17224	15		
alcohol_other	17242	33.4	17245	33.3	17244	33.3				
alcohol_spirits	17246	50	17247	50						
alcohol_wine_fort	17235	16.7	17236	16.7	17237	16.6	17234	50		
alcohol_wine_red_large	17228	100								
alcohol_wine_red_med	17228	100								
alcohol_wine_red_small	17228	100								
alcohol_wine_rose_large	17229	100								
alcohol_wine_rose_med	17229	100								
alcohol_wine_rose_small	17229	100								
alcohol_wine_white_large	17230	25	17231	25	17232	25	17233	25		
alcohol_wine_white_med	17230	25	17231	25	17232	25	17233	25		
alcohol_wine_white_small	17230	25	17231	25	17232	25	17233	25		
biscuit_choc	11508	50	11512	50						
biscuit_choccov	11507	50	11506	50						
biscuit_sweet	11513	33.4	11523	33.3	11514	33.3				
bread_baguette_mixed	11461	33.4	00033	33.3	11472	33.3				
bread_baguette_other	00040	50	00046	50						
bread_baguette_seeded	14844	50	14845	50						
bread_baguette_spread_butter_dunno_med	17486	100								
bread_baguette_spread_butter_dunno_thick	17486	100								
bread_baguette_spread_butter_dunno_thin	17486	100								
bread_baguette_spread_butter_fat_med	17485	100								
bread_baguette_spread_butter_fat_thick	17485	100								
bread_baguette_spread_butter_fat_thin	17485	100								
bread_baguette_spread_butter_lowfat_med	17485	50	17017	50						
bread_baguette_spread_butter_lowfat_thick	17485	50	17017	50						
bread_baguette_spread_butter_lowfat_thin	17485	50	17017	50						
bread_baguette_spread_butter_spread_fat_med	17486	100								
bread_baguette_spread_butter_spread_fat_thick	17486	100								
bread_baguette_spread_butter_spread_fat_thin	17486	100								
bread_baguette_spread_butter_spread_lowfat_med	17486	50	17017	50						
bread_baguette_spread_butter_spread_lowfat_thick	17486	50	17017	50						
bread_baguette_spread_butter_spread_lowfat_thin	17486	50	17017	50						
bread_baguette_spread_dairy_chol_med	17017	100								
bread_baguette_spread_dairy_chol_thick	17017	100								
bread_baguette_spread_dairy_chol_thin	17017	100								
bread_baguette_spread_dairy_dunno_med	17017	30	12258	70						
bread_baguette_spread_dairy_dunno_thick	17017	30	12258	70						
bread_baguette_spread_dairy_dunno_thin	17017	30	12258	70						
bread_baguette_spread_dairy_fat_med	12258	100								
bread_baguette_spread_dairy_fat_thick	12258	100								
bread_baguette_spread_dairy_fat_thin	12258	100								
bread_baguette_spread_dairy_lowfat_med	17017	100								
bread_baguette_spread_dairy_lowfat_thick	17017	100								
bread_baguette_spread_dairy_lowfat_thin	17017	100								
bread_baguette_spread_dairy_vlowfat_med	17028	100								
bread_baguette_spread_dairy_vlowfat_thick	17028	100								
bread_baguette_spread_dairy_vlowfat_thin	17028	100								
bread_baguette_spread_dunno_chol_med	17552	50	17027	50						
bread_baguette_spread_dunno_chol_thick	17552	50	17027	50						
bread_baguette_spread_dunno_chol_thin	17552	50	17027	50						
bread_baguette_spread_dunno_dunno_med	17552	25	17027	25	17025	25	17024	25		
bread_baguette_spread_dunno_dunno_thick	17552	25	17027	25	17025	25	17024	25		
bread_baguette_spread_dunno_dunno_thin	17552	25	17027	25	17025	25	17024	25		
bread_baguette_spread_dunno_fat_med	12258	33.3	17025	33.3	17024	33.4				
bread_baguette_spread_dunno_fat_thick	12258	33.3	17025	33.3	17024	33.4				
bread_baguette_spread_dunno_fat_thin	12258	33.3	17025	33.3	17024	33.4				
bread_baguette_spread_dunno_lowfat_med	17017	33.3	17552	33.3	17027	33.4				
bread_baguette_spread_dunno_lowfat_thick	17017	33.3	17552	33.3	17027	33.4				
bread_baguette_spread_dunno_lowfat_thin	17017	33.3	17552	33.3	17027	33.4				
bread_baguette_spread_dunno_vlowfat_med	17028	50	17029	50						
bread_baguette_spread_dunno_vlowfat_thick	17028	50	17029	50						
bread_baguette_spread_dunno_vlowfat_thin	17028	50	17029	50						
bread_baguette_spread_hardmarg_med	17018	50	17539	50						
bread_baguette_spread_hardmarg_thick	17018	50	17539	50						
bread_baguette_spread_hardmarg_thin	17018	50	17539	50						
bread_baguette_spread_olive_chol_med	17552	50	17025	50						
bread_baguette_spread_olive_chol_thick	17552	50	17025	50						
bread_baguette_spread_olive_chol_thin	17552	50	17025	50						
bread_baguette_spread_olive_dunno_med	17552	50	17025	50						
bread_baguette_spread_olive_dunno_thick	17552	50	17025	50						
bread_baguette_spread_olive_dunno_thin	17552	30	17025	70						
bread_baguette_spread_olive_fat_med	17025	100								
bread_baguette_spread_olive_fat_thick	17025	100								
bread_baguette_spread_olive_fat_thin	17025	100								
bread_baguette_spread_olive_lowfat_med	17552	100								
bread_baguette_spread_olive_lowfat_thick	17552	100								
bread_baguette_spread_olive_lowfat_thin	17552	100								
bread_baguette_spread_olive_vlowfat_med	17028	100								
bread_baguette_spread_olive_vlowfat_thick	17028	100								
bread_baguette_spread_olive_vlowfat_thin	17028	100								
bread_baguette_spread_other_med	17007	50	17487	50						
bread_baguette_spread_other_thick	17007	50	17487	50						
bread_baguette_spread_other_thin	17007	50	17487	50						
bread_baguette_spread_polymarg_chol_med	17027	100								
bread_baguette_spread_polymarg_chol_thick	17027	100								
bread_baguette_spread_polymarg_chol_thin	17027	100								
bread_baguette_spread_polymarg_dunno_med	17027	30	17024	70						
bread_baguette_spread_polymarg_dunno_thick	17027	30	17024	70						
bread_baguette_spread_polymarg_dunno_thin	17027	30	17024	70						
bread_baguette_spread_polymarg_fat_med	17024	100								
bread_baguette_spread_polymarg_fat_thick	17024	100								
bread_baguette_spread_polymarg_fat_thin	17024	100								
bread_baguette_spread_polymarg_lowfat_med	17027	100								
bread_baguette_spread_polymarg_lowfat_thick	17027	100								
bread_baguette_spread_polymarg_lowfat_thin	17027	100								
bread_baguette_spread_polymarg_vlowfat_med	17029	100								
bread_baguette_spread_polymarg_vlowfat_thick	17029	100								
bread_baguette_spread_polymarg_vlowfat_thin	17029	100								
bread_baguette_spread_soya_chol_med	17027	100								
bread_baguette_spread_soya_chol_thick	17027	100								
bread_baguette_spread_soya_chol_thin	17027	100								
bread_baguette_spread_soya_dunno_med	17027	30	17024	70						
bread_baguette_spread_soya_dunno_thick	17027	30	17024	70						
bread_baguette_spread_soya_dunno_thin	17027	30	17024	70						
bread_baguette_spread_soya_fat_med	17024	100								
bread_baguette_spread_soya_fat_thick	17024	100								

Supplementary table 3. Nutrient calculation in the previous version (McCance and Widdowson).

	Food codes from McCance and Widdowson and the % used from each food code									
**Item**	**Code1**	**% from code 1**	**Code2**	**% from code 2**	**Code3**	**% from code 3**	**Code4**	**% from code 4**	**Code5**	**% from code 5**
bread_baguette_spread_soya_fat_thin	17024	100								
bread_baguette_spread_soya_lowfat_med	17027	100								
bread_baguette_spread_soya_lowfat_thick	17027	100								
bread_baguette_spread_soya_lowfat_thin	17027	100								
bread_baguette_spread_soya_vlowfat_med	17029	100								
bread_baguette_spread_soya_vlowfat_thick	17029	100								
bread_baguette_spread_soya_vlowfat_thin	17029	100								
bread_baguette_unanswered	00048	33.4	00056	33.3	00033	33.3				
bread_baguette_white	11471	50	11609	50						
bread_baguette_wholemeal	00056	100								
bread_crisp	11511	33.4	11510	33.3	11572	33.3				
bread_crisp_spread_butter_dunno_med	17486	100								
bread_crisp_spread_butter_dunno_thick	17486	100								
bread_crisp_spread_butter_dunno_thin	17486	100								
bread_crisp_spread_butter_fat_med	17485	100								
bread_crisp_spread_butter_fat_thick	17485	100								
bread_crisp_spread_butter_fat_thin	17485	100								
bread_crisp_spread_butter_lowfat_med	17485	50	17017	50						
bread_crisp_spread_butter_lowfat_thick	17485	50	17017	50						
bread_crisp_spread_butter_lowfat_thin	17485	50	17017	50						
bread_crisp_spread_butter_spread_fat_med	17486	100								
bread_crisp_spread_butter_spread_fat_thick	17486	100								
bread_crisp_spread_butter_spread_fat_thin	17486	100								
bread_crisp_spread_butter_spread_lowfat_med	17486	50	17017	50						
bread_crisp_spread_butter_spread_lowfat_thick	17486	50	17017	50						
bread_crisp_spread_butter_spread_lowfat_thin	17486	50	17017	50						
bread_crisp_spread_dairy_chol_med	17017	100								
bread_crisp_spread_dairy_chol_thick	17017	100								
bread_crisp_spread_dairy_chol_thin	17017	100								
bread_crisp_spread_dairy_dunno_med	17017	30	12258	70						
bread_crisp_spread_dairy_dunno_thick	17017	30	12258	70						
bread_crisp_spread_dairy_dunno_thin	17017	30	12258	70						
bread_crisp_spread_dairy_fat_med	12258	100								
bread_crisp_spread_dairy_fat_thick	12258	100								
bread_crisp_spread_dairy_fat_thin	12258	100								
bread_crisp_spread_dairy_lowfat_med	17017	100								
bread_crisp_spread_dairy_lowfat_thick	17017	100								
bread_crisp_spread_dairy_lowfat_thin	17017	100								
bread_crisp_spread_dairy_vlowfat_med	17028	100								
bread_crisp_spread_dairy_vlowfat_thick	17028	100								
bread_crisp_spread_dairy_vlowfat_thin	17028	100								
bread_crisp_spread_dunno_chol_med	17552	50	17027	50						
bread_crisp_spread_dunno_chol_thick	17552	50	17027	50						
bread_crisp_spread_dunno_chol_thin	17552	50	17027	50						
bread_crisp_spread_dunno_dunno_med	17552	25	17027	25	17025	25	17024	25		
bread_crisp_spread_dunno_dunno_thick	17552	25	17027	25	17025	25	17024	25		
bread_crisp_spread_dunno_dunno_thin	17552	25	17027	25	17025	25	17024	25		
bread_crisp_spread_dunno_fat_med	12258	33.3	17025	33.3	17024	33.4				
bread_crisp_spread_dunno_fat_thick	12258	33.3	17025	33.3	17024	33.4				
bread_crisp_spread_dunno_fat_thin	12258	33.3	17025	33.3	17024	33.4				
bread_crisp_spread_dunno_lowfat_med	17017	33.3	17552	33.3	17027	33.4				
bread_crisp_spread_dunno_lowfat_thick	17017	33.3	17552	33.3	17027	33.4				
bread_crisp_spread_dunno_lowfat_thin	17017	33.3	17552	33.3	17027	33.4				
bread_crisp_spread_dunno_vlowfat_med	17028	50	17029	50						
bread_crisp_spread_dunno_vlowfat_thick	17028	50	17029	50						
bread_crisp_spread_dunno_vlowfat_thin	17028	50	17029	50						
bread_crisp_spread_hardmarg_med	17018	50	17539	50						
bread_crisp_spread_hardmarg_thick	17018	50	17539	50						
bread_crisp_spread_hardmarg_thin	17018	50	17539	50						
bread_crisp_spread_olive_chol_med	17552	50	17025	50						
bread_crisp_spread_olive_chol_thick	17552	50	17025	50						
bread_crisp_spread_olive_chol_thin	17552	50	17025	50						
bread_crisp_spread_olive_dunno_med	17552	50	17025	50						
bread_crisp_spread_olive_dunno_thick	17552	50	17025	50						
bread_crisp_spread_olive_dunno_thin	17552	30	17025	70						
bread_crisp_spread_olive_fat_med	17025	100								
bread_crisp_spread_olive_fat_thick	17025	100								
bread_crisp_spread_olive_fat_thin	17025	100								
bread_crisp_spread_olive_lowfat_med	17552	100								
bread_crisp_spread_olive_lowfat_thick	17552	100								
bread_crisp_spread_olive_lowfat_thin	17552	100								
bread_crisp_spread_olive_vlowfat_med	17028	100								
bread_crisp_spread_olive_vlowfat_thick	17028	100								
bread_crisp_spread_olive_vlowfat_thin	17028	100								
bread_crisp_spread_other_med	17007	50	17487	50						
bread_crisp_spread_other_thick	17007	50	17487	50						
bread_crisp_spread_other_thin	17007	50	17487	50						
bread_crisp_spread_polymarg_chol_med	17027	100								
bread_crisp_spread_polymarg_chol_thick	17027	100								
bread_crisp_spread_polymarg_chol_thin	17027	100								
bread_crisp_spread_polymarg_dunno_med	17027	30	17024	70						
bread_crisp_spread_polymarg_dunno_thick	17027	30	17024	70						
bread_crisp_spread_polymarg_dunno_thin	17027	30	17024	70						
bread_crisp_spread_polymarg_fat_med	17024	100								
bread_crisp_spread_polymarg_fat_thick	17024	100								
bread_crisp_spread_polymarg_fat_thin	17024	100								
bread_crisp_spread_polymarg_lowfat_med	17027	100								
bread_crisp_spread_polymarg_lowfat_thick	17027	100								
bread_crisp_spread_polymarg_lowfat_thin	17027	100								
bread_crisp_spread_polymarg_vlowfat_med	17029	100								
bread_crisp_spread_polymarg_vlowfat_thick	17029	100								
bread_crisp_spread_polymarg_vlowfat_thin	17029	100								
bread_crisp_spread_soya_chol_med	17027	100								
bread_crisp_spread_soya_chol_thick	17027	100								
bread_crisp_spread_soya_chol_thin	17027	100								
bread_crisp_spread_soya_dunno_med	17027	30	17024	70						
bread_crisp_spread_soya_dunno_thick	17027	30	17024	70						
bread_crisp_spread_soya_dunno_thin	17027	30	17024	70						
bread_crisp_spread_soya_fat_med	17024	100								
bread_crisp_spread_soya_fat_thick	17024	100								
bread_crisp_spread_soya_fat_thin	17024	100								
bread_crisp_spread_soya_lowfat_med	17027	100								
bread_crisp_spread_soya_lowfat_thick	17027	100								
bread_crisp_spread_soya_lowfat_thin	17027	100								
bread_crisp_spread_soya_vlowfat_med	17029	100								
bread_crisp_spread_soya_vlowfat_thick	17029	100								
bread_crisp_spread_soya_vlowfat_thin	17029	100								
bread_garlic	11460	100								
bread_large_bap_mixed	11461	33.4	00033	33.3	11472	33.3				

Supplementary table 3. Nutrient calculation in the previous version (McCance and Widdowson).

	Food codes from McCance and Widdowson and the % used from each food code									
**Item**	**Code1**	**% from code 1**	**Code2**	**% from code 2**	**Code3**	**% from code 3**	**Code4**	**% from code 4**	**Code5**	**% from code 5**
bread_large_bap_other	00040	50	00046	50						
bread_large_bap_seeded	14844	50	14845	50						
bread_large_bap_spread_butter_dunno_med	17486	100								
bread_large_bap_spread_butter_dunno_thick	17486	100								
bread_large_bap_spread_butter_dunno_thin	17486	100								
bread_large_bap_spread_butter_fat_med	17485	100								
bread_large_bap_spread_butter_fat_thick	17485	100								
bread_large_bap_spread_butter_fat_thin	17485	100								
bread_large_bap_spread_butter_lowfat_med	17485	50	17017	50						
bread_large_bap_spread_butter_lowfat_thick	17485	50	17017	50						
bread_large_bap_spread_butter_lowfat_thin	17485	50	17017	50						
bread_large_bap_spread_butter_spread_fat_med	17486	100								
bread_large_bap_spread_butter_spread_fat_thick	17486	100								
bread_large_bap_spread_butter_spread_fat_thin	17486	100								
bread_large_bap_spread_butter_spread_lowfat_med	17486	50	17017	50						
bread_large_bap_spread_butter_spread_lowfat_thick	17486	50	17017	50						
bread_large_bap_spread_butter_spread_lowfat_thin	17486	50	17017	50						
bread_large_bap_spread_dairy_chol_med	17017	100								
bread_large_bap_spread_dairy_chol_thick	17017	100								
bread_large_bap_spread_dairy_chol_thin	17017	100								
bread_large_bap_spread_dairy_dunno_med	17017	30	12258	70						
bread_large_bap_spread_dairy_dunno_thick	17017	30	12258	70						
bread_large_bap_spread_dairy_dunno_thin	17017	30	12258	70						
bread_large_bap_spread_dairy_fat_med	12258	100								
bread_large_bap_spread_dairy_fat_thick	12258	100								
bread_large_bap_spread_dairy_fat_thin	12258	100								
bread_large_bap_spread_dairy_lowfat_med	17017	100								
bread_large_bap_spread_dairy_lowfat_thick	17017	100								
bread_large_bap_spread_dairy_lowfat_thin	17017	100								
bread_large_bap_spread_dairy_vlowfat_med	17028	100								
bread_large_bap_spread_dairy_vlowfat_thick	17028	100								
bread_large_bap_spread_dairy_vlowfat_thin	17028	100								
bread_large_bap_spread_dunno_chol_med	17552	50	17027	50						
bread_large_bap_spread_dunno_chol_thick	17552	50	17027	50						
bread_large_bap_spread_dunno_chol_thin	17552	50	17027	50						
bread_large_bap_spread_dunno_dunno_med	17552	25	17027	25	17025	25	17024	25		
bread_large_bap_spread_dunno_dunno_thick	17552	25	17027	25	17025	25	17024	25		
bread_large_bap_spread_dunno_dunno_thin	17552	25	17027	25	17025	25	17024	25		
bread_large_bap_spread_dunno_fat_med	12258	33.3	17025	33.3	17024	33.4				
bread_large_bap_spread_dunno_fat_thick	12258	33.3	17025	33.3	17024	33.4				
bread_large_bap_spread_dunno_fat_thin	12258	33.3	17025	33.3	17024	33.4				
bread_large_bap_spread_dunno_lowfat_med	17017	33.3	17552	33.3	17027	33.4				
bread_large_bap_spread_dunno_lowfat_thick	17017	33.3	17552	33.3	17027	33.4				
bread_large_bap_spread_dunno_lowfat_thin	17017	33.3	17552	33.3	17027	33.4				
bread_large_bap_spread_dunno_vlowfat_med	17028	50	17029	50						
bread_large_bap_spread_dunno_vlowfat_thick	17028	50	17029	50						
bread_large_bap_spread_dunno_vlowfat_thin	17028	50	17029	50						
bread_large_bap_spread_hardmarg_med	17018	50	17539	50						
bread_large_bap_spread_hardmarg_thick	17018	50	17539	50						
bread_large_bap_spread_hardmarg_thin	17018	50	17539	50						
bread_large_bap_spread_olive_chol_med	17552	50	17025	50						
bread_large_bap_spread_olive_chol_thick	17552	50	17025	50						
bread_large_bap_spread_olive_chol_thin	17552	50	17025	50						
bread_large_bap_spread_olive_dunno_med	17552	50	17025	50						
bread_large_bap_spread_olive_dunno_thick	17552	50	17025	50						
bread_large_bap_spread_olive_dunno_thin	17552	30	17025	70						
bread_large_bap_spread_olive_fat_med	17025	100								
bread_large_bap_spread_olive_fat_thick	17025	100								
bread_large_bap_spread_olive_fat_thin	17025	100								
bread_large_bap_spread_olive_lowfat_med	17552	100								
bread_large_bap_spread_olive_lowfat_thick	17552	100								
bread_large_bap_spread_olive_lowfat_thin	17552	100								
bread_large_bap_spread_olive_vlowfat_med	17028	100								
bread_large_bap_spread_olive_vlowfat_thick	17028	100								
bread_large_bap_spread_olive_vlowfat_thin	17028	100								
bread_large_bap_spread_other_med	17007	50	17487	50						
bread_large_bap_spread_other_thick	17007	50	17487	50						
bread_large_bap_spread_other_thin	17007	50	17487	50						
bread_large_bap_spread_polymarg_chol_med	17027	100								
bread_large_bap_spread_polymarg_chol_thick	17027	100								
bread_large_bap_spread_polymarg_chol_thin	17027	100								
bread_large_bap_spread_polymarg_dunno_med	17027	30	17024	70						
bread_large_bap_spread_polymarg_dunno_thick	17027	30	17024	70						
bread_large_bap_spread_polymarg_dunno_thin	17027	30	17024	70						
bread_large_bap_spread_polymarg_fat_med	17024	100								
bread_large_bap_spread_polymarg_fat_thick	17024	100								
bread_large_bap_spread_polymarg_fat_thin	17024	100								
bread_large_bap_spread_polymarg_lowfat_med	17027	100								
bread_large_bap_spread_polymarg_lowfat_thick	17027	100								
bread_large_bap_spread_polymarg_lowfat_thin	17027	100								
bread_large_bap_spread_polymarg_vlowfat_med	17029	100								
bread_large_bap_spread_polymarg_vlowfat_thick	17029	100								
bread_large_bap_spread_polymarg_vlowfat_thin	17029	100								
bread_large_bap_spread_soya_chol_med	17027	100								
bread_large_bap_spread_soya_chol_thick	17027	100								
bread_large_bap_spread_soya_chol_thin	17027	100								
bread_large_bap_spread_soya_dunno_med	17027	30	17024	70						
bread_large_bap_spread_soya_dunno_thick	17027	30	17024	70						
bread_large_bap_spread_soya_dunno_thin	17027	30	17024	70						
bread_large_bap_spread_soya_fat_med	17024	100								
bread_large_bap_spread_soya_fat_thick	17024	100								
bread_large_bap_spread_soya_fat_thin	17024	100								
bread_large_bap_spread_soya_lowfat_med	17027	100								
bread_large_bap_spread_soya_lowfat_thick	17027	100								
bread_large_bap_spread_soya_lowfat_thin	17027	100								
bread_large_bap_spread_soya_vlowfat_med	17029	100								
bread_large_bap_spread_soya_vlowfat_thick	17029	100								
bread_large_bap_spread_soya_vlowfat_thin	17029	100								
bread_large_bap_unanswered	00048	33.4	00056	33.3	00033	33.3				
bread_large_bap_white	11465	50	00048	50						
bread_large_bap_wholemeal	00056	100								
bread_naan	11463	100								
bread_other	11093	45	11535	45	17123	10				
bread_other_spread_butter_dunno_med	17486	100								
bread_other_spread_butter_dunno_thick	17486	100								
bread_other_spread_butter_dunno_thin	17486	100								
bread_other_spread_butter_fat_med	17485	100								
bread_other_spread_butter_fat_thick	17485	100								
bread_other_spread_butter_fat_thin	17485	100								

Supplementary table 3. Nutrient calculation in the previous version (McCance and Widdowson).

	Food codes from McCance and Widdowson and the % used from each food code									
**Item**	**Code1**	**% from code 1**	**Code2**	**% from code 2**	**Code3**	**% from code 3**	**Code4**	**% from code 4**	**Code5**	**% from code 5**
bread_other_spread_butter_lowfat_med	17485	50	17017	50						
bread_other_spread_butter_lowfat_thick	17485	50	17017	50						
bread_other_spread_butter_lowfat_thin	17485	50	17017	50						
bread_other_spread_butter_spread_fat_med	17486	100								
bread_other_spread_butter_spread_fat_thick	17486	100								
bread_other_spread_butter_spread_fat_thin	17486	100								
bread_other_spread_butter_spread_lowfat_med	17486	50	17017	50						
bread_other_spread_butter_spread_lowfat_thick	17486	50	17017	50						
bread_other_spread_butter_spread_lowfat_thin	17486	50	17017	50						
bread_other_spread_dairy_chol_med	17017	100								
bread_other_spread_dairy_chol_thick	17017	100								
bread_other_spread_dairy_chol_thin	17017	100								
bread_other_spread_dairy_dunno_med	17017	30	12258	70						
bread_other_spread_dairy_dunno_thick	17017	30	12258	70						
bread_other_spread_dairy_dunno_thin	17017	30	12258	70						
bread_other_spread_dairy_fat_med	12258	100								
bread_other_spread_dairy_fat_thick	12258	100								
bread_other_spread_dairy_fat_thin	12258	100								
bread_other_spread_dairy_lowfat_med	17017	100								
bread_other_spread_dairy_lowfat_thick	17017	100								
bread_other_spread_dairy_lowfat_thin	17017	100								
bread_other_spread_dairy_vlowfat_med	17028	100								
bread_other_spread_dairy_vlowfat_thick	17028	100								
bread_other_spread_dairy_vlowfat_thin	17028	100								
bread_other_spread_dunno_chol_med	17552	50	17027	50						
bread_other_spread_dunno_chol_thick	17552	50	17027	50						
bread_other_spread_dunno_chol_thin	17552	50	17027	50						
bread_other_spread_dunno_dunno_med	17552	25	17027	25	17025	25	17024	25		
bread_other_spread_dunno_dunno_thick	17552	25	17027	25	17025	25	17024	25		
bread_other_spread_dunno_dunno_thin	17552	25	17027	25	17025	25	17024	25		
bread_other_spread_dunno_fat_med	12258	33.3	17025	33.3	17024	33.4				
bread_other_spread_dunno_fat_thick	12258	33.3	17025	33.3	17024	33.4				
bread_other_spread_dunno_fat_thin	12258	33.3	17025	33.3	17024	33.4				
bread_other_spread_dunno_lowfat_med	17017	33.3	17552	33.3	17027	33.4				
bread_other_spread_dunno_lowfat_thick	17017	33.3	17552	33.3	17027	33.4				
bread_other_spread_dunno_lowfat_thin	17017	33.3	17552	33.3	17027	33.4				
bread_other_spread_dunno_vlowfat_med	17028	50	17029	50						
bread_other_spread_dunno_vlowfat_thick	17028	50	17029	50						
bread_other_spread_dunno_vlowfat_thin	17028	50	17029	50						
bread_other_spread_hardmarg_med	17018	50	17539	50						
bread_other_spread_hardmarg_thick	17018	50	17539	50						
bread_other_spread_hardmarg_thin	17018	50	17539	50						
bread_other_spread_olive_chol_med	17552	50	17025	50						
bread_other_spread_olive_chol_thick	17552	50	17025	50						
bread_other_spread_olive_chol_thin	17552	50	17025	50						
bread_other_spread_olive_dunno_med	17552	50	17025	50						
bread_other_spread_olive_dunno_thick	17552	50	17025	50						
bread_other_spread_olive_dunno_thin	17552	30	17025	70						
bread_other_spread_olive_fat_med	17025	100								
bread_other_spread_olive_fat_thick	17025	100								
bread_other_spread_olive_fat_thin	17025	100								
bread_other_spread_olive_lowfat_med	17552	100								
bread_other_spread_olive_lowfat_thick	17552	100								
bread_other_spread_olive_lowfat_thin	17552	100								
bread_other_spread_olive_vlowfat_med	17028	100								
bread_other_spread_olive_vlowfat_thick	17028	100								
bread_other_spread_olive_vlowfat_thin	17028	100								
bread_other_spread_other_med	17007	50	17487	50						
bread_other_spread_other_thick	17007	50	17487	50						
bread_other_spread_other_thin	17007	50	17487	50						
bread_other_spread_polymarg_chol_med	17027	100								
bread_other_spread_polymarg_chol_thick	17027	100								
bread_other_spread_polymarg_chol_thin	17027	100								
bread_other_spread_polymarg_dunno_med	17027	30	17024	70						
bread_other_spread_polymarg_dunno_thick	17027	30	17024	70						
bread_other_spread_polymarg_dunno_thin	17027	30	17024	70						
bread_other_spread_polymarg_fat_med	17024	100								
bread_other_spread_polymarg_fat_thick	17024	100								
bread_other_spread_polymarg_fat_thin	17024	100								
bread_other_spread_polymarg_lowfat_med	17027	100								
bread_other_spread_polymarg_lowfat_thick	17027	100								
bread_other_spread_polymarg_lowfat_thin	17027	100								
bread_other_spread_polymarg_vlowfat_med	17029	100								
bread_other_spread_polymarg_vlowfat_thick	17029	100								
bread_other_spread_polymarg_vlowfat_thin	17029	100								
bread_other_spread_soya_chol_med	17027	100								
bread_other_spread_soya_chol_thick	17027	100								
bread_other_spread_soya_chol_thin	17027	100								
bread_other_spread_soya_dunno_med	17027	30	17024	70						
bread_other_spread_soya_dunno_thick	17027	30	17024	70						
bread_other_spread_soya_dunno_thin	17027	30	17024	70						
bread_other_spread_soya_fat_med	17024	100								
bread_other_spread_soya_fat_thick	17024	100								
bread_other_spread_soya_fat_thin	17024	100								
bread_other_spread_soya_lowfat_med	17027	100								
bread_other_spread_soya_lowfat_thick	17027	100								
bread_other_spread_soya_lowfat_thin	17027	100								
bread_other_spread_soya_vlowfat_med	17029	100								
bread_other_spread_soya_vlowfat_thick	17029	100								
bread_other_spread_soya_vlowfat_thin	17029	100								
bread_roll_mixed	11461	33.4	00033	33.3	11472	33.3				
bread_roll_other	00040	50	00046	50						
bread_roll_seeded	14844	50	14845	50						
bread_roll_spread_butter_dunno_med	17486	100								
bread_roll_spread_butter_dunno_thick	17486	100								
bread_roll_spread_butter_dunno_thin	17486	100								
bread_roll_spread_butter_fat_med	17485	100								
bread_roll_spread_butter_fat_thick	17485	100								
bread_roll_spread_butter_fat_thin	17485	100								
bread_roll_spread_butter_lowfat_med	17485	50	17017	50						
bread_roll_spread_butter_lowfat_thick	17485	50	17017	50						
bread_roll_spread_butter_lowfat_thin	17485	50	17017	50						
bread_roll_spread_butter_spread_fat_med	17486	100								
bread_roll_spread_butter_spread_fat_thick	17486	100								
bread_roll_spread_butter_spread_fat_thin	17486	100								
bread_roll_spread_butter_spread_lowfat_med	17486	50	17017	50						
bread_roll_spread_butter_spread_lowfat_thick	17486	50	17017	50						
bread_roll_spread_butter_spread_lowfat_thin	17486	50	17017	50						
bread_roll_spread_dairy_chol_med	17017	100								

Supplementary table 3. Nutrient calculation in the previous version (McCance and Widdowson).

	Food codes from McCance and Widdowson and the % used from each food code									
Item	Code1	% from code 1	Code2	% from code 2	Code3	% from code 3	Code4	% from code 4	Code5	% from code 5
bread_roll_spread_dairy_chol_thick	17017	100								
bread_roll_spread_dairy_chol_thin	17017	100								
bread_roll_spread_dairy_dunno_med	17017	30	12258	70						
bread_roll_spread_dairy_dunno_thick	17017	30	12258	70						
bread_roll_spread_dairy_dunno_thin	17017	30	12258	70						
bread_roll_spread_dairy_fat_med	12258	100								
bread_roll_spread_dairy_fat_thick	12258	100								
bread_roll_spread_dairy_fat_thin	12258	100								
bread_roll_spread_dairy_lowfat_med	17017	100								
bread_roll_spread_dairy_lowfat_thick	17017	100								
bread_roll_spread_dairy_lowfat_thin	17017	100								
bread_roll_spread_dairy_vlowfat_med	17028	100								
bread_roll_spread_dairy_vlowfat_thick	17028	100								
bread_roll_spread_dairy_vlowfat_thin	17028	100								
bread_roll_spread_dunno_chol_med	17552	50	17027	50						
bread_roll_spread_dunno_chol_thick	17552	50	17027	50						
bread_roll_spread_dunno_chol_thin	17552	50	17027	50						
bread_roll_spread_dunno_dunno_med	17552	25	17027	25	17025	25	17024	25		
bread_roll_spread_dunno_dunno_thick	17552	25	17027	25	17025	25	17024	25		
bread_roll_spread_dunno_dunno_thin	17552	25	17027	25	17025	25	17024	25		
bread_roll_spread_dunno_fat_med	12258	33.3	17025	33.3	17024	33.4				
bread_roll_spread_dunno_fat_thick	12258	33.3	17025	33.3	17024	33.4				
bread_roll_spread_dunno_fat_thin	12258	33.3	17025	33.3	17024	33.4				
bread_roll_spread_dunno_lowfat_med	17017	33.3	17552	33.3	17027	33.4				
bread_roll_spread_dunno_lowfat_thick	17017	33.3	17552	33.3	17027	33.4				
bread_roll_spread_dunno_lowfat_thin	17017	33.3	17552	33.3	17027	33.4				
bread_roll_spread_dunno_vlowfat_med	17028	50	17029	50						
bread_roll_spread_dunno_vlowfat_thick	17028	50	17029	50						
bread_roll_spread_dunno_vlowfat_thin	17028	50	17029	50						
bread_roll_spread_hardmarg_med	17018	50	17539	50						
bread_roll_spread_hardmarg_thick	17018	50	17539	50						
bread_roll_spread_hardmarg_thin	17018	50	17539	50						
bread_roll_spread_olive_chol_med	17552	50	17025	50						
bread_roll_spread_olive_chol_thick	17552	50	17025	50						
bread_roll_spread_olive_chol_thin	17552	50	17025	50						
bread_roll_spread_olive_dunno_med	17552	50	17025	50						
bread_roll_spread_olive_dunno_thick	17552	50	17025	50						
bread_roll_spread_olive_dunno_thin	17552	30	17025	70						
bread_roll_spread_olive_fat_med	17025	100								
bread_roll_spread_olive_fat_thick	17025	100								
bread_roll_spread_olive_fat_thin	17025	100								
bread_roll_spread_olive_lowfat_med	17552	100								
bread_roll_spread_olive_lowfat_thick	17552	100								
bread_roll_spread_olive_lowfat_thin	17552	100								
bread_roll_spread_olive_vlowfat_med	17028	100								
bread_roll_spread_olive_vlowfat_thick	17028	100								
bread_roll_spread_olive_vlowfat_thin	17028	100								
bread_roll_spread_other_med	17007	50	17487	50						
bread_roll_spread_other_thick	17007	50	17487	50						
bread_roll_spread_other_thin	17007	50	17487	50						
bread_roll_spread_polymarg_chol_med	17027	100								
bread_roll_spread_polymarg_chol_thick	17027	100								
bread_roll_spread_polymarg_chol_thin	17027	100								
bread_roll_spread_polymarg_dunno_med	17027	30	17024	70						
bread_roll_spread_polymarg_dunno_thick	17027	30	17024	70						
bread_roll_spread_polymarg_dunno_thin	17027	30	17024	70						
bread_roll_spread_polymarg_fat_med	17024	100								
bread_roll_spread_polymarg_fat_thick	17024	100								
bread_roll_spread_polymarg_fat_thin	17024	100								
bread_roll_spread_polymarg_lowfat_med	17027	100								
bread_roll_spread_polymarg_lowfat_thick	17027	100								
bread_roll_spread_polymarg_lowfat_thin	17027	100								
bread_roll_spread_polymarg_vlowfat_med	17029	100								
bread_roll_spread_polymarg_vlowfat_thick	17029	100								
bread_roll_spread_polymarg_vlowfat_thin	17029	100								
bread_roll_spread_soya_chol_med	17027	100								
bread_roll_spread_soya_chol_thick	17027	100								
bread_roll_spread_soya_chol_thin	17027	100								
bread_roll_spread_soya_dunno_med	17027	30	17024	70						
bread_roll_spread_soya_dunno_thick	17027	30	17024	70						
bread_roll_spread_soya_dunno_thin	17027	30	17024	70						
bread_roll_spread_soya_fat_med	17024	100								
bread_roll_spread_soya_fat_thick	17024	100								
bread_roll_spread_soya_fat_thin	17024	100								
bread_roll_spread_soya_lowfat_med	17027	100								
bread_roll_spread_soya_lowfat_thick	17027	100								
bread_roll_spread_soya_lowfat_thin	17027	100								
bread_roll_spread_soya_vlowfat_med	17029	100								
bread_roll_spread_soya_vlowfat_thick	17029	100								
bread_roll_spread_soya_vlowfat_thin	17029	100								
bread_roll_unanswered	00048	33.4	00056	33.3	00033	33.3				
bread_roll_white	11482	50	11481	50						
bread_roll_wholemeal	11484	100								
bread_sliced_mixed	11461	33.4	00033	33.3	11472	33.3				
bread_sliced_other	00040	50	00046	50						
bread_sliced_seeded	14844	50	14845	50						
bread_sliced_spread_butter_dunno_med	17486	100								
bread_sliced_spread_butter_dunno_thick	17486	100								
bread_sliced_spread_butter_dunno_thin	17486	100								
bread_sliced_spread_butter_fat_med	17485	100								
bread_sliced_spread_butter_fat_thick	17485	100								
bread_sliced_spread_butter_fat_thin	17485	100								
bread_sliced_spread_butter_lowfat_med	17485	50	17017	50						
bread_sliced_spread_butter_lowfat_thick	17485	50	17017	50						
bread_sliced_spread_butter_lowfat_thin	17485	50	17017	50						
bread_sliced_spread_butter_spread_fat_med	17486	100								
bread_sliced_spread_butter_spread_fat_thick	17486	100								
bread_sliced_spread_butter_spread_fat_thin	17486	100								
bread_sliced_spread_butter_spread_lowfat_med	17486	50	17017	50						
bread_sliced_spread_butter_spread_lowfat_thick	17486	50	17017	50						
bread_sliced_spread_butter_spread_lowfat_thin	17486	50	17017	50						
bread_sliced_spread_dairy_chol_med	17017	100								
bread_sliced_spread_dairy_chol_thick	17017	100								
bread_sliced_spread_dairy_chol_thin	17017	100								
bread_sliced_spread_dairy_dunno_med	17017	30	12258	70						
bread_sliced_spread_dairy_dunno_thick	17017	30	12258	70						
bread_sliced_spread_dairy_dunno_thin	17017	30	12258	70						
bread_sliced_spread_dairy_fat_med	12258	100								
bread_sliced_spread_dairy_fat_thick	12258	100								

Supplementary table 3. Nutrient calculation in the previous version (McCance and Widdowson).

	Food codes from McCance and Widdowson and the % used from each food code									
Item	Code1	% from code 1	Code2	% from code 2	Code3	% from code 3	Code4	% from code 4	Code5	% from code 5
bread_sliced_spread_dairy_fat_thin	12258	100								
bread_sliced_spread_dairy_lowfat_med	17017	100								
bread_sliced_spread_dairy_lowfat_thick	17017	100								
bread_sliced_spread_dairy_lowfat_thin	17017	100								
bread_sliced_spread_dairy_vlowfat_med	17028	100								
bread_sliced_spread_dairy_vlowfat_thick	17028	100								
bread_sliced_spread_dairy_vlowfat_thin	17028	100								
bread_sliced_spread_dunno_chol_med	17552	50	17027	50						
bread_sliced_spread_dunno_chol_thick	17552	50	17027	50						
bread_sliced_spread_dunno_chol_thin	17552	50	17027	50						
bread_sliced_spread_dunno_dunno_med	17552	25	17027	25	17025	25	17024	25		
bread_sliced_spread_dunno_dunno_thick	17552	25	17027	25	17025	25	17024	25		
bread_sliced_spread_dunno_dunno_thin	17552	25	17027	25	17025	25	17024	25		
bread_sliced_spread_dunno_fat_med	12258	33.3	17025	33.3	17024	33.4				
bread_sliced_spread_dunno_fat_thick	12258	33.3	17025	33.3	17024	33.4				
bread_sliced_spread_dunno_fat_thin	12258	33.3	17025	33.3	17024	33.4				
bread_sliced_spread_dunno_lowfat_med	17017	33.3	17552	33.3	17027	33.4				
bread_sliced_spread_dunno_lowfat_thick	17017	33.3	17552	33.3	17027	33.4				
bread_sliced_spread_dunno_lowfat_thin	17017	33.3	17552	33.3	17027	33.4				
bread_sliced_spread_dunno_vlowfat_med	17028	50	17029	50						
bread_sliced_spread_dunno_vlowfat_thick	17028	50	17029	50						
bread_sliced_spread_dunno_vlowfat_thin	17028	50	17029	50						
bread_sliced_spread_hardmarg_med	17018	50	17539	50						
bread_sliced_spread_hardmarg_thick	17018	50	17539	50						
bread_sliced_spread_hardmarg_thin	17018	50	17539	50						
bread_sliced_spread_olive_chol_med	17552	50	17025	50						
bread_sliced_spread_olive_chol_thick	17552	50	17025	50						
bread_sliced_spread_olive_chol_thin	17552	50	17025	50						
bread_sliced_spread_olive_dunno_med	17552	50	17025	50						
bread_sliced_spread_olive_dunno_thick	17552	50	17025	50						
bread_sliced_spread_olive_dunno_thin	17552	30	17025	70						
bread_sliced_spread_olive_fat_med	17025	100								
bread_sliced_spread_olive_fat_thick	17025	100								
bread_sliced_spread_olive_fat_thin	17025	100								
bread_sliced_spread_olive_lowfat_med	17552	100								
bread_sliced_spread_olive_lowfat_thick	17552	100								
bread_sliced_spread_olive_lowfat_thin	17552	100								
bread_sliced_spread_olive_vlowfat_med	17028	100								
bread_sliced_spread_olive_vlowfat_thick	17028	100								
bread_sliced_spread_olive_vlowfat_thin	17028	100								
bread_sliced_spread_other_med	17007	50	17487	50						
bread_sliced_spread_other_thick	17007	50	17487	50						
bread_sliced_spread_other_thin	17007	50	17487	50						
bread_sliced_spread_polymarg_chol_med	17027	100								
bread_sliced_spread_polymarg_chol_thick	17027	100								
bread_sliced_spread_polymarg_chol_thin	17027	100								
bread_sliced_spread_polymarg_dunno_med	17027	30	17024	70						
bread_sliced_spread_polymarg_dunno_thick	17027	30	17024	70						
bread_sliced_spread_polymarg_dunno_thin	17027	30	17024	70						
bread_sliced_spread_polymarg_fat_med	17024	100								
bread_sliced_spread_polymarg_fat_thick	17024	100								
bread_sliced_spread_polymarg_fat_thin	17024	100								
bread_sliced_spread_polymarg_lowfat_med	17027	100								
bread_sliced_spread_polymarg_lowfat_thick	17027	100								
bread_sliced_spread_polymarg_lowfat_thin	17027	100								
bread_sliced_spread_polymarg_vlowfat_med	17029	100								
bread_sliced_spread_polymarg_vlowfat_thick	17029	100								
bread_sliced_spread_polymarg_vlowfat_thin	17029	100								
bread_sliced_spread_soya_chol_med	17027	100								
bread_sliced_spread_soya_chol_thick	17027	100								
bread_sliced_spread_soya_chol_thin	17027	100								
bread_sliced_spread_soya_dunno_med	17027	30	17024	70						
bread_sliced_spread_soya_dunno_thick	17027	30	17024	70						
bread_sliced_spread_soya_dunno_thin	17027	30	17024	70						
bread_sliced_spread_soya_fat_med	17024	100								
bread_sliced_spread_soya_fat_thick	17024	100								
bread_sliced_spread_soya_fat_thin	17024	100								
bread_sliced_spread_soya_lowfat_med	17027	100								
bread_sliced_spread_soya_lowfat_thick	17027	100								
bread_sliced_spread_soya_lowfat_thin	17027	100								
bread_sliced_spread_soya_vlowfat_med	17029	100								
bread_sliced_spread_soya_vlowfat_thick	17029	100								
bread_sliced_spread_soya_vlowfat_thin	17029	100								
bread_sliced_unanswered	00048	33.4	00056	33.3	00033	33.3				
bread_sliced_white	00048	45	00054	10	11468	45				
bread_sliced_wholemeal	00056	100								
cake	11527	25	11571	25	11616	25	12394	25		
cereal_artf_swt										
cereal_bran	11485	50	11486	50						
cereal_bran_driedfruit	11493	100								
cereal_muesli	11494	50	11495	50						
cereal_muesli_driedfruit	11138	100								
cereal_oatcrunch	81361	50	11487	50						
cereal_oatcrunch_driedfruit	81361	45	11487	45	888	10				
cereal_other	81362	100								
cereal_other_driedfruit	81362	80	888	20						
cereal_plain	11490	33.4	11497	33.3	11501	33.3				
cereal_plain_driedfruit	11490	26.6	11497	26.7	11501	26.7	888	20		
cereal_porridge_milk	11570	100								
cereal_porridge_milk_driedfruit	11570	91	888	9						
cereal_porridge_water	11569	90	11496	10						
cereal_porridge_water_driedfruit	11569	83	11496	8	888	9				
cereal_sugar	17063	25	17074	25	17050	25	17065	25		
cereal_sweet	11491	25	11498	25	11488	25	11612	25		
cereal_sweet_driedfruit	11491	20	11498	20	11488	20	11612	20	888	20
cereal_wwheat	11499	33.4	11500	33.3	11505	33.3				
cereal_wwheat_driedfruit	11500	26.6	11501	26.7	11506	26.7	888	20		
cerealbar	17494	50	17103	50						
cheese_blue	12177	33.4	12354	33.3	12367	33.3				
cheese_cottage	12351	33.4	12352	33.3	12148	33.3				
cheese_feta	12356	100								
cheese_goat	12162	50	12357	50						
cheese_hard	12348	50	12359	50						
cheese_hard_lof	12155	33.4	12348	33.3	12355	33.3				
cheese_mozzarella	12170	50	12360	50						
cheese_other	12362	50	12368	50						
cheese_soft	12168	33.4	12344	33.3	12345	33.3				
cheese_spread	12143	25	12353	25	12364	25	12365	25		
cheese_spread_lof	12366	100								

Supplementary table 3. Nutrient calculation in the previous version (McCance and Widdowson).

	Food codes from McCance and Widdowson and the % used from each food code									
**Item**	**Code1**	**% from code 1**	**Code2**	**% from code 2**	**Code3**	**% from code 3**	**Code4**	**% from code 4**	**Code5**	**% from code 5**
cheesecake	12218	50	12395	50						
choc_bar	17547	50	17549	50						
choc_dark	17090	100								
choc_milk	17089	100								
choc_sweets	17088	100								
choc_white	17091	100								
chocyog_raisin	14835	100								
chutney	17341	50	17352	50						
cof_artf_swt										
cof_capp_decaf_other	17153	33.3	82002	66.7						
cof_capp_decaf_semi	17153	33.3	12313	66.7						
cof_capp_decaf_skimmed	17153	33.3	12307	66.7						
cof_capp_decaf_whole	17153	33.3	12316	66.7						
cof_capp_other	17153	33.3	82002	66.7						
cof_capp_semi	17153	33.3	12313	66.7						
cof_capp_skimmed	17153	33.3	12307	66.7						
cof_capp_whole	17153	33.3	12316	66.7						
cof_espresso	17153	100								
cof_espresso_decaf	17153	100								
cof_filter	17152	100								
cof_filter_decaf	17152	100								
cof_instant	17159	100								
cof_instant_decaf	17159	100								
cof_latte_decaf_other	17153	33.3	82002	66.7						
cof_latte_decaf_semi	17153	33.3	12313	66.7						
cof_latte_decaf_skimmed	17153	33.3	12307	66.7						
cof_latte_decaf_whole	17153	33.3	12316	66.7						
cof_latte_other	17153	33.3	82002	66.7						
cof_latte_semi	17153	33.3	12313	66.7						
cof_latte_skimmed	17153	33.3	12307	66.7						
cof_latte_whole	17153	33.3	12316	66.7						
cof_other	17159	100								
cof_other_decaf	17159	100								
cof_sugar	17063	66.7	17061	33.3						
cream	12332	20	12334	20	213	20	12335	20	12353	20
croissant	11480	100								
crumble	11439	25	11018	25	17061	14	17485	22	12346	14
danish_pastry	11538	100								
dessert_milkbased	12400	75	12397	25						
dessert_milkpuds	12225	17	12413	17	12406	33	12217	33		
dessert_other	12405	25	12830	25	12404	25	12252	25		
dessert_soya	12196	100								
double_crust	11585	**90**	11587	**10**						
doughnut	11539	50	11241	25	11242	25				
drink_diethotchoc	17500	10	12307	90						
drink_fizzy	17175	25	17177	25	17178	25	17179	25		
drink_grapefruit	14276	50	14275	50						
drink_hotchoc_other	17498	9	82002	91						
drink_hotchoc_semi	17532	100								
drink_hotchoc_skimmed	12096	100								
drink_hotchoc_whole	17533	100								
drink_lowcal	17505	50	87001	50						
drink_milkbased	12193	25	12326	25	12327	25	17203	25		
drink_orange	14281	100								
drink_other	17501	100								
drink_purejuice	14271	60	17537	30	13382	10				
drink_squash	17190	25	17195	25	17198	25	17201	25		
drink_water	01186	80	17182	20						
drizzle_oil	17038	100								
egg_omelet	12812	25	12926	25	12921	25	12922	25		
egg_other	12812	100								
egg_scotch	12825	50	19320	50						
egg_swich	293	90	17510	10						
egg_whole	12806	33.4	12810	33.3	12919	33.3				
fish_battered	16023	50	16054	50						
fish_breaded	16288	50	16281	50						
fish_lobcrab	16331	50	16332	50						
fish_oily	16176	20	16188	20	16192	20	16327	20	16329	20
fish_other	16013	33.4	16327	33.3	16192	33.3				
fish_prawns	16239	100								
fish_shell	16262	30	16256	70						
fish_tinnedtuna	16339	50	16230	50						
fish_white	16013	50	16045	50						
fruit_apple	14013	100								
fruit_banana	14045	100								
fruit_berry	14244	33.3	14260	33.4	14053	33.3				
fruit_cherry	14061	100								
fruit_dried	14031	33.4	14016	33.3	14242	33.3				
fruit_grapefruit	14105	100								
fruit_grapes	14109	100								
fruit_mango	14148	100								
fruit_melon	14153	100								
fruit_mixed	14303	50	14096	50						
fruit_orange	14176	100								
fruit_other	14124	50	14208	50						
fruit_peach	14183	50	14297	50						
fruit_pear	14191	100								
fruit_pineapple	14208	70	14211	30						
fruit_plum	14213	100								
fruit_prunes	14231	100								
fruit_satsuma	14258	100								
fruit_stewed	14005	33.4	14253	33.3	14215	33.3				
fruitcake	11577	50	11529	50						
grains_couscous	11339	100								
grains_other	11003	33.4	11007	33.3	14843	33.3				
guacamole	15180	100								
hummus	13433	100								
icecream	12387	33.4	12205	33.3	12200	33.3				
indian_snack	15227	25	15231	25	19059	25	175	25		
jam_honey	17074	33.4	17050	33.3	17065	33.3				
mayo	17317	50	17510	50						
mayo_lowfat	17511	100								
meat_bacon_nofat	19008	100								
meat_bacon_withfat	19003	50	19015	50						
meat_beef_nofat	18049	100								
meat_beef_withfat	373	100								
meat_ham_nofat	19308	80	19025	10	19027	10				
meat_ham_withfat	19308	60	19025	10	19027	10	19110	10	19142	10
meat_lamb_nofat	18141	100								

Supplementary table 3. Nutrient calculation in the previous version (McCance and Widdowson).

	Food codes from McCance and Widdowson and the % used from each food code									
Item	Code1	% from code 1	Code2	% from code 2	Code3	% from code 3	Code4	% from code 4	Code5	% from code 5
meat_lamb_withfat	18477	100								
meat_liverpate	488	20	18418	20	19317	60				
meat_other	18405	20	18374	40	19154	40				20
meat_pork_nofat	18251	100								
meat_pork_withfat	18252	100								
meat_sausage	19077	25	19080	75						
milk_chol_cereal	12313	50	12307	50						
milk_chol_coffee	12313	37.5	12307	37.5	12332	12.5	12027	12.5		
milk_chol_glass	12313	50	12307	50						
milk_chol_tea	12313	50	12307	50						
milk_dontknow_cereal	82002	100								
milk_dontknow_coffee	82002	75	12332	12.5	12027	12.5				
milk_dontknow_glass	82002	100								
milk_dontknow_tea	82002	100								
milk_goatsheep_cereal	12328	50	12329	50						
milk_goatsheep_coffee	12328	50	12329	50						
milk_goatsheep_glass	12328	50	12329	50						
milk_goatsheep_tea	12328	50	12329	50						
milk_other_cereal	82002	100								
milk_other_coffee	82002	75	12332	12.5	12027	12.5				
milk_other_glass	82002	100								
milk_other_tea	82002	100								
milk_powdered_cereal	12030	70	12031	30						
milk_powdered_coffee	12030	50	12031	25	12332	12.5	12027	12.5		
milk_powdered_glass	12030	70	12031	30						
milk_powdered_tea	12030	70	12031	30						
milk_riceoatveg_cereal	12042	50	12331	50						
milk_riceoatveg_coffee	12042	50	12331	50						
milk_riceoatveg_glass	12042	50	12331	50						
milk_riceoatveg_tea	12042	50	12331	50						
milk_semi_cereal	12313	100								
milk_semi_coffee	12313	75	12332	12.5	12027	12.5				
milk_semi_glass	12313	100								
milk_semi_tea	12313	100								
milk_skimmed_cereal	12307	100								
milk_skimmed_coffee	12307	75	12332	12.5	12027	12.5				
milk_skimmed_glass	12307	100								
milk_skimmed_tea	12307	100								
milk_soya_ca_cereal	12331	100								
milk_soya_ca_coffee	12331	100								
milk_soya_ca_glass	12331	100								
milk_soya_ca_tea	12331	100								
milk_soya_noca_cereal	12042	50	12331	50						
milk_soya_noca_coffee	12042	50	12331	50						
milk_soya_noca_glass	12042	50	12331	50						
milk_soya_noca_tea	12042	50	12331	50						
milk_whole_cereal	12316	100								
milk_whole_coffee	12316	75	12332	12.5	12027	12.5				
milk_whole_glass	12316	100								
milk_whole_tea	12316	100								
oatcakes	11518	100								
oatcakes_spread_butter_dunno_med	17486	100								
oatcakes_spread_butter_dunno_thick	17486	100								
oatcakes_spread_butter_dunno_thin	17486	100								
oatcakes_spread_butter_fat_med	17485	100								
oatcakes_spread_butter_fat_thick	17485	100								
oatcakes_spread_butter_fat_thin	17485	100								
oatcakes_spread_butter_lowfat_med	17485	50	17017	50						
oatcakes_spread_butter_lowfat_thick	17485	50	17017	50						
oatcakes_spread_butter_lowfat_thin	17485	50	17017	50						
oatcakes_spread_butter_spread_fat_med	17486	100								
oatcakes_spread_butter_spread_fat_thick	17486	100								
oatcakes_spread_butter_spread_fat_thin	17486	100								
oatcakes_spread_butter_spread_lowfat_med	17486	50	17017	50						
oatcakes_spread_butter_spread_lowfat_thick	17486	50	17017	50						
oatcakes_spread_butter_spread_lowfat_thin	17486	50	17017	50						
oatcakes_spread_dairy_chol_med	17017	100								
oatcakes_spread_dairy_chol_thick	17017	100								
oatcakes_spread_dairy_chol_thin	17017	100								
oatcakes_spread_dairy_dunno_med	17017	30	12258	70						
oatcakes_spread_dairy_dunno_thick	17017	30	12258	70						
oatcakes_spread_dairy_dunno_thin	17017	30	12258	70						
oatcakes_spread_dairy_fat_med	12258	100								
oatcakes_spread_dairy_fat_thick	12258	100								
oatcakes_spread_dairy_fat_thin	12258	100								
oatcakes_spread_dairy_lowfat_med	17017	100								
oatcakes_spread_dairy_lowfat_thick	17017	100								
oatcakes_spread_dairy_lowfat_thin	17017	100								
oatcakes_spread_dairy_vlowfat_med	17028	100								
oatcakes_spread_dairy_vlowfat_thick	17028	100								
oatcakes_spread_dairy_vlowfat_thin	17028	100								
oatcakes_spread_dunno_chol_med	17552	50	17027	50						
oatcakes_spread_dunno_chol_thick	17552	50	17027	50						
oatcakes_spread_dunno_chol_thin	17552	50	17027	50						
oatcakes_spread_dunno_dunno_med	17552	25	17027	25	17025	25	17024	25		
oatcakes_spread_dunno_dunno_thick	17552	25	17027	25	17025	25	17024	25		
oatcakes_spread_dunno_dunno_thin	17552	25	17027	25	17025	25	17024	25		
oatcakes_spread_dunno_fat_med	12258	33.3	17025	33.3	17024	33.4				
oatcakes_spread_dunno_fat_thick	12258	33.3	17025	33.3	17024	33.4				
oatcakes_spread_dunno_fat_thin	12258	33.3	17025	33.3	17024	33.4				
oatcakes_spread_dunno_lowfat_med	17017	33.3	17552	33.3	17027	33.4				
oatcakes_spread_dunno_lowfat_thick	17017	33.3	17552	33.3	17027	33.4				
oatcakes_spread_dunno_lowfat_thin	17017	33.3	17552	33.3	17027	33.4				
oatcakes_spread_dunno_vlowfat_med	17028	50	17029	50						
oatcakes_spread_dunno_vlowfat_thick	17028	50	17029	50						
oatcakes_spread_dunno_vlowfat_thin	17028	50	17029	50						
oatcakes_spread_hardmarg_med	17018	50	17539	50						
oatcakes_spread_hardmarg_thick	17018	50	17539	50						
oatcakes_spread_hardmarg_thin	17018	50	17539	50						
oatcakes_spread_olive_chol_med	17552	50	17025	50						
oatcakes_spread_olive_chol_thick	17552	50	17025	50						
oatcakes_spread_olive_chol_thin	17552	50	17025	50						
oatcakes_spread_olive_dunno_med	17552	50	17025	50						
oatcakes_spread_olive_dunno_thick	17552	50	17025	50						
oatcakes_spread_olive_dunno_thin	17552	30	17025	70						
oatcakes_spread_olive_fat_med	17025	100								
oatcakes_spread_olive_fat_thick	17025	100								
oatcakes_spread_olive_fat_thin	17025	100								
oatcakes_spread_olive_lowfat_med	17552	100								

Supplementary table 3. Nutrient calculation in the previous version (McCance and Widdowson).

	Food codes from McCance and Widdowson and the % used from each food code									
**Item**	**Code1**	**% from code 1**	**Code2**	**% from code 2**	**Code3**	**% from code 3**	**Code4**	**% from code 4**	**Code5**	**% from code 5**
oatcakes_spread_olive_lowfat_thick	17552	100								
oatcakes_spread_olive_lowfat_thin	17552	100								
oatcakes_spread_olive_vlowfat_med	17028	100								
oatcakes_spread_olive_vlowfat_thick	17028	100								
oatcakes_spread_olive_vlowfat_thin	17028	100								
oatcakes_spread_other_med	17007	50	17487	50						
oatcakes_spread_other_thick	17007	50	17487	50						
oatcakes_spread_other_thin	17007	50	17487	50						
oatcakes_spread_polymarg_chol_med	17027	100								
oatcakes_spread_polymarg_chol_thick	17027	100								
oatcakes_spread_polymarg_chol_thin	17027	100								
oatcakes_spread_polymarg_dunno_med	17027	30	17024	70						
oatcakes_spread_polymarg_dunno_thick	17027	30	17024	70						
oatcakes_spread_polymarg_dunno_thin	17027	30	17024	70						
oatcakes_spread_polymarg_fat_med	17024	100								
oatcakes_spread_polymarg_fat_thick	17024	100								
oatcakes_spread_polymarg_fat_thin	17024	100								
oatcakes_spread_polymarg_lowfat_med	17027	100								
oatcakes_spread_polymarg_lowfat_thick	17027	100								
oatcakes_spread_polymarg_lowfat_thin	17027	100								
oatcakes_spread_polymarg_vlowfat_med	17029	100								
oatcakes_spread_polymarg_vlowfat_thick	17029	100								
oatcakes_spread_polymarg_vlowfat_thin	17029	100								
oatcakes_spread_soya_chol_med	17027	100								
oatcakes_spread_soya_chol_thick	17027	100								
oatcakes_spread_soya_chol_thin	17027	100								
oatcakes_spread_soya_dunno_med	17027	30	17024	70						
oatcakes_spread_soya_dunno_thick	17027	30	17024	70						
oatcakes_spread_soya_dunno_thin	17027	30	17024	70						
oatcakes_spread_soya_fat_med	17024	100								
oatcakes_spread_soya_fat_thick	17024	100								
oatcakes_spread_soya_fat_thin	17024	100								
oatcakes_spread_soya_lowfat_med	17027	100								
oatcakes_spread_soya_lowfat_thick	17027	100								
oatcakes_spread_soya_lowfat_thin	17027	100								
oatcakes_spread_soya_vlowfat_med	17029	100								
oatcakes_spread_soya_vlowfat_thick	17029	100								
oatcakes_spread_soya_vlowfat_thin	17029	100								
pancake_blini	148	50	11544	50						
pancake_crepe	11347	50	11604	50						
pasta_brown	11455	100								
pasta_white	11450	25	11453	50	28	25				
pesto	15240	100								
pizza	15252	100								
pnutbutter_nutella	14876	70	17070	30						
potato_boil	13002	33.4	13004	33.3	13013	33.3				
potato_boil_marg	17485	50	12258	50						
potato_fried	13411	75	13016	25						
potato_mashed	13402	50	13015	50						
poultry_friedbcrumb_noskin	19122	67	19124	33						
poultry_friedbcrumb_withskin	19122	65	19124	30	18332	5				
poultry_noskin	18331	90	18361	10						
poultry_withskin	18341	90	463	10						
rice_brown	11443	100								
rice_white	11446	100								
salad_dressing	17509	100								
sauce_brown	1165	50	17289	50						
sauce_cheese	17522	100								
sauce_gravy	17311	100								
sauce_ketchup	17513	100								
sauce_tomato	17333	50	17516	50						
sauce_white	17528	100								
scone	11592	50	11543	50						
single_crust	11583	**40**	11585	**50**	11587	**10**				
smoothie_dairy	12193	100								
smoothie_fruit	14271	55	14045	20	14244	12.5	14260	12.5		
snack_cheesybis	11510	100								
snack_crisps	17495	50	17142	25	17497	25				
snack_olives	14173	100								
snack_saltednuts	14823	25	14840	25	14812	50				
snack_saltedpeanuts	14834	100								
snack_savourybis	11511	50	11510	50						
snack_seeds	14844	50	14845	50						
snack_svyother	14827	50	17495	50						
snack_swtother	17547	33.4	11480	33.3	11508	33.3				
snack_unsaltednuts	14811	33.4	14850	33.3	14870	33.3				
snack_unsaltedpeanuts	14831	100								
snackpot	17508	70	11057	30						
soup_canned_fish	17276	85	16256	5	16068	5	12332	5		
soup_canned_meat	17250	33.4	17271	33.3	17272	33.3				
soup_canned_other	17256	50	17275	50						
soup_canned_pasta	17542	100								
soup_canned_pulse	17264	100								
soup_canned_unanswered	17264	33.3	17250	33.3	17283	33.4				
soup_canned_veg	17270	25	17278	25	17283	25	17276	25		
soup_homemade_fish	17276	85	16256	5	16068	5	12332	5		
soup_homemade_meat	17250	33.4	17271	33.3	17272	33.3				
soup_homemade_other	17256	50	17275	50						
soup_homemade_pasta	17542	100								
soup_homemade_pulse	17264	100								
soup_homemade_unanswered	17264	33.3	17250	33.3	17283	33.4				
soup_homemade_veg	17270	25	17278	25	17283	25	17276	25		
soup_powder	17508	100								
spongepuds	164	100								
spreadsauce_other	17313	50	17364	25	17365	25				
sushi	11446	54	13340	1	16207	14	14037	9	13233	22
sweets	17101	25	17120	25	17104	25	17117	25		
sweets_diet	17101	50	17104	50						
tea_artf_swt										
tea_black	17165	100								
tea_black_decaf	17165	100								
tea_green	17171	100								
tea_herbal	17172	100								
tea_other	17170	100								
tea_rooibos	17165	100								
tea_sugar	17063	100								
veg_avocado	14037	100								
veg_bakedbeans	13044	100								
veg_beetroot	13166	50	13165	50						

Supplementary table 3. Nutrient calculation in the previous version (McCance and Widdowson).

	Food codes from McCance and Widdowson and the % used from each food code									
**Item**	**Code1**	**% from code 1**	**Code2**	**% from code 2**	**Code3**	**% from code 3**	**Code4**	**% from code 4**	**Code5**	**% from code 5**
veg_broadbeans	13065	100								
veg_broccoli	13171	100								
veg_butternut	13356	100								
veg_cabbagekale	13349	33.4	13184	33.3	13235	33.3				
veg_carrots	13201	53	13204	47						
veg_cauli	13216	100								
veg_celery	13221	100								
veg_courgette	13231	100								
veg_cucumber	13233	100								
veg_garlic	13244	100								
veg_greenbeans	13082	50	13113	50						
veg_leek	13264	100								
veg_lettuce	13266	100								
veg_mixed	13281	100								
veg_mixtures	13171	33.4	13201	33.3	13082	33.3				
veg_mushrooms	13284	100								
veg_onion	13304	100								
veg_other	13123	25	13159	25	13327	25	13220	25		
veg_parsnip	13313	100								
veg_peas	13133	90	13135	10						
veg_pepper_bell	13318	50	13320	50						
veg_pulses	13092	25	13111	25	13078	25	13072	25		
veg_saladmayo	15077	33.4	15078	33.3	15079	33.3				
veg_sidesalad	13266	25	13384	50	13233	25				
veg_spinach	13344	100								
veg_sprouts	13178	100								
veg_sweetcorn	13369	100								
veg_sweetpot	13363	50	13364	50						
veg_tomato_fresh	13384	100								
veg_tomato_tinned	13387	100								
veg_turnip	13390	50	13360	50						
veg_watercress	13462	100								
vegalt_burger	15330	100								
vegalt_other	15213	50	15202	30	13281	20				
vegalt_quorn	13328	100								
vegalt_tofu	13119	80	15314	20						
yeast_extract	17517	100								
yogurt_fullfat	12184	40	12375	40	12376	20				
yogurt_lowfat	12188	25	12189	25	12190	25	12382	25		
yorkshirepud	11607	50	11360	50						
