# Supplementary material for: Description of the updated nutrition calculation of the Oxford WebQ questionnaire and comparison with the previous version among 207,144 participants in UK Biobank
Source: Eur J Nutr. 2021 May 6;60(7):4019–30. doi: 10.1007/s00394-021-02558-4 (PMC8437868; doi:10.1007/s00394-021-02558-4)
Supplement: Supplementary file 4 — Supplementary file4 (DOCX 20 KB) [file 394_2021_2558_MOESM4_ESM.docx]

| **Supplementary Table 4.** Range of nutrient intake within each fifth in the previous (McCance & Widdowson) and updated (Nutrient databank + other updates) version of the nutrient calculation in 207,144 participants from UK Biobank. | | | | | |
| --- | --- | --- | --- | --- | --- |
|  | **Range of intake within each fifth** | | | | |
|  | **1** | **2** | **3** | **4** | **5** |
| **Energy** (kJ/day) |  |  |  |  |  |
| McCance & Widdowson | ≤ 6,788 | >6,789 to ≤ 7,952 | >7,953 to ≤ 9,039 | >9,040 to ≤ 10,456 | > 10,457 |
| Nutrient databank | ≤ 6,696 | >6,697 to ≤ 7,834 | >7,835 to ≤ 8,896 | >8,897 to ≤ 10,285 | > 10,286 |
| **Protein** (g/day) |  |  |  |  |  |
| McCance & Widdowson | ≤ 62.73 | >62.74 to ≤ 74.50 | >74.51 to ≤ 84.88 | >84.89 to ≤ 98.25 | > 98.26 |
| Nutrient databank | ≤ 61.96 | >61.97 to ≤ 73.36 | >73.37 to ≤ 83.52 | >83.53 to ≤ 96.73 | > 96.74 |
| **Total fat** (g/day) |  |  |  |  |  |
| McCance & Widdowson | ≤ 53.26 | >53.27 to ≤ 67.15 | >67.16 to ≤ 80.47 | >80.48 to ≤ 97.86 | > 97.87 |
| Nutrient databank | ≤ 50.06 | >50.07 to ≤ 63.09 | >63.10 to ≤ 75.73 | >75.74 to ≤ 92.27 | > 92.28 |
| **SFA** (g/day) |  |  |  |  |  |
| McCance & Widdowson | ≤ 19.36 | >19.37 to ≤ 25.11 | >25.12 to ≤ 30.72 | >30.73 to ≤ 38.21 | > 38.22 |
| Nutrient databank | ≤ 17.24 | >17.25 to ≤ 22.64 | >22.65 to ≤ 27.97 | >27.98 to ≤ 35.20 | > 35.21 |
| **PUFA** (g/day) |  |  |  |  |  |
| McCance & Widdowson | ≤ 8.37 | >8.38 to ≤ 11.51 | >11.52 to ≤ 14.76 | >14.77 to ≤ 19.17 | > 19.18 |
| Nutrient databank | ≤ 8.30 | >8.31 to ≤ 10.76 | >10.77 to ≤ 13.21 | >13.22 to ≤ 16.60 | > 16.61 |
| **Trans** **fat** (g/day) |  |  |  |  |  |
| McCance & Widdowson | ≤ 0.82 | >0.83 to ≤ 1.17 | >1.18 to ≤ 1.53 | >1.54 to ≤ 2.03 | > 2.04 |
| Nutrient databank | ≤ 0.65 | >0.66 to ≤ 0.94 | >0.95 to ≤ 1.22 | >1.23 to ≤ 1.62 | > 1.63 |
| **Carbohydrates** (g/day) |  |  |  |  |  |
| McCance & Widdowson | ≤ 188.19 | >188.20 to ≤ 226.22 | >226.23 to ≤ 261.23 | >261.24 to ≤ 306.53 | > 306.54 |
| Nutrient databank | ≤ 192.11 | >192.12 to ≤ 229.50 | >229.51 to ≤ 263.72 | >263.73 to ≤ 308.12 | > 308.13 |
| **Total sugars** (g/day) |  |  |  |  |  |
| McCance & Widdowson | ≤ 81.17 | >81.18 to ≤ 103.06 | >103.07 to ≤ 124.20 | >124.21 to ≤ 152.18 | > 152.19 |
| Nutrient databank | ≤ 86.03 | >86.04 to ≤ 108.32 | >108.33 to ≤ 129.75 | >129.76 to ≤ 158.06 | > 158.07 |
| **Fibre** (g/day) |  |  |  |  |  |
| McCance & Widdowson | ≤ 11.16 | >11.17 to ≤ 14.20 | >14.21 to ≤ 17.10 | >17.11 to ≤ 20.93 | > 20.94 |
| Nutrient databank | ≤ 12.50 | >12.51 to ≤ 15.66 | >15.67 to ≤ 18.62 | >18.63 to ≤ 22.43 | > 22.44 |
| **Alcohol** (g/day) |  |  |  |  |  |
| McCance & Widdowson | ≤ 0.00 | >0.01 to ≤ 3.25 | >3.26 to ≤ 14.73 | >14.74 to ≤ 30.46 | > 30.45 |
| Nutrient databank | ≤ 0.00 | >0.01 to ≤ 3.34 | >3.35 to ≤ 15.57 | >15.58 to ≤ 32.18 | > 32.19 |
| **Calcium** (mg/day) |  |  |  |  |  |
| McCance & Widdowson | ≤ 689.8 | >689.9 to ≤ 848.8 | >848.9 to ≤ 1,000.5 | >1,000.6 to ≤ 1,200.5 | > 1,200.6 |
| Nutrient databank | ≤ 707.4 | >707.5 to ≤ 868.7 | >868.8 to ≤ 1,019.2 | >1,019.3 to ≤ 1,217.8 | > 1,217.9 |
| **Iron** (mg/day) |  |  |  |  |  |
| McCance & Widdowson | ≤ 10.12 | >10.13 to ≤ 12.26 | >12.27 to ≤ 14.24 | >14.25 to ≤ 16.74 | > 16.75 |
| Nutrient databank | ≤ 9.29 | >9.30 to ≤ 11.15 | >11.16 to ≤ 12.86 | >12.87 to ≤ 15.04 | > 15.05 |
| **Magnesium** (mg/day) |  |  |  |  |  |
| McCance & Widdowson | ≤ 264.48 | >264.49 to ≤ 312.86 | >312.87 to ≤ 357.45 | >357.46 to ≤ 415.51 | > 415.52 |
| Nutrient databank | ≤ 257.65 | >257.66 to ≤ 302.61 | >302.62 to ≤ 344.00 | >344.01 to ≤ 397.27 | > 397.28 |
| **Potasium** (mg/day) |  |  |  |  |  |
| McCance & Widdowson | ≤ 2,830 | >2,831 to ≤ 3,365 | >3,366 to ≤ 3,858 | >3,859 to ≤ 4,488 | > 4,489 |
| Nutrient databank | ≤ 2,820 | >2,821 to ≤ 3,339 | >3,340 to ≤ 3,810 | >3,811 to ≤ 4,404 | > 4,405 |
| **Total carotene** (μg/day) |  |  |  |  |  |
| McCance & Widdowson | ≤ 986 | >987 to ≤ 1,984 | >1,985 to ≤ 3,091 | >3,092 to ≤ 4,721 | > 4,722 |
| Nutrient databank | ≤ 782 | >783 to ≤ 1,546 | >1,547 to ≤ 2,775 | >2,776 to ≤ 4,791 | > 4,792 |
| **Folate** (μg/day) |  |  |  |  |  |
| McCance & Widdowson | ≤ 214.38 | >214.39 to ≤ 264.19 | >264.20 to ≤ 311.89 | >311.90 to ≤ 377.07 | > 377.08 |
| Nutrient databank | ≤ 225.45 | >225.46 to ≤ 276.75 | >276.76 to ≤ 324.73 | >324.74 to ≤ 388.07 | > 388.08 |
| **Vitamin B6** (mg/day) |  |  |  |  |  |
| McCance & Widdowson | ≤ 1.59 | >1.60 to ≤ 1.95 | >1.96 to ≤ 2.27 | >2.28 to ≤ 2.69 | > 2.70 |
| Nutrient databank | ≤ 1.51 | >1.52 to ≤ 1.84 | >1.85 to ≤ 2.14 | >2.15 to ≤ 2.54 | > 2.55 |
| **Vitamin B12** (μg/day) |  |  |  |  |  |
| McCance & Widdowson | ≤ 3.18 | >3.19 to ≤ 4.63 | >4.64 to ≤ 6.27 | >6.28 to ≤ 9.01 | > 9.02 |
| Nutrient databank | ≤ 3.72 | >3.73 to ≤ 4.97 | >4.98 to ≤ 6.21 | >6.22 to ≤ 8.02 | > 8.03 |
| **Vitamin C** (mg/day) |  |  |  |  |  |
| McCance & Widdowson | ≤ 69.11 | >69.12 to ≤ 109.43 | >109.44 to ≤ 153.73 | >153.74 to ≤ 216.11 | > 216.12 |
| Nutrient databank | ≤ 63.83 | >63.84 to ≤ 98.26 | >98.27 to ≤ 133.01 | >133.02 to ≤ 180.50 | > 180.51 |
| **Vitamin D** (μg/day) |  |  |  |  |  |
| McCance & Widdowson | ≤ 0.97 | >0.98 to ≤ 1.64 | >1.65 to ≤ 2.50 | >2.51 to ≤ 4.20 | > 4.21 |
| Nutrient databank | ≤ 1.50 | >1.51 to ≤ 2.36 | >2.37 to ≤ 3.39 | >3.40 to ≤ 5.17 | > 5.18 |
| **Vitamin E** (mg/day) |  |  |  |  |  |
| McCance & Widdowson | ≤ 5.75 | >5.76 to ≤ 7.59 | >7.60 to ≤ 9.41 | >9.42 to ≤ 11.86 | > 11.87 |
| Nutrient databank | ≤ 7.29 | >7.30 to ≤ 9.34 | >9.35 to ≤ 11.32 | >11.33 to ≤ 13.98 | > 13.99 |
| **Retinol** (μg/day) |  |  |  |  |  |
| McCance & Widdowson | ≤ 177.40 | >177.41 to ≤ 259.57 | >259.58 to ≤ 343.42 | >343.43 to ≤ 455.91 | > 455.92 |
| Nutrient databank | ≤ 180.01 | >180.02 to ≤ 263.60 | >263.61 to ≤ 349.28 | >349.29 to ≤ 474.84 | > 474.85 |
