# Supplementary material for: Description of the updated nutrition calculation of the Oxford WebQ questionnaire and comparison with the previous version among 207,144 participants in UK Biobank
Source: Eur J Nutr. 2021 May 6;60(7):4019–30. doi: 10.1007/s00394-021-02558-4 (PMC8437868; doi:10.1007/s00394-021-02558-4)
Supplement: Supplementary file 5 — Supplementary file5 (PDF 838 KB) [file 394_2021_2558_MOESM5_ESM.pdf]

Supplementary Table 5. Food groups assigned to each food item (please see Piernas C. et al., 2021 EJON for further details about the food groups)

Food item	Food group name	Food group description
add_salt	N/A	N/A
alcohol_beercider	Beer & Cider	Beer and cider
alcohol_other	Spirits	Spirits and other alcoholic drinks
alcohol_spirits	Spirits	Spirits and other alcoholic drinks
alcohol_wine_fort	Fortified wine	Fortified wine
alcohol_wine_red_large	Red wine	Red and rose wine
alcohol_wine_red_med	Red wine	Red and rose wine
alcohol_wine_red_small	Red wine	Red and rose wine
alcohol_wine_rose_large	Red wine	Red and rose wine
alcohol_wine_rose_med	Red wine	Red and rose wine
alcohol_wine_rose_small	Red wine	Red and rose wine
alcohol_wine_white_large	White wine	White wine
alcohol_wine_white_med	White wine	White wine
alcohol_wine_white_small	White wine	White wine
biscuit_choc	Biscuits	Chocolate biscuits, plain biscuits, sweet biscuits and cookies
biscuit_choc_gf	Biscuits	Chocolate biscuits, plain biscuits, sweet biscuits and cookies
biscuit_choccov	Biscuits	Chocolate biscuits, plain biscuits, sweet biscuits and cookies
biscuit_choccov_gf	Biscuits	Chocolate biscuits, plain biscuits, sweet biscuits and cookies
biscuit_sweet	Biscuits	Chocolate biscuits, plain biscuits, sweet biscuits and cookies
biscuit_sweet_gf	Biscuits	Chocolate biscuits, plain biscuits, sweet biscuits and cookies
bread_baguette_gf_nonwhite	Other bread	Naan, garlic bread, other bread (including gluten free)
bread_baguette_gf_unanswered	White bread	White bread, sliced, baguette, bap, roll
bread_baguette_gf_white	White bread	White bread, sliced, baguette, bap, roll
bread_baguette_mixed	Mixed bread, brown & seeded	Mixed, brown or seeded bread, sliced, baguette, bap, roll
bread_baguette_other	Mixed bread, brown & seeded	Mixed, brown or seeded bread, sliced, baguette, bap, roll
bread_baguette_seeded	Unsalted nuts & seeds	Unsalted peanuts and nuts
bread_baguette_spread_butter_dunno_med	Dairy fat spread	Spreadable normal fat butter, dairy-based normal fat spread (including cholesterol lowering spread)
bread_baguette_spread_butter_dunno_thick	Dairy fat spread	Spreadable normal fat butter, dairy-based normal fat spread (including cholesterol lowering spread)
bread_baguette_spread_butter_dunno_thin	Dairy fat spread	Spreadable normal fat butter, dairy-based normal fat spread (including cholesterol lowering spread)
bread_baguette_spread_butter_fat_med	Dairy fat spread	Spreadable normal fat butter, dairy-based normal fat spread (including cholesterol lowering spread)
bread_baguette_spread_butter_fat_thick	Dairy fat spread	Spreadable normal fat butter, dairy-based normal fat spread (including cholesterol lowering spread)
bread_baguette_spread_butter_fat_thin	Dairy fat spread	Spreadable normal fat butter, dairy-based normal fat spread (including cholesterol lowering spread)
bread_baguette_spread_butter_lowfat_med	Dairy fat spread lower fat	Spreadable/lower fat butter, dairy-based very low fat spread
bread_baguette_spread_butter_lowfat_thick	Dairy fat spread lower fat	Spreadable/lower fat butter, dairy-based very low fat spread
bread_baguette_spread_butter_lowfat_thin	Dairy fat spread lower fat	Spreadable/lower fat butter, dairy-based very low fat spread
bread_baguette_spread_butter_spread_fat_med	Dairy fat spread	Spreadable normal fat butter, dairy-based normal fat spread (including cholesterol lowering spread)
bread_baguette_spread_butter_spread_fat_thick	Dairy fat spread	Spreadable normal fat butter, dairy-based normal fat spread (including cholesterol lowering spread)
bread_baguette_spread_butter_spread_fat_thin	Dairy fat spread	Spreadable normal fat butter, dairy-based normal fat spread (including cholesterol lowering spread)
bread_baguette_spread_butter_spread_lowfat_med	Dairy fat spread lower fat	Spreadable/lower fat butter, dairy-based very low fat spread
bread_baguette_spread_butter_spread_lowfat_thick	Dairy fat spread lower fat	Spreadable/lower fat butter, dairy-based very low fat spread
bread_baguette_spread_butter_spread_lowfat_thin	Dairy fat spread lower fat	Spreadable/lower fat butter, dairy-based very low fat spread
bread_baguette_spread_dairy_chol_med	Dairy fat spread	Spreadable normal fat butter, dairy-based normal fat spread (including cholesterol lowering spread)
bread_baguette_spread_dairy_chol_thick	Dairy fat spread	Spreadable normal fat butter, dairy-based normal fat spread (including cholesterol lowering spread)
bread_baguette_spread_dairy_chol_thin	Dairy fat spread	Spreadable normal fat butter, dairy-based normal fat spread (including cholesterol lowering spread)
bread_baguette_spread_dairy_dunno_med	Dairy fat spread	Spreadable normal fat butter, dairy-based normal fat spread (including cholesterol lowering spread)
bread_baguette_spread_dairy_dunno_thick	Dairy fat spread	Spreadable normal fat butter, dairy-based normal fat spread (including cholesterol lowering spread)
bread_baguette_spread_dairy_dunno_thin	Dairy fat spread	Spreadable normal fat butter, dairy-based normal fat spread (including cholesterol lowering spread)
bread_baguette_spread_dairy_fat_med	Dairy fat spread	Spreadable normal fat butter, dairy-based normal fat spread (including cholesterol lowering spread)
bread_baguette_spread_dairy_fat_thick	Dairy fat spread	Spreadable normal fat butter, dairy-based normal fat spread (including cholesterol lowering spread)
bread_baguette_spread_dairy_fat_thin	Dairy fat spread	Spreadable normal fat butter, dairy-based normal fat spread (including cholesterol lowering spread)
bread_baguette_spread_dairy_lowfat_med	Dairy fat spread lower fat	Spreadable/lower fat butter, dairy-based very low fat spread
bread_baguette_spread_dairy_lowfat_thick	Dairy fat spread lower fat	Spreadable/lower fat butter, dairy-based very low fat spread
bread_baguette_spread_dairy_lowfat_thin	Dairy fat spread lower fat	Spreadable/lower fat butter, dairy-based very low fat spread
bread_baguette_spread_dairy_vlowfat_med	Dairy fat spread lower fat	Spreadable/lower fat butter, dairy-based very low fat spread
bread_baguette_spread_dairy_vlowfat_thick	Dairy fat spread lower fat	Spreadable/lower fat butter, dairy-based very low fat spread
bread_baguette_spread_dairy_vlowfat_thin	Dairy fat spread lower fat	Spreadable/lower fat butter, dairy-based very low fat spread
bread_baguette_spread_dunno_chol_med	Vegetable spread lower fat	Olive oil based lower fat spread, plant-based lower fat margarine and soya-based lower fat spread (including cholesterol lowering spread)
bread_baguette_spread_dunno_chol_thick	Vegetable spread lower fat	Olive oil based lower fat spread, plant-based lower fat margarine and soya-based lower fat spread (including cholesterol lowering spread)
bread_baguette_spread_dunno_chol_thin	Vegetable spread lower fat	Olive oil based lower fat spread, plant-based lower fat margarine and soya-based lower fat spread (including cholesterol lowering spread)
bread_baguette_spread_dunno_dunno_med	Vegetable spread	Olive oil based spread, plant-based soft or hard margarine and soya-based spread (including cholesterol lowering spread)
bread_baguette_spread_dunno_dunno_thick	Vegetable spread	Olive oil based spread, plant-based soft or hard margarine and soya-based spread (including cholesterol lowering spread)
bread_baguette_spread_dunno_dunno_thin	Vegetable spread	Olive oil based spread, plant-based soft or hard margarine and soya-based spread (including cholesterol lowering spread)
bread_baguette_spread_dunno_fat_med	Vegetable spread	Olive oil based spread, plant-based soft or hard margarine and soya-based spread (including cholesterol lowering spread)
bread_baguette_spread_dunno_fat_thick	Vegetable spread	Olive oil based spread, plant-based soft or hard margarine and soya-based spread (including cholesterol lowering spread)
bread_baguette_spread_dunno_fat_thin	Vegetable spread	Olive oil based spread, plant-based soft or hard margarine and soya-based spread (including cholesterol lowering spread)
bread_baguette_spread_dunno_lowfat_med	Vegetable spread lower fat	Olive oil based lower fat spread, plant-based lower fat margarine and soya-based lower fat spread (including cholesterol lowering spread)
bread_baguette_spread_dunno_lowfat_thick	Vegetable spread lower fat	Olive oil based lower fat spread, plant-based lower fat margarine and soya-based lower fat spread (including cholesterol lowering spread)
bread_baguette_spread_dunno_lowfat_thin	Vegetable spread lower fat	Olive oil based lower fat spread, plant-based lower fat margarine and soya-based lower fat spread (including cholesterol lowering spread)
bread_baguette_spread_dunno_vlowfat_med	Vegetable spread lower fat	Olive oil based lower fat spread, plant-based lower fat margarine and soya-based lower fat spread (including cholesterol lowering spread)
bread_baguette_spread_dunno_vlowfat_thick	Vegetable spread lower fat	Olive oil based lower fat spread, plant-based lower fat margarine and soya-based lower fat spread (including cholesterol lowering spread)
bread_baguette_spread_dunno_vlowfat_thin	Vegetable spread lower fat	Olive oil based lower fat spread, plant-based lower fat margarine and soya-based lower fat spread (including cholesterol lowering spread)
bread_baguette_spread_hardmarg_med	Vegetable spread	Olive oil based spread, plant-based soft or hard margarine and soya-based spread (including cholesterol lowering spread)
bread_baguette_spread_hardmarg_thick	Vegetable spread	Olive oil based spread, plant-based soft or hard margarine and soya-based spread (including cholesterol lowering spread)
bread_baguette_spread_hardmarg_thin	Vegetable spread	Olive oil based spread, plant-based soft or hard margarine and soya-based spread (including cholesterol lowering spread)
bread_baguette_spread_olive_chol_med	Vegetable spread	Olive oil based spread, plant-based soft or hard margarine and soya-based spread (including cholesterol lowering spread)
bread_baguette_spread_olive_chol_thick	Vegetable spread	Olive oil based spread, plant-based soft or hard margarine and soya-based spread (including cholesterol lowering spread)
bread_baguette_spread_olive_chol_thin	Vegetable spread	Olive oil based spread, plant-based soft or hard margarine and soya-based spread (including cholesterol lowering spread)
bread_baguette_spread_olive_dunno_med	Vegetable spread	Olive oil based spread, plant-based soft or hard margarine and soya-based spread (including cholesterol lowering spread)
bread_baguette_spread_olive_dunno_thick	Vegetable spread	Olive oil based spread, plant-based soft or hard margarine and soya-based spread (including cholesterol lowering spread)
bread_baguette_spread_olive_dunno_thin	Vegetable spread	Olive oil based spread, plant-based soft or hard margarine and soya-based spread (including cholesterol lowering spread)
bread_baguette_spread_olive_fat_med	Vegetable spread	Olive oil based spread, plant-based soft or hard margarine and soya-based spread (including cholesterol lowering spread)
bread_baguette_spread_olive_fat_thick	Vegetable spread	Olive oil based spread, plant-based soft or hard margarine and soya-based spread (including cholesterol lowering spread)
bread_baguette_spread_olive_fat_thin	Vegetable spread	Olive oil based spread, plant-based soft or hard margarine and soya-based spread (including cholesterol lowering spread)
bread_baguette_spread_olive_lowfat_med	Vegetable spread lower fat	Olive oil based lower fat spread, plant-based lower fat margarine and soya-based lower fat spread (including cholesterol lowering spread)
bread_baguette_spread_olive_lowfat_thick	Vegetable spread lower fat	Olive oil based lower fat spread, plant-based lower fat margarine and soya-based lower fat spread (including cholesterol lowering spread)
bread_baguette_spread_olive_lowfat_thin	Vegetable spread lower fat	Olive oil based lower fat spread, plant-based lower fat margarine and soya-based lower fat spread (including cholesterol lowering spread)
bread_baguette_spread_olive_vlowfat_med	Vegetable spread lower fat	Olive oil based lower fat spread, plant-based lower fat margarine and soya-based lower fat spread (including cholesterol lowering spread)
bread_baguette_spread_olive_vlowfat_thick	Vegetable spread lower fat	Olive oil based lower fat spread, plant-based lower fat margarine and soya-based lower fat spread (including cholesterol lowering spread)
bread_baguette_spread_olive_vlowfat_thin	Vegetable spread lower fat	Olive oil based lower fat spread, plant-based lower fat margarine and soya-based lower fat spread (including cholesterol lowering spread)
bread_baguette_spread_other_med	Dairy fat spread	Spreadable normal fat butter, dairy-based normal fat spread (including cholesterol lowering spread)
bread_baguette_spread_other_thick	Dairy fat spread	Spreadable normal fat butter, dairy-based normal fat spread (including cholesterol lowering spread)
bread_baguette_spread_other_thin	Dairy fat spread	Spreadable normal fat butter, dairy-based normal fat spread (including cholesterol lowering spread)
bread_baguette_spread_polymarg_chol_med	Vegetable spread	Olive oil based spread, plant-based soft or hard margarine and soya-based spread (including cholesterol lowering spread)
bread_baguette_spread_polymarg_chol_thick	Vegetable spread	Olive oil based spread, plant-based soft or hard margarine and soya-based spread (including cholesterol lowering spread)
bread_baguette_spread_polymarg_chol_thin	Vegetable spread	Olive oil based spread, plant-based soft or hard margarine and soya-based spread (including cholesterol lowering spread)
bread_baguette_spread_polymarg_dunno_med	Vegetable spread	Olive oil based spread, plant-based soft or hard margarine and soya-based spread (including cholesterol lowering spread)
bread_baguette_spread_polymarg_dunno_thick	Vegetable spread	Olive oil based spread, plant-based soft or hard margarine and soya-based spread (including cholesterol lowering spread)
bread_baguette_spread_polymarg_dunno_thin	Vegetable spread	Olive oil based spread, plant-based soft or hard margarine and soya-based spread (including cholesterol lowering spread)
bread_baguette_spread_polymarg_fat_med	Vegetable spread	Olive oil based spread, plant-based soft or hard margarine and soya-based spread (including cholesterol lowering spread)
bread_baguette_spread_polymarg_fat_thick	Vegetable spread	Olive oil based spread, plant-based soft or hard margarine and soya-based spread (including cholesterol lowering spread)
bread_baguette_spread_polymarg_fat_thin	Vegetable spread	Olive oil based spread, plant-based soft or hard margarine and soya-based spread (including cholesterol lowering spread)
bread_baguette_spread_polymarg_lowfat_med	Vegetable spread lower fat	Olive oil based lower fat spread, plant-based lower fat margarine and soya-based lower fat spread (including cholesterol lowering spread)
bread_baguette_spread_polymarg_lowfat_thick	Vegetable spread lower fat	Olive oil based lower fat spread, plant-based lower fat margarine and soya-based lower fat spread (including cholesterol lowering spread)
bread_baguette_spread_polymarg_lowfat_thin	Vegetable spread lower fat	Olive oil based lower fat spread, plant-based lower fat margarine and soya-based lower fat spread (including cholesterol lowering spread)
bread_baguette_spread_polymarg_vlowfat_med	Vegetable spread lower fat	Olive oil based lower fat spread, plant-based lower fat margarine and soya-based lower fat spread (including cholesterol lowering spread)
bread_baguette_spread_polymarg_vlowfat_thick	Vegetable spread lower fat	Olive oil based lower fat spread, plant-based lower fat margarine and soya-based lower fat spread (including cholesterol lowering spread)
bread_baguette_spread_polymarg_vlowfat_thin	Vegetable spread lower fat	Olive oil based lower fat spread, plant-based lower fat margarine and soya-based lower fat spread (including cholesterol lowering spread)
bread_baguette_spread_soya_chol_med	Vegetable spread	Olive oil based spread, plant-based soft or hard margarine and soya-based spread (including cholesterol lowering spread)
bread_baguette_spread_soya_chol_thick	Vegetable spread	Olive oil based spread, plant-based soft or hard margarine and soya-based spread (including cholesterol lowering spread)
bread_baguette_spread_soya_chol_thin	Vegetable spread	Olive oil based spread, plant-based soft or hard margarine and soya-based spread (including cholesterol lowering spread)
bread_baguette_spread_soya_dunno_med	Vegetable spread	Olive oil based spread, plant-based soft or hard margarine and soya-based spread (including cholesterol lowering spread)
bread_baguette_spread_soya_dunno_thick	Vegetable spread	Olive oil based spread, plant-based soft or hard margarine and soya-based spread (including cholesterol lowering spread)
bread_baguette_spread_soya_dunno_thin	Vegetable spread	Olive oil based spread, plant-based soft or hard margarine and soya-based spread (including cholesterol lowering spread)
bread_baguette_spread_soya_fat_med	Vegetable spread	Olive oil based spread, plant-based soft or hard margarine and soya-based spread (including cholesterol lowering spread)
bread_baguette_spread_soya_fat_thick	Vegetable spread	Olive oil based spread, plant-based soft or hard margarine and soya-based spread (including cholesterol lowering spread)
bread_baguette_spread_soya_fat_thin	Vegetable spread	Olive oil based spread, plant-based soft or hard margarine and soya-based spread (including cholesterol lowering spread)
bread_baguette_spread_soya_lowfat_med	Vegetable spread lower fat	Olive oil based lower fat spread, plant-based lower fat margarine and soya-based lower fat spread (including cholesterol lowering spread)
bread_baguette_spread_soya_lowfat_thick	Vegetable spread lower fat	Olive oil based lower fat spread, plant-based lower fat margarine and soya-based lower fat spread (including cholesterol lowering spread)
bread_baguette_spread_soya_lowfat_thin	Vegetable spread lower fat	Olive oil based lower fat spread, plant-based lower fat margarine and soya-based lower fat spread (including cholesterol lowering spread)
bread_baguette_spread_soya_vlowfat_med	Vegetable spread lower fat	Olive oil based lower fat spread, plant-based lower fat margarine and soya-based lower fat spread (including cholesterol lowering spread)
bread_baguette_spread_soya_vlowfat_thick	Vegetable spread lower fat	Olive oil based lower fat spread, plant-based lower fat margarine and soya-based lower fat spread (including cholesterol lowering spread)
bread_baguette_spread_soya_vlowfat_thin	Vegetable spread lower fat	Olive oil based lower fat spread, plant-based lower fat margarine and soya-based lower fat spread (including cholesterol lowering spread)
bread_baguette_unanswered	White bread	White bread, sliced, baguette, bap, roll
bread_baguette_white	White bread	White bread, sliced, baguette, bap, roll
bread_baguette_wholemeal	Wholemeal bread	Wholemeal bread, sliced, baguette, bap, roll
bread_crisp	Savoury crackers	Oatcakes, crispbreads (including gluten free)
bread_crisp_gf	Savoury crackers	Oatcakes, crispbreads (including gluten free)
bread_crisp_spread_butter_dunno_med	Dairy fat spread	Spreadable normal fat butter, dairy-based normal fat spread (including cholesterol lowering spread)
bread_crisp_spread_butter_dunno_thick	Dairy fat spread	Spreadable normal fat butter, dairy-based normal fat spread (including cholesterol lowering spread)
bread_crisp_spread_butter_dunno_thin	Dairy fat spread	Spreadable normal fat butter, dairy-based normal fat spread (including cholesterol lowering spread)
bread_crisp_spread_butter_fat_med	Dairy fat spread	Spreadable normal fat butter, dairy-based normal fat spread (including cholesterol lowering spread)
bread_crisp_spread_butter_fat_thick	Dairy fat spread	Spreadable normal fat butter, dairy-based normal fat spread (including cholesterol lowering spread)
bread_crisp_spread_butter_fat_thin	Dairy fat spread	Spreadable normal fat butter, dairy-based normal fat spread (including cholesterol lowering spread)
bread_crisp_spread_butter_lowfat_med	Dairy fat spread lower fat	Spreadable/lower fat butter, dairy-based very low fat spread
bread_crisp_spread_butter_lowfat_thick	Dairy fat spread lower fat	Spreadable/lower fat butter, dairy-based very low fat spread
bread_crisp_spread_butter_lowfat_thin	Dairy fat spread lower fat	Spreadable/lower fat butter, dairy-based very low fat spread
bread_crisp_spread_butter_spread_fat_med	Dairy fat spread	Spreadable normal fat butter, dairy-based normal fat spread (including cholesterol lowering spread)
bread_crisp_spread_butter_spread_fat_thick	Dairy fat spread	Spreadable normal fat butter, dairy-based normal fat spread (including cholesterol lowering spread)
bread_crisp_spread_butter_spread_fat_thin	Dairy fat spread	Spreadable normal fat butter, dairy-based normal fat spread (including cholesterol lowering spread)
bread_crisp_spread_butter_spread_lowfat_med	Dairy fat spread lower fat	Spreadable/lower fat butter, dairy-based very low fat spread
bread_crisp_spread_butter_spread_lowfat_thick	Dairy fat spread lower fat	Spreadable/lower fat butter, dairy-based very low fat spread
bread_crisp_spread_butter_spread_lowfat_thin	Dairy fat spread lower fat	Spreadable/lower fat butter, dairy-based very low fat spread
bread_crisp_spread_dairy_chol_med	Dairy fat spread	Spreadable normal fat butter, dairy-based normal fat spread (including cholesterol lowering spread)
bread_crisp_spread_dairy_chol_thick	Dairy fat spread	Spreadable normal fat butter, dairy-based normal fat spread (including cholesterol lowering spread)
bread_crisp_spread_dairy_chol_thin	Dairy fat spread	Spreadable normal fat butter, dairy-based normal fat spread (including cholesterol lowering spread)
bread_crisp_spread_dairy_dunno_med	Dairy fat spread	Spreadable normal fat butter, dairy-based normal fat spread (including cholesterol lowering spread)
bread_crisp_spread_dairy_dunno_thick	Dairy fat spread	Spreadable normal fat butter, dairy-based normal fat spread (including cholesterol lowering spread)
bread_crisp_spread_dairy_dunno_thin	Dairy fat spread	Spreadable normal fat butter, dairy-based normal fat spread (including cholesterol lowering spread)
bread_crisp_spread_dairy_fat_med	Dairy fat spread	Spreadable normal fat butter, dairy-based normal fat spread (including cholesterol lowering spread)

bread_crisp_spread_dairy_fat_thick	Dairy fat spread	Spreadable normal fat butter, dairy-based normal fat spread (including cholesterol lowering spread)
bread_crisp_spread_dairy_fat_thin	Dairy fat spread	Spreadable normal fat butter, dairy-based normal fat spread (including cholesterol lowering spread)
bread_crisp_spread_dairy_lowfat_med	Dairy fat spread lower fat	Spreadable/lower fat butter, dairy-based very low fat spread
bread_crisp_spread_dairy_lowfat_thick	Dairy fat spread lower fat	Spreadable/lower fat butter, dairy-based very low fat spread
bread_crisp_spread_dairy_lowfat_thin	Dairy fat spread lower fat	Spreadable/lower fat butter, dairy-based very low fat spread
bread_crisp_spread_dairy_vlowfat_med	Dairy fat spread lower fat	Spreadable/lower fat butter, dairy-based very low fat spread
bread_crisp_spread_dairy_vlowfat_thick	Dairy fat spread lower fat	Spreadable/lower fat butter, dairy-based very low fat spread
bread_crisp_spread_dairy_vlowfat_thin	Dairy fat spread lower fat	Spreadable/lower fat butter, dairy-based very low fat spread
bread_crisp_spread_dunno_chol_med	Vegetable spread lower fat	Olive oil based lower fat spread, plant-based lower fat margarine and soya-based lower fat spread (including cholesterol lowering spread)
bread_crisp_spread_dunno_chol_thick	Vegetable spread lower fat	Olive oil based lower fat spread, plant-based lower fat margarine and soya-based lower fat spread (including cholesterol lowering spread)
bread_crisp_spread_dunno_chol_thin	Vegetable spread lower fat	Olive oil based lower fat spread, plant-based lower fat margarine and soya-based lower fat spread (including cholesterol lowering spread)
bread_crisp_spread_dunno_dunno_med	Vegetable spread	Olive oil based spread, plant-based soft or hard margarine and soya-based spread (including cholesterol lowering spread)
bread_crisp_spread_dunno_dunno_thick	Vegetable spread	Olive oil based spread, plant-based soft or hard margarine and soya-based spread (including cholesterol lowering spread)
bread_crisp_spread_dunno_dunno_thin	Vegetable spread	Olive oil based spread, plant-based soft or hard margarine and soya-based spread (including cholesterol lowering spread)
bread_crisp_spread_dunno_fat_med	Vegetable spread	Olive oil based spread, plant-based soft or hard margarine and soya-based spread (including cholesterol lowering spread)
bread_crisp_spread_dunno_fat_thick	Vegetable spread	Olive oil based spread, plant-based soft or hard margarine and soya-based spread (including cholesterol lowering spread)
bread_crisp_spread_dunno_fat_thin	Vegetable spread	Olive oil based spread, plant-based soft or hard margarine and soya-based spread (including cholesterol lowering spread)
bread_crisp_spread_dunno_lowfat_med	Vegetable spread lower fat	Olive oil based lower fat spread, plant-based lower fat margarine and soya-based lower fat spread (including cholesterol lowering spread)
bread_crisp_spread_dunno_lowfat_thick	Vegetable spread lower fat	Olive oil based lower fat spread, plant-based lower fat margarine and soya-based lower fat spread (including cholesterol lowering spread)
bread_crisp_spread_dunno_lowfat_thin	Vegetable spread lower fat	Olive oil based lower fat spread, plant-based lower fat margarine and soya-based lower fat spread (including cholesterol lowering spread)
bread_crisp_spread_dunno_vlowfat_med	Vegetable spread lower fat	Olive oil based lower fat spread, plant-based lower fat margarine and soya-based lower fat spread (including cholesterol lowering spread)
bread_crisp_spread_dunno_vlowfat_thick	Vegetable spread lower fat	Olive oil based lower fat spread, plant-based lower fat margarine and soya-based lower fat spread (including cholesterol lowering spread)
bread_crisp_spread_dunno_vlowfat_thin	Vegetable spread lower fat	Olive oil based lower fat spread, plant-based lower fat margarine and soya-based lower fat spread (including cholesterol lowering spread)
bread_crisp_spread_hardmarg_med	Vegetable spread	Olive oil based spread, plant-based soft or hard margarine and soya-based spread (including cholesterol lowering spread)
bread_crisp_spread_hardmarg_thick	Vegetable spread	Olive oil based spread, plant-based soft or hard margarine and soya-based spread (including cholesterol lowering spread)
bread_crisp_spread_hardmarg_thin	Vegetable spread	Olive oil based spread, plant-based soft or hard margarine and soya-based spread (including cholesterol lowering spread)
bread_crisp_spread_olive_chol_med	Vegetable spread	Olive oil based spread, plant-based soft or hard margarine and soya-based spread (including cholesterol lowering spread)
bread_crisp_spread_olive_chol_thick	Vegetable spread	Olive oil based spread, plant-based soft or hard margarine and soya-based spread (including cholesterol lowering spread)
bread_crisp_spread_olive_chol_thin	Vegetable spread	Olive oil based spread, plant-based soft or hard margarine and soya-based spread (including cholesterol lowering spread)
bread_crisp_spread_olive_dunno_med	Vegetable spread	Olive oil based spread, plant-based soft or hard margarine and soya-based spread (including cholesterol lowering spread)
bread_crisp_spread_olive_dunno_thick	Vegetable spread	Olive oil based spread, plant-based soft or hard margarine and soya-based spread (including cholesterol lowering spread)
bread_crisp_spread_olive_dunno_thin	Vegetable spread	Olive oil based spread, plant-based soft or hard margarine and soya-based spread (including cholesterol lowering spread)
bread_crisp_spread_olive_fat_med	Vegetable spread	Olive oil based spread, plant-based soft or hard margarine and soya-based spread (including cholesterol lowering spread)
bread_crisp_spread_olive_fat_thick	Vegetable spread	Olive oil based spread, plant-based soft or hard margarine and soya-based spread (including cholesterol lowering spread)
bread_crisp_spread_olive_fat_thin	Vegetable spread	Olive oil based spread, plant-based soft or hard margarine and soya-based spread (including cholesterol lowering spread)
bread_crisp_spread_olive_lowfat_med	Vegetable spread lower fat	Olive oil based lower fat spread, plant-based lower fat margarine and soya-based lower fat spread (including cholesterol lowering spread)
bread_crisp_spread_olive_lowfat_thick	Vegetable spread lower fat	Olive oil based lower fat spread, plant-based lower fat margarine and soya-based lower fat spread (including cholesterol lowering spread)
bread_crisp_spread_olive_lowfat_thin	Vegetable spread lower fat	Olive oil based lower fat spread, plant-based lower fat margarine and soya-based lower fat spread (including cholesterol lowering spread)
bread_crisp_spread_olive_vlowfat_med	Vegetable spread lower fat	Olive oil based lower fat spread, plant-based lower fat margarine and soya-based lower fat spread (including cholesterol lowering spread)
bread_crisp_spread_olive_vlowfat_thick	Vegetable spread lower fat	Olive oil based lower fat spread, plant-based lower fat margarine and soya-based lower fat spread (including cholesterol lowering spread)
bread_crisp_spread_olive_vlowfat_thin	Vegetable spread lower fat	Olive oil based lower fat spread, plant-based lower fat margarine and soya-based lower fat spread (including cholesterol lowering spread)
bread_crisp_spread_other_med	Dairy fat spread	Spreadable normal fat butter, dairy-based normal fat spread (including cholesterol lowering spread)
bread_crisp_spread_other_thick	Dairy fat spread	Spreadable normal fat butter, dairy-based normal fat spread (including cholesterol lowering spread)
bread_crisp_spread_other_thin	Dairy fat spread	Spreadable normal fat butter, dairy-based normal fat spread (including cholesterol lowering spread)
bread_crisp_spread_polymarg_chol_med	Vegetable spread	Olive oil based spread, plant-based soft or hard margarine and soya-based spread (including cholesterol lowering spread)
bread_crisp_spread_polymarg_chol_thick	Vegetable spread	Olive oil based spread, plant-based soft or hard margarine and soya-based spread (including cholesterol lowering spread)
bread_crisp_spread_polymarg_chol_thin	Vegetable spread	Olive oil based spread, plant-based soft or hard margarine and soya-based spread (including cholesterol lowering spread)
bread_crisp_spread_polymarg_dunno_med	Vegetable spread	Olive oil based spread, plant-based soft or hard margarine and soya-based spread (including cholesterol lowering spread)
bread_crisp_spread_polymarg_dunno_thick	Vegetable spread	Olive oil based spread, plant-based soft or hard margarine and soya-based spread (including cholesterol lowering spread)
bread_crisp_spread_polymarg_dunno_thin	Vegetable spread	Olive oil based spread, plant-based soft or hard margarine and soya-based spread (including cholesterol lowering spread)
bread_crisp_spread_polymarg_fat_med	Vegetable spread	Olive oil based spread, plant-based soft or hard margarine and soya-based spread (including cholesterol lowering spread)
bread_crisp_spread_polymarg_fat_thick	Vegetable spread	Olive oil based spread, plant-based soft or hard margarine and soya-based spread (including cholesterol lowering spread)
bread_crisp_spread_polymarg_fat_thin	Vegetable spread	Olive oil based spread, plant-based soft or hard margarine and soya-based spread (including cholesterol lowering spread)
bread_crisp_spread_polymarg_lowfat_med	Vegetable spread lower fat	Olive oil based lower fat spread, plant-based lower fat margarine and soya-based lower fat spread (including cholesterol lowering spread)
bread_crisp_spread_polymarg_lowfat_thick	Vegetable spread lower fat	Olive oil based lower fat spread, plant-based lower fat margarine and soya-based lower fat spread (including cholesterol lowering spread)
bread_crisp_spread_polymarg_lowfat_thin	Vegetable spread lower fat	Olive oil based lower fat spread, plant-based lower fat margarine and soya-based lower fat spread (including cholesterol lowering spread)
bread_crisp_spread_polymarg_vlowfat_med	Vegetable spread lower fat	Olive oil based lower fat spread, plant-based lower fat margarine and soya-based lower fat spread (including cholesterol lowering spread)
bread_crisp_spread_polymarg_vlowfat_thick	Vegetable spread lower fat	Olive oil based lower fat spread, plant-based lower fat margarine and soya-based lower fat spread (including cholesterol lowering spread)
bread_crisp_spread_polymarg_vlowfat_thin	Vegetable spread lower fat	Olive oil based lower fat spread, plant-based lower fat margarine and soya-based lower fat spread (including cholesterol lowering spread)
bread_crisp_spread_soya_chol_med	Vegetable spread	Olive oil based spread, plant-based soft or hard margarine and soya-based spread (including cholesterol lowering spread)
bread_crisp_spread_soya_chol_thick	Vegetable spread	Olive oil based spread, plant-based soft or hard margarine and soya-based spread (including cholesterol lowering spread)
bread_crisp_spread_soya_chol_thin	Vegetable spread	Olive oil based spread, plant-based soft or hard margarine and soya-based spread (including cholesterol lowering spread)
bread_crisp_spread_soya_dunno_med	Vegetable spread	Olive oil based spread, plant-based soft or hard margarine and soya-based spread (including cholesterol lowering spread)
bread_crisp_spread_soya_dunno_thick	Vegetable spread	Olive oil based spread, plant-based soft or hard margarine and soya-based spread (including cholesterol lowering spread)
bread_crisp_spread_soya_dunno_thin	Vegetable spread	Olive oil based spread, plant-based soft or hard margarine and soya-based spread (including cholesterol lowering spread)
bread_crisp_spread_soya_fat_med	Vegetable spread	Olive oil based spread, plant-based soft or hard margarine and soya-based spread (including cholesterol lowering spread)
bread_crisp_spread_soya_fat_thick	Vegetable spread	Olive oil based spread, plant-based soft or hard margarine and soya-based spread (including cholesterol lowering spread)
bread_crisp_spread_soya_fat_thin	Vegetable spread	Olive oil based spread, plant-based soft or hard margarine and soya-based spread (including cholesterol lowering spread)
bread_crisp_spread_soya_lowfat_med	Vegetable spread lower fat	Olive oil based lower fat spread, plant-based lower fat margarine and soya-based lower fat spread (including cholesterol lowering spread)
bread_crisp_spread_soya_lowfat_thick	Vegetable spread lower fat	Olive oil based lower fat spread, plant-based lower fat margarine and soya-based lower fat spread (including cholesterol lowering spread)
bread_crisp_spread_soya_lowfat_thin	Vegetable spread lower fat	Olive oil based lower fat spread, plant-based lower fat margarine and soya-based lower fat spread (including cholesterol lowering spread)
bread_crisp_spread_soya_vlowfat_med	Vegetable spread lower fat	Olive oil based lower fat spread, plant-based lower fat margarine and soya-based lower fat spread (including cholesterol lowering spread)
bread_crisp_spread_soya_vlowfat_thick	Vegetable spread lower fat	Olive oil based lower fat spread, plant-based lower fat margarine and soya-based lower fat spread (including cholesterol lowering spread)
bread_crisp_spread_soya_vlowfat_thin	Vegetable spread lower fat	Olive oil based lower fat spread, plant-based lower fat margarine and soya-based lower fat spread (including cholesterol lowering spread)
bread_garlic	Other bread	Naan, garlic bread, other bread (including gluten free)
bread_large_bap_gf_nonwhite	Other bread	Naan, garlic bread, other bread (including gluten free)
bread_large_bap_gf_unanswered	White bread	White bread, sliced, baguette, bap, roll
bread_large_bap_gf_white	White bread	White bread, sliced, baguette, bap, roll
bread_large_bap_mixed	Mixed bread, brown & seeded	Mixed, brown or seeded bread, sliced, baguette, bap, roll
bread_large_bap_other	Mixed bread, brown & seeded	Mixed, brown or seeded bread, sliced, baguette, bap, roll
bread_large_bap_seeded	Unsalted nuts & seeds	Unsalted peanuts and nuts
bread_large_bap_spread_butter_dunno_med	Dairy fat spread	Spreadable normal fat butter, dairy-based normal fat spread (including cholesterol lowering spread)
bread_large_bap_spread_butter_dunno_thick	Dairy fat spread	Spreadable normal fat butter, dairy-based normal fat spread (including cholesterol lowering spread)
bread_large_bap_spread_butter_dunno_thin	Dairy fat spread	Spreadable normal fat butter, dairy-based normal fat spread (including cholesterol lowering spread)
bread_large_bap_spread_butter_fat_med	Dairy fat spread	Spreadable normal fat butter, dairy-based normal fat spread (including cholesterol lowering spread)
bread_large_bap_spread_butter_fat_thick	Dairy fat spread	Spreadable normal fat butter, dairy-based normal fat spread (including cholesterol lowering spread)
bread_large_bap_spread_butter_fat_thin	Dairy fat spread	Spreadable normal fat butter, dairy-based normal fat spread (including cholesterol lowering spread)
bread_large_bap_spread_butter_lowfat_med	Dairy fat spread lower fat	Spreadable/lower fat butter, dairy-based very low fat spread
bread_large_bap_spread_butter_lowfat_thick	Dairy fat spread lower fat	Spreadable/lower fat butter, dairy-based very low fat spread
bread_large_bap_spread_butter_lowfat_thin	Dairy fat spread lower fat	Spreadable/lower fat butter, dairy-based very low fat spread
bread_large_bap_spread_butter_spread_fat_med	Dairy fat spread	Spreadable normal fat butter, dairy-based normal fat spread (including cholesterol lowering spread)
bread_large_bap_spread_butter_spread_fat_thick	Dairy fat spread	Spreadable normal fat butter, dairy-based normal fat spread (including cholesterol lowering spread)
bread_large_bap_spread_butter_spread_fat_thin	Dairy fat spread	Spreadable normal fat butter, dairy-based normal fat spread (including cholesterol lowering spread)
bread_large_bap_spread_butter_spread_lowfat_med	Dairy fat spread lower fat	Spreadable/lower fat butter, dairy-based very low fat spread
bread_large_bap_spread_butter_spread_lowfat_thick	Dairy fat spread lower fat	Spreadable/lower fat butter, dairy-based very low fat spread
bread_large_bap_spread_butter_spread_lowfat_thin	Dairy fat spread lower fat	Spreadable/lower fat butter, dairy-based very low fat spread
bread_large_bap_spread_dairy_chol_med	Dairy fat spread	Spreadable normal fat butter, dairy-based normal fat spread (including cholesterol lowering spread)
bread_large_bap_spread_dairy_chol_thick	Dairy fat spread	Spreadable normal fat butter, dairy-based normal fat spread (including cholesterol lowering spread)
bread_large_bap_spread_dairy_chol_thin	Dairy fat spread	Spreadable normal fat butter, dairy-based normal fat spread (including cholesterol lowering spread)
bread_large_bap_spread_dairy_dunno_med	Dairy fat spread	Spreadable normal fat butter, dairy-based normal fat spread (including cholesterol lowering spread)
bread_large_bap_spread_dairy_dunno_thick	Dairy fat spread	Spreadable normal fat butter, dairy-based normal fat spread (including cholesterol lowering spread)
bread_large_bap_spread_dairy_dunno_thin	Dairy fat spread	Spreadable normal fat butter, dairy-based normal fat spread (including cholesterol lowering spread)
bread_large_bap_spread_dairy_fat_med	Dairy fat spread	Spreadable normal fat butter, dairy-based normal fat spread (including cholesterol lowering spread)
bread_large_bap_spread_dairy_fat_thick	Dairy fat spread	Spreadable normal fat butter, dairy-based normal fat spread (including cholesterol lowering spread)
bread_large_bap_spread_dairy_fat_thin	Dairy fat spread	Spreadable normal fat butter, dairy-based normal fat spread (including cholesterol lowering spread)
bread_large_bap_spread_dairy_lowfat_med	Dairy fat spread lower fat	Spreadable/lower fat butter, dairy-based very low fat spread
bread_large_bap_spread_dairy_lowfat_thick	Dairy fat spread lower fat	Spreadable/lower fat butter, dairy-based very low fat spread
bread_large_bap_spread_dairy_lowfat_thin	Dairy fat spread lower fat	Spreadable/lower fat butter, dairy-based very low fat spread
bread_large_bap_spread_dairy_vlowfat_med	Dairy fat spread lower fat	Spreadable/lower fat butter, dairy-based very low fat spread
bread_large_bap_spread_dairy_vlowfat_thick	Dairy fat spread lower fat	Spreadable/lower fat butter, dairy-based very low fat spread
bread_large_bap_spread_dairy_vlowfat_thin	Dairy fat spread lower fat	Spreadable/lower fat butter, dairy-based very low fat spread
bread_large_bap_spread_dunno_chol_med	Vegetable spread lower fat	Olive oil based lower fat spread, plant-based lower fat margarine and soya-based lower fat spread (including cholesterol lowering spread)
bread_large_bap_spread_dunno_chol_thick	Vegetable spread lower fat	Olive oil based lower fat spread, plant-based lower fat margarine and soya-based lower fat spread (including cholesterol lowering spread)
bread_large_bap_spread_dunno_chol_thin	Vegetable spread lower fat	Olive oil based lower fat spread, plant-based lower fat margarine and soya-based lower fat spread (including cholesterol lowering spread)
bread_large_bap_spread_dunno_dunno_med	Vegetable spread	Olive oil based spread, plant-based soft or hard margarine and soya-based spread (including cholesterol lowering spread)
bread_large_bap_spread_dunno_dunno_thick	Vegetable spread	Olive oil based spread, plant-based soft or hard margarine and soya-based spread (including cholesterol lowering spread)
bread_large_bap_spread_dunno_dunno_thin	Vegetable spread	Olive oil based spread, plant-based soft or hard margarine and soya-based spread (including cholesterol lowering spread)
bread_large_bap_spread_dunno_fat_med	Vegetable spread	Olive oil based spread, plant-based soft or hard margarine and soya-based spread (including cholesterol lowering spread)
bread_large_bap_spread_dunno_fat_thick	Vegetable spread	Olive oil based spread, plant-based soft or hard margarine and soya-based spread (including cholesterol lowering spread)
bread_large_bap_spread_dunno_fat_thin	Vegetable spread	Olive oil based spread, plant-based soft or hard margarine and soya-based spread (including cholesterol lowering spread)
bread_large_bap_spread_dunno_lowfat_med	Vegetable spread lower fat	Olive oil based lower fat spread, plant-based lower fat margarine and soya-based lower fat spread (including cholesterol lowering spread)
bread_large_bap_spread_dunno_lowfat_thick	Vegetable spread lower fat	Olive oil based lower fat spread, plant-based lower fat margarine and soya-based lower fat spread (including cholesterol lowering spread)
bread_large_bap_spread_dunno_lowfat_thin	Vegetable spread lower fat	Olive oil based lower fat spread, plant-based lower fat margarine and soya-based lower fat spread (including cholesterol lowering spread)
bread_large_bap_spread_dunno_vlowfat_med	Vegetable spread lower fat	Olive oil based lower fat spread, plant-based lower fat margarine and soya-based lower fat spread (including cholesterol lowering spread)
bread_large_bap_spread_dunno_vlowfat_thick	Vegetable spread lower fat	Olive oil based lower fat spread, plant-based lower fat margarine and soya-based lower fat spread (including cholesterol lowering spread)
bread_large_bap_spread_dunno_vlowfat_thin	Vegetable spread lower fat	Olive oil based lower fat spread, plant-based lower fat margarine and soya-based lower fat spread (including cholesterol lowering spread)
bread_large_bap_spread_hardmarg_med	Vegetable spread	Olive oil based spread, plant-based soft or hard margarine and soya-based spread (including cholesterol lowering spread)
bread_large_bap_spread_hardmarg_thick	Vegetable spread	Olive oil based spread, plant-based soft or hard margarine and soya-based spread (including cholesterol lowering spread)
bread_large_bap_spread_hardmarg_thin	Vegetable spread	Olive oil based spread, plant-based soft or hard margarine and soya-based spread (including cholesterol lowering spread)
bread_large_bap_spread_olive_chol_med	Vegetable spread	Olive oil based spread, plant-based soft or hard margarine and soya-based spread (including cholesterol lowering spread)
bread_large_bap_spread_olive_chol_thick	Vegetable spread	Olive oil based spread, plant-based soft or hard margarine and soya-based spread (including cholesterol lowering spread)
bread_large_bap_spread_olive_chol_thin	Vegetable spread	Olive oil based spread, plant-based soft or hard margarine and soya-based spread (including cholesterol lowering spread)
bread_large_bap_spread_olive_dunno_med	Vegetable spread	Olive oil based spread, plant-based soft or hard margarine and soya-based spread (including cholesterol lowering spread)
bread_large_bap_spread_olive_dunno_thick	Vegetable spread	Olive oil based spread, plant-based soft or hard margarine and soya-based spread (including cholesterol lowering spread)
bread_large_bap_spread_olive_dunno_thin	Vegetable spread	Olive oil based spread, plant-based soft or hard margarine and soya-based spread (including cholesterol lowering spread)
bread_large_bap_spread_olive_fat_med	Vegetable spread	Olive oil based spread, plant-based soft or hard margarine and soya-based spread (including cholesterol lowering spread)
bread_large_bap_spread_olive_fat_thick	Vegetable spread	Olive oil based spread, plant-based soft or hard margarine and soya-based spread (including cholesterol lowering spread)
bread_large_bap_spread_olive_fat_thin	Vegetable spread	Olive oil based spread, plant-based soft or hard margarine and soya-based spread (including cholesterol lowering spread)
bread_large_bap_spread_olive_lowfat_med	Vegetable spread lower fat	Olive oil based lower fat spread, plant-based lower fat margarine and soya-based lower fat spread (including cholesterol lowering spread)
bread_large_bap_spread_olive_lowfat_thick	Vegetable spread lower fat	Olive oil based lower fat spread, plant-based lower fat margarine and soya-based lower fat spread (including cholesterol lowering spread)
bread_large_bap_spread_olive_lowfat_thin	Vegetable spread lower fat	Olive oil based lower fat spread, plant-based lower fat margarine and soya-based lower fat spread (including cholesterol lowering spread)
bread_large_bap_spread_olive_vlowfat_med	Vegetable spread lower fat	Olive oil based lower fat spread, plant-based lower fat margarine and soya-based lower fat spread (including cholesterol lowering spread)
bread_large_bap_spread_olive_vlowfat_thick	Vegetable spread lower fat	Olive oil based lower fat spread, plant-based lower fat margarine and soya-based lower fat spread (including cholesterol lowering spread)
bread_large_bap_spread_olive_vlowfat_thin	Vegetable spread lower fat	Olive oil based lower fat spread, plant-based lower fat margarine and soya-based lower fat spread (including cholesterol lowering spread)
bread_large_bap_spread_other_med	Dairy fat spread	Spreadable normal fat butter, dairy-based normal fat spread (including cholesterol lowering spread)
bread_large_bap_spread_other_thick	Dairy fat spread	Spreadable normal fat butter, dairy-based normal fat spread (including cholesterol lowering spread)
bread_large_bap_spread_other_thin	Dairy fat spread	Spreadable normal fat butter, dairy-based normal fat spread (including cholesterol lowering spread)
bread_large_bap_spread_polymarg_chol_med	Vegetable spread	Olive oil based spread, plant-based soft or hard margarine and soya-based spread (including cholesterol lowering spread)
bread_large_bap_spread_polymarg_chol_thick	Vegetable spread	Olive oil based spread, plant-based soft or hard margarine and soya-based spread (including cholesterol lowering spread)
bread_large_bap_spread_polymarg_chol_thin	Vegetable spread	Olive oil based spread, plant-based soft or hard margarine and soya-based spread (including cholesterol lowering spread)
bread_large_bap_spread_polymarg_dunno_med	Vegetable spread	Olive oil based spread, plant-based soft or hard margarine and soya-based spread (including cholesterol lowering spread)
bread_large_bap_spread_polymarg_dunno_thick	Vegetable spread	Olive oil based spread, plant-based soft or hard margarine and soya-based spread (including cholesterol lowering spread)

bread_large_bap_spread_polymarg_dunno_thin	Vegetable spread	Olive oil based spread, plant-based soft or hard margarine and soya-based spread (including cholesterol lowering spread)
bread_large_bap_spread_polymarg_fat_med	Vegetable spread	Olive oil based spread, plant-based soft or hard margarine and soya-based spread (including cholesterol lowering spread)
bread_large_bap_spread_polymarg_fat_thick	Vegetable spread	Olive oil based spread, plant-based soft or hard margarine and soya-based spread (including cholesterol lowering spread)
bread_large_bap_spread_polymarg_fat_thin	Vegetable spread	Olive oil based spread, plant-based soft or hard margarine and soya-based spread (including cholesterol lowering spread)
bread_large_bap_spread_polymarg_lowfat_med	Vegetable spread lower fat	Olive oil based lower fat spread, plant-based lower fat margarine and soya-based lower fat spread (including cholesterol lowering spread)
bread_large_bap_spread_polymarg_lowfat_thick	Vegetable spread lower fat	Olive oil based lower fat spread, plant-based lower fat margarine and soya-based lower fat spread (including cholesterol lowering spread)
bread_large_bap_spread_polymarg_lowfat_thin	Vegetable spread lower fat	Olive oil based lower fat spread, plant-based lower fat margarine and soya-based lower fat spread (including cholesterol lowering spread)
bread_large_bap_spread_polymarg_vlowfat_med	Vegetable spread lower fat	Olive oil based lower fat spread, plant-based lower fat margarine and soya-based lower fat spread (including cholesterol lowering spread)
bread_large_bap_spread_polymarg_vlowfat_thick	Vegetable spread lower fat	Olive oil based lower fat spread, plant-based lower fat margarine and soya-based lower fat spread (including cholesterol lowering spread)
bread_large_bap_spread_polymarg_vlowfat_thin	Vegetable spread lower fat	Olive oil based lower fat spread, plant-based lower fat margarine and soya-based lower fat spread (including cholesterol lowering spread)
bread_large_bap_spread_soya_chol_med	Vegetable spread	Olive oil based spread, plant-based soft or hard margarine and soya-based spread (including cholesterol lowering spread)
bread_large_bap_spread_soya_chol_thick	Vegetable spread	Olive oil based spread, plant-based soft or hard margarine and soya-based spread (including cholesterol lowering spread)
bread_large_bap_spread_soya_chol_thin	Vegetable spread	Olive oil based spread, plant-based soft or hard margarine and soya-based spread (including cholesterol lowering spread)
bread_large_bap_spread_soya_dunno_med	Vegetable spread	Olive oil based spread, plant-based soft or hard margarine and soya-based spread (including cholesterol lowering spread)
bread_large_bap_spread_soya_dunno_thick	Vegetable spread	Olive oil based spread, plant-based soft or hard margarine and soya-based spread (including cholesterol lowering spread)
bread_large_bap_spread_soya_dunno_thin	Vegetable spread	Olive oil based spread, plant-based soft or hard margarine and soya-based spread (including cholesterol lowering spread)
bread_large_bap_spread_soya_fat_med	Vegetable spread	Olive oil based spread, plant-based soft or hard margarine and soya-based spread (including cholesterol lowering spread)
bread_large_bap_spread_soya_fat_thick	Vegetable spread	Olive oil based spread, plant-based soft or hard margarine and soya-based spread (including cholesterol lowering spread)
bread_large_bap_spread_soya_fat_thin	Vegetable spread	Olive oil based spread, plant-based soft or hard margarine and soya-based spread (including cholesterol lowering spread)
bread_large_bap_spread_soya_lowfat_med	Vegetable spread lower fat	Olive oil based lower fat spread, plant-based lower fat margarine and soya-based lower fat spread (including cholesterol lowering spread)
bread_large_bap_spread_soya_lowfat_thick	Vegetable spread lower fat	Olive oil based lower fat spread, plant-based lower fat margarine and soya-based lower fat spread (including cholesterol lowering spread)
bread_large_bap_spread_soya_lowfat_thin	Vegetable spread lower fat	Olive oil based lower fat spread, plant-based lower fat margarine and soya-based lower fat spread (including cholesterol lowering spread)
bread_large_bap_spread_soya_vlowfat_med	Vegetable spread lower fat	Olive oil based lower fat spread, plant-based lower fat margarine and soya-based lower fat spread (including cholesterol lowering spread)
bread_large_bap_spread_soya_vlowfat_thick	Vegetable spread lower fat	Olive oil based lower fat spread, plant-based lower fat margarine and soya-based lower fat spread (including cholesterol lowering spread)
bread_large_bap_spread_soya_vlowfat_thin	Vegetable spread lower fat	Olive oil based lower fat spread, plant-based lower fat margarine and soya-based lower fat spread (including cholesterol lowering spread)
bread_large_bap_unanswered	White bread	White bread, sliced, baguette, bap, roll
bread_large_bap_white	White bread	White bread, sliced, baguette, bap, roll
bread_large_bap_wholemeal	Wholemeal bread	Wholemeal bread, sliced, baguette, bap, roll
bread_naan	Other bread	Naan, garlic bread, other bread (including gluten free)
bread_other	Other bread	Naan, garlic bread, other bread (including gluten free)
bread_other_gf	Other bread	Naan, garlic bread, other bread (including gluten free)
bread_other_spread_butter_dunno_med	Dairy fat spread	Spreadable normal fat butter, dairy-based normal fat spread (including cholesterol lowering spread)
bread_other_spread_butter_dunno_thick	Dairy fat spread	Spreadable normal fat butter, dairy-based normal fat spread (including cholesterol lowering spread)
bread_other_spread_butter_dunno_thin	Dairy fat spread	Spreadable normal fat butter, dairy-based normal fat spread (including cholesterol lowering spread)
bread_other_spread_butter_fat_med	Dairy fat spread	Spreadable normal fat butter, dairy-based normal fat spread (including cholesterol lowering spread)
bread_other_spread_butter_fat_thick	Dairy fat spread	Spreadable normal fat butter, dairy-based normal fat spread (including cholesterol lowering spread)
bread_other_spread_butter_fat_thin	Dairy fat spread	Spreadable normal fat butter, dairy-based normal fat spread (including cholesterol lowering spread)
bread_other_spread_butter_lowfat_med	Dairy fat spread lower fat	Spreadable/lower fat butter, dairy-based very low fat spread
bread_other_spread_butter_lowfat_thick	Dairy fat spread lower fat	Spreadable/lower fat butter, dairy-based very low fat spread
bread_other_spread_butter_lowfat_thin	Dairy fat spread lower fat	Spreadable/lower fat butter, dairy-based very low fat spread
bread_other_spread_butter_spread_fat_med	Dairy fat spread	Spreadable normal fat butter, dairy-based normal fat spread (including cholesterol lowering spread)
bread_other_spread_butter_spread_fat_thick	Dairy fat spread	Spreadable normal fat butter, dairy-based normal fat spread (including cholesterol lowering spread)
bread_other_spread_butter_spread_fat_thin	Dairy fat spread	Spreadable normal fat butter, dairy-based normal fat spread (including cholesterol lowering spread)
bread_other_spread_butter_spread_lowfat_med	Dairy fat spread lower fat	Spreadable/lower fat butter, dairy-based very low fat spread
bread_other_spread_butter_spread_lowfat_thick	Dairy fat spread lower fat	Spreadable/lower fat butter, dairy-based very low fat spread
bread_other_spread_butter_spread_lowfat_thin	Dairy fat spread lower fat	Spreadable/lower fat butter, dairy-based very low fat spread
bread_other_spread_dairy_chol_med	Dairy fat spread	Spreadable normal fat butter, dairy-based normal fat spread (including cholesterol lowering spread)
bread_other_spread_dairy_chol_thick	Dairy fat spread	Spreadable normal fat butter, dairy-based normal fat spread (including cholesterol lowering spread)
bread_other_spread_dairy_chol_thin	Dairy fat spread	Spreadable normal fat butter, dairy-based normal fat spread (including cholesterol lowering spread)
bread_other_spread_dairy_dunno_med	Dairy fat spread	Spreadable normal fat butter, dairy-based normal fat spread (including cholesterol lowering spread)
bread_other_spread_dairy_dunno_thick	Dairy fat spread	Spreadable normal fat butter, dairy-based normal fat spread (including cholesterol lowering spread)
bread_other_spread_dairy_dunno_thin	Dairy fat spread	Spreadable normal fat butter, dairy-based normal fat spread (including cholesterol lowering spread)
bread_other_spread_dairy_fat_med	Dairy fat spread	Spreadable normal fat butter, dairy-based normal fat spread (including cholesterol lowering spread)
bread_other_spread_dairy_fat_thick	Dairy fat spread	Spreadable normal fat butter, dairy-based normal fat spread (including cholesterol lowering spread)
bread_other_spread_dairy_fat_thin	Dairy fat spread	Spreadable normal fat butter, dairy-based normal fat spread (including cholesterol lowering spread)
bread_other_spread_dairy_lowfat_med	Dairy fat spread lower fat	Spreadable/lower fat butter, dairy-based very low fat spread
bread_other_spread_dairy_lowfat_thick	Dairy fat spread lower fat	Spreadable/lower fat butter, dairy-based very low fat spread
bread_other_spread_dairy_lowfat_thin	Dairy fat spread lower fat	Spreadable/lower fat butter, dairy-based very low fat spread
bread_other_spread_dairy_vlowfat_med	Dairy fat spread lower fat	Spreadable/lower fat butter, dairy-based very low fat spread
bread_other_spread_dairy_vlowfat_thick	Dairy fat spread lower fat	Spreadable/lower fat butter, dairy-based very low fat spread
bread_other_spread_dairy_vlowfat_thin	Dairy fat spread lower fat	Spreadable/lower fat butter, dairy-based very low fat spread
bread_other_spread_dunno_chol_med	Vegetable spread lower fat	Olive oil based lower fat spread, plant-based lower fat margarine and soya-based lower fat spread (including cholesterol lowering spread)
bread_other_spread_dunno_chol_thick	Vegetable spread lower fat	Olive oil based lower fat spread, plant-based lower fat margarine and soya-based lower fat spread (including cholesterol lowering spread)
bread_other_spread_dunno_chol_thin	Vegetable spread lower fat	Olive oil based lower fat spread, plant-based lower fat margarine and soya-based lower fat spread (including cholesterol lowering spread)
bread_other_spread_dunno_dunno_med	Vegetable spread	Olive oil based spread, plant-based soft or hard margarine and soya-based spread (including cholesterol lowering spread)
bread_other_spread_dunno_dunno_thick	Vegetable spread	Olive oil based spread, plant-based soft or hard margarine and soya-based spread (including cholesterol lowering spread)
bread_other_spread_dunno_dunno_thin	Vegetable spread	Olive oil based spread, plant-based soft or hard margarine and soya-based spread (including cholesterol lowering spread)
bread_other_spread_dunno_fat_med	Vegetable spread	Olive oil based spread, plant-based soft or hard margarine and soya-based spread (including cholesterol lowering spread)
bread_other_spread_dunno_fat_thick	Vegetable spread	Olive oil based spread, plant-based soft or hard margarine and soya-based spread (including cholesterol lowering spread)
bread_other_spread_dunno_fat_thin	Vegetable spread	Olive oil based spread, plant-based soft or hard margarine and soya-based spread (including cholesterol lowering spread)
bread_other_spread_dunno_lowfat_med	Vegetable spread lower fat	Olive oil based lower fat spread, plant-based lower fat margarine and soya-based lower fat spread (including cholesterol lowering spread)
bread_other_spread_dunno_lowfat_thick	Vegetable spread lower fat	Olive oil based lower fat spread, plant-based lower fat margarine and soya-based lower fat spread (including cholesterol lowering spread)
bread_other_spread_dunno_lowfat_thin	Vegetable spread lower fat	Olive oil based lower fat spread, plant-based lower fat margarine and soya-based lower fat spread (including cholesterol lowering spread)
bread_other_spread_dunno_vlowfat_med	Vegetable spread lower fat	Olive oil based lower fat spread, plant-based lower fat margarine and soya-based lower fat spread (including cholesterol lowering spread)
bread_other_spread_dunno_vlowfat_thick	Vegetable spread lower fat	Olive oil based lower fat spread, plant-based lower fat margarine and soya-based lower fat spread (including cholesterol lowering spread)
bread_other_spread_dunno_vlowfat_thin	Vegetable spread lower fat	Olive oil based lower fat spread, plant-based lower fat margarine and soya-based lower fat spread (including cholesterol lowering spread)
bread_other_spread_hardmarg_med	Vegetable spread	Olive oil based spread, plant-based soft or hard margarine and soya-based spread (including cholesterol lowering spread)
bread_other_spread_hardmarg_thick	Vegetable spread	Olive oil based spread, plant-based soft or hard margarine and soya-based spread (including cholesterol lowering spread)
bread_other_spread_hardmarg_thin	Vegetable spread	Olive oil based spread, plant-based soft or hard margarine and soya-based spread (including cholesterol lowering spread)
bread_other_spread_olive_chol_med	Vegetable spread	Olive oil based spread, plant-based soft or hard margarine and soya-based spread (including cholesterol lowering spread)
bread_other_spread_olive_chol_thick	Vegetable spread	Olive oil based spread, plant-based soft or hard margarine and soya-based spread (including cholesterol lowering spread)
bread_other_spread_olive_chol_thin	Vegetable spread	Olive oil based spread, plant-based soft or hard margarine and soya-based spread (including cholesterol lowering spread)
bread_other_spread_olive_dunno_med	Vegetable spread	Olive oil based spread, plant-based soft or hard margarine and soya-based spread (including cholesterol lowering spread)
bread_other_spread_olive_dunno_thick	Vegetable spread	Olive oil based spread, plant-based soft or hard margarine and soya-based spread (including cholesterol lowering spread)
bread_other_spread_olive_dunno_thin	Vegetable spread	Olive oil based spread, plant-based soft or hard margarine and soya-based spread (including cholesterol lowering spread)
bread_other_spread_olive_fat_med	Vegetable spread	Olive oil based spread, plant-based soft or hard margarine and soya-based spread (including cholesterol lowering spread)
bread_other_spread_olive_fat_thick	Vegetable spread	Olive oil based spread, plant-based soft or hard margarine and soya-based spread (including cholesterol lowering spread)
bread_other_spread_olive_fat_thin	Vegetable spread	Olive oil based spread, plant-based soft or hard margarine and soya-based spread (including cholesterol lowering spread)
bread_other_spread_olive_lowfat_med	Vegetable spread lower fat	Olive oil based lower fat spread, plant-based lower fat margarine and soya-based lower fat spread (including cholesterol lowering spread)
bread_other_spread_olive_lowfat_thick	Vegetable spread lower fat	Olive oil based lower fat spread, plant-based lower fat margarine and soya-based lower fat spread (including cholesterol lowering spread)
bread_other_spread_olive_lowfat_thin	Vegetable spread lower fat	Olive oil based lower fat spread, plant-based lower fat margarine and soya-based lower fat spread (including cholesterol lowering spread)
bread_other_spread_olive_vlowfat_med	Vegetable spread lower fat	Olive oil based lower fat spread, plant-based lower fat margarine and soya-based lower fat spread (including cholesterol lowering spread)
bread_other_spread_olive_vlowfat_thick	Vegetable spread lower fat	Olive oil based lower fat spread, plant-based lower fat margarine and soya-based lower fat spread (including cholesterol lowering spread)
bread_other_spread_olive_vlowfat_thin	Vegetable spread lower fat	Olive oil based lower fat spread, plant-based lower fat margarine and soya-based lower fat spread (including cholesterol lowering spread)
bread_other_spread_other_med	Dairy fat spread	Spreadable normal fat butter, dairy-based normal fat spread (including cholesterol lowering spread)
bread_other_spread_other_thick	Dairy fat spread	Spreadable normal fat butter, dairy-based normal fat spread (including cholesterol lowering spread)
bread_other_spread_other_thin	Dairy fat spread	Spreadable normal fat butter, dairy-based normal fat spread (including cholesterol lowering spread)
bread_other_spread_polymarg_chol_med	Vegetable spread	Olive oil based spread, plant-based soft or hard margarine and soya-based spread (including cholesterol lowering spread)
bread_other_spread_polymarg_chol_thick	Vegetable spread	Olive oil based spread, plant-based soft or hard margarine and soya-based spread (including cholesterol lowering spread)
bread_other_spread_polymarg_chol_thin	Vegetable spread	Olive oil based spread, plant-based soft or hard margarine and soya-based spread (including cholesterol lowering spread)
bread_other_spread_polymarg_dunno_med	Vegetable spread	Olive oil based spread, plant-based soft or hard margarine and soya-based spread (including cholesterol lowering spread)
bread_other_spread_polymarg_dunno_thick	Vegetable spread	Olive oil based spread, plant-based soft or hard margarine and soya-based spread (including cholesterol lowering spread)
bread_other_spread_polymarg_dunno_thin	Vegetable spread	Olive oil based spread, plant-based soft or hard margarine and soya-based spread (including cholesterol lowering spread)
bread_other_spread_polymarg_fat_med	Vegetable spread	Olive oil based spread, plant-based soft or hard margarine and soya-based spread (including cholesterol lowering spread)
bread_other_spread_polymarg_fat_thick	Vegetable spread	Olive oil based spread, plant-based soft or hard margarine and soya-based spread (including cholesterol lowering spread)
bread_other_spread_polymarg_fat_thin	Vegetable spread	Olive oil based spread, plant-based soft or hard margarine and soya-based spread (including cholesterol lowering spread)
bread_other_spread_polymarg_lowfat_med	Vegetable spread lower fat	Olive oil based lower fat spread, plant-based lower fat margarine and soya-based lower fat spread (including cholesterol lowering spread)
bread_other_spread_polymarg_lowfat_thick	Vegetable spread lower fat	Olive oil based lower fat spread, plant-based lower fat margarine and soya-based lower fat spread (including cholesterol lowering spread)
bread_other_spread_polymarg_lowfat_thin	Vegetable spread lower fat	Olive oil based lower fat spread, plant-based lower fat margarine and soya-based lower fat spread (including cholesterol lowering spread)
bread_other_spread_polymarg_vlowfat_med	Vegetable spread lower fat	Olive oil based lower fat spread, plant-based lower fat margarine and soya-based lower fat spread (including cholesterol lowering spread)
bread_other_spread_polymarg_vlowfat_thick	Vegetable spread lower fat	Olive oil based lower fat spread, plant-based lower fat margarine and soya-based lower fat spread (including cholesterol lowering spread)
bread_other_spread_polymarg_vlowfat_thin	Vegetable spread lower fat	Olive oil based lower fat spread, plant-based lower fat margarine and soya-based lower fat spread (including cholesterol lowering spread)
bread_other_spread_soya_chol_med	Vegetable spread	Olive oil based spread, plant-based soft or hard margarine and soya-based spread (including cholesterol lowering spread)
bread_other_spread_soya_chol_thick	Vegetable spread	Olive oil based spread, plant-based soft or hard margarine and soya-based spread (including cholesterol lowering spread)
bread_other_spread_soya_chol_thin	Vegetable spread	Olive oil based spread, plant-based soft or hard margarine and soya-based spread (including cholesterol lowering spread)
bread_other_spread_soya_dunno_med	Vegetable spread	Olive oil based spread, plant-based soft or hard margarine and soya-based spread (including cholesterol lowering spread)
bread_other_spread_soya_dunno_thick	Vegetable spread	Olive oil based spread, plant-based soft or hard margarine and soya-based spread (including cholesterol lowering spread)
bread_other_spread_soya_dunno_thin	Vegetable spread	Olive oil based spread, plant-based soft or hard margarine and soya-based spread (including cholesterol lowering spread)
bread_other_spread_soya_fat_med	Vegetable spread	Olive oil based spread, plant-based soft or hard margarine and soya-based spread (including cholesterol lowering spread)
bread_other_spread_soya_fat_thick	Vegetable spread	Olive oil based spread, plant-based soft or hard margarine and soya-based spread (including cholesterol lowering spread)
bread_other_spread_soya_fat_thin	Vegetable spread	Olive oil based spread, plant-based soft or hard margarine and soya-based spread (including cholesterol lowering spread)
bread_other_spread_soya_lowfat_med	Vegetable spread lower fat	Olive oil based lower fat spread, plant-based lower fat margarine and soya-based lower fat spread (including cholesterol lowering spread)
bread_other_spread_soya_lowfat_thick	Vegetable spread lower fat	Olive oil based lower fat spread, plant-based lower fat margarine and soya-based lower fat spread (including cholesterol lowering spread)
bread_other_spread_soya_lowfat_thin	Vegetable spread lower fat	Olive oil based lower fat spread, plant-based lower fat margarine and soya-based lower fat spread (including cholesterol lowering spread)
bread_other_spread_soya_vlowfat_med	Vegetable spread lower fat	Olive oil based lower fat spread, plant-based lower fat margarine and soya-based lower fat spread (including cholesterol lowering spread)
bread_other_spread_soya_vlowfat_thick	Vegetable spread lower fat	Olive oil based lower fat spread, plant-based lower fat margarine and soya-based lower fat spread (including cholesterol lowering spread)
bread_other_spread_soya_vlowfat_thin	Vegetable spread lower fat	Olive oil based lower fat spread, plant-based lower fat margarine and soya-based lower fat spread (including cholesterol lowering spread)
bread_roll_gf_nonwhite	Other bread	Naan, garlic bread, other bread (including gluten free)
bread_roll_gf_unanswered	White bread	White bread, sliced, baguette, bap, roll
bread_roll_gf_white	White bread	White bread, sliced, baguette, bap, roll
bread_roll_mixed	Mixed bread, brown & seeded	Mixed, brown or seeded bread, sliced, baguette, bap, roll
bread_roll_other	Mixed bread, brown & seeded	Mixed, brown or seeded bread, sliced, baguette, bap, roll
bread_roll_seeded	Unsalted nuts & seeds	Unsalted peanuts and nuts
bread_roll_spread_butter_dunno_med	Dairy fat spread	Spreadable normal fat butter, dairy-based normal fat spread (including cholesterol lowering spread)
bread_roll_spread_butter_dunno_thick	Dairy fat spread	Spreadable normal fat butter, dairy-based normal fat spread (including cholesterol lowering spread)
bread_roll_spread_butter_dunno_thin	Dairy fat spread	Spreadable normal fat butter, dairy-based normal fat spread (including cholesterol lowering spread)
bread_roll_spread_butter_fat_med	Dairy fat spread	Spreadable normal fat butter, dairy-based normal fat spread (including cholesterol lowering spread)
bread_roll_spread_butter_fat_thick	Dairy fat spread	Spreadable normal fat butter, dairy-based normal fat spread (including cholesterol lowering spread)
bread_roll_spread_butter_fat_thin	Dairy fat spread	Spreadable normal fat butter, dairy-based normal fat spread (including cholesterol lowering spread)
bread_roll_spread_butter_lowfat_med	Dairy fat spread lower fat	Spreadable/lower fat butter, dairy-based very low fat spread
bread_roll_spread_butter_lowfat_thick	Dairy fat spread lower fat	Spreadable/lower fat butter, dairy-based very low fat spread
bread_roll_spread_butter_lowfat_thin	Dairy fat spread lower fat	Spreadable/lower fat butter, dairy-based very low fat spread
bread_roll_spread_butter_spread_fat_med	Dairy fat spread	Spreadable normal fat butter, dairy-based normal fat spread (including cholesterol lowering spread)
bread_roll_spread_butter_spread_fat_thick	Dairy fat spread	Spreadable normal fat butter, dairy-based normal fat spread (including cholesterol lowering spread)
bread_roll_spread_butter_spread_fat_thin	Dairy fat spread	Spreadable normal fat butter, dairy-based normal fat spread (including cholesterol lowering spread)
bread_roll_spread_butter_spread_lowfat_med	Dairy fat spread lower fat	Spreadable/lower fat butter, dairy-based very low fat spread
bread_roll_spread_butter_spread_lowfat_thick	Dairy fat spread lower fat	Spreadable/lower fat butter, dairy-based very low fat spread
bread_roll_spread_butter_spread_lowfat_thin	Dairy fat spread lower fat	Spreadable/lower fat butter, dairy-based very low fat spread
bread_roll_spread_dairy_chol_med	Dairy fat spread	Spreadable normal fat butter, dairy-based normal fat spread (including cholesterol lowering spread)
bread_roll_spread_dairy_chol_thick	Dairy fat spread	Spreadable normal fat butter, dairy-based normal fat spread (including cholesterol lowering spread)
bread_roll_spread_dairy_chol_thin	Dairy fat spread	Spreadable normal fat butter, dairy-based normal fat spread (including cholesterol lowering spread)
bread_roll_spread_dairy_dunno_med	Dairy fat spread	Spreadable normal fat butter, dairy-based normal fat spread (including cholesterol lowering spread)

bread_roll_spread_dairy_dunno_thick	Dairy fat spread	Spreadable normal fat butter, dairy-based normal fat spread (including cholesterol lowering spread)
bread_roll_spread_dairy_dunno_thin	Dairy fat spread	Spreadable normal fat butter, dairy-based normal fat spread (including cholesterol lowering spread)
bread_roll_spread_dairy_fat_med	Dairy fat spread	Spreadable normal fat butter, dairy-based normal fat spread (including cholesterol lowering spread)
bread_roll_spread_dairy_fat_thick	Dairy fat spread	Spreadable normal fat butter, dairy-based normal fat spread (including cholesterol lowering spread)
bread_roll_spread_dairy_fat_thin	Dairy fat spread	Spreadable normal fat butter, dairy-based normal fat spread (including cholesterol lowering spread)
bread_roll_spread_dairy_lowfat_med	Dairy fat spread lower fat	Spreadable/lower fat butter, dairy-based very low fat spread
bread_roll_spread_dairy_lowfat_thick	Dairy fat spread lower fat	Spreadable/lower fat butter, dairy-based very low fat spread
bread_roll_spread_dairy_lowfat_thin	Dairy fat spread lower fat	Spreadable/lower fat butter, dairy-based very low fat spread
bread_roll_spread_dairy_vlowfat_med	Dairy fat spread lower fat	Spreadable/lower fat butter, dairy-based very low fat spread
bread_roll_spread_dairy_vlowfat_thick	Dairy fat spread lower fat	Spreadable/lower fat butter, dairy-based very low fat spread
bread_roll_spread_dairy_vlowfat_thin	Dairy fat spread lower fat	Spreadable/lower fat butter, dairy-based very low fat spread
bread_roll_spread_dunno_chol_med	Vegetable spread lower fat	Olive oil based lower fat spread, plant-based lower fat margarine and soya-based lower fat spread (including cholesterol lowering spread)
bread_roll_spread_dunno_chol_thick	Vegetable spread lower fat	Olive oil based lower fat spread, plant-based lower fat margarine and soya-based lower fat spread (including cholesterol lowering spread)
bread_roll_spread_dunno_chol_thin	Vegetable spread lower fat	Olive oil based lower fat spread, plant-based lower fat margarine and soya-based lower fat spread (including cholesterol lowering spread)
bread_roll_spread_dunno_dunno_med	Vegetable spread	Olive oil based spread, plant-based soft or hard margarine and soya-based spread (including cholesterol lowering spread)
bread_roll_spread_dunno_dunno_thick	Vegetable spread	Olive oil based spread, plant-based soft or hard margarine and soya-based spread (including cholesterol lowering spread)
bread_roll_spread_dunno_dunno_thin	Vegetable spread	Olive oil based spread, plant-based soft or hard margarine and soya-based spread (including cholesterol lowering spread)
bread_roll_spread_dunno_fat_med	Vegetable spread	Olive oil based spread, plant-based soft or hard margarine and soya-based spread (including cholesterol lowering spread)
bread_roll_spread_dunno_fat_thick	Vegetable spread	Olive oil based spread, plant-based soft or hard margarine and soya-based spread (including cholesterol lowering spread)
bread_roll_spread_dunno_fat_thin	Vegetable spread	Olive oil based spread, plant-based soft or hard margarine and soya-based spread (including cholesterol lowering spread)
bread_roll_spread_dunno_lowfat_med	Vegetable spread lower fat	Olive oil based lower fat spread, plant-based lower fat margarine and soya-based lower fat spread (including cholesterol lowering spread)
bread_roll_spread_dunno_lowfat_thick	Vegetable spread lower fat	Olive oil based lower fat spread, plant-based lower fat margarine and soya-based lower fat spread (including cholesterol lowering spread)
bread_roll_spread_dunno_lowfat_thin	Vegetable spread lower fat	Olive oil based lower fat spread, plant-based lower fat margarine and soya-based lower fat spread (including cholesterol lowering spread)
bread_roll_spread_dunno_vlowfat_med	Vegetable spread lower fat	Olive oil based lower fat spread, plant-based lower fat margarine and soya-based lower fat spread (including cholesterol lowering spread)
bread_roll_spread_dunno_vlowfat_thick	Vegetable spread lower fat	Olive oil based lower fat spread, plant-based lower fat margarine and soya-based lower fat spread (including cholesterol lowering spread)
bread_roll_spread_dunno_vlowfat_thin	Vegetable spread lower fat	Olive oil based lower fat spread, plant-based lower fat margarine and soya-based lower fat spread (including cholesterol lowering spread)
bread_roll_spread_hardmarg_med	Vegetable spread	Olive oil based spread, plant-based soft or hard margarine and soya-based spread (including cholesterol lowering spread)
bread_roll_spread_hardmarg_thick	Vegetable spread	Olive oil based spread, plant-based soft or hard margarine and soya-based spread (including cholesterol lowering spread)
bread_roll_spread_hardmarg_thin	Vegetable spread	Olive oil based spread, plant-based soft or hard margarine and soya-based spread (including cholesterol lowering spread)
bread_roll_spread_olive_chol_med	Vegetable spread	Olive oil based spread, plant-based soft or hard margarine and soya-based spread (including cholesterol lowering spread)
bread_roll_spread_olive_chol_thick	Vegetable spread	Olive oil based spread, plant-based soft or hard margarine and soya-based spread (including cholesterol lowering spread)
bread_roll_spread_olive_chol_thin	Vegetable spread	Olive oil based spread, plant-based soft or hard margarine and soya-based spread (including cholesterol lowering spread)
bread_roll_spread_olive_dunno_med	Vegetable spread	Olive oil based spread, plant-based soft or hard margarine and soya-based spread (including cholesterol lowering spread)
bread_roll_spread_olive_dunno_thick	Vegetable spread	Olive oil based spread, plant-based soft or hard margarine and soya-based spread (including cholesterol lowering spread)
bread_roll_spread_olive_dunno_thin	Vegetable spread	Olive oil based spread, plant-based soft or hard margarine and soya-based spread (including cholesterol lowering spread)
bread_roll_spread_olive_fat_med	Vegetable spread	Olive oil based spread, plant-based soft or hard margarine and soya-based spread (including cholesterol lowering spread)
bread_roll_spread_olive_fat_thick	Vegetable spread	Olive oil based spread, plant-based soft or hard margarine and soya-based spread (including cholesterol lowering spread)
bread_roll_spread_olive_fat_thin	Vegetable spread	Olive oil based spread, plant-based soft or hard margarine and soya-based spread (including cholesterol lowering spread)
bread_roll_spread_olive_lowfat_med	Vegetable spread lower fat	Olive oil based lower fat spread, plant-based lower fat margarine and soya-based lower fat spread (including cholesterol lowering spread)
bread_roll_spread_olive_lowfat_thick	Vegetable spread lower fat	Olive oil based lower fat spread, plant-based lower fat margarine and soya-based lower fat spread (including cholesterol lowering spread)
bread_roll_spread_olive_lowfat_thin	Vegetable spread lower fat	Olive oil based lower fat spread, plant-based lower fat margarine and soya-based lower fat spread (including cholesterol lowering spread)
bread_roll_spread_olive_vlowfat_med	Vegetable spread lower fat	Olive oil based lower fat spread, plant-based lower fat margarine and soya-based lower fat spread (including cholesterol lowering spread)
bread_roll_spread_olive_vlowfat_thick	Vegetable spread lower fat	Olive oil based lower fat spread, plant-based lower fat margarine and soya-based lower fat spread (including cholesterol lowering spread)
bread_roll_spread_olive_vlowfat_thin	Vegetable spread lower fat	Olive oil based lower fat spread, plant-based lower fat margarine and soya-based lower fat spread (including cholesterol lowering spread)
bread_roll_spread_other_med	Dairy fat spread	Spreadable normal fat butter, dairy-based normal fat spread (including cholesterol lowering spread)
bread_roll_spread_other_thick	Dairy fat spread	Spreadable normal fat butter, dairy-based normal fat spread (including cholesterol lowering spread)
bread_roll_spread_other_thin	Dairy fat spread	Spreadable normal fat butter, dairy-based normal fat spread (including cholesterol lowering spread)
bread_roll_spread_polymarg_chol_med	Vegetable spread	Olive oil based spread, plant-based soft or hard margarine and soya-based spread (including cholesterol lowering spread)
bread_roll_spread_polymarg_chol_thick	Vegetable spread	Olive oil based spread, plant-based soft or hard margarine and soya-based spread (including cholesterol lowering spread)
bread_roll_spread_polymarg_chol_thin	Vegetable spread	Olive oil based spread, plant-based soft or hard margarine and soya-based spread (including cholesterol lowering spread)
bread_roll_spread_polymarg_dunno_med	Vegetable spread	Olive oil based spread, plant-based soft or hard margarine and soya-based spread (including cholesterol lowering spread)
bread_roll_spread_polymarg_dunno_thick	Vegetable spread	Olive oil based spread, plant-based soft or hard margarine and soya-based spread (including cholesterol lowering spread)
bread_roll_spread_polymarg_dunno_thin	Vegetable spread	Olive oil based spread, plant-based soft or hard margarine and soya-based spread (including cholesterol lowering spread)
bread_roll_spread_polymarg_fat_med	Vegetable spread	Olive oil based spread, plant-based soft or hard margarine and soya-based spread (including cholesterol lowering spread)
bread_roll_spread_polymarg_fat_thick	Vegetable spread	Olive oil based spread, plant-based soft or hard margarine and soya-based spread (including cholesterol lowering spread)
bread_roll_spread_polymarg_fat_thin	Vegetable spread	Olive oil based spread, plant-based soft or hard margarine and soya-based spread (including cholesterol lowering spread)
bread_roll_spread_polymarg_lowfat_med	Vegetable spread lower fat	Olive oil based lower fat spread, plant-based lower fat margarine and soya-based lower fat spread (including cholesterol lowering spread)
bread_roll_spread_polymarg_lowfat_thick	Vegetable spread lower fat	Olive oil based lower fat spread, plant-based lower fat margarine and soya-based lower fat spread (including cholesterol lowering spread)
bread_roll_spread_polymarg_lowfat_thin	Vegetable spread lower fat	Olive oil based lower fat spread, plant-based lower fat margarine and soya-based lower fat spread (including cholesterol lowering spread)
bread_roll_spread_polymarg_vlowfat_med	Vegetable spread lower fat	Olive oil based lower fat spread, plant-based lower fat margarine and soya-based lower fat spread (including cholesterol lowering spread)
bread_roll_spread_polymarg_vlowfat_thick	Vegetable spread lower fat	Olive oil based lower fat spread, plant-based lower fat margarine and soya-based lower fat spread (including cholesterol lowering spread)
bread_roll_spread_polymarg_vlowfat_thin	Vegetable spread lower fat	Olive oil based lower fat spread, plant-based lower fat margarine and soya-based lower fat spread (including cholesterol lowering spread)
bread_roll_spread_soya_chol_med	Vegetable spread	Olive oil based spread, plant-based soft or hard margarine and soya-based spread (including cholesterol lowering spread)
bread_roll_spread_soya_chol_thick	Vegetable spread	Olive oil based spread, plant-based soft or hard margarine and soya-based spread (including cholesterol lowering spread)
bread_roll_spread_soya_chol_thin	Vegetable spread	Olive oil based spread, plant-based soft or hard margarine and soya-based spread (including cholesterol lowering spread)
bread_roll_spread_soya_dunno_med	Vegetable spread	Olive oil based spread, plant-based soft or hard margarine and soya-based spread (including cholesterol lowering spread)
bread_roll_spread_soya_dunno_thick	Vegetable spread	Olive oil based spread, plant-based soft or hard margarine and soya-based spread (including cholesterol lowering spread)
bread_roll_spread_soya_dunno_thin	Vegetable spread	Olive oil based spread, plant-based soft or hard margarine and soya-based spread (including cholesterol lowering spread)
bread_roll_spread_soya_fat_med	Vegetable spread	Olive oil based spread, plant-based soft or hard margarine and soya-based spread (including cholesterol lowering spread)
bread_roll_spread_soya_fat_thick	Vegetable spread	Olive oil based spread, plant-based soft or hard margarine and soya-based spread (including cholesterol lowering spread)
bread_roll_spread_soya_fat_thin	Vegetable spread	Olive oil based spread, plant-based soft or hard margarine and soya-based spread (including cholesterol lowering spread)
bread_roll_spread_soya_lowfat_med	Vegetable spread lower fat	Olive oil based lower fat spread, plant-based lower fat margarine and soya-based lower fat spread (including cholesterol lowering spread)
bread_roll_spread_soya_lowfat_thick	Vegetable spread lower fat	Olive oil based lower fat spread, plant-based lower fat margarine and soya-based lower fat spread (including cholesterol lowering spread)
bread_roll_spread_soya_lowfat_thin	Vegetable spread lower fat	Olive oil based lower fat spread, plant-based lower fat margarine and soya-based lower fat spread (including cholesterol lowering spread)
bread_roll_spread_soya_vlowfat_med	Vegetable spread lower fat	Olive oil based lower fat spread, plant-based lower fat margarine and soya-based lower fat spread (including cholesterol lowering spread)
bread_roll_spread_soya_vlowfat_thick	Vegetable spread lower fat	Olive oil based lower fat spread, plant-based lower fat margarine and soya-based lower fat spread (including cholesterol lowering spread)
bread_roll_spread_soya_vlowfat_thin	Vegetable spread lower fat	Olive oil based lower fat spread, plant-based lower fat margarine and soya-based lower fat spread (including cholesterol lowering spread)
bread_roll_unanswered	White bread	White bread, sliced, baguette, bap, roll
bread_roll_white	White bread	White bread, sliced, baguette, bap, roll
bread_roll_wholemeal	Wholemeal bread	Wholemeal bread, sliced, baguette, bap, roll
bread_sliced_gf_nonwhite	Other bread	Naan, garlic bread, other bread (including gluten free)
bread_sliced_gf_unanswered	White bread	White bread, sliced, baguette, bap, roll
bread_sliced_gf_white	White bread	White bread, sliced, baguette, bap, roll
bread_sliced_mixed	Mixed bread, brown & seeded	Mixed, brown or seeded bread, sliced, baguette, bap, roll
bread_sliced_other	Mixed bread, brown & seeded	Mixed, brown or seeded bread, sliced, baguette, bap, roll
bread_sliced_seeded	Unsalted nuts & seeds	Unsalted peanuts and nuts
bread_sliced_spread_butter_dunno_med	Dairy fat spread	Spreadable normal fat butter, dairy-based normal fat spread (including cholesterol lowering spread)
bread_sliced_spread_butter_dunno_thick	Dairy fat spread	Spreadable normal fat butter, dairy-based normal fat spread (including cholesterol lowering spread)
bread_sliced_spread_butter_dunno_thin	Dairy fat spread	Spreadable normal fat butter, dairy-based normal fat spread (including cholesterol lowering spread)
bread_sliced_spread_butter_fat_med	Dairy fat spread	Spreadable normal fat butter, dairy-based normal fat spread (including cholesterol lowering spread)
bread_sliced_spread_butter_fat_thick	Dairy fat spread	Spreadable normal fat butter, dairy-based normal fat spread (including cholesterol lowering spread)
bread_sliced_spread_butter_fat_thin	Dairy fat spread	Spreadable normal fat butter, dairy-based normal fat spread (including cholesterol lowering spread)
bread_sliced_spread_butter_lowfat_med	Dairy fat spread lower fat	Spreadable/lower fat butter, dairy-based very low fat spread
bread_sliced_spread_butter_lowfat_thick	Dairy fat spread lower fat	Spreadable/lower fat butter, dairy-based very low fat spread
bread_sliced_spread_butter_lowfat_thin	Dairy fat spread lower fat	Spreadable/lower fat butter, dairy-based very low fat spread
bread_sliced_spread_butter_spread_fat_med	Dairy fat spread	Spreadable normal fat butter, dairy-based normal fat spread (including cholesterol lowering spread)
bread_sliced_spread_butter_spread_fat_thick	Dairy fat spread	Spreadable normal fat butter, dairy-based normal fat spread (including cholesterol lowering spread)
bread_sliced_spread_butter_spread_fat_thin	Dairy fat spread	Spreadable normal fat butter, dairy-based normal fat spread (including cholesterol lowering spread)
bread_sliced_spread_butter_spread_lowfat_med	Dairy fat spread lower fat	Spreadable/lower fat butter, dairy-based very low fat spread
bread_sliced_spread_butter_spread_lowfat_thick	Dairy fat spread lower fat	Spreadable/lower fat butter, dairy-based very low fat spread
bread_sliced_spread_butter_spread_lowfat_thin	Dairy fat spread lower fat	Spreadable/lower fat butter, dairy-based very low fat spread
bread_sliced_spread_dairy_chol_med	Dairy fat spread	Spreadable normal fat butter, dairy-based normal fat spread (including cholesterol lowering spread)
bread_sliced_spread_dairy_chol_thick	Dairy fat spread	Spreadable normal fat butter, dairy-based normal fat spread (including cholesterol lowering spread)
bread_sliced_spread_dairy_chol_thin	Dairy fat spread	Spreadable normal fat butter, dairy-based normal fat spread (including cholesterol lowering spread)
bread_sliced_spread_dairy_dunno_med	Dairy fat spread	Spreadable normal fat butter, dairy-based normal fat spread (including cholesterol lowering spread)
bread_sliced_spread_dairy_dunno_thick	Dairy fat spread	Spreadable normal fat butter, dairy-based normal fat spread (including cholesterol lowering spread)
bread_sliced_spread_dairy_dunno_thin	Dairy fat spread	Spreadable normal fat butter, dairy-based normal fat spread (including cholesterol lowering spread)
bread_sliced_spread_dairy_fat_med	Dairy fat spread	Spreadable normal fat butter, dairy-based normal fat spread (including cholesterol lowering spread)
bread_sliced_spread_dairy_fat_thick	Dairy fat spread	Spreadable normal fat butter, dairy-based normal fat spread (including cholesterol lowering spread)
bread_sliced_spread_dairy_fat_thin	Dairy fat spread	Spreadable normal fat butter, dairy-based normal fat spread (including cholesterol lowering spread)
bread_sliced_spread_dairy_lowfat_med	Dairy fat spread lower fat	Spreadable/lower fat butter, dairy-based very low fat spread
bread_sliced_spread_dairy_lowfat_thick	Dairy fat spread lower fat	Spreadable/lower fat butter, dairy-based very low fat spread
bread_sliced_spread_dairy_lowfat_thin	Dairy fat spread lower fat	Spreadable/lower fat butter, dairy-based very low fat spread
bread_sliced_spread_dairy_vlowfat_med	Dairy fat spread lower fat	Spreadable/lower fat butter, dairy-based very low fat spread
bread_sliced_spread_dairy_vlowfat_thick	Dairy fat spread lower fat	Spreadable/lower fat butter, dairy-based very low fat spread
bread_sliced_spread_dairy_vlowfat_thin	Dairy fat spread lower fat	Spreadable/lower fat butter, dairy-based very low fat spread
bread_sliced_spread_dunno_chol_med	Vegetable spread lower fat	Olive oil based lower fat spread, plant-based lower fat margarine and soya-based lower fat spread (including cholesterol lowering spread)
bread_sliced_spread_dunno_chol_thick	Vegetable spread lower fat	Olive oil based lower fat spread, plant-based lower fat margarine and soya-based lower fat spread (including cholesterol lowering spread)
bread_sliced_spread_dunno_chol_thin	Vegetable spread lower fat	Olive oil based lower fat spread, plant-based lower fat margarine and soya-based lower fat spread (including cholesterol lowering spread)
bread_sliced_spread_dunno_dunno_med	Vegetable spread	Olive oil based spread, plant-based soft or hard margarine and soya-based spread (including cholesterol lowering spread)
bread_sliced_spread_dunno_dunno_thick	Vegetable spread	Olive oil based spread, plant-based soft or hard margarine and soya-based spread (including cholesterol lowering spread)
bread_sliced_spread_dunno_dunno_thin	Vegetable spread	Olive oil based spread, plant-based soft or hard margarine and soya-based spread (including cholesterol lowering spread)
bread_sliced_spread_dunno_fat_med	Vegetable spread	Olive oil based spread, plant-based soft or hard margarine and soya-based spread (including cholesterol lowering spread)
bread_sliced_spread_dunno_fat_thick	Vegetable spread	Olive oil based spread, plant-based soft or hard margarine and soya-based spread (including cholesterol lowering spread)
bread_sliced_spread_dunno_fat_thin	Vegetable spread	Olive oil based spread, plant-based soft or hard margarine and soya-based spread (including cholesterol lowering spread)
bread_sliced_spread_dunno_lowfat_med	Vegetable spread lower fat	Olive oil based lower fat spread, plant-based lower fat margarine and soya-based lower fat spread (including cholesterol lowering spread)
bread_sliced_spread_dunno_lowfat_thick	Vegetable spread lower fat	Olive oil based lower fat spread, plant-based lower fat margarine and soya-based lower fat spread (including cholesterol lowering spread)
bread_sliced_spread_dunno_lowfat_thin	Vegetable spread lower fat	Olive oil based lower fat spread, plant-based lower fat margarine and soya-based lower fat spread (including cholesterol lowering spread)
bread_sliced_spread_dunno_vlowfat_med	Vegetable spread lower fat	Olive oil based lower fat spread, plant-based lower fat margarine and soya-based lower fat spread (including cholesterol lowering spread)
bread_sliced_spread_dunno_vlowfat_thick	Vegetable spread lower fat	Olive oil based lower fat spread, plant-based lower fat margarine and soya-based lower fat spread (including cholesterol lowering spread)
bread_sliced_spread_dunno_vlowfat_thin	Vegetable spread lower fat	Olive oil based lower fat spread, plant-based lower fat margarine and soya-based lower fat spread (including cholesterol lowering spread)
bread_sliced_spread_hardmarg_med	Vegetable spread	Olive oil based spread, plant-based soft or hard margarine and soya-based spread (including cholesterol lowering spread)
bread_sliced_spread_hardmarg_thick	Vegetable spread	Olive oil based spread, plant-based soft or hard margarine and soya-based spread (including cholesterol lowering spread)
bread_sliced_spread_hardmarg_thin	Vegetable spread	Olive oil based spread, plant-based soft or hard margarine and soya-based spread (including cholesterol lowering spread)
bread_sliced_spread_olive_chol_med	Vegetable spread	Olive oil based spread, plant-based soft or hard margarine and soya-based spread (including cholesterol lowering spread)
bread_sliced_spread_olive_chol_thick	Vegetable spread	Olive oil based spread, plant-based soft or hard margarine and soya-based spread (including cholesterol lowering spread)
bread_sliced_spread_olive_chol_thin	Vegetable spread	Olive oil based spread, plant-based soft or hard margarine and soya-based spread (including cholesterol lowering spread)
bread_sliced_spread_olive_dunno_med	Vegetable spread	Olive oil based spread, plant-based soft or hard margarine and soya-based spread (including cholesterol lowering spread)
bread_sliced_spread_olive_dunno_thick	Vegetable spread	Olive oil based spread, plant-based soft or hard margarine and soya-based spread (including cholesterol lowering spread)
bread_sliced_spread_olive_dunno_thin	Vegetable spread	Olive oil based spread, plant-based soft or hard margarine and soya-based spread (including cholesterol lowering spread)
bread_sliced_spread_olive_fat_med	Vegetable spread	Olive oil based spread, plant-based soft or hard margarine and soya-based spread (including cholesterol lowering spread)
bread_sliced_spread_olive_fat_thick	Vegetable spread	Olive oil based spread, plant-based soft or hard margarine and soya-based spread (including cholesterol lowering spread)
bread_sliced_spread_olive_fat_thin	Vegetable spread	Olive oil based spread, plant-based soft or hard margarine and soya-based spread (including cholesterol lowering spread)
bread_sliced_spread_olive_lowfat_med	Vegetable spread lower fat	Olive oil based lower fat spread, plant-based lower fat margarine and soya-based lower fat spread (including cholesterol lowering spread)
bread_sliced_spread_olive_lowfat_thick	Vegetable spread lower fat	Olive oil based lower fat spread, plant-based lower fat margarine and soya-based lower fat spread (including cholesterol lowering spread)
bread_sliced_spread_olive_lowfat_thin	Vegetable spread lower fat	Olive oil based lower fat spread, plant-based lower fat margarine and soya-based lower fat spread (including cholesterol lowering spread)
bread_sliced_spread_olive_vlowfat_med	Vegetable spread lower fat	Olive oil based lower fat spread, plant-based lower fat margarine and soya-based lower fat spread (including cholesterol lowering spread)
bread_sliced_spread_olive_vlowfat_thick	Vegetable spread lower fat	Olive oil based lower fat spread, plant-based lower fat margarine and soya-based lower fat spread (including cholesterol lowering spread)
bread_sliced_spread_olive_vlowfat_thin	Vegetable spread lower fat	Olive oil based lower fat spread, plant-based lower fat margarine and soya-based lower fat spread (including cholesterol lowering spread)
bread_sliced_spread_other_med	Dairy fat spread	Spreadable normal fat butter, dairy-based normal fat spread (including cholesterol lowering spread)
bread_sliced_spread_other_thick	Dairy fat spread	Spreadable normal fat butter, dairy-based normal fat spread (including cholesterol lowering spread)
bread_sliced_spread_other_thin	Dairy fat spread	Spreadable normal fat butter, dairy-based normal fat spread (including cholesterol lowering spread)

bread_sliced_spread_polymarg_chol_med	Vegetable spread	Olive oil based spread, plant-based soft or hard margarine and soya-based spread (including cholesterol lowering spread)
bread_sliced_spread_polymarg_chol_thick	Vegetable spread	Olive oil based spread, plant-based soft or hard margarine and soya-based spread (including cholesterol lowering spread)
bread_sliced_spread_polymarg_chol_thin	Vegetable spread	Olive oil based spread, plant-based soft or hard margarine and soya-based spread (including cholesterol lowering spread)
bread_sliced_spread_polymarg_dunno_med	Vegetable spread	Olive oil based spread, plant-based soft or hard margarine and soya-based spread (including cholesterol lowering spread)
bread_sliced_spread_polymarg_dunno_thick	Vegetable spread	Olive oil based spread, plant-based soft or hard margarine and soya-based spread (including cholesterol lowering spread)
bread_sliced_spread_polymarg_dunno_thin	Vegetable spread	Olive oil based spread, plant-based soft or hard margarine and soya-based spread (including cholesterol lowering spread)
bread_sliced_spread_polymarg_fat_med	Vegetable spread	Olive oil based spread, plant-based soft or hard margarine and soya-based spread (including cholesterol lowering spread)
bread_sliced_spread_polymarg_fat_thick	Vegetable spread	Olive oil based spread, plant-based soft or hard margarine and soya-based spread (including cholesterol lowering spread)
bread_sliced_spread_polymarg_fat_thin	Vegetable spread	Olive oil based spread, plant-based soft or hard margarine and soya-based spread (including cholesterol lowering spread)
bread_sliced_spread_polymarg_lowfat_med	Vegetable spread lower fat	Olive oil based lower fat spread, plant-based lower fat margarine and soya-based lower fat spread (including cholesterol lowering spread)
bread_sliced_spread_polymarg_lowfat_thick	Vegetable spread lower fat	Olive oil based lower fat spread, plant-based lower fat margarine and soya-based lower fat spread (including cholesterol lowering spread)
bread_sliced_spread_polymarg_lowfat_thin	Vegetable spread lower fat	Olive oil based lower fat spread, plant-based lower fat margarine and soya-based lower fat spread (including cholesterol lowering spread)
bread_sliced_spread_polymarg_vlowfat_med	Vegetable spread lower fat	Olive oil based lower fat spread, plant-based lower fat margarine and soya-based lower fat spread (including cholesterol lowering spread)
bread_sliced_spread_polymarg_vlowfat_thick	Vegetable spread lower fat	Olive oil based lower fat spread, plant-based lower fat margarine and soya-based lower fat spread (including cholesterol lowering spread)
bread_sliced_spread_polymarg_vlowfat_thin	Vegetable spread lower fat	Olive oil based lower fat spread, plant-based lower fat margarine and soya-based lower fat spread (including cholesterol lowering spread)
bread_sliced_spread_soya_chol_med	Vegetable spread	Olive oil based spread, plant-based soft or hard margarine and soya-based spread (including cholesterol lowering spread)
bread_sliced_spread_soya_chol_thick	Vegetable spread	Olive oil based spread, plant-based soft or hard margarine and soya-based spread (including cholesterol lowering spread)
bread_sliced_spread_soya_chol_thin	Vegetable spread	Olive oil based spread, plant-based soft or hard margarine and soya-based spread (including cholesterol lowering spread)
bread_sliced_spread_soya_dunno_med	Vegetable spread	Olive oil based spread, plant-based soft or hard margarine and soya-based spread (including cholesterol lowering spread)
bread_sliced_spread_soya_dunno_thick	Vegetable spread	Olive oil based spread, plant-based soft or hard margarine and soya-based spread (including cholesterol lowering spread)
bread_sliced_spread_soya_dunno_thin	Vegetable spread	Olive oil based spread, plant-based soft or hard margarine and soya-based spread (including cholesterol lowering spread)
bread_sliced_spread_soya_fat_med	Vegetable spread	Olive oil based spread, plant-based soft or hard margarine and soya-based spread (including cholesterol lowering spread)
bread_sliced_spread_soya_fat_thick	Vegetable spread	Olive oil based spread, plant-based soft or hard margarine and soya-based spread (including cholesterol lowering spread)
bread_sliced_spread_soya_fat_thin	Vegetable spread	Olive oil based spread, plant-based soft or hard margarine and soya-based spread (including cholesterol lowering spread)
bread_sliced_spread_soya_lowfat_med	Vegetable spread lower fat	Olive oil based lower fat spread, plant-based lower fat margarine and soya-based lower fat spread (including cholesterol lowering spread)
bread_sliced_spread_soya_lowfat_thick	Vegetable spread lower fat	Olive oil based lower fat spread, plant-based lower fat margarine and soya-based lower fat spread (including cholesterol lowering spread)
bread_sliced_spread_soya_lowfat_thin	Vegetable spread lower fat	Olive oil based lower fat spread, plant-based lower fat margarine and soya-based lower fat spread (including cholesterol lowering spread)
bread_sliced_spread_soya_vlowfat_med	Vegetable spread lower fat	Olive oil based lower fat spread, plant-based lower fat margarine and soya-based lower fat spread (including cholesterol lowering spread)
bread_sliced_spread_soya_vlowfat_thick	Vegetable spread lower fat	Olive oil based lower fat spread, plant-based lower fat margarine and soya-based lower fat spread (including cholesterol lowering spread)
bread_sliced_spread_soya_vlowfat_thin	Vegetable spread lower fat	Olive oil based lower fat spread, plant-based lower fat margarine and soya-based lower fat spread (including cholesterol lowering spread)
bread_sliced_unanswered	White bread	White bread, sliced, baguette, bap, roll
bread_sliced_white	White bread	White bread, sliced, baguette, bap, roll
bread_sliced_wholemeal	Wholemeal bread	Wholemeal bread, sliced, baguette, bap, roll
cake	Desserts & cakes & pastries	Pancakes, croissant, Danish pastries, scones, fruitcakes, cakes, doughnuts, sponge puddings, other desserts, cereal bars, sweet snacks
cake_gf	Desserts & cakes & pastries	Pancakes, croissant, Danish pastries, scones, fruitcakes, cakes, doughnuts, sponge puddings, other desserts, cereal bars, sweet snacks
cereal_bran	Bran cereal	Bran cereal
cereal_bran_driedfruit	Bran cereal	Bran cereal
cereal_muesli	Muesli	Muesli (with or without dried fruit)
cereal_muesli_driedfruit	Muesli	Muesli (with or without dried fruit)
cereal_oatcrunch	Oat cereal (sugar)	Oatcrunch breakfast cereal
cereal_oatcrunch_driedfruit	Oat cereal (sugar)	Oatcrunch breakfast cereal
cereal_other	Other cereal (sugar)	Plain and sugary breakfast cereal (with/without dried fruit)
cereal_other_driedfruit	Other cereal (sugar)	Plain and sugary breakfast cereal (with/without dried fruit)
cereal_plain	Other cereal (sugar)	Plain and sugary breakfast cereal (with/without dried fruit)
cereal_plain_driedfruit	Other cereal (sugar)	Plain and sugary breakfast cereal (with/without dried fruit)
cereal_porridge_milk_chol	Oat cereal (non-sugar)	Porridge oats (including milk/dried fruit added)
cereal_porridge_milk_chol_driedfruit	Oat cereal (non-sugar)	Porridge oats (including milk/dried fruit added)
cereal_porridge_milk_dontknow	Oat cereal (non-sugar)	Porridge oats (including milk/dried fruit added)
cereal_porridge_milk_dontknow_driedfruit	Oat cereal (non-sugar)	Porridge oats (including milk/dried fruit added)
cereal_porridge_milk_goatsheep	Oat cereal (non-sugar)	Porridge oats (including milk/dried fruit added)
cereal_porridge_milk_goatsheep_driedfruit	Oat cereal (non-sugar)	Porridge oats (including milk/dried fruit added)
cereal_porridge_milk_other	Oat cereal (non-sugar)	Porridge oats (including milk/dried fruit added)
cereal_porridge_milk_other_driedfruit	Oat cereal (non-sugar)	Porridge oats (including milk/dried fruit added)
cereal_porridge_milk_powdered	Oat cereal (non-sugar)	Porridge oats (including milk/dried fruit added)
cereal_porridge_milk_powdered_driedfruit	Oat cereal (non-sugar)	Porridge oats (including milk/dried fruit added)
cereal_porridge_milk_riceoatveg	Oat cereal (non-sugar)	Porridge oats (including milk/dried fruit added)
cereal_porridge_milk_riceoatveg_driedfruit	Oat cereal (non-sugar)	Porridge oats (including milk/dried fruit added)
cereal_porridge_milk_semi	Oat cereal (non-sugar)	Porridge oats (including milk/dried fruit added)
cereal_porridge_milk_semi_driedfruit	Oat cereal (non-sugar)	Porridge oats (including milk/dried fruit added)
cereal_porridge_milk_skimmed	Oat cereal (non-sugar)	Porridge oats (including milk/dried fruit added)
cereal_porridge_milk_skimmed_driedfruit	Oat cereal (non-sugar)	Porridge oats (including milk/dried fruit added)
cereal_porridge_milk_soya_ca	Oat cereal (non-sugar)	Porridge oats (including milk/dried fruit added)
cereal_porridge_milk_soya_ca_driedfruit	Oat cereal (non-sugar)	Porridge oats (including milk/dried fruit added)
cereal_porridge_milk_soya_noca	Oat cereal (non-sugar)	Porridge oats (including milk/dried fruit added)
cereal_porridge_milk_soya_noca_driedfruit	Oat cereal (non-sugar)	Porridge oats (including milk/dried fruit added)
cereal_porridge_milk_whole	Oat cereal (non-sugar)	Porridge oats (including milk/dried fruit added)
cereal_porridge_milk_whole_driedfruit	Oat cereal (non-sugar)	Porridge oats (including milk/dried fruit added)
cereal_porridge_water	Oat cereal (non-sugar)	Porridge oats (including milk/dried fruit added)
cereal_porridge_water_driedfruit	Oat cereal (non-sugar)	Porridge oats (including milk/dried fruit added)
cereal_sugar	Added sugars & preserves	Table sugar, honey, jam and preserves
cereal_sweet	Other cereal (sugar)	Plain and sugary breakfast cereal (with/without dried fruit)
cereal_sweet_driedfruit	Other cereal (sugar)	Plain and sugary breakfast cereal (with/without dried fruit)
cereal_wwheat	Biscuit cereal	Wholewheat biscuit cereal
cereal_wwheat_driedfruit	Biscuit cereal	Wholewheat biscuit cereal
cerealbar	Desserts & cakes & pastries	Pancakes, croissant, Danish pastries, scones, fruitcakes, cakes, doughnuts, sponge puddings, other desserts, cereal bars, sweet snacks
cerealbar_gf	Desserts & cakes & pastries	Pancakes, croissant, Danish pastries, scones, fruitcakes, cakes, doughnuts, sponge puddings, other desserts, cereal bars, sweet snacks
cheese_blue	High fat cheese	Cheese >17.5 g fat per 100 g, including hard cheese, soft cheese, spreadable, Blue, Feta, Mozzarella, Goats, other)
cheese_cottage	Medium & low fat cheese	Cheese <=17.5g fat per 100 g, including hard and spreadable lower fat cheese, Cottage
cheese_feta	High fat cheese	Cheese >17.5 g fat per 100 g, including hard cheese, soft cheese, spreadable, Blue, Feta, Mozzarella, Goats, other)
cheese_goat	High fat cheese	Cheese >17.5 g fat per 100 g, including hard cheese, soft cheese, spreadable, Blue, Feta, Mozzarella, Goats, other)
cheese_hard	High fat cheese	Cheese >17.5 g fat per 100 g, including hard cheese, soft cheese, spreadable, Blue, Feta, Mozzarella, Goats, other)
cheese_hard_lof	Medium & low fat cheese	Cheese <=17.5g fat per 100 g, including hard and spreadable lower fat cheese, Cottage
cheese_mozzarella	High fat cheese	Cheese >17.5 g fat per 100 g, including hard cheese, soft cheese, spreadable, Blue, Feta, Mozzarella, Goats, other)
cheese_other	High fat cheese	Cheese >17.5 g fat per 100 g, including hard cheese, soft cheese, spreadable, Blue, Feta, Mozzarella, Goats, other)
cheese_soft	High fat cheese	Cheese >17.5 g fat per 100 g, including hard cheese, soft cheese, spreadable, Blue, Feta, Mozzarella, Goats, other)
cheese_spread	High fat cheese	Cheese >17.5 g fat per 100 g, including hard cheese, soft cheese, spreadable, Blue, Feta, Mozzarella, Goats, other)
cheese_spread_lof	Medium & low fat cheese	Cheese <=17.5g fat per 100 g, including hard and spreadable lower fat cheese, Cottage
cheesecake	Milk-dairy desserts	Ice cream, milk puddings, milk-based desserts, cheesecake
cheesecake_gf	Milk-dairy desserts	Ice cream, milk puddings, milk-based desserts, cheesecake
choc_bar	Chocolate confectionery	Chocolate bar (including white, milk and dark chocolate), chocolate-covered raisins, chocolate-covered sweets
choc_dark	Chocolate confectionery	Chocolate bar (including white, milk and dark chocolate), chocolate-covered raisins, chocolate-covered sweets
choc_milk	Chocolate confectionery	Chocolate bar (including white, milk and dark chocolate), chocolate-covered raisins, chocolate-covered sweets
choc_sweets	Chocolate confectionery	Chocolate bar (including white, milk and dark chocolate), chocolate-covered raisins, chocolate-covered sweets
choc_white	Chocolate confectionery	Chocolate bar (including white, milk and dark chocolate), chocolate-covered raisins, chocolate-covered sweets
chocyog_raisin	Chocolate confectionery	Chocolate bar (including white, milk and dark chocolate), chocolate-covered raisins, chocolate-covered sweets
chutney	Sauces (lower fat)	Yeast, chutney, olives, ketchup, brown sauce, tomato sauce
cof_capp	Coffee, caffeinated	Normal instant, filter, cappuccino, espresso coffee
cof_capp_decaf	Coffee, decaffeinated	Decaffeinated instant, filter, cappuccino, espresso coffee
cof_capp_milk_chol	Skimmed milk	Skimmed milk <1 g fat per 100 g (cow, cholesterol lowering, powdered)
cof_capp_milk_dontknow	Semi-skimmed milk	Semi-skimmed milk >1 g fat per 100 g (cow, other)
cof_capp_milk_goatsheep	Whole milk	Whole milk >3.6 g fat per 100 g (cow, goat, sheep)
cof_capp_milk_other	Semi-skimmed milk	Semi-skimmed milk >1 g fat per 100 g (cow, other)
cof_capp_milk_powdered	Skimmed milk	Skimmed milk <1 g fat per 100 g (cow, cholesterol lowering, powdered)
cof_capp_milk_riceoatveg	Rice/oat drink	Rice and oat vegetable drinks
cof_capp_milk_semi	Semi-skimmed milk	Semi-skimmed milk >1 g fat per 100 g (cow, other)
cof_capp_milk_skimmed	Skimmed milk	Skimmed milk <1 g fat per 100 g (cow, cholesterol lowering, powdered)
cof_capp_milk_soya_ca	Soy drink	Soya drinks (including calcium fortified)
cof_capp_milk_soya_noca	Soy drink	Soya drinks (including calcium fortified)
cof_capp_milk_whole	Whole milk	Whole milk >3.6 g fat per 100 g (cow, goat, sheep)
cof_espresso	Coffee, caffeinated	Normal instant, filter, cappuccino, espresso coffee
cof_espresso_decaf	Coffee, decaffeinated	Decaffeinated instant, filter, cappuccino, espresso coffee
cof_filter	Coffee, caffeinated	Normal instant, filter, cappuccino, espresso coffee
cof_filter_decaf	Coffee, decaffeinated	Decaffeinated instant, filter, cappuccino, espresso coffee
cof_instant	Coffee, caffeinated	Normal instant, filter, cappuccino, espresso coffee
cof_instant_decaf	Coffee, decaffeinated	Decaffeinated instant, filter, cappuccino, espresso coffee
cof_latte	Coffee, caffeinated	Normal instant, filter, cappuccino, espresso coffee
cof_latte_decaf	Coffee, decaffeinated	Decaffeinated instant, filter, cappuccino, espresso coffee
cof_latte_milk_chol	Skimmed milk	Skimmed milk <1 g fat per 100 g (cow, cholesterol lowering, powdered)
cof_latte_milk_dontknow	Semi-skimmed milk	Semi-skimmed milk >1 g fat per 100 g (cow, other)
cof_latte_milk_goatsheep	Whole milk	Whole milk >3.6 g fat per 100 g (cow, goat, sheep)
cof_latte_milk_other	Semi-skimmed milk	Semi-skimmed milk >1 g fat per 100 g (cow, other)
cof_latte_milk_powdered	Skimmed milk	Skimmed milk <1 g fat per 100 g (cow, cholesterol lowering, powdered)
cof_latte_milk_riceoatveg	Rice/oat drink	Rice and oat vegetable drinks
cof_latte_milk_semi	Semi-skimmed milk	Semi-skimmed milk >1 g fat per 100 g (cow, other)
cof_latte_milk_skimmed	Skimmed milk	Skimmed milk <1 g fat per 100 g (cow, cholesterol lowering, powdered)
cof_latte_milk_soya_ca	Soy drink	Soya drinks (including calcium fortified)
cof_latte_milk_soya_noca	Soy drink	Soya drinks (including calcium fortified)
cof_latte_milk_whole	Whole milk	Whole milk >3.6 g fat per 100 g (cow, goat, sheep)
cof_other	Coffee, caffeinated	Normal instant, filter, cappuccino, espresso coffee
cof_other_decaf	Coffee, decaffeinated	Decaffeinated instant, filter, cappuccino, espresso coffee
cof_sugar	Added sugars & preserves	Table sugar, honey, jam and preserves
cream	Cream	Cream (cow's milk)
croissant	Desserts & cakes & pastries	Pancakes, croissant, Danish pastries, scones, fruitcakes, cakes, doughnuts, sponge puddings, other desserts, cereal bars, sweet snacks
crumble	Grain dishes - added fat	Double and single crust pies, crumble pies, Yorkshire pudding, snackpot noodles
danish_pastry	Desserts & cakes & pastries	Pancakes, croissant, Danish pastries, scones, fruitcakes, cakes, doughnuts, sponge puddings, other desserts, cereal bars, sweet snacks
dessert_milkbased	Milk-dairy desserts	Ice cream, milk puddings, milk-based desserts, cheesecake
dessert_milkpuds	Milk-dairy desserts	Ice cream, milk puddings, milk-based desserts, cheesecake
dessert_other	Desserts & cakes & pastries	Pancakes, croissant, Danish pastries, scones, fruitcakes, cakes, doughnuts, sponge puddings, other desserts, cereal bars, sweet snacks
dessert_soya	Soya-based desserts & yogurt	Soya-based desserts
double_crust	Grain dishes - added fat	Double and single crust pies, crumble pies, Yorkshire pudding, snackpot noodles
doughnut	Desserts & cakes & pastries	Pancakes, croissant, Danish pastries, scones, fruitcakes, cakes, doughnuts, sponge puddings, other desserts, cereal bars, sweet snacks
drink_diethotchoc	Milk-based & powdered drinks	Dairy-based smoothies, milk-based drinks, hot chocolate
drink_fizzy	SSBs & other sugary drinks	Fizzy sugary drinks, squash, fruit smoothies
drink_grapefruit	Fruit juice	Orange, grapefruit drink and 100% fruit juice
drink_hotchoc	Milk-based & powdered drinks	Dairy-based smoothies, milk-based drinks, hot chocolate
drink_hotchoc_milk_chol	Skimmed milk	Skimmed milk <1 g fat per 100 g (cow, cholesterol lowering, powdered)
drink_hotchoc_milk_dontknow	Semi-skimmed milk	Semi-skimmed milk >1 g fat per 100 g (cow, other)
drink_hotchoc_milk_goatsheep	Whole milk	Whole milk >3.6 g fat per 100 g (cow, goat, sheep)
drink_hotchoc_milk_other	Semi-skimmed milk	Semi-skimmed milk >1 g fat per 100 g (cow, other)
drink_hotchoc_milk_powdered	Skimmed milk	Skimmed milk <1 g fat per 100 g (cow, cholesterol lowering, powdered)
drink_hotchoc_milk_riceoatveg	Rice/oat drink	Rice and oat vegetable drinks
drink_hotchoc_milk_semi	Semi-skimmed milk	Semi-skimmed milk >1 g fat per 100 g (cow, other)

drink_hotchoc_milk_skimmed	Skimmed milk	Skimmed milk <1 g fat per 100 g (cow, cholesterol lowering, powdered)
drink_hotchoc_milk_soya_ca	Soy drink	Soya drinks (including calcium fortified)
drink_hotchoc_milk_soya_noca	Soy drink	Soya drinks (including calcium fortified)
drink_hotchoc_milk_whole	Whole milk	Whole milk >3.6 g fat per 100 g (cow, goat, sheep)
drink_lowcal	Low/non sugar SSBs	Low calorie fizzy drinks and squash
drink_milkbased	Milk-based & powdered drinks	Dairy-based smoothies, milk-based drinks, hot chocolate
drink_orange	Fruit juice	Orange, grapefruit drink and 100% fruit juice
drink_other	Milk-based & powdered drinks	Dairy-based smoothies, milk-based drinks, hot chocolate
drink_purejuice	Fruit juice	Orange, grapefruit drink and 100% fruit juice
drink_squash	SSBs & other sugary drinks	Fizzy sugary drinks, squash, fruit smoothies
drink_water	Water/sparkling water	Plain water, sparkling water
drizzle_oil	Olive oil (drizzling/dunking)	Olive oil
egg_omelet	Egg and egg dishes	Whole eggs and processed (omelette, scotch eggs, other)
egg_other	Egg and egg dishes	Whole eggs and processed (omelette, scotch eggs, other)
egg_scotch	Egg and egg dishes	Whole eggs and processed (omelette, scotch eggs, other)
egg_swich	Egg and egg dishes	Whole eggs and processed (omelette, scotch eggs, other)
egg_whole	Egg and egg dishes	Whole eggs and processed (omelette, scotch eggs, other)
fish_battered	Breaded/battered Fish	Fried fish with batter/breadcrumbs
fish_breaded	Breaded/battered Fish	Fried fish with batter/breadcrumbs
fish_lobcrab	Shellfish	Prawns, lobster, crab, shellfish
fish_oily	Oily fish	Oily fish, including salmon,
fish_other	White fish & tinned tuna	Tinned tuna, white fish, other fish
fish_prawns	Shellfish	Prawns, lobster, crab, shellfish
fish_shell	Shellfish	Prawns, lobster, crab, shellfish
fish_tinnedtuna	White fish & tinned tuna	Tinned tuna, white fish, other fish
fish_white	White fish & tinned tuna	Tinned tuna, white fish, other fish
fruit_apple	Apples & pears	Apples and pears
fruit_banana	Bananas & other fruit	Bananas, mixed fruit, grapes, mango, melon, peach, pineapple, kiwi, other
fruit_berry	Berries	Blackberries, strawberries, blueberries, raspberries, cherries
fruit_cherry	Berries	Blackberries, strawberries, blueberries, raspberries, cherries
fruit_dried	Dried fruit	Dried fruit, prunes
fruit_grapefruit	Citrus	Grapefruit, orange, satsuma
fruit_grapes	Bananas & other fruit	Bananas, mixed fruit, grapes, mango, melon, peach, pineapple, kiwi, other
fruit_mango	Bananas & other fruit	Bananas, mixed fruit, grapes, mango, melon, peach, pineapple, kiwi, other
fruit_melon	Bananas & other fruit	Bananas, mixed fruit, grapes, mango, melon, peach, pineapple, kiwi, other
fruit_mixed	Bananas & other fruit	Bananas, mixed fruit, grapes, mango, melon, peach, pineapple, kiwi, other
fruit_orange	Citrus	Grapefruit, orange, satsuma
fruit_other	Bananas & other fruit	Bananas, mixed fruit, grapes, mango, melon, peach, pineapple, kiwi, other
fruit_peach	Bananas & other fruit	Bananas, mixed fruit, grapes, mango, melon, peach, pineapple, kiwi, other
fruit_pear	Apples & pears	Apples and pears
fruit_pineapple	Bananas & other fruit	Bananas, mixed fruit, grapes, mango, melon, peach, pineapple, kiwi, other
fruit_plum	Stewed fruit	Stewed fruit, plums
fruit_prunes	Dried fruit	Dried fruit, prunes
fruit_satsuma	Citrus	Grapefruit, orange, satsuma
fruit_stewed	Stewed fruit	Stewed fruit, plums
fruitcake	Desserts & cakes & pastries	Pancakes, croissant, Danish pastries, scones, fruitcakes, cakes, doughnuts, sponge puddings, other desserts, cereal bars, sweet snacks
fruitcake_gf	Desserts & cakes & pastries	Pancakes, croissant, Danish pastries, scones, fruitcakes, cakes, doughnuts, sponge puddings, other desserts, cereal bars, sweet snacks
grains_couscous	White pasta & rice	White pasta, rice, couscous, gluten free pasta
grains_other	Wholemeal pasta, brown rice & other wholegrains	Brown and wholemeal pasta and rice
guacamole	Vegetable dips	Hummus, guacamole
hummus	Vegetable dips	Hummus, guacamole
icecream	Milk-dairy desserts	Ice cream, milk puddings, milk-based desserts, cheesecake
indian_snack	Samosa, pakora	Indian samosa, pakora snacks
jam_honey	Added sugars & preserves	Table sugar, honey, jam and preserves
mayo	Sauces (higher fat)	Mayonnaise, salad dressing, pesto, cheese sauce, white sauce, gravy
mayo_lowfat	Sauces (higher fat)	Mayonnaise, salad dressing, pesto, cheese sauce, white sauce, gravy
meat_bacon_nofat	Processed meat	Sausages, bacon (with and without fat), ham, liver pate
meat_bacon_withfat	Processed meat	Sausages, bacon (with and without fat), ham, liver pate
meat_beef_nofat	Beef	Beef
meat_beef_withfat	Beef	Beef
meat_ham_nofat	Processed meat	Sausages, bacon (with and without fat), ham, liver pate
meat_ham_withfat	Processed meat	Sausages, bacon (with and without fat), ham, liver pate
meat_lamb_nofat	Lamb	Lamb
meat_lamb_withfat	Lamb	Lamb
meat_liverpate	Processed meat	Sausages, bacon (with and without fat), ham, liver pate
meat_other	Other meat, offal	Other meat including offal
meat_pork_nofat	Pork	Pork
meat_pork_withfat	Pork	Pork
meat_sausage	Processed meat	Sausages, bacon (with and without fat), ham, liver pate
milk_chol_cereal	Skimmed milk	Skimmed milk <1 g fat per 100 g (cow, cholesterol lowering, powdered)
milk_chol_coffee	Skimmed milk	Skimmed milk <1 g fat per 100 g (cow, cholesterol lowering, powdered)
milk_chol_glass	Skimmed milk	Skimmed milk <1 g fat per 100 g (cow, cholesterol lowering, powdered)
milk_chol_tea	Skimmed milk	Skimmed milk <1 g fat per 100 g (cow, cholesterol lowering, powdered)
milk_dontknow_cereal	Semi-skimmed milk	Semi-skimmed milk >1 g fat per 100 g (cow, other)
milk_dontknow_coffee	Semi-skimmed milk	Semi-skimmed milk >1 g fat per 100 g (cow, other)
milk_dontknow_glass	Semi-skimmed milk	Semi-skimmed milk >1 g fat per 100 g (cow, other)
milk_dontknow_tea	Semi-skimmed milk	Semi-skimmed milk >1 g fat per 100 g (cow, other)
milk_goatsheep_cereal	Whole milk	Whole milk >3.6 g fat per 100 g (cow, goat, sheep)
milk_goatsheep_coffee	Whole milk	Whole milk >3.6 g fat per 100 g (cow, goat, sheep)
milk_goatsheep_glass	Whole milk	Whole milk >3.6 g fat per 100 g (cow, goat, sheep)
milk_goatsheep_tea	Whole milk	Whole milk >3.6 g fat per 100 g (cow, goat, sheep)
milk_other_cereal	Semi-skimmed milk	Semi-skimmed milk >1 g fat per 100 g (cow, other)
milk_other_coffee	Semi-skimmed milk	Semi-skimmed milk >1 g fat per 100 g (cow, other)
milk_other_glass	Semi-skimmed milk	Semi-skimmed milk >1 g fat per 100 g (cow, other)
milk_other_tea	Semi-skimmed milk	Semi-skimmed milk >1 g fat per 100 g (cow, other)
milk_powdered_cereal	Skimmed milk	Skimmed milk <1 g fat per 100 g (cow, cholesterol lowering, powdered)
milk_powdered_coffee	Skimmed milk	Skimmed milk <1 g fat per 100 g (cow, cholesterol lowering, powdered)
milk_powdered_glass	Skimmed milk	Skimmed milk <1 g fat per 100 g (cow, cholesterol lowering, powdered)
milk_powdered_tea	Skimmed milk	Skimmed milk <1 g fat per 100 g (cow, cholesterol lowering, powdered)
milk_riceoatveg_cereal	Rice/oat drink	Rice and oat vegetable drinks
milk_riceoatveg_coffee	Rice/oat drink	Rice and oat vegetable drinks
milk_riceoatveg_glass	Rice/oat drink	Rice and oat vegetable drinks
milk_riceoatveg_tea	Rice/oat drink	Rice and oat vegetable drinks
milk_semi_cereal	Semi-skimmed milk	Semi-skimmed milk >1 g fat per 100 g (cow, other)
milk_semi_coffee	Semi-skimmed milk	Semi-skimmed milk >1 g fat per 100 g (cow, other)
milk_semi_glass	Semi-skimmed milk	Semi-skimmed milk >1 g fat per 100 g (cow, other)
milk_semi_tea	Semi-skimmed milk	Semi-skimmed milk >1 g fat per 100 g (cow, other)
milk_skimmed_cereal	Skimmed milk	Skimmed milk <1 g fat per 100 g (cow, cholesterol lowering, powdered)
milk_skimmed_coffee	Skimmed milk	Skimmed milk <1 g fat per 100 g (cow, cholesterol lowering, powdered)
milk_skimmed_glass	Skimmed milk	Skimmed milk <1 g fat per 100 g (cow, cholesterol lowering, powdered)
milk_skimmed_tea	Skimmed milk	Skimmed milk <1 g fat per 100 g (cow, cholesterol lowering, powdered)
milk_soya_ca_cereal	Soy drink	Soya drinks (including calcium fortified)
milk_soya_ca_coffee	Soy drink	Soya drinks (including calcium fortified)
milk_soya_ca_glass	Soy drink	Soya drinks (including calcium fortified)
milk_soya_ca_tea	Soy drink	Soya drinks (including calcium fortified)
milk_soya_noca_cereal	Soy drink	Soya drinks (including calcium fortified)
milk_soya_noca_coffee	Soy drink	Soya drinks (including calcium fortified)
milk_soya_noca_glass	Soy drink	Soya drinks (including calcium fortified)
milk_soya_noca_tea	Soy drink	Soya drinks (including calcium fortified)
milk_whole_cereal	Whole milk	Whole milk >3.6 g fat per 100 g (cow, goat, sheep)
milk_whole_coffee	Whole milk	Whole milk >3.6 g fat per 100 g (cow, goat, sheep)
milk_whole_glass	Whole milk	Whole milk >3.6 g fat per 100 g (cow, goat, sheep)
milk_whole_tea	Whole milk	Whole milk >3.6 g fat per 100 g (cow, goat, sheep)
oatcakes	Savoury crackers	Oatcakes, crispbreads (including gluten free)
oatcakes_spread_butter_dunno_med	Dairy fat spread	Spreadable normal fat butter, dairy-based normal fat spread (including cholesterol lowering spread)
oatcakes_spread_butter_dunno_thick	Dairy fat spread	Spreadable normal fat butter, dairy-based normal fat spread (including cholesterol lowering spread)
oatcakes_spread_butter_dunno_thin	Dairy fat spread	Spreadable normal fat butter, dairy-based normal fat spread (including cholesterol lowering spread)
oatcakes_spread_butter_fat_med	Dairy fat spread	Spreadable normal fat butter, dairy-based normal fat spread (including cholesterol lowering spread)
oatcakes_spread_butter_fat_thick	Dairy fat spread	Spreadable normal fat butter, dairy-based normal fat spread (including cholesterol lowering spread)
oatcakes_spread_butter_fat_thin	Dairy fat spread	Spreadable normal fat butter, dairy-based normal fat spread (including cholesterol lowering spread)
oatcakes_spread_butter_lowfat_med	Dairy fat spread lower fat	Spreadable/lower fat butter, dairy-based very low fat spread
oatcakes_spread_butter_lowfat_thick	Dairy fat spread lower fat	Spreadable/lower fat butter, dairy-based very low fat spread
oatcakes_spread_butter_lowfat_thin	Dairy fat spread lower fat	Spreadable/lower fat butter, dairy-based very low fat spread
oatcakes_spread_butter_spread_fat_med	Dairy fat spread	Spreadable normal fat butter, dairy-based normal fat spread (including cholesterol lowering spread)
oatcakes_spread_butter_spread_fat_thick	Dairy fat spread	Spreadable normal fat butter, dairy-based normal fat spread (including cholesterol lowering spread)
oatcakes_spread_butter_spread_fat_thin	Dairy fat spread	Spreadable normal fat butter, dairy-based normal fat spread (including cholesterol lowering spread)
oatcakes_spread_butter_spread_lowfat_med	Dairy fat spread lower fat	Spreadable/lower fat butter, dairy-based very low fat spread
oatcakes_spread_butter_spread_lowfat_thick	Dairy fat spread lower fat	Spreadable/lower fat butter, dairy-based very low fat spread
oatcakes_spread_butter_spread_lowfat_thin	Dairy fat spread lower fat	Spreadable/lower fat butter, dairy-based very low fat spread
oatcakes_spread_dairy_chol_med	Dairy fat spread	Spreadable normal fat butter, dairy-based normal fat spread (including cholesterol lowering spread)
oatcakes_spread_dairy_chol_thick	Dairy fat spread	Spreadable normal fat butter, dairy-based normal fat spread (including cholesterol lowering spread)
oatcakes_spread_dairy_chol_thin	Dairy fat spread	Spreadable normal fat butter, dairy-based normal fat spread (including cholesterol lowering spread)
oatcakes_spread_dairy_dunno_med	Dairy fat spread	Spreadable normal fat butter, dairy-based normal fat spread (including cholesterol lowering spread)
oatcakes_spread_dairy_dunno_thick	Dairy fat spread	Spreadable normal fat butter, dairy-based normal fat spread (including cholesterol lowering spread)
oatcakes_spread_dairy_dunno_thin	Dairy fat spread	Spreadable normal fat butter, dairy-based normal fat spread (including cholesterol lowering spread)
oatcakes_spread_dairy_fat_med	Dairy fat spread	Spreadable normal fat butter, dairy-based normal fat spread (including cholesterol lowering spread)
oatcakes_spread_dairy_fat_thick	Dairy fat spread	Spreadable normal fat butter, dairy-based normal fat spread (including cholesterol lowering spread)
oatcakes_spread_dairy_fat_thin	Dairy fat spread	Spreadable normal fat butter, dairy-based normal fat spread (including cholesterol lowering spread)
oatcakes_spread_dairy_lowfat_med	Dairy fat spread lower fat	Spreadable/lower fat butter, dairy-based very low fat spread
oatcakes_spread_dairy_lowfat_thick	Dairy fat spread lower fat	Spreadable/lower fat butter, dairy-based very low fat spread
oatcakes_spread_dairy_lowfat_thin	Dairy fat spread lower fat	Spreadable/lower fat butter, dairy-based very low fat spread
oatcakes_spread_dairy_vlowfat_med	Dairy fat spread lower fat	Spreadable/lower fat butter, dairy-based very low fat spread
oatcakes_spread_dairy_vlowfat_thick	Dairy fat spread lower fat	Spreadable/lower fat butter, dairy-based very low fat spread
oatcakes_spread_dairy_vlowfat_thin	Dairy fat spread lower fat	Spreadable/lower fat butter, dairy-based very low fat spread
oatcakes_spread_dunno_chol_med	Vegetable spread lower fat	Olive oil based lower fat spread, plant-based lower fat margarine and soya-based lower fat spread (including cholesterol lowering spread)
oatcakes_spread_dunno_chol_thick	Vegetable spread lower fat	Olive oil based lower fat spread, plant-based lower fat margarine and soya-based lower fat spread (including cholesterol lowering spread)
oatcakes_spread_dunno_chol_thin	Vegetable spread lower fat	Olive oil based lower fat spread, plant-based lower fat margarine and soya-based lower fat spread (including cholesterol lowering spread)
oatcakes_spread_dunno_dunno_med	Vegetable spread	Olive oil based spread, plant-based soft or hard margarine and soya-based spread (including cholesterol lowering spread)
oatcakes_spread_dunno_dunno_thick	Vegetable spread	Olive oil based spread, plant-based soft or hard margarine and soya-based spread (including cholesterol lowering spread)
oatcakes_spread_dunno_dunno_thin	Vegetable spread	Olive oil based spread, plant-based soft or hard margarine and soya-based spread (including cholesterol lowering spread)
oatcakes_spread_dunno_fat_med	Vegetable spread	Olive oil based spread, plant-based soft or hard margarine and soya-based spread (including cholesterol lowering spread)
oatcakes_spread_dunno_fat_thick	Vegetable spread	Olive oil based spread, plant-based soft or hard margarine and soya-based spread (including cholesterol lowering spread)

oatcakes_spread_dunno_fat_thin	Vegetable spread	Olive oil based spread, plant-based soft or hard margarine and soya-based spread (including cholesterol lowering spread)
oatcakes_spread_dunno_lowfat_med	Vegetable spread lower fat	Olive oil based lower fat spread, plant-based lower fat margarine and soya-based lower fat spread (including cholesterol lowering spread)
oatcakes_spread_dunno_lowfat_thick	Vegetable spread lower fat	Olive oil based lower fat spread, plant-based lower fat margarine and soya-based lower fat spread (including cholesterol lowering spread)
oatcakes_spread_dunno_lowfat_thin	Vegetable spread lower fat	Olive oil based lower fat spread, plant-based lower fat margarine and soya-based lower fat spread (including cholesterol lowering spread)
oatcakes_spread_dunno_vlowfat_med	Vegetable spread lower fat	Olive oil based lower fat spread, plant-based lower fat margarine and soya-based lower fat spread (including cholesterol lowering spread)
oatcakes_spread_dunno_vlowfat_thick	Vegetable spread lower fat	Olive oil based lower fat spread, plant-based lower fat margarine and soya-based lower fat spread (including cholesterol lowering spread)
oatcakes_spread_dunno_vlowfat_thin	Vegetable spread lower fat	Olive oil based lower fat spread, plant-based lower fat margarine and soya-based lower fat spread (including cholesterol lowering spread)
oatcakes_spread_hardmarg_med	Vegetable spread	Olive oil based spread, plant-based soft or hard margarine and soya-based spread (including cholesterol lowering spread)
oatcakes_spread_hardmarg_thick	Vegetable spread	Olive oil based spread, plant-based soft or hard margarine and soya-based spread (including cholesterol lowering spread)
oatcakes_spread_hardmarg_thin	Vegetable spread	Olive oil based spread, plant-based soft or hard margarine and soya-based spread (including cholesterol lowering spread)
oatcakes_spread_olive_chol_med	Vegetable spread	Olive oil based spread, plant-based soft or hard margarine and soya-based spread (including cholesterol lowering spread)
oatcakes_spread_olive_chol_thick	Vegetable spread	Olive oil based spread, plant-based soft or hard margarine and soya-based spread (including cholesterol lowering spread)
oatcakes_spread_olive_chol_thin	Vegetable spread	Olive oil based spread, plant-based soft or hard margarine and soya-based spread (including cholesterol lowering spread)
oatcakes_spread_olive_dunno_med	Vegetable spread	Olive oil based spread, plant-based soft or hard margarine and soya-based spread (including cholesterol lowering spread)
oatcakes_spread_olive_dunno_thick	Vegetable spread	Olive oil based spread, plant-based soft or hard margarine and soya-based spread (including cholesterol lowering spread)
oatcakes_spread_olive_dunno_thin	Vegetable spread	Olive oil based spread, plant-based soft or hard margarine and soya-based spread (including cholesterol lowering spread)
oatcakes_spread_olive_fat_med	Vegetable spread	Olive oil based spread, plant-based soft or hard margarine and soya-based spread (including cholesterol lowering spread)
oatcakes_spread_olive_fat_thick	Vegetable spread	Olive oil based spread, plant-based soft or hard margarine and soya-based spread (including cholesterol lowering spread)
oatcakes_spread_olive_fat_thin	Vegetable spread	Olive oil based spread, plant-based soft or hard margarine and soya-based spread (including cholesterol lowering spread)
oatcakes_spread_olive_lowfat_med	Vegetable spread lower fat	Olive oil based lower fat spread, plant-based lower fat margarine and soya-based lower fat spread (including cholesterol lowering spread)
oatcakes_spread_olive_lowfat_thick	Vegetable spread lower fat	Olive oil based lower fat spread, plant-based lower fat margarine and soya-based lower fat spread (including cholesterol lowering spread)
oatcakes_spread_olive_lowfat_thin	Vegetable spread lower fat	Olive oil based lower fat spread, plant-based lower fat margarine and soya-based lower fat spread (including cholesterol lowering spread)
oatcakes_spread_olive_vlowfat_med	Vegetable spread lower fat	Olive oil based lower fat spread, plant-based lower fat margarine and soya-based lower fat spread (including cholesterol lowering spread)
oatcakes_spread_olive_vlowfat_thick	Vegetable spread lower fat	Olive oil based lower fat spread, plant-based lower fat margarine and soya-based lower fat spread (including cholesterol lowering spread)
oatcakes_spread_olive_vlowfat_thin	Vegetable spread lower fat	Olive oil based lower fat spread, plant-based lower fat margarine and soya-based lower fat spread (including cholesterol lowering spread)
oatcakes_spread_other_med	Dairy fat spread	Spreadable normal fat butter, dairy-based normal fat spread (including cholesterol lowering spread)
oatcakes_spread_other_thick	Dairy fat spread	Spreadable normal fat butter, dairy-based normal fat spread (including cholesterol lowering spread)
oatcakes_spread_other_thin	Dairy fat spread	Spreadable normal fat butter, dairy-based normal fat spread (including cholesterol lowering spread)
oatcakes_spread_polymarg_chol_med	Vegetable spread	Olive oil based spread, plant-based soft or hard margarine and soya-based spread (including cholesterol lowering spread)
oatcakes_spread_polymarg_chol_thick	Vegetable spread	Olive oil based spread, plant-based soft or hard margarine and soya-based spread (including cholesterol lowering spread)
oatcakes_spread_polymarg_chol_thin	Vegetable spread	Olive oil based spread, plant-based soft or hard margarine and soya-based spread (including cholesterol lowering spread)
oatcakes_spread_polymarg_dunno_med	Vegetable spread	Olive oil based spread, plant-based soft or hard margarine and soya-based spread (including cholesterol lowering spread)
oatcakes_spread_polymarg_dunno_thick	Vegetable spread	Olive oil based spread, plant-based soft or hard margarine and soya-based spread (including cholesterol lowering spread)
oatcakes_spread_polymarg_dunno_thin	Vegetable spread	Olive oil based spread, plant-based soft or hard margarine and soya-based spread (including cholesterol lowering spread)
oatcakes_spread_polymarg_fat_med	Vegetable spread	Olive oil based spread, plant-based soft or hard margarine and soya-based spread (including cholesterol lowering spread)
oatcakes_spread_polymarg_fat_thick	Vegetable spread	Olive oil based spread, plant-based soft or hard margarine and soya-based spread (including cholesterol lowering spread)
oatcakes_spread_polymarg_fat_thin	Vegetable spread	Olive oil based spread, plant-based soft or hard margarine and soya-based spread (including cholesterol lowering spread)
oatcakes_spread_polymarg_lowfat_med	Vegetable spread lower fat	Olive oil based lower fat spread, plant-based lower fat margarine and soya-based lower fat spread (including cholesterol lowering spread)
oatcakes_spread_polymarg_lowfat_thick	Vegetable spread lower fat	Olive oil based lower fat spread, plant-based lower fat margarine and soya-based lower fat spread (including cholesterol lowering spread)
oatcakes_spread_polymarg_lowfat_thin	Vegetable spread lower fat	Olive oil based lower fat spread, plant-based lower fat margarine and soya-based lower fat spread (including cholesterol lowering spread)
oatcakes_spread_polymarg_vlowfat_med	Vegetable spread lower fat	Olive oil based lower fat spread, plant-based lower fat margarine and soya-based lower fat spread (including cholesterol lowering spread)
oatcakes_spread_polymarg_vlowfat_thick	Vegetable spread lower fat	Olive oil based lower fat spread, plant-based lower fat margarine and soya-based lower fat spread (including cholesterol lowering spread)
oatcakes_spread_polymarg_vlowfat_thin	Vegetable spread lower fat	Olive oil based lower fat spread, plant-based lower fat margarine and soya-based lower fat spread (including cholesterol lowering spread)
oatcakes_spread_soya_chol_med	Vegetable spread	Olive oil based spread, plant-based soft or hard margarine and soya-based spread (including cholesterol lowering spread)
oatcakes_spread_soya_chol_thick	Vegetable spread	Olive oil based spread, plant-based soft or hard margarine and soya-based spread (including cholesterol lowering spread)
oatcakes_spread_soya_chol_thin	Vegetable spread	Olive oil based spread, plant-based soft or hard margarine and soya-based spread (including cholesterol lowering spread)
oatcakes_spread_soya_dunno_med	Vegetable spread	Olive oil based spread, plant-based soft or hard margarine and soya-based spread (including cholesterol lowering spread)
oatcakes_spread_soya_dunno_thick	Vegetable spread	Olive oil based spread, plant-based soft or hard margarine and soya-based spread (including cholesterol lowering spread)
oatcakes_spread_soya_dunno_thin	Vegetable spread	Olive oil based spread, plant-based soft or hard margarine and soya-based spread (including cholesterol lowering spread)
oatcakes_spread_soya_fat_med	Vegetable spread	Olive oil based spread, plant-based soft or hard margarine and soya-based spread (including cholesterol lowering spread)
oatcakes_spread_soya_fat_thick	Vegetable spread	Olive oil based spread, plant-based soft or hard margarine and soya-based spread (including cholesterol lowering spread)
oatcakes_spread_soya_fat_thin	Vegetable spread	Olive oil based spread, plant-based soft or hard margarine and soya-based spread (including cholesterol lowering spread)
oatcakes_spread_soya_lowfat_med	Vegetable spread lower fat	Olive oil based lower fat spread, plant-based lower fat margarine and soya-based lower fat spread (including cholesterol lowering spread)
oatcakes_spread_soya_lowfat_thick	Vegetable spread lower fat	Olive oil based lower fat spread, plant-based lower fat margarine and soya-based lower fat spread (including cholesterol lowering spread)
oatcakes_spread_soya_lowfat_thin	Vegetable spread lower fat	Olive oil based lower fat spread, plant-based lower fat margarine and soya-based lower fat spread (including cholesterol lowering spread)
oatcakes_spread_soya_vlowfat_med	Vegetable spread lower fat	Olive oil based lower fat spread, plant-based lower fat margarine and soya-based lower fat spread (including cholesterol lowering spread)
oatcakes_spread_soya_vlowfat_thick	Vegetable spread lower fat	Olive oil based lower fat spread, plant-based lower fat margarine and soya-based lower fat spread (including cholesterol lowering spread)
oatcakes_spread_soya_vlowfat_thin	Vegetable spread lower fat	Olive oil based lower fat spread, plant-based lower fat margarine and soya-based lower fat spread (including cholesterol lowering spread)
pancake_blini_chol	Desserts & cakes & pastries	Pancakes, croissant, Danish pastries, scones, fruitcakes, cakes, doughnuts, sponge puddings, other desserts, cereal bars, sweet snacks
pancake_blini_dontknow	Desserts & cakes & pastries	Pancakes, croissant, Danish pastries, scones, fruitcakes, cakes, doughnuts, sponge puddings, other desserts, cereal bars, sweet snacks
pancake_blini_goatsheep	Desserts & cakes & pastries	Pancakes, croissant, Danish pastries, scones, fruitcakes, cakes, doughnuts, sponge puddings, other desserts, cereal bars, sweet snacks
pancake_blini_other	Desserts & cakes & pastries	Pancakes, croissant, Danish pastries, scones, fruitcakes, cakes, doughnuts, sponge puddings, other desserts, cereal bars, sweet snacks
pancake_blini_powdered	Desserts & cakes & pastries	Pancakes, croissant, Danish pastries, scones, fruitcakes, cakes, doughnuts, sponge puddings, other desserts, cereal bars, sweet snacks
pancake_blini_riceoatveg	Desserts & cakes & pastries	Pancakes, croissant, Danish pastries, scones, fruitcakes, cakes, doughnuts, sponge puddings, other desserts, cereal bars, sweet snacks
pancake_blini_semi	Desserts & cakes & pastries	Pancakes, croissant, Danish pastries, scones, fruitcakes, cakes, doughnuts, sponge puddings, other desserts, cereal bars, sweet snacks
pancake_blini_skimmed	Desserts & cakes & pastries	Pancakes, croissant, Danish pastries, scones, fruitcakes, cakes, doughnuts, sponge puddings, other desserts, cereal bars, sweet snacks
pancake_blini_soya_ca	Desserts & cakes & pastries	Pancakes, croissant, Danish pastries, scones, fruitcakes, cakes, doughnuts, sponge puddings, other desserts, cereal bars, sweet snacks
pancake_blini_soya_noca	Desserts & cakes & pastries	Pancakes, croissant, Danish pastries, scones, fruitcakes, cakes, doughnuts, sponge puddings, other desserts, cereal bars, sweet snacks
pancake_blini_whole	Desserts & cakes & pastries	Pancakes, croissant, Danish pastries, scones, fruitcakes, cakes, doughnuts, sponge puddings, other desserts, cereal bars, sweet snacks
pancake_crepe_chol	Desserts & cakes & pastries	Pancakes, croissant, Danish pastries, scones, fruitcakes, cakes, doughnuts, sponge puddings, other desserts, cereal bars, sweet snacks
pancake_crepe_dontknow	Desserts & cakes & pastries	Pancakes, croissant, Danish pastries, scones, fruitcakes, cakes, doughnuts, sponge puddings, other desserts, cereal bars, sweet snacks
pancake_crepe_goatsheep	Desserts & cakes & pastries	Pancakes, croissant, Danish pastries, scones, fruitcakes, cakes, doughnuts, sponge puddings, other desserts, cereal bars, sweet snacks
pancake_crepe_other	Desserts & cakes & pastries	Pancakes, croissant, Danish pastries, scones, fruitcakes, cakes, doughnuts, sponge puddings, other desserts, cereal bars, sweet snacks
pancake_crepe_powdered	Desserts & cakes & pastries	Pancakes, croissant, Danish pastries, scones, fruitcakes, cakes, doughnuts, sponge puddings, other desserts, cereal bars, sweet snacks
pancake_crepe_riceoatveg	Desserts & cakes & pastries	Pancakes, croissant, Danish pastries, scones, fruitcakes, cakes, doughnuts, sponge puddings, other desserts, cereal bars, sweet snacks
pancake_crepe_semi	Desserts & cakes & pastries	Pancakes, croissant, Danish pastries, scones, fruitcakes, cakes, doughnuts, sponge puddings, other desserts, cereal bars, sweet snacks
pancake_crepe_skimmed	Desserts & cakes & pastries	Pancakes, croissant, Danish pastries, scones, fruitcakes, cakes, doughnuts, sponge puddings, other desserts, cereal bars, sweet snacks
pancake_crepe_soya_ca	Desserts & cakes & pastries	Pancakes, croissant, Danish pastries, scones, fruitcakes, cakes, doughnuts, sponge puddings, other desserts, cereal bars, sweet snacks
pancake_crepe_soya_noca	Desserts & cakes & pastries	Pancakes, croissant, Danish pastries, scones, fruitcakes, cakes, doughnuts, sponge puddings, other desserts, cereal bars, sweet snacks
pancake_crepe_whole	Desserts & cakes & pastries	Pancakes, croissant, Danish pastries, scones, fruitcakes, cakes, doughnuts, sponge puddings, other desserts, cereal bars, sweet snacks
pasta_brown	Wholemeal pasta, brown rice & other wholegrains	Brown and wholemeal pasta and rice
pasta_gf	White pasta & rice	White pasta, rice, couscous, gluten free pasta
pasta_white	White pasta & rice	White pasta, rice, couscous, gluten free pasta
pesto	Sauces (higher fat)	Mayonnaise, salad dressing, pesto, cheese sauce, white sauce, gravy
pizza	Pizza	Pizza (including gluten free crust)
pizza_gf	Pizza	Pizza (including gluten free crust)
pnutbutter_nutella	Nut-based spreads	Peanut-butter and chocolate-based spread
potato_boil	Potatoes/sweet potatoes (baked/boiled)	Potatoes, sweet potatoes, boiled or baked
potato_boil_marg	Potatoes/sweet potatoes (baked/boiled)	Potatoes, sweet potatoes, boiled or baked
potato_fried	Fried/roast potatoes	Potatoes and chips, fried or roasted with fat
potato_mashed_butter_dunno	Mashed potatoes	Potatoes, mashed
potato_mashed_butter_fat	Mashed potatoes	Potatoes, mashed
potato_mashed_butter_lowfat	Mashed potatoes	Potatoes, mashed
potato_mashed_butter_spread_fat	Mashed potatoes	Potatoes, mashed
potato_mashed_butter_spread_lowfat	Mashed potatoes	Potatoes, mashed
potato_mashed_fat_dunno	Mashed potatoes	Potatoes, mashed
potato_mashed_fat_none	Mashed potatoes	Potatoes, mashed
potato_mashed_fat_other	Mashed potatoes	Potatoes, mashed
potato_mashed_lard	Mashed potatoes	Potatoes, mashed
potato_mashed_marg_hard	Mashed potatoes	Potatoes, mashed
potato_mashed_marg_poly_chol	Mashed potatoes	Potatoes, mashed
potato_mashed_marg_poly_dunno	Mashed potatoes	Potatoes, mashed
potato_mashed_marg_poly_fat	Mashed potatoes	Potatoes, mashed
potato_mashed_marg_poly_lowfat	Mashed potatoes	Potatoes, mashed
potato_mashed_marg_poly_vlowfat	Mashed potatoes	Potatoes, mashed
potato_mashed_marg_soya_chol	Mashed potatoes	Potatoes, mashed
potato_mashed_marg_soya_dunno	Mashed potatoes	Potatoes, mashed
potato_mashed_marg_soya_fat	Mashed potatoes	Potatoes, mashed
potato_mashed_marg_soya_lowfat	Mashed potatoes	Potatoes, mashed
potato_mashed_marg_soya_vlowfat	Mashed potatoes	Potatoes, mashed
potato_mashed_oil_olive	Mashed potatoes	Potatoes, mashed
potato_mashed_oil_other	Mashed potatoes	Potatoes, mashed
potato_mashed_oil_rapeseed	Mashed potatoes	Potatoes, mashed
potato_mashed_oil_sunflower	Mashed potatoes	Potatoes, mashed
potato_mashed_oil_veg	Mashed potatoes	Potatoes, mashed
potato_mashed_spread_dairy_chol	Mashed potatoes	Potatoes, mashed
potato_mashed_spread_dairy_dunno	Mashed potatoes	Potatoes, mashed
potato_mashed_spread_dairy_fat	Mashed potatoes	Potatoes, mashed
potato_mashed_spread_dairy_lowfat	Mashed potatoes	Potatoes, mashed
potato_mashed_spread_dairy_vlowfat	Mashed potatoes	Potatoes, mashed
potato_mashed_spread_dunno_chol	Mashed potatoes	Potatoes, mashed
potato_mashed_spread_dunno_dunno	Mashed potatoes	Potatoes, mashed
potato_mashed_spread_dunno_fat	Mashed potatoes	Potatoes, mashed
potato_mashed_spread_dunno_lowfat	Mashed potatoes	Potatoes, mashed
potato_mashed_spread_dunno_vlowfat	Mashed potatoes	Potatoes, mashed
potato_mashed_spread_olive_chol	Mashed potatoes	Potatoes, mashed
potato_mashed_spread_olive_dunno	Mashed potatoes	Potatoes, mashed
potato_mashed_spread_olive_fat	Mashed potatoes	Potatoes, mashed
potato_mashed_spread_olive_lowfat	Mashed potatoes	Potatoes, mashed
potato_mashed_spread_olive_vlowfat	Mashed potatoes	Potatoes, mashed
poultry_friedbcrumb_noskin	Breaded/battered Chicken	Fried poultry with batter/breadcrumbs
poultry_friedbcrumb_withskin	Breaded/battered Chicken	Fried poultry with batter/breadcrumbs
poultry_noskin	Poultry	Poultry (with/without skin)
poultry_withskin	Poultry	Poultry (with/without skin)
rice_brown	Wholemeal pasta, brown rice & other wholegrains	Brown and wholemeal pasta and rice
rice_white	White pasta & rice	White pasta, rice, couscous, gluten free pasta
salad_dressing	Sauces (higher fat)	Mayonnaise, salad dressing, pesto, cheese sauce, white sauce, gravy
sauce_brown	Sauces (lower fat)	Yeast, chutney, olives, ketchup, brown sauce, tomato sauce
sauce_cheese_chol	Sauces (higher fat)	Mayonnaise, salad dressing, pesto, cheese sauce, white sauce, gravy
sauce_cheese_dontknow	Sauces (higher fat)	Mayonnaise, salad dressing, pesto, cheese sauce, white sauce, gravy
sauce_cheese_goatsheep	Sauces (higher fat)	Mayonnaise, salad dressing, pesto, cheese sauce, white sauce, gravy
sauce_cheese_other	Sauces (higher fat)	Mayonnaise, salad dressing, pesto, cheese sauce, white sauce, gravy
sauce_cheese_powdered	Sauces (higher fat)	Mayonnaise, salad dressing, pesto, cheese sauce, white sauce, gravy
sauce_cheese_riceoatveg	Sauces (higher fat)	Mayonnaise, salad dressing, pesto, cheese sauce, white sauce, gravy
sauce_cheese_semi	Sauces (higher fat)	Mayonnaise, salad dressing, pesto, cheese sauce, white sauce, gravy
sauce_cheese_skimmed	Sauces (higher fat)	Mayonnaise, salad dressing, pesto, cheese sauce, white sauce, gravy
sauce_cheese_soya_ca	Sauces (higher fat)	Mayonnaise, salad dressing, pesto, cheese sauce, white sauce, gravy
sauce_cheese_soya_noca	Sauces (higher fat)	Mayonnaise, salad dressing, pesto, cheese sauce, white sauce, gravy
sauce_cheese_whole	Sauces (higher fat)	Mayonnaise, salad dressing, pesto, cheese sauce, white sauce, gravy
sauce_gravy	Sauces (higher fat)	Mayonnaise, salad dressing, pesto, cheese sauce, white sauce, gravy
sauce_ketchup	Sauces (lower fat)	Yeast, chutney, olives, ketchup, brown sauce, tomato sauce
sauce_tomato	Sauces (lower fat)	Yeast, chutney, olives, ketchup, brown sauce, tomato sauce

sauce_white_chol	Sauces (higher fat)	Mayonnaise, salad dressing, pesto, cheese sauce, white sauce, gravy
sauce_white_dontknow	Sauces (higher fat)	Mayonnaise, salad dressing, pesto, cheese sauce, white sauce, gravy
sauce_white_goatsheep	Sauces (higher fat)	Mayonnaise, salad dressing, pesto, cheese sauce, white sauce, gravy
sauce_white_other	Sauces (higher fat)	Mayonnaise, salad dressing, pesto, cheese sauce, white sauce, gravy
sauce_white_powdered	Sauces (higher fat)	Mayonnaise, salad dressing, pesto, cheese sauce, white sauce, gravy
sauce_white_riceoatveg	Sauces (higher fat)	Mayonnaise, salad dressing, pesto, cheese sauce, white sauce, gravy
sauce_white_semi	Sauces (higher fat)	Mayonnaise, salad dressing, pesto, cheese sauce, white sauce, gravy
sauce_white_skimmed	Sauces (higher fat)	Mayonnaise, salad dressing, pesto, cheese sauce, white sauce, gravy
sauce_white_soya_ca	Sauces (higher fat)	Mayonnaise, salad dressing, pesto, cheese sauce, white sauce, gravy
sauce_white_soya_noca	Sauces (higher fat)	Mayonnaise, salad dressing, pesto, cheese sauce, white sauce, gravy
sauce_white_whole	Sauces (higher fat)	Mayonnaise, salad dressing, pesto, cheese sauce, white sauce, gravy
scone	Desserts & cakes & pastries	Pancakes, croissant, Danish pastries, scones, fruitcakes, cakes, doughnuts, sponge puddings, other desserts, cereal bars, sweet snacks
scone_gf	Desserts & cakes & pastries	Pancakes, croissant, Danish pastries, scones, fruitcakes, cakes, doughnuts, sponge puddings, other desserts, cereal bars, sweet snacks
single_crust	Grain dishes - added fat	Double and single crust pies, crumble pies, Yorkshire pudding, snackpot noodles
smoothie_dairy	Milk-based & powdered drinks	Dairy-based smoothies, milk-based drinks, hot chocolate
smoothie_fruit	SSBs & other sugary drinks	Fizzy sugary drinks, squash, fruit smoothies
snack_cheesybis	Savoury snacks	Crisps, savoury biscuits, cheese snacks, other savoury biscuits
snack_crisps	Savoury snacks	Crisps, savoury biscuits, cheese snacks, other savoury biscuits
snack_olives	Sauces (lower fat)	Yeast, chutney, olives, ketchup, brown sauce, tomato sauce
snack_saltednuts	Salted nuts & seeds	Salted peanuts and nuts
snack_saltedpeanuts	Salted nuts & seeds	Salted peanuts and nuts
snack_savourybis	Savoury snacks	Crisps, savoury biscuits, cheese snacks, other savoury biscuits
snack_seeds	Unsalted nuts & seeds	Unsalted peanuts and nuts
snack_svyother	Savoury snacks	Crisps, savoury biscuits, cheese snacks, other savoury biscuits
snack_swtother	Desserts & cakes & pastries	Pancakes, croissant, Danish pastries, scones, fruitcakes, cakes, doughnuts, sponge puddings, other desserts, cereal bars, sweet snacks
snack_unsaltednuts	Unsalted nuts & seeds	Unsalted peanuts and nuts
snack_unsaltedpeanuts	Unsalted nuts & seeds	Unsalted peanuts and nuts
snackpot	Grain dishes - added fat	Double and single crust pies, crumble pies, Yorkshire pudding, snackpot noodles
soup_canned_fish	Soups	Soups, homemade, powdered and canned
soup_canned_meat	Soups	Soups, homemade, powdered and canned
soup_canned_other	Soups	Soups, homemade, powdered and canned
soup_canned_pasta	Soups	Soups, homemade, powdered and canned
soup_canned_pulse	Soups	Soups, homemade, powdered and canned
soup_canned_unanswered	Soups	Soups, homemade, powdered and canned
soup_canned_veg	Soups	Soups, homemade, powdered and canned
soup_homemade_fish	Soups	Soups, homemade, powdered and canned
soup_homemade_meat	Soups	Soups, homemade, powdered and canned
soup_homemade_other	Soups	Soups, homemade, powdered and canned
soup_homemade_pasta	Soups	Soups, homemade, powdered and canned
soup_homemade_pulse	Soups	Soups, homemade, powdered and canned
soup_homemade_unanswered	Soups	Soups, homemade, powdered and canned
soup_homemade_veg	Soups	Soups, homemade, powdered and canned
soup_powder	Soups	Soups, homemade, powdered and canned
spongepuds	Desserts & cakes & pastries	Pancakes, croissant, Danish pastries, scones, fruitcakes, cakes, doughnuts, sponge puddings, other desserts, cereal bars, sweet snacks
spongepuds_gf	Desserts & cakes & pastries	Pancakes, croissant, Danish pastries, scones, fruitcakes, cakes, doughnuts, sponge puddings, other desserts, cereal bars, sweet snacks
spreadsauce_other	Sauces (higher fat)	Mayonnaise, salad dressing, pesto, cheese sauce, white sauce, gravy
sushi	Sushi	Sushi
sweets	Other sweets	Hard and soft sweets (including sugar free)
sweets_diet	Other sweets	Hard and soft sweets (including sugar free)
tea_black	Tea	Black, green and other tea
tea_black_decaf	Tea, decaffeinated	Decaffeinated black, herbal tea, rooibos
tea_green	Tea	Black, green and other tea
tea_herbal	Tea, decaffeinated	Decaffeinated black, herbal tea, rooibos
tea_other	Tea	Black, green and other tea
tea_rooibos	Tea, decaffeinated	Decaffeinated black, herbal tea, rooibos
tea_sugar	Added sugars & preserves	Table sugar, honey, jam and preserves
veg_avocado	Other vegetables (mushrooms, fruiting, mixed)	Mushrooms, mixed vegetables, avocado, broad beans, green beans, butternut squash, courgettes, , peppers, other
veg_bakedbeans	Legumes/pulses	Baked beans, pulses
veg_beetroot	Root vegetables	Beetroot, carrots, celery, parsnip, turnip
veg_broadbeans	Other vegetables (mushrooms, fruiting, mixed)	Mushrooms, mixed vegetables, avocado, broad beans, green beans, butternut squash, courgettes, , peppers, other
veg_broccoli	Green leafy/cabbages	Broccoli, cabbage, kale, cauliflower, spinach, sprouts
veg_butternut	Other vegetables (mushrooms, fruiting, mixed)	Mushrooms, mixed vegetables, avocado, broad beans, green beans, butternut squash, courgettes, , peppers, other
veg_cabbagekale	Green leafy/cabbages	Broccoli, cabbage, kale, cauliflower, spinach, sprouts
veg_carrots	Root vegetables	Beetroot, carrots, celery, parsnip, turnip
veg_cauli	Green leafy/cabbages	Broccoli, cabbage, kale, cauliflower, spinach, sprouts
veg_celery	Root vegetables	Beetroot, carrots, celery, parsnip, turnip
veg_courgette_butter_dunno	Other vegetables (mushrooms, fruiting, mixed)	Mushrooms, mixed vegetables, avocado, broad beans, green beans, butternut squash, courgettes, , peppers, other
veg_courgette_butter_fat	Other vegetables (mushrooms, fruiting, mixed)	Mushrooms, mixed vegetables, avocado, broad beans, green beans, butternut squash, courgettes, , peppers, other
veg_courgette_butter_lowfat	Other vegetables (mushrooms, fruiting, mixed)	Mushrooms, mixed vegetables, avocado, broad beans, green beans, butternut squash, courgettes, , peppers, other
veg_courgette_butter_spread_fat	Other vegetables (mushrooms, fruiting, mixed)	Mushrooms, mixed vegetables, avocado, broad beans, green beans, butternut squash, courgettes, , peppers, other
veg_courgette_butter_spread_lowfat	Other vegetables (mushrooms, fruiting, mixed)	Mushrooms, mixed vegetables, avocado, broad beans, green beans, butternut squash, courgettes, , peppers, other
veg_courgette_fat_dunno	Other vegetables (mushrooms, fruiting, mixed)	Mushrooms, mixed vegetables, avocado, broad beans, green beans, butternut squash, courgettes, , peppers, other
veg_courgette_fat_none	Other vegetables (mushrooms, fruiting, mixed)	Mushrooms, mixed vegetables, avocado, broad beans, green beans, butternut squash, courgettes, , peppers, other
veg_courgette_fat_other	Other vegetables (mushrooms, fruiting, mixed)	Mushrooms, mixed vegetables, avocado, broad beans, green beans, butternut squash, courgettes, , peppers, other
veg_courgette_lard	Other vegetables (mushrooms, fruiting, mixed)	Mushrooms, mixed vegetables, avocado, broad beans, green beans, butternut squash, courgettes, , peppers, other
veg_courgette_marg_hard	Other vegetables (mushrooms, fruiting, mixed)	Mushrooms, mixed vegetables, avocado, broad beans, green beans, butternut squash, courgettes, , peppers, other
veg_courgette_marg_poly_chol	Other vegetables (mushrooms, fruiting, mixed)	Mushrooms, mixed vegetables, avocado, broad beans, green beans, butternut squash, courgettes, , peppers, other
veg_courgette_marg_poly_dunno	Other vegetables (mushrooms, fruiting, mixed)	Mushrooms, mixed vegetables, avocado, broad beans, green beans, butternut squash, courgettes, , peppers, other
veg_courgette_marg_poly_fat	Other vegetables (mushrooms, fruiting, mixed)	Mushrooms, mixed vegetables, avocado, broad beans, green beans, butternut squash, courgettes, , peppers, other
veg_courgette_marg_poly_lowfat	Other vegetables (mushrooms, fruiting, mixed)	Mushrooms, mixed vegetables, avocado, broad beans, green beans, butternut squash, courgettes, , peppers, other
veg_courgette_marg_poly_vlowfat	Other vegetables (mushrooms, fruiting, mixed)	Mushrooms, mixed vegetables, avocado, broad beans, green beans, butternut squash, courgettes, , peppers, other
veg_courgette_marg_soya_chol	Other vegetables (mushrooms, fruiting, mixed)	Mushrooms, mixed vegetables, avocado, broad beans, green beans, butternut squash, courgettes, , peppers, other
veg_courgette_marg_soya_dunno	Other vegetables (mushrooms, fruiting, mixed)	Mushrooms, mixed vegetables, avocado, broad beans, green beans, butternut squash, courgettes, , peppers, other
veg_courgette_marg_soya_fat	Other vegetables (mushrooms, fruiting, mixed)	Mushrooms, mixed vegetables, avocado, broad beans, green beans, butternut squash, courgettes, , peppers, other
veg_courgette_marg_soya_lowfat	Other vegetables (mushrooms, fruiting, mixed)	Mushrooms, mixed vegetables, avocado, broad beans, green beans, butternut squash, courgettes, , peppers, other
veg_courgette_marg_soya_vlowfat	Other vegetables (mushrooms, fruiting, mixed)	Mushrooms, mixed vegetables, avocado, broad beans, green beans, butternut squash, courgettes, , peppers, other
veg_courgette_oil_olive	Other vegetables (mushrooms, fruiting, mixed)	Mushrooms, mixed vegetables, avocado, broad beans, green beans, butternut squash, courgettes, , peppers, other
veg_courgette_oil_other	Other vegetables (mushrooms, fruiting, mixed)	Mushrooms, mixed vegetables, avocado, broad beans, green beans, butternut squash, courgettes, , peppers, other
veg_courgette_oil_rapeseed	Other vegetables (mushrooms, fruiting, mixed)	Mushrooms, mixed vegetables, avocado, broad beans, green beans, butternut squash, courgettes, , peppers, other
veg_courgette_oil_sunflower	Other vegetables (mushrooms, fruiting, mixed)	Mushrooms, mixed vegetables, avocado, broad beans, green beans, butternut squash, courgettes, , peppers, other
veg_courgette_oil_veg	Other vegetables (mushrooms, fruiting, mixed)	Mushrooms, mixed vegetables, avocado, broad beans, green beans, butternut squash, courgettes, , peppers, other
veg_courgette_spread_dairy_chol	Other vegetables (mushrooms, fruiting, mixed)	Mushrooms, mixed vegetables, avocado, broad beans, green beans, butternut squash, courgettes, , peppers, other
veg_courgette_spread_dairy_dunno	Other vegetables (mushrooms, fruiting, mixed)	Mushrooms, mixed vegetables, avocado, broad beans, green beans, butternut squash, courgettes, , peppers, other
veg_courgette_spread_dairy_fat	Other vegetables (mushrooms, fruiting, mixed)	Mushrooms, mixed vegetables, avocado, broad beans, green beans, butternut squash, courgettes, , peppers, other
veg_courgette_spread_dairy_lowfat	Other vegetables (mushrooms, fruiting, mixed)	Mushrooms, mixed vegetables, avocado, broad beans, green beans, butternut squash, courgettes, , peppers, other
veg_courgette_spread_dairy_vlowfat	Other vegetables (mushrooms, fruiting, mixed)	Mushrooms, mixed vegetables, avocado, broad beans, green beans, butternut squash, courgettes, , peppers, other
veg_courgette_spread_dunno_chol	Other vegetables (mushrooms, fruiting, mixed)	Mushrooms, mixed vegetables, avocado, broad beans, green beans, butternut squash, courgettes, , peppers, other
veg_courgette_spread_dunno_dunno	Other vegetables (mushrooms, fruiting, mixed)	Mushrooms, mixed vegetables, avocado, broad beans, green beans, butternut squash, courgettes, , peppers, other
veg_courgette_spread_dunno_fat	Other vegetables (mushrooms, fruiting, mixed)	Mushrooms, mixed vegetables, avocado, broad beans, green beans, butternut squash, courgettes, , peppers, other
veg_courgette_spread_dunno_lowfat	Other vegetables (mushrooms, fruiting, mixed)	Mushrooms, mixed vegetables, avocado, broad beans, green beans, butternut squash, courgettes, , peppers, other
veg_courgette_spread_dunno_vlowfat	Other vegetables (mushrooms, fruiting, mixed)	Mushrooms, mixed vegetables, avocado, broad beans, green beans, butternut squash, courgettes, , peppers, other
veg_courgette_spread_olive_chol	Other vegetables (mushrooms, fruiting, mixed)	Mushrooms, mixed vegetables, avocado, broad beans, green beans, butternut squash, courgettes, , peppers, other
veg_courgette_spread_olive_dunno	Other vegetables (mushrooms, fruiting, mixed)	Mushrooms, mixed vegetables, avocado, broad beans, green beans, butternut squash, courgettes, , peppers, other
veg_courgette_spread_olive_fat	Other vegetables (mushrooms, fruiting, mixed)	Mushrooms, mixed vegetables, avocado, broad beans, green beans, butternut squash, courgettes, , peppers, other
veg_courgette_spread_olive_lowfat	Other vegetables (mushrooms, fruiting, mixed)	Mushrooms, mixed vegetables, avocado, broad beans, green beans, butternut squash, courgettes, , peppers, other
veg_courgette_spread_olive_vlowfat	Other vegetables (mushrooms, fruiting, mixed)	Mushrooms, mixed vegetables, avocado, broad beans, green beans, butternut squash, courgettes, , peppers, other
veg_cucumber	Other vegetables (mushrooms, fruiting, mixed)	Mushrooms, mixed vegetables, avocado, broad beans, green beans, butternut squash, courgettes, , peppers, other
veg_garlic	Allium vegetables	Garlic, leek, onion
veg_greenbeans	Other vegetables (mushrooms, fruiting, mixed)	Mushrooms, mixed vegetables, avocado, broad beans, green beans, butternut squash, courgettes, , peppers, other
veg_leek_butter_dunno	Allium vegetables	Garlic, leek, onion
veg_leek_butter_fat	Allium vegetables	Garlic, leek, onion
veg_leek_butter_lowfat	Allium vegetables	Garlic, leek, onion
veg_leek_butter_spread_fat	Allium vegetables	Garlic, leek, onion
veg_leek_butter_spread_lowfat	Allium vegetables	Garlic, leek, onion
veg_leek_fat_dunno	Allium vegetables	Garlic, leek, onion
veg_leek_fat_none	Allium vegetables	Garlic, leek, onion
veg_leek_fat_other	Allium vegetables	Garlic, leek, onion
veg_leek_lard	Allium vegetables	Garlic, leek, onion
veg_leek_marg_hard	Allium vegetables	Garlic, leek, onion
veg_leek_marg_poly_chol	Allium vegetables	Garlic, leek, onion
veg_leek_marg_poly_dunno	Allium vegetables	Garlic, leek, onion
veg_leek_marg_poly_fat	Allium vegetables	Garlic, leek, onion
veg_leek_marg_poly_lowfat	Allium vegetables	Garlic, leek, onion
veg_leek_marg_poly_vlowfat	Allium vegetables	Garlic, leek, onion
veg_leek_marg_soya_chol	Allium vegetables	Garlic, leek, onion
veg_leek_marg_soya_dunno	Allium vegetables	Garlic, leek, onion
veg_leek_marg_soya_fat	Allium vegetables	Garlic, leek, onion
veg_leek_marg_soya_lowfat	Allium vegetables	Garlic, leek, onion
veg_leek_marg_soya_vlowfat	Allium vegetables	Garlic, leek, onion
veg_leek_oil_olive	Allium vegetables	Garlic, leek, onion
veg_leek_oil_other	Allium vegetables	Garlic, leek, onion
veg_leek_oil_rapeseed	Allium vegetables	Garlic, leek, onion
veg_leek_oil_sunflower	Allium vegetables	Garlic, leek, onion
veg_leek_oil_veg	Allium vegetables	Garlic, leek, onion
veg_leek_spread_dairy_chol	Allium vegetables	Garlic, leek, onion
veg_leek_spread_dairy_dunno	Allium vegetables	Garlic, leek, onion
veg_leek_spread_dairy_fat	Allium vegetables	Garlic, leek, onion
veg_leek_spread_dairy_lowfat	Allium vegetables	Garlic, leek, onion
veg_leek_spread_dairy_vlowfat	Allium vegetables	Garlic, leek, onion
veg_leek_spread_dunno_chol	Allium vegetables	Garlic, leek, onion
veg_leek_spread_dunno_dunno	Allium vegetables	Garlic, leek, onion
veg_leek_spread_dunno_fat	Allium vegetables	Garlic, leek, onion
veg_leek_spread_dunno_lowfat	Allium vegetables	Garlic, leek, onion
veg_leek_spread_dunno_vlowfat	Allium vegetables	Garlic, leek, onion
veg_leek_spread_olive_chol	Allium vegetables	Garlic, leek, onion
veg_leek_spread_olive_dunno	Allium vegetables	Garlic, leek, onion
veg_leek_spread_olive_fat	Allium vegetables	Garlic, leek, onion
veg_leek_spread_olive_lowfat	Allium vegetables	Garlic, leek, onion
veg_leek_spread_olive_vlowfat	Allium vegetables	Garlic, leek, onion
veg_lettuce	Raw salad	Mixed side salad, lettuce, watercress
veg_mixed	Other vegetables (mushrooms, fruiting, mixed)	Mushrooms, mixed vegetables, avocado, broad beans, green beans, butternut squash, courgettes, , peppers, other
veg_mixtures_butter_dunno	Other vegetables (mushrooms, fruiting, mixed)	Mushrooms, mixed vegetables, avocado, broad beans, green beans, butternut squash, courgettes, , peppers, other

veg_mixtures_butter_fat	Other vegetables (mushrooms, fruiting, mixed)	Mushrooms, mixed vegetables, avocado, broad beans, green beans, butternut squash, courgettes, , peppers, other
veg_mixtures_butter_lowfat	Other vegetables (mushrooms, fruiting, mixed)	Mushrooms, mixed vegetables, avocado, broad beans, green beans, butternut squash, courgettes, , peppers, other
veg_mixtures_butter_spread_fat	Other vegetables (mushrooms, fruiting, mixed)	Mushrooms, mixed vegetables, avocado, broad beans, green beans, butternut squash, courgettes, , peppers, other
veg_mixtures_butter_spread_lowfat	Other vegetables (mushrooms, fruiting, mixed)	Mushrooms, mixed vegetables, avocado, broad beans, green beans, butternut squash, courgettes, , peppers, other
veg_mixtures_fat_dunno	Other vegetables (mushrooms, fruiting, mixed)	Mushrooms, mixed vegetables, avocado, broad beans, green beans, butternut squash, courgettes, , peppers, other
veg_mixtures_fat_none	Other vegetables (mushrooms, fruiting, mixed)	Mushrooms, mixed vegetables, avocado, broad beans, green beans, butternut squash, courgettes, , peppers, other
veg_mixtures_fat_other	Other vegetables (mushrooms, fruiting, mixed)	Mushrooms, mixed vegetables, avocado, broad beans, green beans, butternut squash, courgettes, , peppers, other
veg_mixtures_lard	Other vegetables (mushrooms, fruiting, mixed)	Mushrooms, mixed vegetables, avocado, broad beans, green beans, butternut squash, courgettes, , peppers, other
veg_mixtures_marg_hard	Other vegetables (mushrooms, fruiting, mixed)	Mushrooms, mixed vegetables, avocado, broad beans, green beans, butternut squash, courgettes, , peppers, other
veg_mixtures_marg_poly_chol	Other vegetables (mushrooms, fruiting, mixed)	Mushrooms, mixed vegetables, avocado, broad beans, green beans, butternut squash, courgettes, , peppers, other
veg_mixtures_marg_poly_dunno	Other vegetables (mushrooms, fruiting, mixed)	Mushrooms, mixed vegetables, avocado, broad beans, green beans, butternut squash, courgettes, , peppers, other
veg_mixtures_marg_poly_fat	Other vegetables (mushrooms, fruiting, mixed)	Mushrooms, mixed vegetables, avocado, broad beans, green beans, butternut squash, courgettes, , peppers, other
veg_mixtures_marg_poly_lowfat	Other vegetables (mushrooms, fruiting, mixed)	Mushrooms, mixed vegetables, avocado, broad beans, green beans, butternut squash, courgettes, , peppers, other
veg_mixtures_marg_poly_vlowfat	Other vegetables (mushrooms, fruiting, mixed)	Mushrooms, mixed vegetables, avocado, broad beans, green beans, butternut squash, courgettes, , peppers, other
veg_mixtures_marg_soya_chol	Other vegetables (mushrooms, fruiting, mixed)	Mushrooms, mixed vegetables, avocado, broad beans, green beans, butternut squash, courgettes, , peppers, other
veg_mixtures_marg_soya_dunno	Other vegetables (mushrooms, fruiting, mixed)	Mushrooms, mixed vegetables, avocado, broad beans, green beans, butternut squash, courgettes, , peppers, other
veg_mixtures_marg_soya_fat	Other vegetables (mushrooms, fruiting, mixed)	Mushrooms, mixed vegetables, avocado, broad beans, green beans, butternut squash, courgettes, , peppers, other
veg_mixtures_marg_soya_lowfat	Other vegetables (mushrooms, fruiting, mixed)	Mushrooms, mixed vegetables, avocado, broad beans, green beans, butternut squash, courgettes, , peppers, other
veg_mixtures_marg_soya_vlowfat	Other vegetables (mushrooms, fruiting, mixed)	Mushrooms, mixed vegetables, avocado, broad beans, green beans, butternut squash, courgettes, , peppers, other
veg_mixtures_oil_olive	Other vegetables (mushrooms, fruiting, mixed)	Mushrooms, mixed vegetables, avocado, broad beans, green beans, butternut squash, courgettes, , peppers, other
veg_mixtures_oil_other	Other vegetables (mushrooms, fruiting, mixed)	Mushrooms, mixed vegetables, avocado, broad beans, green beans, butternut squash, courgettes, , peppers, other
veg_mixtures_oil_rapeseed	Other vegetables (mushrooms, fruiting, mixed)	Mushrooms, mixed vegetables, avocado, broad beans, green beans, butternut squash, courgettes, , peppers, other
veg_mixtures_oil_sunflower	Other vegetables (mushrooms, fruiting, mixed)	Mushrooms, mixed vegetables, avocado, broad beans, green beans, butternut squash, courgettes, , peppers, other
veg_mixtures_oil_veg	Other vegetables (mushrooms, fruiting, mixed)	Mushrooms, mixed vegetables, avocado, broad beans, green beans, butternut squash, courgettes, , peppers, other
veg_mixtures_spread_dairy_chol	Other vegetables (mushrooms, fruiting, mixed)	Mushrooms, mixed vegetables, avocado, broad beans, green beans, butternut squash, courgettes, , peppers, other
veg_mixtures_spread_dairy_dunno	Other vegetables (mushrooms, fruiting, mixed)	Mushrooms, mixed vegetables, avocado, broad beans, green beans, butternut squash, courgettes, , peppers, other
veg_mixtures_spread_dairy_fat	Other vegetables (mushrooms, fruiting, mixed)	Mushrooms, mixed vegetables, avocado, broad beans, green beans, butternut squash, courgettes, , peppers, other
veg_mixtures_spread_dairy_lowfat	Other vegetables (mushrooms, fruiting, mixed)	Mushrooms, mixed vegetables, avocado, broad beans, green beans, butternut squash, courgettes, , peppers, other
veg_mixtures_spread_dairy_vlowfat	Other vegetables (mushrooms, fruiting, mixed)	Mushrooms, mixed vegetables, avocado, broad beans, green beans, butternut squash, courgettes, , peppers, other
veg_mixtures_spread_dunno_chol	Other vegetables (mushrooms, fruiting, mixed)	Mushrooms, mixed vegetables, avocado, broad beans, green beans, butternut squash, courgettes, , peppers, other
veg_mixtures_spread_dunno_dunno	Other vegetables (mushrooms, fruiting, mixed)	Mushrooms, mixed vegetables, avocado, broad beans, green beans, butternut squash, courgettes, , peppers, other
veg_mixtures_spread_dunno_fat	Other vegetables (mushrooms, fruiting, mixed)	Mushrooms, mixed vegetables, avocado, broad beans, green beans, butternut squash, courgettes, , peppers, other
veg_mixtures_spread_dunno_lowfat	Other vegetables (mushrooms, fruiting, mixed)	Mushrooms, mixed vegetables, avocado, broad beans, green beans, butternut squash, courgettes, , peppers, other
veg_mixtures_spread_dunno_vlowfat	Other vegetables (mushrooms, fruiting, mixed)	Mushrooms, mixed vegetables, avocado, broad beans, green beans, butternut squash, courgettes, , peppers, other
veg_mixtures_spread_olive_chol	Other vegetables (mushrooms, fruiting, mixed)	Mushrooms, mixed vegetables, avocado, broad beans, green beans, butternut squash, courgettes, , peppers, other
veg_mixtures_spread_olive_dunno	Other vegetables (mushrooms, fruiting, mixed)	Mushrooms, mixed vegetables, avocado, broad beans, green beans, butternut squash, courgettes, , peppers, other
veg_mixtures_spread_olive_fat	Other vegetables (mushrooms, fruiting, mixed)	Mushrooms, mixed vegetables, avocado, broad beans, green beans, butternut squash, courgettes, , peppers, other
veg_mixtures_spread_olive_lowfat	Other vegetables (mushrooms, fruiting, mixed)	Mushrooms, mixed vegetables, avocado, broad beans, green beans, butternut squash, courgettes, , peppers, other
veg_mixtures_spread_olive_vlowfat	Other vegetables (mushrooms, fruiting, mixed)	Mushrooms, mixed vegetables, avocado, broad beans, green beans, butternut squash, courgettes, , peppers, other
veg_mushrooms_butter_dunno	Other vegetables (mushrooms, fruiting, mixed)	Mushrooms, mixed vegetables, avocado, broad beans, green beans, butternut squash, courgettes, , peppers, other
veg_mushrooms_butter_fat	Other vegetables (mushrooms, fruiting, mixed)	Mushrooms, mixed vegetables, avocado, broad beans, green beans, butternut squash, courgettes, , peppers, other
veg_mushrooms_butter_lowfat	Other vegetables (mushrooms, fruiting, mixed)	Mushrooms, mixed vegetables, avocado, broad beans, green beans, butternut squash, courgettes, , peppers, other
veg_mushrooms_butter_spread_fat	Other vegetables (mushrooms, fruiting, mixed)	Mushrooms, mixed vegetables, avocado, broad beans, green beans, butternut squash, courgettes, , peppers, other
veg_mushrooms_butter_spread_lowfat	Other vegetables (mushrooms, fruiting, mixed)	Mushrooms, mixed vegetables, avocado, broad beans, green beans, butternut squash, courgettes, , peppers, other
veg_mushrooms_fat_dunno	Other vegetables (mushrooms, fruiting, mixed)	Mushrooms, mixed vegetables, avocado, broad beans, green beans, butternut squash, courgettes, , peppers, other
veg_mushrooms_fat_none	Other vegetables (mushrooms, fruiting, mixed)	Mushrooms, mixed vegetables, avocado, broad beans, green beans, butternut squash, courgettes, , peppers, other
veg_mushrooms_fat_other	Other vegetables (mushrooms, fruiting, mixed)	Mushrooms, mixed vegetables, avocado, broad beans, green beans, butternut squash, courgettes, , peppers, other
veg_mushrooms_lard	Other vegetables (mushrooms, fruiting, mixed)	Mushrooms, mixed vegetables, avocado, broad beans, green beans, butternut squash, courgettes, , peppers, other
veg_mushrooms_marg_hard	Other vegetables (mushrooms, fruiting, mixed)	Mushrooms, mixed vegetables, avocado, broad beans, green beans, butternut squash, courgettes, , peppers, other
veg_mushrooms_marg_poly_chol	Other vegetables (mushrooms, fruiting, mixed)	Mushrooms, mixed vegetables, avocado, broad beans, green beans, butternut squash, courgettes, , peppers, other
veg_mushrooms_marg_poly_dunno	Other vegetables (mushrooms, fruiting, mixed)	Mushrooms, mixed vegetables, avocado, broad beans, green beans, butternut squash, courgettes, , peppers, other
veg_mushrooms_marg_poly_fat	Other vegetables (mushrooms, fruiting, mixed)	Mushrooms, mixed vegetables, avocado, broad beans, green beans, butternut squash, courgettes, , peppers, other
veg_mushrooms_marg_poly_lowfat	Other vegetables (mushrooms, fruiting, mixed)	Mushrooms, mixed vegetables, avocado, broad beans, green beans, butternut squash, courgettes, , peppers, other
veg_mushrooms_marg_poly_vlowfat	Other vegetables (mushrooms, fruiting, mixed)	Mushrooms, mixed vegetables, avocado, broad beans, green beans, butternut squash, courgettes, , peppers, other
veg_mushrooms_marg_soya_chol	Other vegetables (mushrooms, fruiting, mixed)	Mushrooms, mixed vegetables, avocado, broad beans, green beans, butternut squash, courgettes, , peppers, other
veg_mushrooms_marg_soya_dunno	Other vegetables (mushrooms, fruiting, mixed)	Mushrooms, mixed vegetables, avocado, broad beans, green beans, butternut squash, courgettes, , peppers, other
veg_mushrooms_marg_soya_fat	Other vegetables (mushrooms, fruiting, mixed)	Mushrooms, mixed vegetables, avocado, broad beans, green beans, butternut squash, courgettes, , peppers, other
veg_mushrooms_marg_soya_lowfat	Other vegetables (mushrooms, fruiting, mixed)	Mushrooms, mixed vegetables, avocado, broad beans, green beans, butternut squash, courgettes, , peppers, other
veg_mushrooms_marg_soya_vlowfat	Other vegetables (mushrooms, fruiting, mixed)	Mushrooms, mixed vegetables, avocado, broad beans, green beans, butternut squash, courgettes, , peppers, other
veg_mushrooms_oil_olive	Other vegetables (mushrooms, fruiting, mixed)	Mushrooms, mixed vegetables, avocado, broad beans, green beans, butternut squash, courgettes, , peppers, other
veg_mushrooms_oil_other	Other vegetables (mushrooms, fruiting, mixed)	Mushrooms, mixed vegetables, avocado, broad beans, green beans, butternut squash, courgettes, , peppers, other
veg_mushrooms_oil_rapeseed	Other vegetables (mushrooms, fruiting, mixed)	Mushrooms, mixed vegetables, avocado, broad beans, green beans, butternut squash, courgettes, , peppers, other
veg_mushrooms_oil_sunflower	Other vegetables (mushrooms, fruiting, mixed)	Mushrooms, mixed vegetables, avocado, broad beans, green beans, butternut squash, courgettes, , peppers, other
veg_mushrooms_oil_veg	Other vegetables (mushrooms, fruiting, mixed)	Mushrooms, mixed vegetables, avocado, broad beans, green beans, butternut squash, courgettes, , peppers, other
veg_mushrooms_spread_dairy_chol	Other vegetables (mushrooms, fruiting, mixed)	Mushrooms, mixed vegetables, avocado, broad beans, green beans, butternut squash, courgettes, , peppers, other
veg_mushrooms_spread_dairy_dunno	Other vegetables (mushrooms, fruiting, mixed)	Mushrooms, mixed vegetables, avocado, broad beans, green beans, butternut squash, courgettes, , peppers, other
veg_mushrooms_spread_dairy_fat	Other vegetables (mushrooms, fruiting, mixed)	Mushrooms, mixed vegetables, avocado, broad beans, green beans, butternut squash, courgettes, , peppers, other
veg_mushrooms_spread_dairy_lowfat	Other vegetables (mushrooms, fruiting, mixed)	Mushrooms, mixed vegetables, avocado, broad beans, green beans, butternut squash, courgettes, , peppers, other
veg_mushrooms_spread_dairy_vlowfat	Other vegetables (mushrooms, fruiting, mixed)	Mushrooms, mixed vegetables, avocado, broad beans, green beans, butternut squash, courgettes, , peppers, other
veg_mushrooms_spread_dunno_chol	Other vegetables (mushrooms, fruiting, mixed)	Mushrooms, mixed vegetables, avocado, broad beans, green beans, butternut squash, courgettes, , peppers, other
veg_mushrooms_spread_dunno_dunno	Other vegetables (mushrooms, fruiting, mixed)	Mushrooms, mixed vegetables, avocado, broad beans, green beans, butternut squash, courgettes, , peppers, other
veg_mushrooms_spread_dunno_fat	Other vegetables (mushrooms, fruiting, mixed)	Mushrooms, mixed vegetables, avocado, broad beans, green beans, butternut squash, courgettes, , peppers, other
veg_mushrooms_spread_dunno_lowfat	Other vegetables (mushrooms, fruiting, mixed)	Mushrooms, mixed vegetables, avocado, broad beans, green beans, butternut squash, courgettes, , peppers, other
veg_mushrooms_spread_dunno_vlowfat	Other vegetables (mushrooms, fruiting, mixed)	Mushrooms, mixed vegetables, avocado, broad beans, green beans, butternut squash, courgettes, , peppers, other
veg_mushrooms_spread_olive_chol	Other vegetables (mushrooms, fruiting, mixed)	Mushrooms, mixed vegetables, avocado, broad beans, green beans, butternut squash, courgettes, , peppers, other
veg_mushrooms_spread_olive_dunno	Other vegetables (mushrooms, fruiting, mixed)	Mushrooms, mixed vegetables, avocado, broad beans, green beans, butternut squash, courgettes, , peppers, other
veg_mushrooms_spread_olive_fat	Other vegetables (mushrooms, fruiting, mixed)	Mushrooms, mixed vegetables, avocado, broad beans, green beans, butternut squash, courgettes, , peppers, other
veg_mushrooms_spread_olive_lowfat	Other vegetables (mushrooms, fruiting, mixed)	Mushrooms, mixed vegetables, avocado, broad beans, green beans, butternut squash, courgettes, , peppers, other
veg_mushrooms_spread_olive_vlowfat	Other vegetables (mushrooms, fruiting, mixed)	Mushrooms, mixed vegetables, avocado, broad beans, green beans, butternut squash, courgettes, , peppers, other
veg_onion_butter_dunno	Allium vegetables	Garlic, leek, onion
veg_onion_butter_fat	Allium vegetables	Garlic, leek, onion
veg_onion_butter_lowfat	Allium vegetables	Garlic, leek, onion
veg_onion_butter_spread_fat	Allium vegetables	Garlic, leek, onion
veg_onion_butter_spread_lowfat	Allium vegetables	Garlic, leek, onion
veg_onion_fat_dunno	Allium vegetables	Garlic, leek, onion
veg_onion_fat_none	Allium vegetables	Garlic, leek, onion
veg_onion_fat_other	Allium vegetables	Garlic, leek, onion
veg_onion_lard	Allium vegetables	Garlic, leek, onion
veg_onion_marg_hard	Allium vegetables	Garlic, leek, onion
veg_onion_marg_poly_chol	Allium vegetables	Garlic, leek, onion
veg_onion_marg_poly_dunno	Allium vegetables	Garlic, leek, onion
veg_onion_marg_poly_fat	Allium vegetables	Garlic, leek, onion
veg_onion_marg_poly_lowfat	Allium vegetables	Garlic, leek, onion
veg_onion_marg_poly_vlowfat	Allium vegetables	Garlic, leek, onion
veg_onion_marg_soya_chol	Allium vegetables	Garlic, leek, onion
veg_onion_marg_soya_dunno	Allium vegetables	Garlic, leek, onion
veg_onion_marg_soya_fat	Allium vegetables	Garlic, leek, onion
veg_onion_marg_soya_lowfat	Allium vegetables	Garlic, leek, onion
veg_onion_marg_soya_vlowfat	Allium vegetables	Garlic, leek, onion
veg_onion_oil_olive	Allium vegetables	Garlic, leek, onion
veg_onion_oil_other	Allium vegetables	Garlic, leek, onion
veg_onion_oil_rapeseed	Allium vegetables	Garlic, leek, onion
veg_onion_oil_sunflower	Allium vegetables	Garlic, leek, onion
veg_onion_oil_veg	Allium vegetables	Garlic, leek, onion
veg_onion_spread_dairy_chol	Allium vegetables	Garlic, leek, onion
veg_onion_spread_dairy_dunno	Allium vegetables	Garlic, leek, onion
veg_onion_spread_dairy_fat	Allium vegetables	Garlic, leek, onion
veg_onion_spread_dairy_lowfat	Allium vegetables	Garlic, leek, onion
veg_onion_spread_dairy_vlowfat	Allium vegetables	Garlic, leek, onion
veg_onion_spread_dunno_chol	Allium vegetables	Garlic, leek, onion
veg_onion_spread_dunno_dunno	Allium vegetables	Garlic, leek, onion
veg_onion_spread_dunno_fat	Allium vegetables	Garlic, leek, onion
veg_onion_spread_dunno_lowfat	Allium vegetables	Garlic, leek, onion
veg_onion_spread_dunno_vlowfat	Allium vegetables	Garlic, leek, onion
veg_onion_spread_olive_chol	Allium vegetables	Garlic, leek, onion
veg_onion_spread_olive_dunno	Allium vegetables	Garlic, leek, onion
veg_onion_spread_olive_fat	Allium vegetables	Garlic, leek, onion
veg_onion_spread_olive_lowfat	Allium vegetables	Garlic, leek, onion
veg_onion_spread_olive_vlowfat	Allium vegetables	Garlic, leek, onion
veg_other_butter_dunno	Other vegetables (mushrooms, fruiting, mixed)	Mushrooms, mixed vegetables, avocado, broad beans, green beans, butternut squash, courgettes, , peppers, other
veg_other_butter_fat	Other vegetables (mushrooms, fruiting, mixed)	Mushrooms, mixed vegetables, avocado, broad beans, green beans, butternut squash, courgettes, , peppers, other
veg_other_butter_lowfat	Other vegetables (mushrooms, fruiting, mixed)	Mushrooms, mixed vegetables, avocado, broad beans, green beans, butternut squash, courgettes, , peppers, other
veg_other_butter_spread_fat	Other vegetables (mushrooms, fruiting, mixed)	Mushrooms, mixed vegetables, avocado, broad beans, green beans, butternut squash, courgettes, , peppers, other
veg_other_butter_spread_lowfat	Other vegetables (mushrooms, fruiting, mixed)	Mushrooms, mixed vegetables, avocado, broad beans, green beans, butternut squash, courgettes, , peppers, other
veg_other_fat_dunno	Other vegetables (mushrooms, fruiting, mixed)	Mushrooms, mixed vegetables, avocado, broad beans, green beans, butternut squash, courgettes, , peppers, other
veg_other_fat_none	Other vegetables (mushrooms, fruiting, mixed)	Mushrooms, mixed vegetables, avocado, broad beans, green beans, butternut squash, courgettes, , peppers, other
veg_other_fat_other	Other vegetables (mushrooms, fruiting, mixed)	Mushrooms, mixed vegetables, avocado, broad beans, green beans, butternut squash, courgettes, , peppers, other
veg_other_lard	Other vegetables (mushrooms, fruiting, mixed)	Mushrooms, mixed vegetables, avocado, broad beans, green beans, butternut squash, courgettes, , peppers, other
veg_other_marg_hard	Other vegetables (mushrooms, fruiting, mixed)	Mushrooms, mixed vegetables, avocado, broad beans, green beans, butternut squash, courgettes, , peppers, other
veg_other_marg_poly_chol	Other vegetables (mushrooms, fruiting, mixed)	Mushrooms, mixed vegetables, avocado, broad beans, green beans, butternut squash, courgettes, , peppers, other
veg_other_marg_poly_dunno	Other vegetables (mushrooms, fruiting, mixed)	Mushrooms, mixed vegetables, avocado, broad beans, green beans, butternut squash, courgettes, , peppers, other
veg_other_marg_poly_fat	Other vegetables (mushrooms, fruiting, mixed)	Mushrooms, mixed vegetables, avocado, broad beans, green beans, butternut squash, courgettes, , peppers, other
veg_other_marg_poly_lowfat	Other vegetables (mushrooms, fruiting, mixed)	Mushrooms, mixed vegetables, avocado, broad beans, green beans, butternut squash, courgettes, , peppers, other
veg_other_marg_poly_vlowfat	Other vegetables (mushrooms, fruiting, mixed)	Mushrooms, mixed vegetables, avocado, broad beans, green beans, butternut squash, courgettes, , peppers, other
veg_other_marg_soya_chol	Other vegetables (mushrooms, fruiting, mixed)	Mushrooms, mixed vegetables, avocado, broad beans, green beans, butternut squash, courgettes, , peppers, other
veg_other_marg_soya_dunno	Other vegetables (mushrooms, fruiting, mixed)	Mushrooms, mixed vegetables, avocado, broad beans, green beans, butternut squash, courgettes, , peppers, other
veg_other_marg_soya_fat	Other vegetables (mushrooms, fruiting, mixed)	Mushrooms, mixed vegetables, avocado, broad beans, green beans, butternut squash, courgettes, , peppers, other
veg_other_marg_soya_lowfat	Other vegetables (mushrooms, fruiting, mixed)	Mushrooms, mixed vegetables, avocado, broad beans, green beans, butternut squash, courgettes, , peppers, other
veg_other_marg_soya_vlowfat	Other vegetables (mushrooms, fruiting, mixed)	Mushrooms, mixed vegetables, avocado, broad beans, green beans, butternut squash, courgettes, , peppers, other
veg_other_oil_olive	Other vegetables (mushrooms, fruiting, mixed)	Mushrooms, mixed vegetables, avocado, broad beans, green beans, butternut squash, courgettes, , peppers, other
veg_other_oil_other	Other vegetables (mushrooms, fruiting, mixed)	Mushrooms, mixed vegetables, avocado, broad beans, green beans, butternut squash, courgettes, , peppers, other
veg_other_oil_rapeseed	Other vegetables (mushrooms, fruiting, mixed)	Mushrooms, mixed vegetables, avocado, broad beans, green beans, butternut squash, courgettes, , peppers, other
veg_other_oil_sunflower	Other vegetables (mushrooms, fruiting, mixed)	Mushrooms, mixed vegetables, avocado, broad beans, green beans, butternut squash, courgettes, , peppers, other
veg_other_oil_veg	Other vegetables (mushrooms, fruiting, mixed)	Mushrooms, mixed vegetables, avocado, broad beans, green beans, butternut squash, courgettes, , peppers, other
veg_other_spread_dairy_chol	Other vegetables (mushrooms, fruiting, mixed)	Mushrooms, mixed vegetables, avocado, broad beans, green beans, butternut squash, courgettes, , peppers, other
veg_other_spread_dairy_dunno	Other vegetables (mushrooms, fruiting, mixed)	Mushrooms, mixed vegetables, avocado, broad beans, green beans, butternut squash, courgettes, , peppers, other
veg_other_spread_dairy_fat	Other vegetables (mushrooms, fruiting, mixed)	Mushrooms, mixed vegetables, avocado, broad beans, green beans, butternut squash, courgettes, , peppers, other
veg_other_spread_dairy_lowfat	Other vegetables (mushrooms, fruiting, mixed)	Mushrooms, mixed vegetables, avocado, broad beans, green beans, butternut squash, courgettes, , peppers, other
veg_other_spread_dairy_vlowfat	Other vegetables (mushrooms, fruiting, mixed)	Mushrooms, mixed vegetables, avocado, broad beans, green beans, butternut squash, courgettes, , peppers, other
veg_other_spread_dunno_chol	Other vegetables (mushrooms, fruiting, mixed)	Mushrooms, mixed vegetables, avocado, broad beans, green beans, butternut squash, courgettes, , peppers, other
veg_other_spread_dunno_dunno	Other vegetables (mushrooms, fruiting, mixed)	Mushrooms, mixed vegetables, avocado, broad beans, green beans, butternut squash, courgettes, , peppers, other
veg_other_spread_dunno_fat	Other vegetables (mushrooms, fruiting, mixed)	Mushrooms, mixed vegetables, avocado, broad beans, green beans, butternut squash, courgettes, , peppers, other

veg_other_spread_dunno_lowfat	Other vegetables (mushrooms, fruiting, mixed)	Mushrooms, mixed vegetables, avocado, broad beans, green beans, butternut squash, courgettes, , peppers, other
veg_other_spread_dunno_vlowfat	Other vegetables (mushrooms, fruiting, mixed)	Mushrooms, mixed vegetables, avocado, broad beans, green beans, butternut squash, courgettes, , peppers, other
veg_other_spread_olive_chol	Other vegetables (mushrooms, fruiting, mixed)	Mushrooms, mixed vegetables, avocado, broad beans, green beans, butternut squash, courgettes, , peppers, other
veg_other_spread_olive_dunno	Other vegetables (mushrooms, fruiting, mixed)	Mushrooms, mixed vegetables, avocado, broad beans, green beans, butternut squash, courgettes, , peppers, other
veg_other_spread_olive_fat	Other vegetables (mushrooms, fruiting, mixed)	Mushrooms, mixed vegetables, avocado, broad beans, green beans, butternut squash, courgettes, , peppers, other
veg_other_spread_olive_lowfat	Other vegetables (mushrooms, fruiting, mixed)	Mushrooms, mixed vegetables, avocado, broad beans, green beans, butternut squash, courgettes, , peppers, other
veg_other_spread_olive_vlowfat	Other vegetables (mushrooms, fruiting, mixed)	Mushrooms, mixed vegetables, avocado, broad beans, green beans, butternut squash, courgettes, , peppers, other
veg_parsnip_butter_dunno	Root vegetables	Beetroot, carrots, celery, parsnip, turnip
veg_parsnip_butter_fat	Root vegetables	Beetroot, carrots, celery, parsnip, turnip
veg_parsnip_butter_lowfat	Root vegetables	Beetroot, carrots, celery, parsnip, turnip
veg_parsnip_butter_spread_fat	Root vegetables	Beetroot, carrots, celery, parsnip, turnip
veg_parsnip_butter_spread_lowfat	Root vegetables	Beetroot, carrots, celery, parsnip, turnip
veg_parsnip_fat_dunno	Root vegetables	Beetroot, carrots, celery, parsnip, turnip
veg_parsnip_fat_none	Root vegetables	Beetroot, carrots, celery, parsnip, turnip
veg_parsnip_fat_other	Root vegetables	Beetroot, carrots, celery, parsnip, turnip
veg_parsnip_lard	Root vegetables	Beetroot, carrots, celery, parsnip, turnip
veg_parsnip_marg_hard	Root vegetables	Beetroot, carrots, celery, parsnip, turnip
veg_parsnip_marg_poly_chol	Root vegetables	Beetroot, carrots, celery, parsnip, turnip
veg_parsnip_marg_poly_dunno	Root vegetables	Beetroot, carrots, celery, parsnip, turnip
veg_parsnip_marg_poly_fat	Root vegetables	Beetroot, carrots, celery, parsnip, turnip
veg_parsnip_marg_poly_lowfat	Root vegetables	Beetroot, carrots, celery, parsnip, turnip
veg_parsnip_marg_poly_vlowfat	Root vegetables	Beetroot, carrots, celery, parsnip, turnip
veg_parsnip_marg_soya_chol	Root vegetables	Beetroot, carrots, celery, parsnip, turnip
veg_parsnip_marg_soya_dunno	Root vegetables	Beetroot, carrots, celery, parsnip, turnip
veg_parsnip_marg_soya_fat	Root vegetables	Beetroot, carrots, celery, parsnip, turnip
veg_parsnip_marg_soya_lowfat	Root vegetables	Beetroot, carrots, celery, parsnip, turnip
veg_parsnip_marg_soya_vlowfat	Root vegetables	Beetroot, carrots, celery, parsnip, turnip
veg_parsnip_oil_olive	Root vegetables	Beetroot, carrots, celery, parsnip, turnip
veg_parsnip_oil_other	Root vegetables	Beetroot, carrots, celery, parsnip, turnip
veg_parsnip_oil_rapeseed	Root vegetables	Beetroot, carrots, celery, parsnip, turnip
veg_parsnip_oil_sunflower	Root vegetables	Beetroot, carrots, celery, parsnip, turnip
veg_parsnip_oil_veg	Root vegetables	Beetroot, carrots, celery, parsnip, turnip
veg_parsnip_spread_dairy_chol	Root vegetables	Beetroot, carrots, celery, parsnip, turnip
veg_parsnip_spread_dairy_dunno	Root vegetables	Beetroot, carrots, celery, parsnip, turnip
veg_parsnip_spread_dairy_fat	Root vegetables	Beetroot, carrots, celery, parsnip, turnip
veg_parsnip_spread_dairy_lowfat	Root vegetables	Beetroot, carrots, celery, parsnip, turnip
veg_parsnip_spread_dairy_vlowfat	Root vegetables	Beetroot, carrots, celery, parsnip, turnip
veg_parsnip_spread_dunno_chol	Root vegetables	Beetroot, carrots, celery, parsnip, turnip
veg_parsnip_spread_dunno_dunno	Root vegetables	Beetroot, carrots, celery, parsnip, turnip
veg_parsnip_spread_dunno_fat	Root vegetables	Beetroot, carrots, celery, parsnip, turnip
veg_parsnip_spread_dunno_lowfat	Root vegetables	Beetroot, carrots, celery, parsnip, turnip
veg_parsnip_spread_dunno_vlowfat	Root vegetables	Beetroot, carrots, celery, parsnip, turnip
veg_parsnip_spread_olive_chol	Root vegetables	Beetroot, carrots, celery, parsnip, turnip
veg_parsnip_spread_olive_dunno	Root vegetables	Beetroot, carrots, celery, parsnip, turnip
veg_parsnip_spread_olive_fat	Root vegetables	Beetroot, carrots, celery, parsnip, turnip
veg_parsnip_spread_olive_lowfat	Root vegetables	Beetroot, carrots, celery, parsnip, turnip
veg_parsnip_spread_olive_vlowfat	Root vegetables	Beetroot, carrots, celery, parsnip, turnip
veg_peas	Peas/sweetcorn	Peas, sweetcorn
veg_pepper_bell_butter_dunno	Other vegetables (mushrooms, fruiting, mixed)	Mushrooms, mixed vegetables, avocado, broad beans, green beans, butternut squash, courgettes, , peppers, other
veg_pepper_bell_butter_fat	Other vegetables (mushrooms, fruiting, mixed)	Mushrooms, mixed vegetables, avocado, broad beans, green beans, butternut squash, courgettes, , peppers, other
veg_pepper_bell_butter_lowfat	Other vegetables (mushrooms, fruiting, mixed)	Mushrooms, mixed vegetables, avocado, broad beans, green beans, butternut squash, courgettes, , peppers, other
veg_pepper_bell_butter_spread_fat	Other vegetables (mushrooms, fruiting, mixed)	Mushrooms, mixed vegetables, avocado, broad beans, green beans, butternut squash, courgettes, , peppers, other
veg_pepper_bell_butter_spread_lowfat	Other vegetables (mushrooms, fruiting, mixed)	Mushrooms, mixed vegetables, avocado, broad beans, green beans, butternut squash, courgettes, , peppers, other
veg_pepper_bell_fat_dunno	Other vegetables (mushrooms, fruiting, mixed)	Mushrooms, mixed vegetables, avocado, broad beans, green beans, butternut squash, courgettes, , peppers, other
veg_pepper_bell_fat_none	Other vegetables (mushrooms, fruiting, mixed)	Mushrooms, mixed vegetables, avocado, broad beans, green beans, butternut squash, courgettes, , peppers, other
veg_pepper_bell_fat_other	Other vegetables (mushrooms, fruiting, mixed)	Mushrooms, mixed vegetables, avocado, broad beans, green beans, butternut squash, courgettes, , peppers, other
veg_pepper_bell_lard	Other vegetables (mushrooms, fruiting, mixed)	Mushrooms, mixed vegetables, avocado, broad beans, green beans, butternut squash, courgettes, , peppers, other
veg_pepper_bell_marg_hard	Other vegetables (mushrooms, fruiting, mixed)	Mushrooms, mixed vegetables, avocado, broad beans, green beans, butternut squash, courgettes, , peppers, other
veg_pepper_bell_marg_poly_chol	Other vegetables (mushrooms, fruiting, mixed)	Mushrooms, mixed vegetables, avocado, broad beans, green beans, butternut squash, courgettes, , peppers, other
veg_pepper_bell_marg_poly_dunno	Other vegetables (mushrooms, fruiting, mixed)	Mushrooms, mixed vegetables, avocado, broad beans, green beans, butternut squash, courgettes, , peppers, other
veg_pepper_bell_marg_poly_fat	Other vegetables (mushrooms, fruiting, mixed)	Mushrooms, mixed vegetables, avocado, broad beans, green beans, butternut squash, courgettes, , peppers, other
veg_pepper_bell_marg_poly_lowfat	Other vegetables (mushrooms, fruiting, mixed)	Mushrooms, mixed vegetables, avocado, broad beans, green beans, butternut squash, courgettes, , peppers, other
veg_pepper_bell_marg_poly_vlowfat	Other vegetables (mushrooms, fruiting, mixed)	Mushrooms, mixed vegetables, avocado, broad beans, green beans, butternut squash, courgettes, , peppers, other
veg_pepper_bell_marg_soya_chol	Other vegetables (mushrooms, fruiting, mixed)	Mushrooms, mixed vegetables, avocado, broad beans, green beans, butternut squash, courgettes, , peppers, other
veg_pepper_bell_marg_soya_dunno	Other vegetables (mushrooms, fruiting, mixed)	Mushrooms, mixed vegetables, avocado, broad beans, green beans, butternut squash, courgettes, , peppers, other
veg_pepper_bell_marg_soya_fat	Other vegetables (mushrooms, fruiting, mixed)	Mushrooms, mixed vegetables, avocado, broad beans, green beans, butternut squash, courgettes, , peppers, other
veg_pepper_bell_marg_soya_lowfat	Other vegetables (mushrooms, fruiting, mixed)	Mushrooms, mixed vegetables, avocado, broad beans, green beans, butternut squash, courgettes, , peppers, other
veg_pepper_bell_marg_soya_vlowfat	Other vegetables (mushrooms, fruiting, mixed)	Mushrooms, mixed vegetables, avocado, broad beans, green beans, butternut squash, courgettes, , peppers, other
veg_pepper_bell_oil_olive	Other vegetables (mushrooms, fruiting, mixed)	Mushrooms, mixed vegetables, avocado, broad beans, green beans, butternut squash, courgettes, , peppers, other
veg_pepper_bell_oil_other	Other vegetables (mushrooms, fruiting, mixed)	Mushrooms, mixed vegetables, avocado, broad beans, green beans, butternut squash, courgettes, , peppers, other
veg_pepper_bell_oil_rapeseed	Other vegetables (mushrooms, fruiting, mixed)	Mushrooms, mixed vegetables, avocado, broad beans, green beans, butternut squash, courgettes, , peppers, other
veg_pepper_bell_oil_sunflower	Other vegetables (mushrooms, fruiting, mixed)	Mushrooms, mixed vegetables, avocado, broad beans, green beans, butternut squash, courgettes, , peppers, other
veg_pepper_bell_oil_veg	Other vegetables (mushrooms, fruiting, mixed)	Mushrooms, mixed vegetables, avocado, broad beans, green beans, butternut squash, courgettes, , peppers, other
veg_pepper_bell_spread_dairy_chol	Other vegetables (mushrooms, fruiting, mixed)	Mushrooms, mixed vegetables, avocado, broad beans, green beans, butternut squash, courgettes, , peppers, other
veg_pepper_bell_spread_dairy_dunno	Other vegetables (mushrooms, fruiting, mixed)	Mushrooms, mixed vegetables, avocado, broad beans, green beans, butternut squash, courgettes, , peppers, other
veg_pepper_bell_spread_dairy_fat	Other vegetables (mushrooms, fruiting, mixed)	Mushrooms, mixed vegetables, avocado, broad beans, green beans, butternut squash, courgettes, , peppers, other
veg_pepper_bell_spread_dairy_lowfat	Other vegetables (mushrooms, fruiting, mixed)	Mushrooms, mixed vegetables, avocado, broad beans, green beans, butternut squash, courgettes, , peppers, other
veg_pepper_bell_spread_dairy_vlowfat	Other vegetables (mushrooms, fruiting, mixed)	Mushrooms, mixed vegetables, avocado, broad beans, green beans, butternut squash, courgettes, , peppers, other
veg_pepper_bell_spread_dunno_chol	Other vegetables (mushrooms, fruiting, mixed)	Mushrooms, mixed vegetables, avocado, broad beans, green beans, butternut squash, courgettes, , peppers, other
veg_pepper_bell_spread_dunno_dunno	Other vegetables (mushrooms, fruiting, mixed)	Mushrooms, mixed vegetables, avocado, broad beans, green beans, butternut squash, courgettes, , peppers, other
veg_pepper_bell_spread_dunno_fat	Other vegetables (mushrooms, fruiting, mixed)	Mushrooms, mixed vegetables, avocado, broad beans, green beans, butternut squash, courgettes, , peppers, other
veg_pepper_bell_spread_dunno_lowfat	Other vegetables (mushrooms, fruiting, mixed)	Mushrooms, mixed vegetables, avocado, broad beans, green beans, butternut squash, courgettes, , peppers, other
veg_pepper_bell_spread_dunno_vlowfat	Other vegetables (mushrooms, fruiting, mixed)	Mushrooms, mixed vegetables, avocado, broad beans, green beans, butternut squash, courgettes, , peppers, other
veg_pepper_bell_spread_olive_chol	Other vegetables (mushrooms, fruiting, mixed)	Mushrooms, mixed vegetables, avocado, broad beans, green beans, butternut squash, courgettes, , peppers, other
veg_pepper_bell_spread_olive_dunno	Other vegetables (mushrooms, fruiting, mixed)	Mushrooms, mixed vegetables, avocado, broad beans, green beans, butternut squash, courgettes, , peppers, other
veg_pepper_bell_spread_olive_fat	Other vegetables (mushrooms, fruiting, mixed)	Mushrooms, mixed vegetables, avocado, broad beans, green beans, butternut squash, courgettes, , peppers, other
veg_pepper_bell_spread_olive_lowfat	Other vegetables (mushrooms, fruiting, mixed)	Mushrooms, mixed vegetables, avocado, broad beans, green beans, butternut squash, courgettes, , peppers, other
veg_pepper_bell_spread_olive_vlowfat	Other vegetables (mushrooms, fruiting, mixed)	Mushrooms, mixed vegetables, avocado, broad beans, green beans, butternut squash, courgettes, , peppers, other
veg_pulses	Legumes/pulses	Baked beans, pulses
veg_saladmayo	Vegetable side dishes	Coleslaw, salad with added fat/mayonnaise
veg_sidesalad	Raw salad	Mixed side salad, lettuce, watercress
veg_spinach	Green leafy/cabbages	Broccoli, cabbage, kale, cauliflower, spinach, sprouts
veg_sprouts	Green leafy/cabbages	Broccoli, cabbage, kale, cauliflower, spinach, sprouts
veg_sweetcorn	Peas/sweetcorn	Peas, sweetcorn
veg_sweetpot	Potatoes/sweet potatoes (baked/boiled)	Potatoes, sweet potatoes, boiled or baked
veg_tomato_fresh	Tomatoes	Fresh and tinned tomatoes
veg_tomato_tinned	Tomatoes	Fresh and tinned tomatoes
veg_turnip	Root vegetables	Beetroot, carrots, celery, parsnip, turnip
veg_watercress	Raw salad	Mixed side salad, lettuce, watercress
vegalt_burger	Vegetarian meals	Quorn-based and vegetarian burgers and products
vegalt_other	Vegetarian meals	Quorn-based and vegetarian burgers and products
vegalt_quorn	Vegetarian meals	Quorn-based and vegetarian burgers and products
vegalt_tofu	Soy-based meals	Tofu-based products
yeast_extract	Sauces (lower fat)	Yeast, chutney, olives, ketchup, brown sauce, tomato sauce
yogurt_fullfat	Full fat yogurt	Whole milk yogurt (plain)
yogurt_lowfat	Low fat yogurt	Fat free and lower fat yogurt, plain or flavoured
yorkshirepud	Grain dishes - added fat	Double and single crust pies, crumble pies, Yorkshire pudding, snackpot noodles
